# Complete chloroplast genome sequences of *Phlomis fruticosa* and *Phlomoides strigosa* and comparative analysis of the genus *Phlomis* sensu lato (Lamiaceae)

**DOI:** 10.3389/fpls.2022.1022273

**Published:** 2022-10-28

**Authors:** Wei Zhao, Lirong Guo, Yu Yang, Yan Wang, Li Yang, Cuimei Wei, Jian Guo, Kan Yan, Haijuan Chen, Zhigang Yang, Yimeng Li

**Affiliations:** ^1^ School of Pharmacy, Lanzhou University, Lanzhou, China; ^2^ School of Biological and Pharmaceutical Engineering, Lanzhou Jiaotong University, Lanzhou, China; ^3^ Key Laboratory of Medicinal Animal and Plant Resources of Qinghai-Tibetan Plateau, Academy of Plateau Science and Sustainability, Qinghai Normal University, Xining, China; ^4^ RIKEN Center for Sustainable Resource Science, Yokohama, Kanagawa, Japan

**Keywords:** Lamiaceae, tribe Phlomideae, *Phlomis*, *Phlomoides*, independent genera, chloroplast genome

## Abstract

The taxonomic terms “*Phlomis*” and “*Phlomoides*” had been used to describe two sections within the genus *Phlomis* belonging to the Lamiaceae family. Recently, phylogenetic analyses using molecular markers showed that *Phlomis* and *Phlomoides* formed two monophyletic clades, and thus they are generally accepted as separate genera. In this study, we assembled the complete chloroplast genome of *Phlomis fruticosa*, which is the first reported chloroplast genome belonging to *Phlomis* genus, as well as the complete chloroplast genome of *Phlomoides strigosa* belonging to *Phlomoides* genus. The results showed that the length of chloroplast genome was 151,639 bp (*Phlomis fruticosa*) and 152,432 bp (*Phlomoides strigosa*), with conserved large single copy regions, small single copy regions, and inverted repeat regions. 121 genes in *Phlomis fruticosa* and 120 genes in *Phlomoides strigosa* were annotated. The chloroplast genomes of *Phlomis fruticosa*, *Phlomoides strigosa*, and three reported *Phlomoides* species, as well as those of 51 species from the Lamiaceae family, which covered 12 subfamilies, were subjected to phylogenetic analyses. The *Phlomis* and *Phlomoides* species were split into two groups, which were well supported by both maximum likelihood and Bayesian inference tree analyses. Our study provided further evidence to recognize *Phlomis* and *Phlomoides* as independent genera.

## Introduction

The genus *Phlomis* L. belonging to the Lamiaceae family was proposed by Linnaeus (1753). Moench (1794) recognized different morphology of corollas and fruits within this genus, which then was divided into two genera. This taxonomy was accepted and developed by some researchers (Adylov et al., 1986; Adylov and Makhmedov, 1987; Kamelin and Makhmedov, 1990a; Kamelin and Makhmedov, 1990b; Scheen et al., 2010; Mathiesen et al., 2011; Salmaki et al, 2012; Xiang et al., 2014; Zhao et al., 2021). By contrast, some taxonomists recognized *Phlomis* and *Phlomoides* as two sections within the genus *Phlomis* (i.e., sect. *Phlomis* and sect. *Phlomoides*) (Bentham, 1832-1836; Boissier, 1879; Briquet, 1895-1897; Rechinger, 1982). Many morphological variations were observed between these two groups. For example, most *Phlomis* plants are perennial herbs or small shrubs, leaves are lanceolate to oblong-lanceolate, leaf venation is fan-shaped, the upper lips of corolla are laterally compressed, flattened, and sickle-shaped, and verticillasters are in a dense scapose capitulum or short spike; while most *Phlomoides* plants are perennial herbs with woody rhizomes and/or tubers, leaves are cordate to triangular-ovate, leaf venation is not fan-shaped, the upper lips of corolla are always hairy or fringed-incised, and verticillasters are axillary, lax, or dense (Mathiesen et al., 2011; Salmaki et al., 2012; Xiang et al., 2014). Anatomy, cytology, and phytochemistry analyses also revealed distinct features between *Phlomis* and *Phlomoides*. The chromosome number of *Phlomis* plants is 2*n* = 20, while that of *Phlomoides* plants is 2*n* = 22 (Azizian and Cutler, 1982). In the last two decades, phylogenetic studies based on molecular markers, including nuclear internal transcribed spacer (ITS) and chloroplast sequence (including *rpl16*, *trnL*-*F*, *trnL* intron, *trnL*-*F* intergenic spacer, *rps16* intron, partial *trnK*, *rpl32*-*trnL*, *trnT*-*A*, and *matK*), supported a split of *Phlomis* s.l. into two monophyletic clades (Pan et al., 2009; Scheen et al., 2010; Bendiksby et al., 2011; Mathiesen et al., 2011; Salmaki et al, 2012; Xiang et al., 2014; Zhao et al., 2021). Therefore, most taxonomists accepted that *Phlomis* and *Phlomoides* should be treated as separate genera, i.e., *Phlomis* s.str. and *Phlomoides* Moench.

In recent years, numerous chloroplast genomes have been published. Comparative analysis of complete chloroplast genomes provided the phylogeny with high resolution to investigate the intergeneric and infrageneric relationships of several genera, including *Pogostemon* (Lamiaceae) (He et al., 2016), *Isodon* (Lamiaceae) (Lian et al., 2022), *Chenopodium* (Amaranthaceae) (Hong et al., 2017), *Viola* (Violaceae) (Cheon et al., 2019), *Curcuma* (Zingiberaceae) (Liang et al., 2020), *Hibiscus* (Malvaceae) (Abdullah et al., 2020; Cheng et al., 2020), *Salvadora* (Salvadoraceae) (Khan et al., 2021), *Edgeworthia* (Thymelaeaceae) (Qian et al., 2021), *Fragaria* (Rosaceae) (Sun et al, 2021), etc. Due to the lack of complete nuclear genomes of most Lamiaceae plants at present, phylogenetic studies using the complete chloroplast genomes is an effective approach to investigate the evolutionary relationships of *Phlomis* and *Phlomoides*.

*Phlomis fruticosa* L. is distributed in the Mediterranean region. The extracts of *Phlomis fruticosa* was reported to exhibit antimicrobial, antioxidant, and antineurodegenerative activities, and protective effect on colon tissues (Ristíc et al., 2000; Ferrante et al., 2019; Stojkovic et al., 2021; Stojkovic et al., 2022). As a representative species of *Phlomis*, the ITS and chloroplast markers of *Phlomis fruticosa* was commonly used in previous phylogenetic studies. In the present study, we sequenced and assembled the complete chloroplast genomes of *Phlomis fruticosa* as well as *Phlomoides strigosa*, which belongs to the *Phlomoides*. Our phylogenetic analysis with published chloroplast genomes of three *Phlomoides* and another 51 Lamiaceae species confirmed that *Phlomoides* was monophyletic, and supported the treatment of independent genera of *Phlomis* and *Phlomoides*.

## Materials and methods

### Plant materials

The plants of *Phlomis fruticosa* and *Phlomoides strigosa* were collected from Sichuan province of China and cultivated in our lab. The morphological characteristics of plants were authorized by Prof. Yinsuo Zhou from Lanzhou University. The fresh leaves of plant materials were sampled and quickly frozen in liquid nitrogen. All sampled were stored at -80°C until use. The sample collection was complied with the national and international legislation and guidelines.

### DNA extraction and sequencing

Total genomic DNA was isolated from leaves utilizing a DNeasy plant DNA extraction kit (Qiagen, CA, USA) following the manufacturer’s guidelines. The integrity of the extracted total genomic DNA was detected by 1% agarose gel electrophoresis, and then the qualified sample were selected for subsequent experiments.

After the samples were qualified, sequencing libraries were constructed by means of purified the fragment, repaired the terminal, added with A in 3’ segment connected with sequencing connector, PCR amplification, etc. The library type is the DNA small fragment library of 250 bp. DNA sequencing was performed by an Illumina NovaSeq platform pair-end sequencing method at Benagen Technology Services Limited (Wuhan, China).

### Chloroplast genome assembly and gene annotation

Quality control of raw sequences reads was carried out using FastQC (Andrews, 2010). The low quality and redundant reads (Q < 20) were trimmed using Samtools (Li et al., 2009). A *de novo* assembly of the chloroplast genomes was implemented by using the GetOrganele (Jin et al., 2020). Chloroplast reads were extracted from total genomic reads, and SPAdes (Bankevich et al., 2012) and A5-miseq (Coil et al., 2015) were used to assemble chloroplast genomes. After the initial assembly, several assembly results were obtained, in which multiple contigs/scaffolds sequences were found. After the eligible sequences were selected, scaffolds were connected according to the overlapping regions. If there was any gap in sequences, another sequence would be used to fix the gaps. After obtaining the complete chloroplast genome sequences of *Phlomis fruticosa* and *Phlomoides strigosa*, gene annotation was performed using the CPGAVAS2 (Shi et al., 2019) with default parameters. After the initial annotation, several items were checked, include fake gene, missing of termination codon or initiation codon. The physical chloroplast genome map was drawn using the Chloroplot program (Zheng et al., 2020) with default parameters.

### Condon usage bias analysis

The protein-coding genes were extracted from the five chloroplast genomes for codon analysis. The relative synonymous codon usage (RSCU) values (Sharp and Li, 1986) were calculated in the program CodonW v1.4.2 (https://sourceforge.net/projects/codonw/). A total of 44 shared protein-coding genes (CDS) were screened out from five species following the guidance of removing CDS smaller than 300 bp (Wright, 1990) and the overlapping genes. The effective number of codons (ENc) and GC content of the synonymous third codons positions (GC3s) values were calculated by using CodonW v1.4.2 to assess the extent of influence of mutation bias and natural selection on CDS.

### Long repeats, simple sequence repeats, and genome comparison

The REPuter (Kurtz et al., 2001) was used to detect the long repeats with default parameters. Simple sequence repeat analysis was performed on the assembled chloroplast sequence genomes using MISA tools (Beier et al., 2017), with a threshold of 8 for mono-nucleotide, 4 for di-nucleotide, 3 for tri-nucleotide, and 3 for tetra-nucleotide, penta-nucleotide, and hexa-nucleotide simple sequence repeats. The minimum distance between two SSRs was set to 0 bp.

For genome comparison, the chloroplast genomic sequences alignment was carried out by ClustalW (Larkin et al., 2007). The mVISTA (Mayor et al., 2000) program in the Shuffle-LAGAN mode was used to compare the whole chloroplast genomes of *P. fruticosa*, *P. strigosa*, *Phlomoides alpina*, *Phlomoides betonicoides*, and *Phlomoides younghushandii*. The chloroplast genome sequence of *P. alpina* was used as the reference sequence. The boundaries of large single-copy (LSC) region, inverted repeated (IR) regions, and small single-copy (SSC) region of the five species were mapped using IRscope (Amiryousefi et al., 2018) program.

### Phylogenetic analysis

The complete chloroplast genomes of 51 species belonging to the Lamiaceae family available from NCBI were downloaded. These species covered 12 subfamilies of Lamiaceae, and the detailed information was listed in **Table S1**. Four species including *Lancea hirsuta*, *Lancea tibetica*, *Erythranthe lutea* and *Paulownia coreana* were selected as outgroups based on previous phylogenetic study (Zhao et al, 2019). Since noncoding regions can be variable even among species and are often difficult to align across a family as large as Lamiaceae, protein-coding genes were selected for phylogenetic analyses. Alignments of sequences were performed using the MAFFT (Katoh and Standley, 2013). Since the chloroplast is uniparentally inherited in most angiosperms and generally does not undergo recombination, sequences of the coding genes were concatenated in our study to generate a supermatrix of all coding regions. Phylogenetic trees based on all datasets were built by Bayesian inference (BI) analysis or maximum likelihood (ML) analysis. PhyloSuite (Zhang et al., 2020) was used to determine the best-fit models for nucleotide sequences for BI analyses and the JTT+F+I+G4 model was used to perform Bayesian analyses. ML analyses were performed using RAxML (Stamatakis, 2014). The GTR+G+I model was used for analyses and bootstrapping. Bootstrap iterations were set to 1000. According to previous study (Zhao et al, 2021), the branches with posterior probabilities (PP) < 0.90 and bootstrap values (BS) < 70% as weakly support, PP = 0.90–0.95 and BS = 70%–80% as moderately supported, and PP ≥ 0.95 and BS ≥ 80% as strongly supported.

## Results

### Features of chloroplast genomes

**Figure 1:**
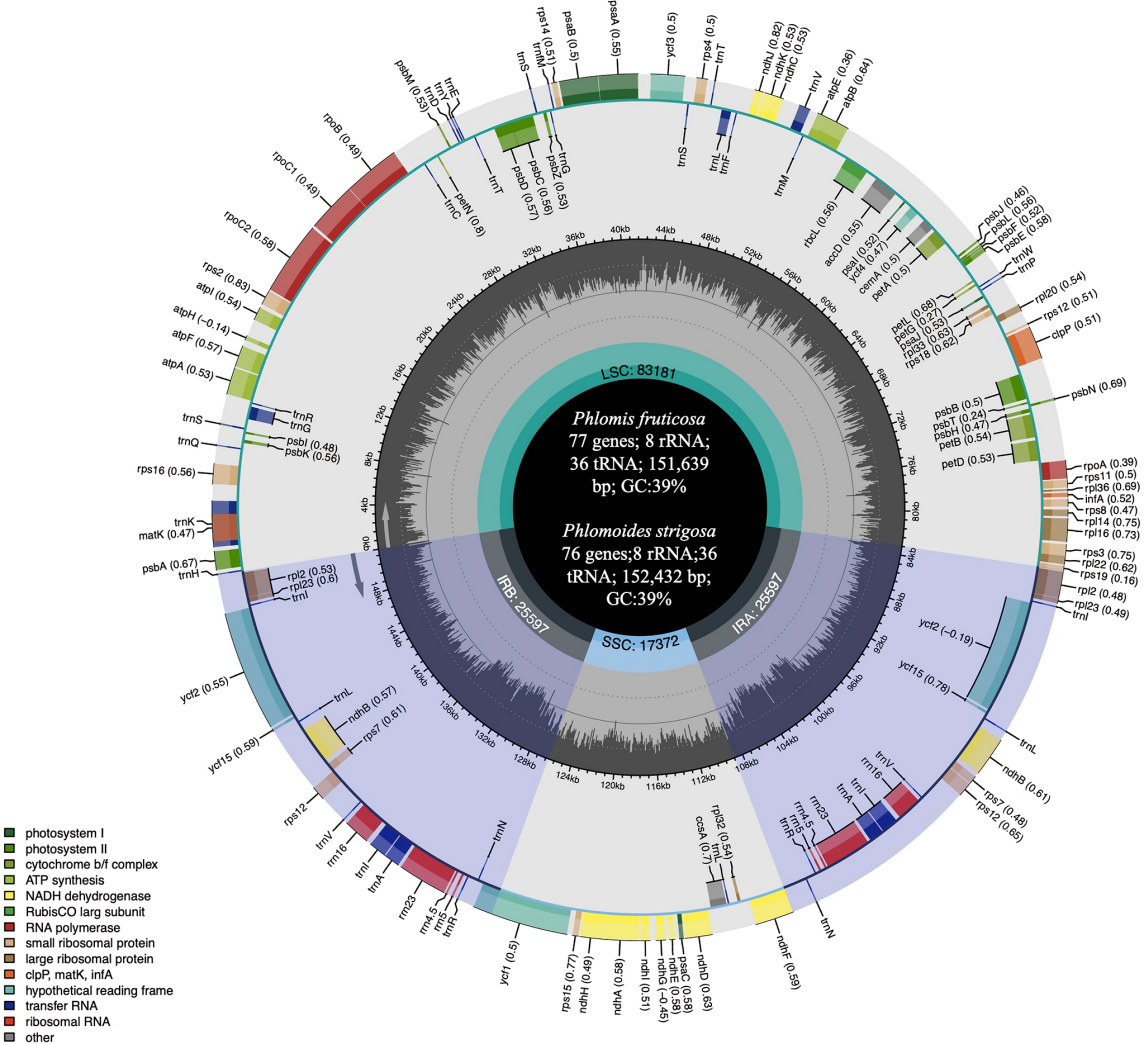
Gene map of the complete chloroplast of *Phlomis fruticosa* and *Phlomoides strigosa*. The species name and information regarding the genome are depicted in the center of the plot, including the genome length, GC contents, and annotated gene numbers. In the first inner circle, the GC content is depicted as the proportion of the shaded parts of each section. The length of the corresponding single short copy (SSC), inverted repeat (IRa and IRb), and large single-copy (LSC) regions is also given. The gradient GC content of the genome is plotted in the second circle with zero level based on the outer circle. The gene names are labeled on the outer circle, with their codon usage bias in brackets. Genes are color-coded by their functional classification. Represented with arrows, the transcription directions for the inner and outer genes are listed clockwise and anticlockwise, respectively.

Approximately 2.4 GB of paired-end raw sequence data for each species were obtained and submitted to NCBI (PRJNA864879 for *Phlomis fruticosa* and PRJNA864880 for *Phlomoides strigosa*). The complete chloroplast genome sequences of *Phlomis fruticosa* and *Phlomoides strigosa* were assembled and deposited to NCBI database with the GenBank accession number MZ670599 and ON811679, respectively. In addition, complete and annotated chloroplast genome sequence of *Phlomis fruticosa* and *Phlomoides strigosa* was uploaded as supplementary files. The assembled chloroplast genomes of *Phlomis fruticosa* and *Phlomoides strigosa* were 151,639 bp and 152,432 bp in length, respectively, with typical quadripartite structures and conserved constitute regions, including a small single-copy (SSC) region, a large single-copy (LSC) region and two inverted repeated (IR) regions (**Figure 1**; **Table 1**). The length of LSC, SSC, and IR in *Phlomis fruticosa* was 82,969 bp, 17,368 bp, and 25,651 bp, respectively, and in *Phlomoides strigosa* was 83,639 bp, 17,517 bp, 25,638 bp, respectively (**Table 1**). The GC contents were high similar between *Phlomis fruticosa* and *Phlomoides strigosa*. Each region of chloroplast showed different GC contents, with LSC, SSC, and IR ranging from 36.7–36.8%, 32.6%, 43.4%, respectively (**Table 1**).

**Table 1:** Summary of chloroplast genomes of *Phlomis fruticosa* and *Phlomoides strigosa*.

Region	Features	*Phlomis fruticosa*	*Phlomoides strigosa*
Total	Length (bp)	151,639	152,432
	GC content (%)	38.6	38.5
LSC	Length (bp/%)	82,969/54.7	83,639/54.9
	GC content (%)	36.8	36.7
SSC	Length (bp/%)	17,368/11.5	17,517/11.5
	GC content (%)	32.6	32.6
IR	Length (bp/%)	25.651/16.9	25,638/16.8
	GC content (%)	43.4	43.4

The chloroplast genome of *Phlomis fruticosa* was predicted to encode 121 genes, including 77 protein-coding genes, 8 rRNA genes, and 36 tRNA genes; while that of *Phlomoides strigosa* was predicted to encode 120 genes, including 76 protein-coding genes, 8 rRNA genes, and 36 tRNA genes. The difference in annotated gene numbers between *Phlomis fruticosa* and *Phlomoides strigosa* was attributed to the absence of *ndhF* in *Phlomoides strigosa* (**Table 2**). In addition, the IRs regions of *Phlomis fruticosa* contained 12 protein-coding genes, 12 tRNA genes, and 8 rRNA genes, and the LSC and SSC regions contained 53 and 12 protein-coding genes, and 24 and 1 tRNA genes, respectively. In *Phlomoides strigosa*, the IRs regions contained 14 protein-coding genes, 14 tRNA genes, and 8 rRNA genes, and the LSC and SSC regions contained 50 and 12 protein-coding genes, and 21 and 1 tRNA genes, respectively (**Figure 1**). In *Phlomis fruticosa*, 12 protein-coding and 8 tRNA genes were intron-containing genes. *clpP* contained two introns, while other genes contained one intron. In *Phlomoides strigosa*, 12 protein-coding and 8 tRNA genes were intron-containing genes. *pafI* and *clpP* contained two introns, while other genes contained one intron. The length of intron in *trnK-UUU* gene was 2,511 and 2,519 bp in *Phlomis fruticosa* and *Phlomoides strigosa*, respectively, which was the longest among all genes (**Table 3**).

**Table 2:** List of genes annotated in the *Phlomis fruticosa* and *Phlomoides strigosa* chloroplast genomes.

Category	Group of genes	Name of genes
Self-replication	Large subunit of ribosomal proteins	*rpl14*, *rpl16*, *rpl2* ^*2,2^, *rpl20*, *rpl22*, *rpl23* ^*2,2^, *rpl33*, *rpl36*
	Small subunit of ribosomal proteins	*rps2*, *rps3*, *rps4*, *rps7* ^*2,2^, *rps8*, *rps11*, *rps12*, *rps14*, *rps16*, *rps18*, *rps19* ^*2,2^
	DNA-dependent RNA polymerase	*rpoA*, *rpoB*, *rpoC1* ^*^, *rpoC2*
	rRNA genes	*rrn16S* ^*2,2^, *rrn23S* ^*2,2^, *rrn4.5S* ^*2,2^, *rrn5S* ^*2,2^
	tRNA genes	*trnA-UGC* ^*2,2^, *trnC-GCA*, *trnD-GUC*, *trnE-UUC*, *trnG-GCC*, *trnG-UCC*, *trnH-GUG*, *trnI-CAU* ^*2,2^, *trnI-GAU* ^*2,2^, *trnK-UUU*, *trnL-CAA* ^*2,2^, *trnL-UAA*, *trnM-CAU* ^2,2^, *trnN-GUU* ^*2,2^, *trnP-GAA*, *trnP-UGG*, *trnQ-UUG*, *trnR-ACG* ^*2,2^, *trnR-UCU*, *trnS-GCU*, *trnS-GGA*, *trnS-UGA*, *trnT-GGU*, *trnT-UGU*, *trnV-GAC* ^*2,2^, *trnV-UAC*, *trnW-CCA*, *trnY-GUA*
Photosynthesis	Photosystem I	*psaA*, *psaB*, *psaI*, *psaJ*
	Photosystem II	*psbA*, *psbB*, *psbC*, *psbD*, *psbE*, *psbF*, *psbH*, *psbI*, *psbJ*, *psbK*, *psbL*, *psbM*, *psbT*, *psbZ*
	NADH oxidoreductase	*ndhB* ^*2,2^, *ndhC*, *ndhF* ^1,0^, *ndhJ*, *ndhK*
	Cytochrome b6/f complex	*petA*, *petB*, *petD*, *petG*, *petL*, *petN*
	ATP synthase	*atpA*, *atpB*, *atpE*, *atpF*, *atpH*, *atpI*
	Rubisco	*rbcL*
Other genes	Maturase	*matK*
	Protease	*clp*
	Envelope membrane protein	*cemA*
	Subunit acetyl-CoA-carboxylase	*accD*
	c-Type cytochrome synthesis gene	*ccsA*
	Conserved open reading frames	*ycf1* ^*2,2^, *ycf2* ^*2,2^
	Translational initiation factor	*infA*

Asterisks indicate IR-located genes. Numbers marked by superscript indicate duplicated genes in the chloroplast genomes of Phlomis fruticosa (left) or Phlomoides strigosa (right).

**Table 3:** Intron-containing genes and the length of exons and introns.

Species	Region	Gene	Length (bp)				
			Exon I	Intron I	Exon II	Intron II	Exon III
*Phlomis fruticosa*	LSC	*atpF*	145	654	410		
	LSC	*clpP*	71	658	294	621	226
	LSC	*petB*	6	728	642		
	LSC	*petD*	8	737	475		
	LSC	*rpl16*	9	902	393		
	LSC	*rpoC1*	430	782	1625		
	LSC	*rps16*	40	868	227		
	LSC	*trnG-UCC*	23	681	48		
	LSC	*trnK-UUU*	37	2511	35		
	LSC	*trnL-UAA*	35	488	50		
	LSC	*trnV-UAC*	38	574	35		
	SSC	*ndhA*	553	1068	539		
	IR	*ndhB* ^*^	775	674	758		
	IR	*rpl2* ^*^	391	667	434		
	IR	*trnA-UGC* ^*^	38	806	35		
	IR	*trnI-GAU* ^*^	37	937	35		
*Phlomoides strigosa*	LSC	*clpP*	71	659	292	619	228
	LSC	*pafI*	124	709	230	726	153
	LSC	*petB*	6	736	642		
	LSC	*petD*	8	731	475		
	LSC	*rpl16*	9	915	399		
	LSC	*rpoC1*	432	795	1611		
	LSC	*rps16*	36	873	231		
	LSC	*trnG-UCC*	23	688	47		
	LSC	*trnK-UUU*	37	2519	35		
	LSC	*trnL-UAA*	35	482	50		
	LSC	*trnV-UAC*	36	580	37		
	SSC	*ndhA*	553	1074	539		
	IR	*ndhB* ^*^	777	679	756		
	IR	*rpl2* ^*^	391	667	434		
	IR	*trnA-UGC* ^*^	38	806	35		
	IR	*trnI-GAU* ^*^	37	937	35		

Asterisks indicate duplicated genes.

### Long repeat structure and simple sequence repeat analysis

**Figure 2:**
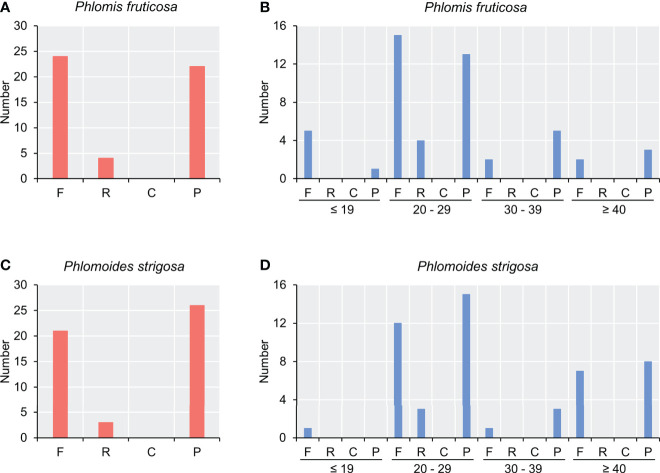
Analysis of long repeat sequence in chloroplast genomes of *Phlomis fruticosa* and *Phlomoides strigosa*. Four types of long repeat were analyzed in **(A, B)** *Phlomis fruticosa* and **(C, D)** *Phlomoides strigosa*. The x-axis represents the long repeat types, and the y-axis represents the numbers of corresponding long repeat type. F, forward repeat; R, reverse repeat; C, complement repeat; P, palindromic repeat.

In total, 50 long repeats were detected in two species. There were 24 forward repeats, 4 reverse repeats, and 22 palindromic repeats in the chloroplast genome of *Phlomis fruticosa* (**Figures 2A, B**), while there were 21 forward repeats, 3 reverse repeats, and 26 palindromic repeats in that of *Phlomoides strigosa* (**Figure 2C, D**). No complement repeat was detected in two species. The size of long repeats was 19–64 bp in *Phlomis fruticosa* and 18–46 bp in *Phlomoides strigosa*. The repeats ranging from 20 to 29 bp in length were predominant in both species (**Figures 2B, D**).

**Figure 3:**
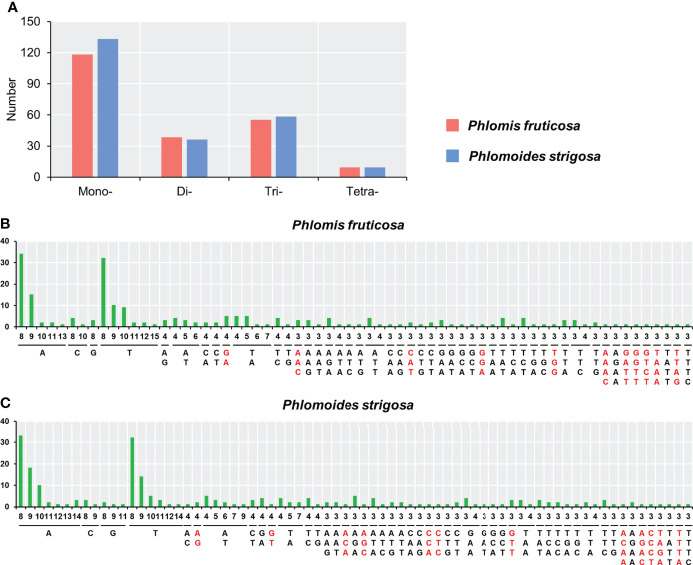
Analysis of simple sequence repeats in chloroplast genomes of *Phlomis fruticosa* and *Phlomoides strigosa*. Simple sequence repeats (SSRs) were analyzed in *Phlomis fruticosa* and *Phlomoides strigosa*. **(A)** Statistics of four types of SSRs in *P. fruticosa* and *P. strigosa*. **(B, C)** The x-axis represents the nucleotide units of each SSR, and the numbers of units within each SSR. The y-axis represents the numbers of each SSR in the chloroplast genome. SSRs unique to *P. fruticosa* or *P. strigosa* are marked in red. Mono-, mono-nucleotide repeat; Di-, di-nucleotide repeat; Tri-, tri-nucleotide repeat; Tetra-, tetra-nucleotide repeat.

Simple sequence repeats (SSRs), also known as a microsatellite, are widely distributed the genome of plants. SSRs generally consist of 1–6 nucleotides, mainly in units of repeats of 2–3 nucleotides, such as (GA)_n_, (AC)_n_, and (GAA)_n_. SSR analysis showed that the numbers and distributions of the SSRs were very similar in the two chloroplast genomes. A total of 220 and 236 SSRs were identified in *Phlomis fruticosa* and *Phlomoides strigosa* chloroplast genome, respectively. The mononucleotide, dinucleotide, trinucleotide, and tetranucleotide SSR were 118, 38, 55, 9 in the chloroplast genome of *Phlomis fruticosa*, respectively, and 133, 36, 58, 9 in the chloroplast genome of *Phlomoides strigosa*, respectively (**Figure 3A**). No pentanucleotide or hexanucleotide SSR was found in either *Phlomis fruticosa* or *Phlomoides strigosa*. The predominant SSR types were A/T, AT/TA, AAG/CTT, AAT/ATT and AG/CT. 61 different SSR types were found, 11 types were unique to *Phlomis fruticosa* had 11 unique types, while 12 types were unique to *Phlomoides strigosa* (**Figures 3B, C**). These long repeats and SSRs may provide materials for the molecular identification of *Phlomis* and *Phlomoides* species.

### Comparative chloroplast genomic analysis

**Figure 4:**
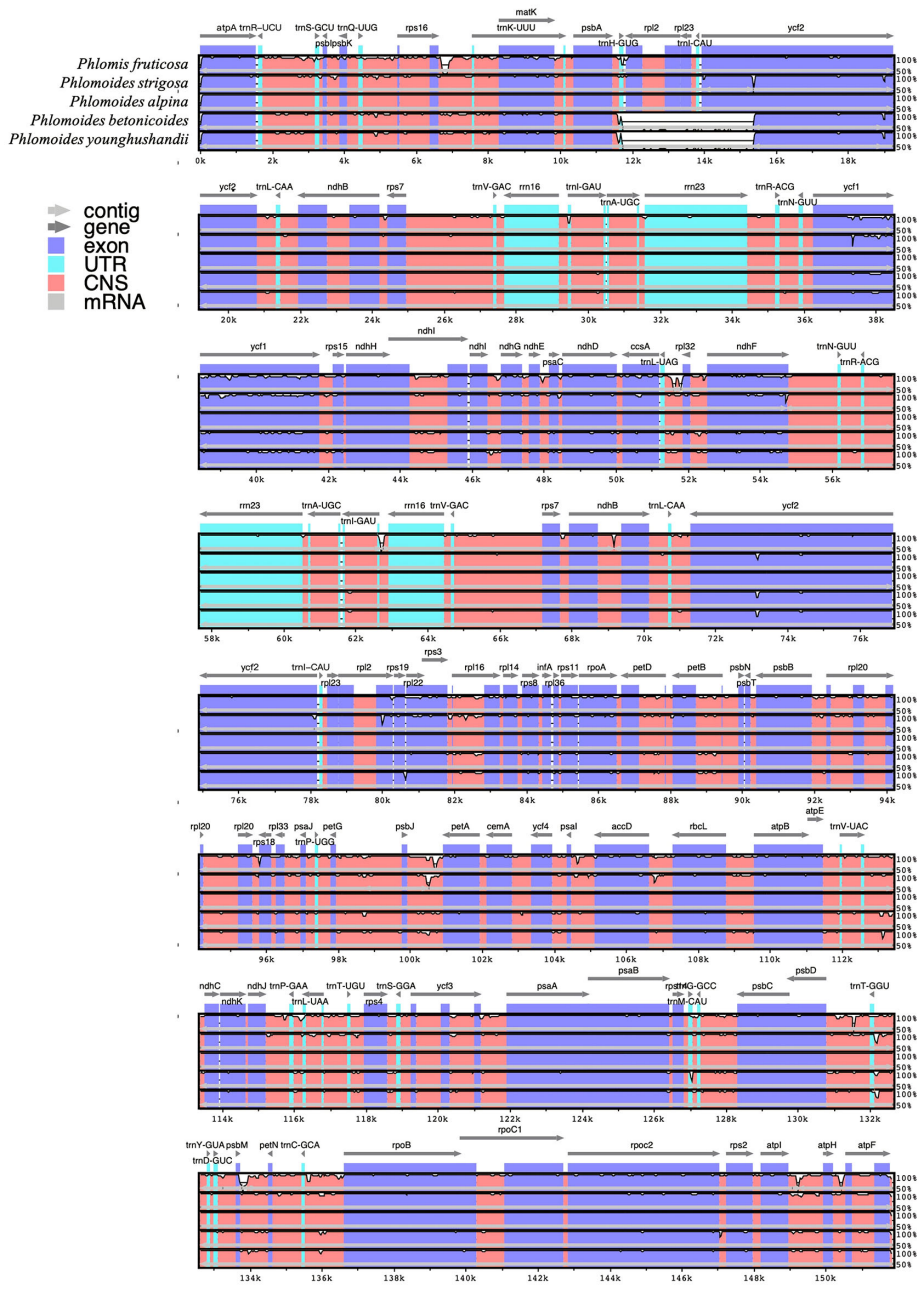
Comparison of the chloroplast genomes of *Phlomis fruticosa* and four *Phlomoides* species. The mVISTA plot is based on moving a specified window over the entire alignment and calculating the percent identity over the window at each base pair. The x-axis represents the base sequence, and the y-axis represents the percent identity. Arrows indicate the direction of genes, while the coding exons and UTRs are marked with rectangles of different color. Conserved regions (defined below) are highlighted under the curve, with red indicating a conserved non-coding region and blue indicating a conserved exon. Conserved UTRs are colored turquoise. A conserved regions are defined with percentage and length cutoffs. Conserved segments with percent identity x and length y are defined to be regions in which each contiguous subsegment of length y is at least x% identical to its paired sequence. These segments are merged to define the conserved regions.

**Figure 5:**
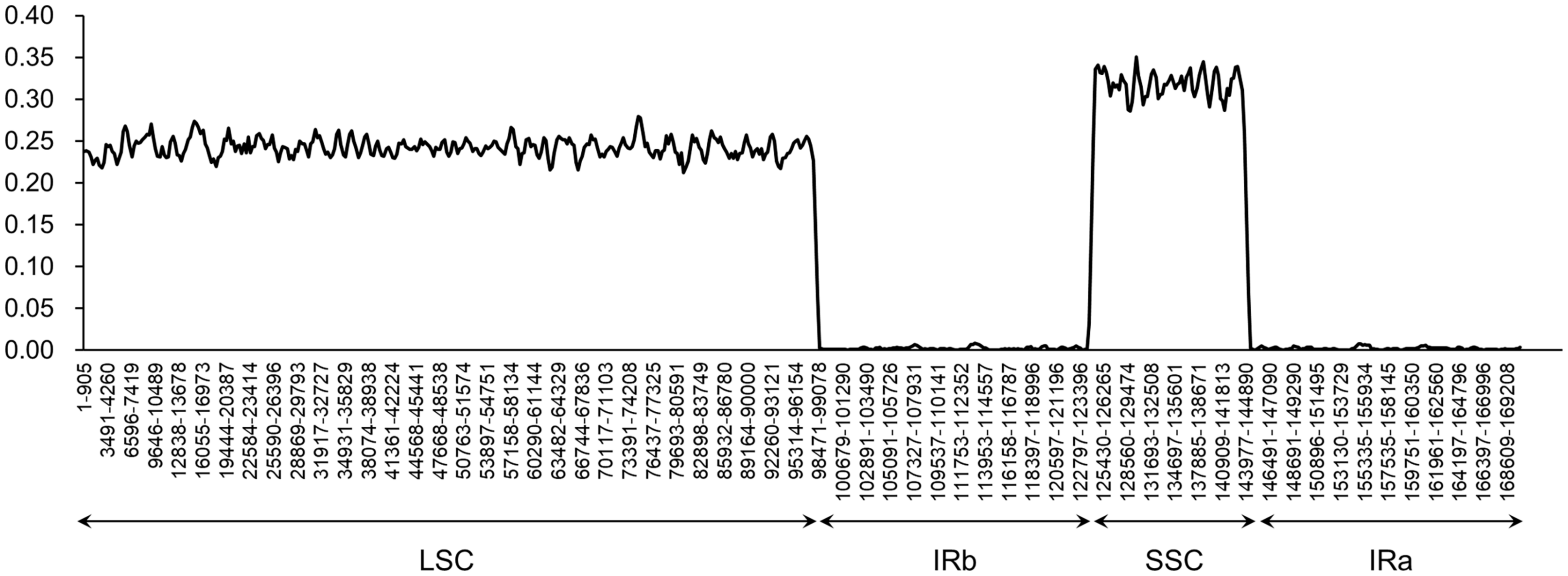
The nucleotide diversity of the plastid genome of *Phlomis fruticosa* and four *Phlomoides* species. .

A comparative analysis of chloroplast genomes was conducted among *Phlomis fruticosa*, *Phlomoides strigosa*, and three published *Phlomoides* species (*Phlomoides alpina*, *Phlomoides betonicoides*, and *Phlomoides younghushandii*) to investigate the difference of chloroplast genome structures among these species. As expected, more variable regions were found in the non-coding sequence (CNS) than the coding sequence (**Figure 4**), since the coding sequence is generally more conserved than CNS. Although the gene-coding region was overall highly conserved, the conservation of the *rpl2*, *rpl23* and *ycf2* genes was poor, resulting a large gap around this region in *Phlomoides betonicoides* and *Phlomoides younghushandii*. The nucleotide diversity of the chloroplast genomes of *Phlomis fruticosa* and four *Phlomoides* species were calculated to evaluate their sequence divergence levels. For all five species in this study, Pi values in the LSC region ranged from 0.21278 to 0.27917, with a mean of 0.24251, and from 0.06806 to 0.35111 in the SSC region, with an average of 0.31170, while in the IR regions, Pi values changed from 0.0083 to 0.0075 with an average value of 0.00199 (**Figure 5**). As expected, this result showed that the SSC regions of five chloroplast genomes were the most divergent, followed by LSC regions, and the IR regions were most conserved.

### IR contraction and expansion in the chloroplast genomes

**Figure 6:**
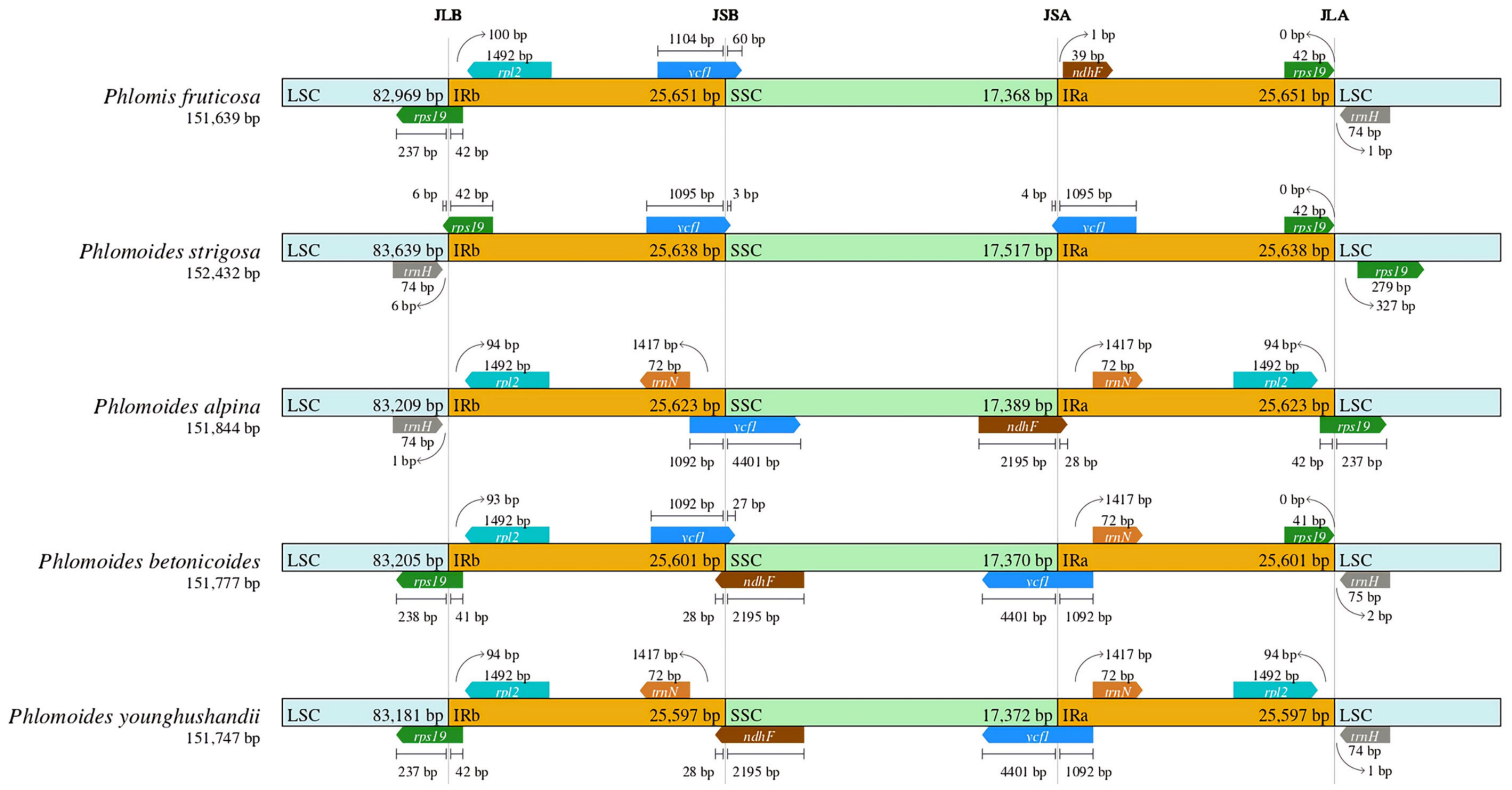
Comparison of border distance between adjacent genes and junctions of the LSC, SSC and two IR regions among the chloroplast genomes of *Phlomis fruticosa* and four *Phlomoides* species. The species names and the length of chloroplast genomes are depicted on the left of each track. Boxes above or below the main line indicate the adjacent border genes. Genes transcribed forward and reverse strands are presented above and below the tracks, respectively. The arrows indicate the bp distance of the start or end coordinate of genes from the corresponding junction site. For the genes extending from a region to another, the T bar on the top or below shows the extent of their parts with their corresponding values. This figure does not reflect the scale with respect to sequence length, but only shows relative changes at or near the IR/SC borders. LSC, large single-copy region; SSC, small single-copy region; IR, inverted repeated region; JLA, junction of LSC and IRa; JLB, junction of LSC and IRb; JSA, junction of SSC and IRa; JSB, junction of SSC and IRb. .

A comparative analysis of the IR-LSC and IR-SSC border structure among *Phlomis* and *Phlomoides* species was performed. The result showed that LSC/IRb junctions were located 42 bp, 42 bp, 41 bp, 42 bp inside *rps19* gene of *Phlomis fruticosa*, *Phlomoides strigosa*, *Phlomoides betonicoides* and *Phlomoides younghushandii*, respectively, and located 94 bp away from *rpl2* gene of *Phlomoide alpina.* The SSC/IRb junctions were located 1092 bp, 1104 bp, 1095 bp, 1092 bp inside *ycf1* gene of *Phlomoides alpina*, *Phlomis fruticosa, Phlomoides strigosa and Phlomoides betonicoides*, respectively, and located within *ndhF* gene with 2195 bp of *Phlomoides younghushandii*. The SSC/IRa junctions were located 1092 bp, 1095 bp, 1092 bp, 1092 bp inside *ycf1* gene of *Phlomoides alpina*, *Phlomoides strigosa, Phlomoides betonicoides* and *Phlomoides younghushandii*, respectively, and located 1 bp away from *ndhF* gene of *Phlomis fruticosa.* The LSC/IRa junctions were located 42 bp, 42 bp, 42 bp, 41 bp inside *rps19* gene of *Phlomoides alpina*, *Phlomis fruticosa*, *Phlomoides strigosa* and *Phlomoides betonicoides*, respectively, and located 94 bp away from *rpl2* gene of *Phlomoides younghushandii* (**Figure 6**).

### Codon usage bias analysis

**Figure 7:**
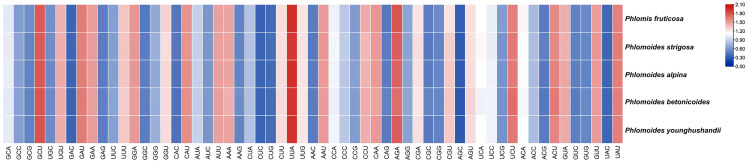
The RSCU values of shared protein-coding genes for *Phlomis fruticosa* and four *Phlomoides* species. In the colored boxes, deeper colors in red and blue indicate higher and lower RSCU values, respectively.

**Figure 8:**
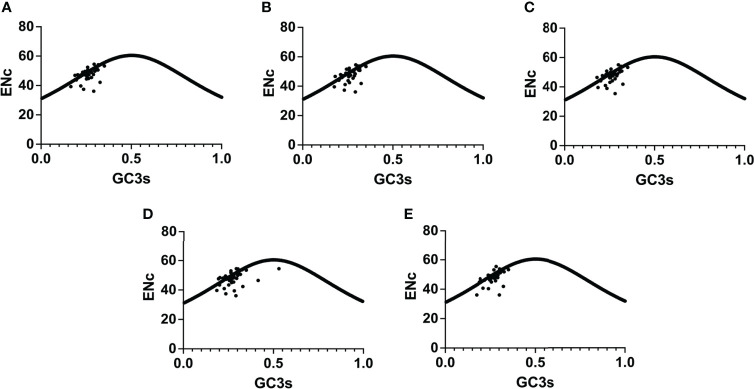
ENc-plot analysis for Phlomis fruticosa and four Phlomoides species. From **(A–E)** are the ENc-plot analysis of Phlomoides alpina, Phlomoides betonicoides, Phlomoides younghushandii, Phlomoides strigosa, and Phlomis fruticosa. If the point in the figure falls near the curve, it indicates that the actual value of ENc is close to the expected value. On the surface, its codon bias is mainly affected by base mutation. If the point falls away from the curve, it indicates that base mutation is not the main factor affecting its codon preference.

The chloroplast genomes of *Phlomis fruticosa* and four *Phlomoides* species were subjected to a codon usage bias analysis by calculating the RSCU values. The codon usage biases with high similarity among five species were observed (**Figure 7**; **Table S2**). The RSCU values of 32 codons were bigger than 1, indicating that these codons showed biased usage. The codons encoding Leu were the most abundant (1527-1704), while those encoding Cys were the least (139-156) (**Table S3**). To further investigate the factors affecting the codon usage bias, the ENc and GC3s values were calculated (**Table S4**). The result showed that the most of plots were distributed along or near the standard curve, whereas less plots deviated from the standard curve, indicating that base mutation was the main factor affecting codon bias (**Figure 8**).

### Phylogenetic analysis based on chloroplast genome sequences

**Figure 9:**
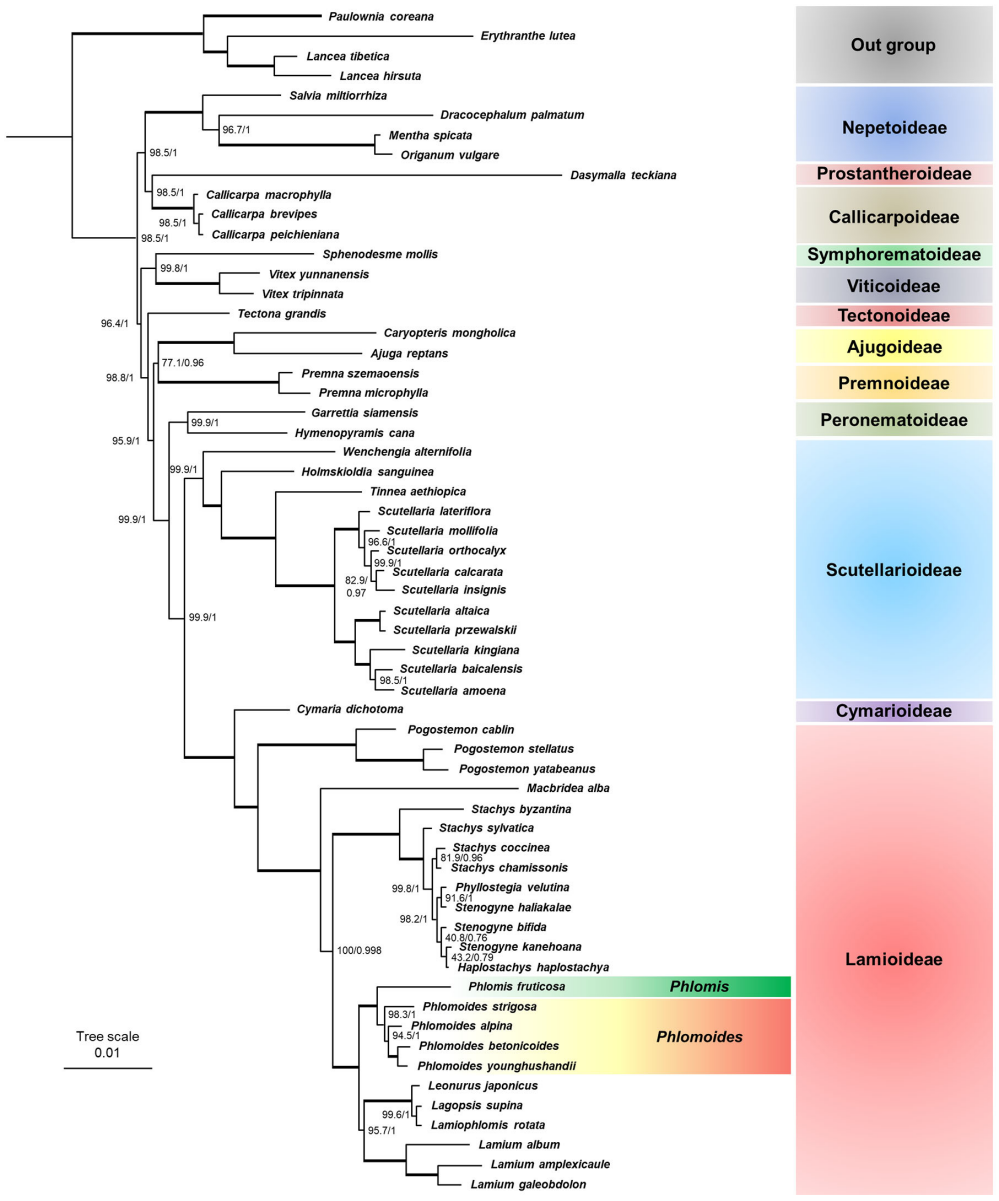
Phylogenetic tree based on chloroplast genomes of 56 Lamiaceae species. The topology of phylogenetic tree and the associated edge length is depicted. Maximum likelihood bootstrap support (MLBS) and Bayesian inference posterior probability (BIPP) are shown forward and backward on the right of bifurcation, respectively. Bold horizontal lines indicate clades with BIPP = 1.00 and MLBS = 100%.

In recent years, chloroplast genomes are widely used for phylogenetic analysis because the information in these sequences are robust and authentic. To investigate the taxonomic position of *Phlomis fruticosa* and *Phlomoides strigosa*, and confirm the relation between *Phlomis* and *Phlomoides* genera, 54 published chloroplast genomes from the Lamiaceae family were applied for phylogenetic analyses. These assembled chloroplast genomes covered 12 subfamilies of Lamiaceae, which enabled the investigation of taxonomic position of *Phlomis fruticosa* and *Phlomoides strigosa*. Within the Lamioideae subfamily, several species from *Leonurus*, *Lagopsis*, *Stachys*, *Haplostachys*, and *Stenogyne* genera were selected since their close relationship with *Phlomis* and *Phlomoides* (Pan et al., 2009; Scheen et al., 2010; Bendiksby et al., 2011; Mathiesen et al., 2011). Both analyses using ML and BI methods yielded the identity topologies with strong support at most nodes (**Figure 9**). Within the Lamiaceae family, all 12 subfamilies were recovered with strong support. Two primary clades were identified. Clade I comprised the Nepetoideae, Prostantheroideae, and Callicarpoideae. Clade II comprised Symphorematoideae, Viticoideae, Tectonoideae, Ajugoideae, Premnoideae, Peronematoideae, Scutellarioideae, Cymarioideae, and Lamioideae. All subfamilies that contained more than one species formed as monophyletic. Within Lamioideae (100%, 1.00), Pogostemoneae was the earliest branch (100%, 1.00), and sister to other species in Lamioideae. *Phlomis* and *Phlomoides* were recovered as sister to the clade of Leonureae + Lamieae with strong support (100%, 1.00), and split into two clades. *Phlomoides* was well-supported as a monophyletic group (98.3%, 1.00), in which *Phlomoides strigosa* was the closest relative to *Phlomis fruticosa*. These results confirmed that *Phlomis* and *Phlomoides* were separate genera.

## Discussion

The Lamiaceae family, commonly known as the mint family, contains approximately 236 genera and more than 7,000 species. Numbers of Lamiaceae plants are famous for their fragrance, flavor, spice, and medicinal properties. *Phlomis* s.l. is a large genus consisting of more than 100 species and widely distributed in Asia and Europe. However, the chloroplast genomic data of only several species has been reported, which hampered the understanding of their evolution and taxonomy.

In this study, we assembled the complete chloroplast genome of two *Phlomis* species: *Phlomis fruticosa*, which is the first reported chloroplast genome of *Phlomis*, and *Phlomoides strigosa*, which belongs to *Phlomoides*. The length of chloroplast genomes of *Phlomis fruticosa* and *Phlomoides strigosa* was 151,639 bp and 152,432 bp, respectively. The GC content was lower than AT content, and the GC content in the IR region was higher than other regions in both species, which may be caused by the presence of a large amount of rRNA in the IR region. The numbers of introns within genes are important information for plant chloroplast genomes. In our study, *ycf3*, *pafI*, and *clpP* included two introns, while the other intron-containing genes included one intron. All these features are consistent with the chloroplast genomes of most angiosperms.

The long repeat sequences and SSR sequences of chloroplast genome are important as molecular markers for the identification of plant germplasm. Our study provided detailed information about long repeat and SSR sequences, which may be suitable for the identification *Phlomis* and *Phlomoides* species, especially when morphological data of samples are not available. On the other hand, due to the extreme variability and wide distribution in the chloroplast genome, SSRs have been used in studies of genetic population structure and maternal analysis (Ebert and Peakall, 2009; Dong et al., 2016). Due to the difficult availability of complete chloroplast genomes at present, genetic studies in *Phlomis* and *Phlomoides* using SSR is a suitable method.

The comparative analysis of five *Phlomis* s.l. species (*Phlomis fruticosa*, *Phlomoides strigosa*, *Phlomoides betonicoides*, *Phlomoides younghushandii*, and *Phlomoides alpina*) revealed that these chloroplast genomes ranged from 151 kb to 152 kb in size, and comprised all the four major components of chloroplast genome architecture. Like typical angiosperms cp genomes, the IR regions of these species ranged from 25,597 to 25,651 bp in length. The highest variation in nucleotide was observed in the SSC regions, while the IR regions were the most conserved, which is consistent with most other plant species. The codon usage bias analysis revealed that the codon bias was mainly caused by base mutation.

Although *Phlomis* and *Phlomoides* were previously treated as two sections within the *Phlomis* genus by some taxonomists (Bentham, 1832-1836; Boissier, 1879; Briquet, 1895-1897; Rechinger, 1982), there is obvious variation between *Phlomis* and *Phlomoides*, including the growth form, leaf shape, leaf venation, upper corolla lip, verticillasters, and distribution (Xiang et al., 2014). The chromosome number in *Phlomis* and *Phlomoides* (Azizian and Cutler, 1982) is also a potent difference. Several molecular phylogenetic studies supported a split of *Phlomis* and *Phlomoides* into two separate groups (Bendiksby et al., 2011; Mathiesen et al., 2011; Salmaki et al, 2012; Zhao et al., 2021). Therefore, it is general accepted that *Phlomis* and *Phlomoides* are independent genera at present. Our molecular phylogenetic analysis using the chloroplast genomes showed that *Phlomoides* was a monophyletic group, and *Phlomoides strigosa* and *Phlomis fruticosa* were closely related. Our result supported the treatment of independent genera of *Phlomis* and *Phlomoides*. More public data of *Phlomis* chloroplast genomes will further confirm this conclusion.

## Data availability statement

The original contributions presented in the study are publicly available. All annotated chloroplast genomes have been deposited in GenBank as MZ670599 (*Phlomis fruticosa*) and ON811679 (*Phlomoides strigosa*), and available in the Supplementary Material. Raw data has been deposited in NCBI (https://www.ncbi.nlm.nih.gov/) as PRJNA864879 (*Phlomis fruticosa*) and PRJNA864880 (*Phlomoides strigosa*). Further inquiries can be directed to the corresponding authors.

## Author contributions

YL, ZY, and HC conceived and designed the experiments. WZ, LG, YY, YW, and LY performed the experiments. WZ, CW, JG, and KY analyzed the data. WZ and YL prepared the manuscript. ZY and KY provided advice on the experiments and revised the manuscript. YL, HC, and ZY supervised the project. All authors contributed to the article and approved the submitted version.

## Funding

This work was supported by the Qinghai Province International Cooperation Project (2021-HZ-805) to YL and HC, the Fundamental Research Funds for the Central Universities (lzujbky-2020-46) to YL, Qinghai Province Applied Basic Project (2020-ZJ-743) to HC, the Key Project of NMPA Key Laboratory for Quality Control of Traditional Chinese Medicine (2020GSMPA-KL11) to ZY, the Lanzhou Talent Innovation and Entrepreneurship Project (2017-RC-39) to KY.

## Acknowledgments

We thank Prof. Yinsuo Zhou from School of Pharmacy, Lanzhou University for providing the characterization of plant samples.

## Conflict of interest

The authors declare that the research was conducted in the absence of any commercial or financial relationships that could be construed as a potential conflict of interest.

## Publisher’s note

All claims expressed in this article are solely those of the authors and do not necessarily represent those of their affiliated organizations, or those of the publisher, the editors and the reviewers. Any product that may be evaluated in this article, or claim that may be made by its manufacturer, is not guaranteed or endorsed by the publisher.

## Abbreviations

s.l., sensu lato; s.str., sensu stricto; sect., section; ITS, internal transcribed spacer; CNS, conserved non-coding sequences.
