# Supplementary material for: Complete chloroplast genome sequences of Phlomis fruticosa and Phlomoides strigosa and comparative analysis of the genus Phlomis sensu lato (Lamiaceae)
Source: Front Plant Sci. 2022 Oct 28;13:1022273. doi: 10.3389/fpls.2022.1022273 (PMC9650320; doi:10.3389/fpls.2022.1022273)
Supplement: Supplementary file 3 [file DataSheet_3.docx]

LOCUS Phlomis fruticosa 151639 bp DNA circular 10-JUL-2021

DEFINITION Phlomis fruticosa chromoplast.

ACCESSION Phlomis.fruticosa

VERSION Phlomis.fruticosa

KEYWORDS .

SOURCE chromoplast Phlomis fruticosa

ORGANISM Phlomis fruticosa

Unclassified.

REFERENCE 1 (bases 1 to 151639)

AUTHORS Author,U.

TITLE Your Publication

JOURNAL Unpublished

REFERENCE 2 (bases 1 to 151639)

AUTHORS Author,U.

TITLE Direct Submission

JOURNAL Submitted (10-JUL-2021) Your Department, Your Institute, Your

Address 1, City, State 12345, Country

FEATURES Location/Qualifiers

source 1..151639

/organism="Phlomis fruticosa"

/organelle="plastid:chromoplast"

/mol_type="genomic DNA"

gene complement(join(121792..122585,94488..94601))

/gene="rps12"

/trans_splicing

CDS complement(join(121792..121817,122354..122585,

94488..94601))

/gene="rps12"

/trans_splicing

/codon_start=1

/transl_table=11

/product="ribosomal protein S12"

/translation="MPTIKQLIRNTRQPIRNVTKSPALGGCPQRRGTCTRVYTITPKK

PNSALRKVARVRLTSGFEITAYIPGIGHNSQEHSSVLVRGGRVKDLPGVRYHIVRGTL

DAVGVKDRQQGRSKYGVKKPK"

gene complement(join(147174..151639,1..1054))

/gene="ycf1"

CDS complement(join(147174..151639,1..1054))

/gene="ycf1"

/codon_start=1

/transl_table=11

/product="Ycf1 protein"

/translation="MIFQSFLLGNLVSLCMKIINSVVVVGLYYGFLTTFSIGPSYLFL

LRAQVMEEGTEKKVSATTGFITGQLMMFISIYYAPLHLALGRPHTITVLALPYLLFHF

FWNNHKHFFDYGSTTRNSMRNFSIQCLFLNNLIFQLFNHFILPSSMLARLVNIYMFRC

NNKMLFVTSSFVGWLIGHILFMKWLGLVLVWIRQNHSIRSNVLIRSNKYLVSELINSK

ARILSILLFITCVYYLGRIPSPLFTKKLKETSKTGAGERVESAEERDVEIETASEMKG

TKQEQEGSTEEDPSPYFFSEERADPNKIDETEEIQVNGKEKEFHFRFTETDYQNRPVS

EESYLMNINENQDNSRLKIVDQKTENKELIKKIDDKPLVTILFDSKRWNRPFRYIKNK

RFDKAVRNEMSQYFFDICQSDGKERISFTYPPSLSIFFEMIKKRISRISPPTIEKFSF

NELYNPWVYTNKQKENSFNNEFLNRIKALDKENSYLNILETRTRLWNDHSTKEYLSKR

YDPFLNGSYRKTIYKSPSPSTRKKTLIENFLDQFGINRIHGILLPDTDYQEFDQKINR

FEKKSLSTEIVNFLTLISKFVKESGSTNLNPSSLYLFSEREIDSQKETKYFNYLLNLN

KIVTDVNGQKIDRKSIRINEITKNVPRRSYKLITDLEQQSRKYKEDIPIGHQIRSRRG

KRVVILTAAKGTSKTVHRRMSDTKTDVTLMRYSQQSDFRRGIIKGSMRAQRRKVVIFE

LFQANVKSPLFLERLHKSPPSYFNISGLIKLIFKNGLEKGEAFQIGEYTKEQTKRKEN

EENNKRKEKARIKVAEDWNRIPFAQGIRGCVLLTQSIFRKYILFPSLIIAKNIGRIFL

LQRPEWAEDFQEWNKEIYIKCTYNGIPLSQTEFPKNWLTEGIQIKIVFPFCLKPWHKA

KLRSSQKDLMKKTKEGGDSCFLTVWGMETEFPFCAPRKKPSFFKPILKEFAKKIGKFK

KKYFRVLTVFKIKIKLLRKVSKETKKRVIKSVFFRKRFRKRIIKELSKVNPILLFRLR

EVGVDKSSQIKEEKDSIINNQMIHESFSQMQIASPTWTNSSLTEKKMEDLADRTSTIR

NQIERITKEKKKVTPRINNLSPTSSNAKKLEKQQMFKMLKRRNARLICKLSPFVKFFI

EKIYTDIFLYIINIARINTKLFLKLTKKIIDKSIYNNERKQERINKKKKTTIPSNSSI

IIRKELENISNIKANSHIFSDLSYVPQPYVFYKLGKIQVNNLLRFVVQYQRIPFFLKA

KIKDSFETQGMLDSKSADNKLPSYEMNPWKSWLRGHYQYHLSQIGWSRLIPEKWRNTF

RQHRIAKKENFSKRHSYENNPLMSSKKQKQLEVYSLSNQKENFLKYYQSDLLSYKFIH

YEKKTECFFYGSPLQGNPNQEISYNAPKKNFFDMLRNIPIKNDLGKIHMEKPADRKYF

DWKIFQFDLIQKVDIEAWIIIDTNRNQNTQIRTKNSQIISKKDFFYLKIPEINLPNSH

KGFYDWMGMNEKMLKHPISNLELWFFPEFLLIYKTYKMKPWFIPSKLLLLNLNRSETK

KINEKGNFLIASNKKHRNQEEKEPTSGGKRRSVLSPPKDIEENYARSNMKKGKKKKQY

TKAERRLFMKRYLLFQLRWDETLNEKMINNINLYCLLRQLIPSAKLIISSIQKKQMNL

DIIRINDNLTLSEFMKKGVLILEPIRLSEQKDGQFVMYQTVGISLVHKNKHQKYQEQG

HASNNNFDLLVPENILSFRRRRKLRILICFNSKKRNYIDQNPVFWNVKNSSQVLHDNN

HLDREKNQLMKLKFFLWPNYRFEDLACMNRYWFDTNNGSRFGMLRIQMYPRLKIF"

gene 1379..1452

/gene="trnN-GUU"

tRNA 1379..1452

/gene="trnN-GUU"

/product="tRNA-Asn"

gene 1380..1451

/gene="trnN-GUU"

tRNA 1380..1451

/gene="trnN-GUU"

/product="tRNA-Asn"

gene complement(2023..2097)

/gene="trnR-ACG"

tRNA complement(2023..2097)

/gene="trnR-ACG"

/product="tRNA-Arg"

gene complement(2024..2097)

/gene="trnR-ACG"

tRNA complement(2024..2097)

/gene="trnR-ACG"

/product="tRNA-Arg"

gene complement(2332..2452)

/gene="rrn5"

rRNA complement(2332..2452)

/gene="rrn5"

/product="5S ribosomal RNA"

gene complement(2708..2810)

/gene="rrn4.5"

rRNA complement(2708..2810)

/gene="rrn4.5"

/product="4.5S ribosomal RNA"

gene complement(2909..5719)

/gene="rrn23"

rRNA complement(2909..5719)

/gene="rrn23"

/product="23S ribosomal RNA"

gene complement(2909..5161)

/gene="rrn23"

rRNA complement(2909..5161)

/gene="rrn23"

/product="23S ribosomal RNA"

gene complement(5408..5719)

/gene="rrn23-fragment"

rRNA complement(5408..5719)

/gene="rrn23-fragment"

gene complement(5916..6794)

/gene="trnA-UGC"

tRNA complement(join(5916..5951,6758..6794))

/gene="trnA-UGC"

/product="tRNA-Ala"

tRNA complement(join(5916..5950,6757..6794))

/gene="trnA-UGC"

/product="tRNA-Ala"

tRNA complement(join(5916..5952,6757..6794))

/gene="trnA-UGC"

/product="tRNA-Ala"

exon complement(5916..5952)

/gene="trnA-UGC"

/number=2

exon complement(5916..5951)

/gene="trnA-UGC"

/number=2

exon complement(5916..5950)

/gene="trnA-UGC"

/number=2

intron complement(5951..6756)

/gene="trnA-UGC"

/number=1

intron complement(5952..6757)

/gene="trnA-UGC"

/number=1

intron complement(5953..6756)

/gene="trnA-UGC"

/number=1

exon complement(6757..6794)

/gene="trnA-UGC"

/number=1

exon complement(6758..6794)

/gene="trnA-UGC"

/number=1

gene complement(6859..7867)

/gene="trnI-GAU | trnE-UUC"

tRNA complement(join(6859..6898,7836..7867))

/gene="trnE-UUC"

/product="tRNA-Glu"

tRNA complement(join(6859..6893,7831..7867))

/gene="trnI-GAU"

/product="tRNA-Ile"

tRNA complement(join(6859..6893,7826..7867))

/gene="trnI-GAU"

/product="tRNA-Ile"

exon complement(6859..6898)

/gene="trnE-UUC"

/number=2

exon complement(6859..6893)

/gene="trnI-GAU"

/number=2

intron complement(6894..7830)

/gene="trnI-GAU"

/number=1

intron complement(6894..7825)

/gene="trnI-GAU"

/number=1

intron complement(6899..7835)

/gene="trnE-UUC"

/number=1

exon complement(7826..7867)

/gene="trnI-GAU"

/number=1

exon complement(7831..7867)

/gene="trnI-GAU"

/number=1

exon complement(7836..7867)

/gene="trnE-UUC"

/number=1

gene complement(8187..9677)

/gene="rrn16"

rRNA complement(8187..9677)

/gene="rrn16"

/product="16S ribosomal RNA"

gene complement(9900..9971)

/gene="trnV-GAC"

tRNA complement(9900..9971)

/gene="trnV-GAC"

/product="tRNA-Val"

gene join(complement(94488..94601),11587..12380)

/gene="rps12"

/trans_splicing

CDS join(complement(94488..94601),11587..11818,12355..12380)

/gene="rps12"

/trans_splicing

/codon_start=1

/transl_table=11

/product="ribosomal protein S12"

/translation="MPTIKQLIRNTRQPIRNVTKSPALGGCPQRRGTCTRVYTITPKK

PNSALRKVARVRLTSGFEITAYIPGIGHNSQEHSSVLVRGGRVKDLPGVRYHIVRGTL

DAVGVKDRQQGRSKYGVKKPK"

exon 11587..11818

/gene="rps12"

/number=2

intron 11819..12354

/gene="rps12"

/number=1

intron 11820..12355

/gene="rps12"

/number=1

exon 12355..12380

/gene="rps12"

/number=3

gene 12434..12901

/gene="rps7"

CDS 12434..12901

/gene="rps7"

/codon_start=1

/transl_table=11

/product="ribosomal protein S7"

/translation="MSRRGTAEEKTAKSDPIYRNRLVNMLVNRILKHGKKSLAYQIIY

RAMKKIQQKTETNPLSVLRQAIRGVTPDIAVKARRVGGSTHQVPIEIGSTQGKALAIR

WLLAASRKRPGRNMAFKLSSELVDAAKGSGDAIRKKEETHKMAEANRAFAHFR"

gene 13165..15371

/gene="ndhB"

CDS join(13165..13941,14616..15371)

/gene="ndhB"

/codon_start=1

/transl_table=11

/product="NADH dehydrogenase subunit B"

/translation="MIWHVQNENFILDSTRIFMKAFHLLLFDGSLIFPECILIFGLIL

LLMIDSTSDQKDIPWLYFISSTSLVMSITALLFRWREEPIISFSGNFQTNNFNEIFQF

LILLCSTLCIPLSVEYIECTEMAITEFLLFVLTATLGGMFLCGANDLITIFVAPECFS

LCSYLLSGYTKKDVRSNEATMKYLLMGGASSSILVHGFSWLYGLSGGETELQEIVSGL

INTQMYNSPGISIALIFITVGIGFKLSPAPSHQWTPDVYEGSPTPVVAFLSVTSKVAA

SASATRILDIPFYFSSNEWHLLLEILAILSMILGNIIAITQTSMKRMLAYSSIGQIGY

VIIGIIVGDSNDGYASMITYMLFYISMNLGTFACIVLFGLRTGTDNIRDYAGLYTKDP

FLALSLALCLLSLGGLPPLAGFFGKLYLFWCGWQAGLYSLVLIGLLTSVVSIYYYLKI

IKLLMTGRNQEITPHVRNYRRSPLRSNNSIELSMIVCVIASTIPGISMNPIIAIAQDT

LF"

exon 13165..13941

/gene="ndhB"

/number=1

intron 13942..14615

/gene="ndhB"

/number=1

exon 14616..15371

/gene="ndhB"

/number=2

gene 15915..15997

/gene="trnL-CAA"

tRNA 15915..15997

/gene="trnL-CAA"

/product="tRNA-Leu"

gene 15916..15996

/gene="trnL-CAA"

tRNA 15916..15996

/gene="trnL-CAA"

/product="tRNA-Leu"

gene complement(16543..23382)

/gene="ycf2"

CDS complement(16543..23382)

/gene="ycf2"

/codon_start=1

/transl_table=11

/product="Ycf2 protein"

/translation="MKGHQFQSWIFELREILREIKNSHCFLDSWTQFNSVGSFIRIFF

HQERFLKLFDPRILSILLSRNSQGSTSNRYFTIKGVLLFVVAVLIYRINNRNMVERKN

LYLRGLLPIPMNSIGPRNDTLEESVGSSNINRLIVSLLYLPKGKKISESCFLNPKEST

WVLPITKKCSMPESNWGSRWWRNWIGKKRDSSCKISNETVTGIGILFKEKDLKYLEFL

FVYYMDDPIRKDHDWELFDRLSLRKRRNRINLNSGPLFEILVKHWISYLMSAFREKIP

IEVEGFFKQQRAGSTIQSNDIEHVSHLLSRNKRAISLQNCAQFHMWQFRQDLFVSWGK

NPHESDFLRNVSRENWIWLDNVWLVNKDRFFRKVRNVSSNIQYDSTRSSFVQVTDSSQ

LKGSSDQSRDHLDSISNEDSEYHTLINQREIQPLKERSILWDPSFLQTEGTEIESDRF

PKCLSGYSSMSRLFTEREKQMINHLLPEEIQEFLGNPTRSVRSFFSDRWSELHLGSNP

TERSTRDPKLLKKQQDLSFVPPRRSENKELVNIFKIITYLQNTVSIHPISSDPGCDRV

LKDDPDMDSSNKISFLNKNPFFDLFHLFHDRNRGGYTLHHDFESEERFQEMADLFTLS

ITEPDLVYHKGFPFSIDSYGLDQKQFLNEARDESKKKSLLVLPPIFYEENESFSRRIR

KKWVRISCGNDLEDPKPKIVVFASNNIMEAVNQYRLIRNLIQIQYSTYGYIRNVLNRL

FLMNRSDRNFEYGIQRDQIGKDTLNHRTIMKYTINPHLSNLKKSQKKWLDPLILISRT

ERSMNRAPDAYRYKWSNGSKNFQEHLEHFVSEQKSRFQIVFDRLRINQYSIDWSEVID

KKDLSKPLRFFLSKSLLFLSKLLFFLSNSLPFFCVSFGNIPIHRSEIYIYELKGPNDQ

LCNQLLESIGLQIVHLKKWKPFLLDEHDTSRKSKFLINGGTPFLFNKIPKWMIDSFHT

RNNRRKSFDNADSYFSMIFHNQDNWLNPVKPFHISSLISSFYKANRLRFLNNPHHFCF

YCNTRFPFSVEKARINNYDFTYGQFLNILFIRNKIFSLCVGKKKHAFGGRDTISPIES

QVSNIFIPNDFPQSGDETYNLYKSFHFPSQHDPFVRRTIYSIADMFGTPLTEGQIVHF

ERTYCQPLSDMNLSDSEGKNLHQYLNSNVGLIHTPCSGKYLPSEKRKRWSLCLKKCVE

KGQMYRTFQRDGAFSTLSKWNLFQTYIPWFLTSTGYKYLNLIFLDTFSDLLPILSSSQ

KFVSIFHDIMHGSGIAWRILQKKWCLPQWNLISEISSKCFHNLLLSEEMIHRNNESPS

THLRSPNVREFLYSILFLLLVAGYLVRTHLLFVSRASSELQTEFEKVKSLMIPSSMIE

LRKLLDRYPTSAPNSFWLKNLFLVALEQLGDSLEEIRASGGNMPGPAYGVKSIRSKKK

YLSINLIDLIPNPINQITFSRNTRHLSHTSKEIYSLIRKRKNVNGDWIDDKIESWVAN

SDSIDDEEREFLVQFSALTTEKRIDQILLSLTHSDHLSKNDSGYQMIEQPGAIYLRYL

VDIHKKYLLNYEFNTSSLAERRVFLAHYQTITYSQTSCGTNTLHFPSHGKPFSLRLAL

SPSRGILVIGSIGTGRSYLVKYLATNSYVPFITVFLNKFLDNKPKGFIFDDIDIDASD

DIDASDDIDASDDIDRDLDTELELLTMDRMPEIDRFYITLQFELAKAISPCIIWIPNI

HDLDVNESNYLSLGLLVNHLSERCSTRNILVIASTHIPQKVDPALIAPNKLNTCIKIR

RLLIPQQRKHFFTLSYTKGFHLEKKMFHTNGFGSITMGSNARDLVALTNEALSISITQ

KKSIIDTKTIRSALHRQTWDLRSQVRSVQDHGILFYQIGRAVAQNVLLSNCPIDPISI

YMKKKSCNEGDSYLYKWYFELGTSMKKLTILLYLLSCSAGSVAQDLWSLPGSDEKNGI

TSYGLVENDSDLVHGLLEVEGALVGSSRTEKDCSPFDNDRVTLLLRPELRNPLDMMQN

GSCSIFDQRFLYEKYESEFEEGKGEAALDPQQIEEDLFNHIVWAPRIWRPWAFLFDCI

ERPNELGFPYWSRSFRGKRIIYDEEDELQENDSEFLQSGTMQYQTRDRSSKEQGLFQI

SQFIWDPADPLFFLFKDQPPGSVFSHRELFADEEMSKGLLTSQTDPPTSIYKRWFIKN

TQEKHFELLINRQRWLRTNSSLSNGSFRSNTLSESYQYLSTLFLSNGTLLDQMTKTLV

RKRWLFPDEMKIGFMEQEKDFPFLSRKDMWL"

gene 23471..23545

/gene="trnM-CAU"

tRNA 23471..23545

/gene="trnM-CAU"

/product="tRNA-Met"

gene 23471..23544

/gene="trnI-CAU"

tRNA 23471..23544

/gene="trnI-CAU"

/product="tRNA-Ile"

gene 23710..23991

/gene="rpl23"

CDS 23710..23991

/gene="rpl23"

/codon_start=1

/transl_table=11

/product="ribosomal protein L23"

/translation="MDGIKHAVFTDKSIRLLGKNQYTSNVESGSTRTELKHWVELFFG

VRVIAMNSHRLPGKGRRMGPIMGHTMHYRRMIITLQPGYSIPPLRKKRT"

gene 24010..25501

/gene="rpl2"

CDS join(24010..24400,25068..25501)

/gene="rpl2"

/codon_start=1

/transl_table=11

/product="ribosomal protein L2"

/translation="MAIHLYKTSTPSTRNGTVDSQVKSNPRNNLIYGQHHCGKGRNAR

GIITSRHRGGGHKRLYRKIDFRRNEKDIYGRIVTIEYDPNRNAYICLIHYGDGEKRYI

LHPRGAIIGDTIVSGTEVPIKMGNALPLTDMPLGTAIHNIEITLGKGGQLVRAAGAVA

KLIAKEGKSATLKLPSGEVRLISKNCSATVGQVGNVGANQKSLGRAGSKRWLGKRPVV

RGVVMNPVDHPHGGGEGRAPIGRKKPTTPWGYPALGRRSRKRNKYSENLIVRRRSK"

exon 24010..24400

/gene="rpl2"

/number=1

intron 24401..25067

/gene="rpl2"

/number=1

exon 25068..25501

/gene="rpl2"

/number=2

repeat_region complement(25600..151590)

/note="inverted repeat A (IRA)"

/rpt_type=inverted

misc_feature 25601..108570

/note="large single copy (LSC)"

gene complement(25602..25676)

/gene="trnH-GUG"

tRNA complement(25602..25676)

/gene="trnH-GUG"

/product="tRNA-His"

gene complement(25603..25677)

/gene="trnH-GUG"

tRNA complement(25603..25677)

/gene="trnH-GUG"

/product="tRNA-His"

gene complement(25603..25676)

/gene="trnH-GUG"

tRNA complement(25603..25676)

/gene="trnH-GUG"

/product="tRNA-His"

gene complement(25907..26965)

/gene="psbA"

CDS complement(25907..26965)

/gene="psbA"

/codon_start=1

/transl_table=11

/product="photosystem II protein D1"

/translation="MTAILERRESESLWGRFCNWITSTENRLYIGWFGVLMIPTLLTA

TSVFIIAFIAAPPVDIDGIREPVSGSLLYGNNIISGAIIPTSAAIGLHFYPIWEAASV

DEWLYNGGPYELIVLHFLLGVACYMGREWELSFRLGMRPWIAVAYSAPVAAATAVFLI

YPIGQGSFSDGMPLGISGTFNFMIVFQAEHNILMHPFHMLGVAGVFGGSLFSAMHGSL

VTSSLIRETTENESANEGYRFGQEEETYNIVAAHGYFGRLIFQYASFNNSRSLHFFLA

AWPVVGIWFTALGISTMAFNLNGFNFNQSVVDSQGRVINTWADIINRANLGMEVMHER

NAHNFPLDLASIEAPTNG"

gene complement(27195..29780)

/gene="trnK-UUU"

tRNA complement(join(27195..27232,29744..29780))

/gene="trnK-UUU"

/product="tRNA-Lys"

exon complement(27195..27232)

/gene="trnK-UUU"

/number=2

gene complement(27198..29780)

/gene="trnK-UUU"

tRNA complement(join(27198..27232,29744..29780))

/gene="trnK-UUU"

/product="tRNA-Lys"

exon complement(27198..27232)

/gene="trnK-UUU"

/number=2

intron complement(27233..29743)

/gene="trnK-UUU"

/number=1

gene complement(27496..29031)

/gene="matK"

CDS complement(27496..29031)

/gene="matK"

/codon_start=1

/transl_table=11

/product="maturase K"

/translation="MEEIQRYLQLERSQQHDFLYPLIFQEYIYAFAHNRALSKLILSE

NLGYDNKSSLLIVKRLITRLYQQNHFLISPNDSNQNPFWARNNNLYSQIISEGFAFIV

EIPFSLQFLSCLEGETNKIGKSQNLRSIHSIFPFFEDTFSHFNFVLDMVIPRPVHVEI

LVQILRDCVKDASSLHLLRVFLNEYCNWNSLIIPKKASSPSSKKNKRLFLFLYNSHVC

EYESIFVFLRNQSFYLRSTSSGVFLERIYFYIKMERLVNVFVKIKDLGTNLRLVKEPF

MHYIRYQKRSILASKGTFIFMKKWKFYLVTFWQWHFSVWFHPRSIYINQLSKHSLEFL

GYLSSVRISPSVVRSQSIENSFLINNAINKFETLVPIIPLIASLAKAKFCNVLGHPVS

KPIRADLSDSNIIDRFGRICRNISRYHSGSSEKKSLYRIKYILRLSCARTLARKHKST

VRAFLKRLGSEFLEEFLMSEEHVLFLNFKKTSYTLRGVYRSRIWYLDMISINDLANQK

SKF"

gene complement(29744..29780)

/gene="trnK-UUU"

tRNA complement(29744..29780)

/gene="trnK-UUU"

/product="tRNA-Lys"

exon complement(29744..29780)

/gene="trnK-UUU"

/number=1

gene complement(30561..31680)

/gene="rps16"

CDS complement(join(30561..30772,31641..31680))

/gene="rps16"

/codon_start=1

/transl_table=11

/product="ribosomal protein S16"

/translation="MVKLRLKRCGRKQRAIYRIVAIDVRSRREGKDLRRVGFYDPMKN

QTFLNVPLILYFLERGAQPTGTVQNLLKKAEVFKELTPN"

exon complement(30561..30772)

/gene="rps16"

/number=2

intron complement(30773..31640)

/gene="rps16"

/number=1

exon complement(31641..31680)

/gene="rps16"

/number=1

gene complement(32672..32744)

/gene="trnQ-UUG"

tRNA complement(32672..32744)

/gene="trnQ-UUG"

/product="tRNA-Gln"

gene complement(32673..32744)

/gene="trnQ-UUG"

tRNA complement(32673..32744)

/gene="trnQ-UUG"

/product="tRNA-Gln"

gene 33081..33266

/gene="psbK"

CDS 33081..33266

/gene="psbK"

/codon_start=1

/transl_table=11

/product="photosystem II subunit K"

/translation="MLNIVSLICICINSPLFSSHFFFGKLPEAYAFLNPIVDFMPVIP

LFFFLLAFVWQAAVSFR"

gene 33649..33759

/gene="psbI"

CDS 33649..33759

/gene="psbI"

/codon_start=1

/transl_table=11

/product="photosystem II subunit I"

/translation="MLTLKLFVYTVVIFFVSLFIFGFLSNDPGRNPGREE"

gene complement(33876..33963)

/gene="trnS-GCU"

tRNA complement(33876..33963)

/gene="trnS-GCU"

/product="tRNA-Ser"

gene 34531..35284

/gene="trnS-CGA"

tRNA join(34531..34562,35225..35284)

/gene="trnS-CGA"

exon 34531..34562

/gene="trnS-CGA"

/number=1

gene 34532..35283

/gene="trnG-UCC"

tRNA join(34532..34554,35236..35283)

/gene="trnG-UCC"

/product="tRNA-Gly"

tRNA join(34532..34552,35253..35283)

/gene="trnG-UCC"

/product="tRNA-Gly"

exon 34532..34554

/gene="trnG-UCC"

/number=1

exon 34532..34552

/gene="trnG-UCC"

/number=1

intron 34553..35252

/gene="trnG-UCC"

/number=1

intron 34555..35235

/gene="trnG-UCC"

/number=1

intron 34563..35224

/gene="trnS-CGA"

/number=1

exon 35225..35284

/gene="trnS-CGA"

/number=2

exon 35236..35283

/gene="trnG-UCC"

/number=2

exon 35253..35283

/gene="trnG-UCC"

/number=2

gene 35460..35531

/gene="trnR-UCU"

tRNA 35460..35531

/gene="trnR-UCU"

/product="tRNA-Arg"

gene complement(35628..37151)

/gene="atpA"

CDS complement(35628..37151)

/gene="atpA"

/codon_start=1

/transl_table=11

/product="CF1 subunit alpha"

/translation="MVTIQADEISNILRERIEQYNREVKIVNTGTVLQVGDGIARIYG

LDEVMAGELVEFEEGTIGIALNLESNNVGVVLMGDGLMIQEGSSVKATGRIAQIPVSE

AYLGRVINALAKPIDGRGEISASESRLIESPAPGIISRRSVYEPLQTGLIAIDSMIPI

GRGQRELIIGDRQTGKTAVATDTILNQQGQNVICVYVAIGQKASSVAQVVTTLQERGA

MEYTIVVAEMADSPATLQYLAPYTGAALAEFFMYRKQHTLIIYDDPSKQAQAYRQMSL

LLRRPPGREAYPGDVFYLHSRLLERAAKSSSSLGEGSMTALPIVETQSGDVSAYIPTN

VISITDGQIFLSADLFNAGIRPAINVGISVSRVGSAAQIKAMKQVAGKLKLELAQFAE

LEAFAQFASDLDKATQNQLARGQRLRELLKQSQAAPLAVEEQIMTIYTGTNGYLDSLE

IGQVRKFIVELRTYLKSNKPQFQEIISSTKIFTEEAEALLKEAIQEQMDRFLLQEQA"

gene complement(37238..38446)

/gene="atpF"

CDS complement(join(37238..37648,38303..38446))

/gene="atpF"

/codon_start=1

/transl_table=11

/product="CF0 subunit I"

/translation="MKNVTDSFVSLVHWPSAGSFGFNTDILATNPINLSVVIGVLIFF

GKGVLSDLLDNRKQRILNTIRNSEELRGGAIEQMEKARARLRKVEMEADQFRVNGYSE

IEQEKWNLINSTYNTLEQLENYKNETIQFEQQRAINQVRQRVFQQALQGAIETLNSCL

NNELHLRTISANIGLLGAMKERTD"

CDS complement(join(37238..37647,38302..38446))

/gene="atpF"

/codon_start=1

/transl_table=11

/product="CF0 subunit I"

/translation="MKNVTDSFVSLVHWPSAGSFGFNTDILATNPINLSVVIGVLIFF

GKGVLSDLLDNRKQRILNTIRNSEELRGGAIEQMEKARARLRKVEMEADQFRVNGYSE

IEQEKWNLINSTYNTLEQLENYKNETIQFEQQRAINQVRQRVFQQALQGAIETLNSCL

NNELHLRTISANIGLLGAMKERTD"

exon complement(37238..37648)

/gene="atpF"

/number=2

exon complement(37238..37647)

/gene="atpF"

/number=2

intron complement(37648..38301)

/gene="atpF"

/number=1

intron complement(37649..38302)

/gene="atpF"

/number=1

exon complement(38302..38446)

/gene="atpF"

/number=1

exon complement(38303..38446)

/gene="atpF"

/number=1

gene complement(38807..39052)

/gene="atpH"

CDS complement(38807..39052)

/gene="atpH"

/codon_start=1

/transl_table=11

/product="CF0 subunit III"

/translation="MNPLISAASVIAAGLAVGLASIGPGVGQGTAAGQAVEGIARQPE

AEGKIRGTLLLSLAFMEALTIYGLVVALALLFANPFV"

gene complement(40039..40782)

/gene="atpI"

CDS complement(40039..40782)

/gene="atpI"

/codon_start=1

/transl_table=11

/product="CF0 subunit IV"

/translation="MNVISCSSNTLKGLYDISGVEVGQHFYWQIGGFQVHGQVLITSW

VVIAILLGSATIAVRNPQTIPTGGQNFFEYVLEFIRDVSKTQIGEEYGPWVPFIGTMF

LFIFVSNWSGALLPWKIIELPHGELAAPTNDINTTVALALLTSAAYFYAGLSKKGLSY

FGKYIQPTPILLPINILEDFTKPLSLSFRLFGNILADELVVVVLVSLVPSVIPIPVMF

LGLFTSGIQALIFATLAAAYIGESMEGHH"

gene complement(41013..41723)

/gene="rps2"

CDS complement(41013..41723)

/gene="rps2"

/codon_start=1

/transl_table=11

/product="ribosomal protein S2"

/translation="MTRRYWNINLDEMLEAGVHFGHGTRKWNPKMAPYISAKRKGIHI

TNLTKTARFLSEACDLVFDAASRGKQFLIVGTKKKAADSVARAAIKARCHCVNKKWLG

GMLTNWSTTETRLHKFRDLRMEQKTGRLNRLPKRDAAVVKRQLYRLQTYLGGIKYMTG

LPDIVIIVDQHEEYTALRECITLGIPTICLIDTNCDPDLADISIPANDDAISSIRLIL

NKLVFAISEGHSSYIRNP"

gene complement(41942..46120)

/gene="rpoC2"

CDS complement(41942..46120)

/gene="rpoC2"

/codon_start=1

/transl_table=11

/product="RNA polymerase subunit beta''"

/translation="MAERANLVFHNKVIDGTAMKRLISRLIDHFGMAYTSHILDQVKT

LGFQQATATSISLGIDDLLTIPSKKWLVQDAEQQSLILEKHHHYGNVHAVEKLRQSIE

IWYATSEYLRQEMNPNFRMTDPLNPVHIMSFSGARGNASQVHQLVGMRGLMSDPQGQM

IDLPIQSNLREGLSLTEYIISCYGARKGVVDTAVRTSDAGYLTRRLVEVVQHIVVRRI

DCGTARGISVSPQNGMMPERIFIQTLIGRVLADDIYMGTRCIATRNQDIGIGLVNRFI

TFRAQPIAIRTPFTCRSASWICRLCYGRSSTHGDLVELGEAVGIIAGQSIGEPGTQLT

LRTFHTGGVFTGGTAEHVRAPSNGKIKFNEDLVHPTRTRHGHPALLCSINLYVTIESE

DIRHNVNIPPQSFLLVQNDQYVESEQVIAEIRAGTSTFNFKEKIRKHIYSDSDGEMHW

STDVYHAPEFTYGSVHLLPKTSHLWILLGEPRGSSLVSLSIHKDQDQMSANSRSVKSF

NLSGTDDQLREKFFTSDFSGKKEDRIPDYSDLSRIICTGCCNLVDPTLLYHNSDLFSK

RRRNRFIIPLQSIQERENGLTPPSDILIEIPINGIFRRNSILAYLDDPRYRRKSSGIT

KYGTLEMHSIVKKEDLIEYRGGKEFSPKYQMKVDRFFFIPEEVHILPGSSSIMVRNNS

IIGVDTQITLNMRSRVAGLVRVERKKKRIELKIFSGDIHFPGETDKISRHSGVLIPLR

TGKRNSKESKKRENWIYVQRITPSKKKYFVLVRPVVTYEITDGITLGTLFPPDLLQER

DNVKLRVVNYILYGNGKPIRGISDTNIQLVRTCLVLNWDQDKKSSSSQEARASFVEIR

VNGLIRHLLRIDLLKSTFSYIGKRNDPSGSGLFSDNGSDCTKINPFSSIYSKAIIQQP

LNQNKRTIHTLLNRNAGFQSLIILSSSNCFRMGPFNDVKYHNHNVIHNGIKESINITK

DPVIPIQNSLGPLGTVPLIRIANVYSFYHLITHNQILVKNYLQLDNFKQTFQVIKLKY

YLIDEKEKIYNPDPCSNIICNLNWYFFHPNYCQETSTIMSLGQFICENIYISKSAPHL

KSGQVILVQVDSVVIRSAKTYLATPGATVHGHYGEILYEGDTLITFIYEKSRSGDITQ

GLPKVEQVLEVRSIDSISMNLDKRIEGWNERITRILGMPWAFLIGAELTIVQSRISLV

NKIQKVYRSQGVQIHNRHIEIIVRQITSRVLVSEDGMSNVFSPGELIGLVRAERMGRA

LEEAVCYRALLLGITRASLNTQSFISEASFQETARVLAKAALRGRIDWLKGLKENVVL

GGMIPVGTGLKGFVPPSKQHNRSPLEMETKKKNLFEGEMRDILFHHRKLFDSFLSNNL

DDRPEQSFIGFNDS"

gene complement(46287..49123)

/gene="rpoC1"

CDS complement(join(46287..47899,48694..49123))

/gene="rpoC1"

/codon_start=1

/transl_table=11

/product="RNA polymerase subunit beta'"

/translation="MIDRYKHQQLRIGLVSPQQISAWATKILPNGEIVGEVTKPYTFH

YKTNKPEKGGLFCERIFGPIKSGICACGNYRVIGDEKEDPKFCEQCGVEFVDSRIRRY

QMGYIKLACPVTHVWYLKRLPSYIANLLDKPLKELEGLVYCDFSFARPITKKPTFLRL

RGLFEYEIQSWKYSIPLFFTTQGFDTFRNREISTGASAIREQLADLDLRTILHNSLVE

WKELGEEGPTGNEWEDRKVGRRKDFLVRRMELAKHFLRTNIEPEWMVLCLLPVLPPEL

RPIIQIDGGKLMSSDINELYRRVIYRNNTLTDLLTTSRSTPGELVMCQEKLVQEAVDT

LLDNGIRGQPTRDGHNKAYKSFSDVIEGKEGRFRETLLGKRVDYSGRSVIVVGPSLSL

HRCGLPREIAIELFQTFVIRGLIRQHLASNIGVAKSKIREKEPIVWEILQEVMQGHPV

LLNRAPTLHKLGIQAFQPILVEGRAICLHPLVCKGFNADFDGDQMAVHVPLSLEAQAE

ARLLMFSHMNLLSPAIGDPISVPTQDMLIGLYVLTSGNRRGICINRYNPWNLKNYQNK

RSDNNKYKYTKEPFFSNSYDAIGAYRQKRINLDSPLWLRWRLDQRVIASRETPIEVHY

ESLGNYYEIYGHYLILRSIKKEILFLYIRTTVGHISLYREIEEAIQGFSRACSYGT"

CDS complement(join(46287..47897,48692..49123))

/gene="rpoC1"

/codon_start=1

/transl_table=11

/product="RNA polymerase subunit beta'"

/translation="MIDRYKHQQLRIGLVSPQQISAWATKILPNGEIVGEVTKPYTFH

YKTNKPEKGGLFCERIFGPIKSGICACGNYRVIGDEKEDPKFCEQCGVEFVDSRIRRY

QMGYIKLACPVTHVWYLKRLPSYIANLLDKPLKELEGLVYCDFSFARPITKKPTFLRL

RGLFEYEIQSWKYSIPLFFTTQGFDTFRNREISTGASAIREQLADLDLRTILHNSLVE

WKELGEEGPTGNEWEDRKVGRRKDFLVRRMELAKHFLRTNIEPEWMVLCLLPVLPPEL

RPIIQIDGGKLMSSDINELYRRVIYRNNTLTDLLTTSRSTPGELVMCQEKLVQEAVDT

LLDNGIRGQPTRDGHNKAYKSFSDVIEGKEGRFRETLLGKRVDYSGRSVIVVGPSLSL

HRCGLPREIAIELFQTFVIRGLIRQHLASNIGVAKSKIREKEPIVWEILQEVMQGHPV

LLNRAPTLHKLGIQAFQPILVEGRAICLHPLVCKGFNADFDGDQMAVHVPLSLEAQAE

ARLLMFSHMNLLSPAIGDPISVPTQDMLIGLYVLTSGNRRGICINRYNPWNLKNYQNK

RSDNNKYKYTKEPFFSNSYDAIGAYRQKRINLDSPLWLRWRLDQRVIASRETPIEVHY

ESLGNYYEIYGHYLILRSIKKEILFLYIRTTVGHISLYREIEEAIQGFSRACSYGT"

exon complement(46287..47899)

/gene="rpoC1"

/number=2

exon complement(46287..47897)

/gene="rpoC1"

/number=2

intron complement(47898..48691)

/gene="rpoC1"

/number=1

intron complement(47900..48693)

/gene="rpoC1"

/number=1

exon complement(48692..49123)

/gene="rpoC1"

/number=1

exon complement(48694..49123)

/gene="rpoC1"

/number=1

gene complement(49150..52362)

/gene="rpoB"

CDS complement(49150..52362)

/gene="rpoB"

/codon_start=1

/transl_table=11

/product="RNA polymerase subunit beta"

/translation="MLGDANEAMSTIPGFHQIQFEGFCRFINRGLTEELYKFPKIEDT

DHEIEFQLFLERYQLVEPLIKERNAVYESLTYSSELYVSAGLIWKTSRDMQEQTILVG

NIPLMNSLGTSIVNGIYRIVINQILQSPGIYYRSELDHNGIAVYTGTIISDWGGRSEL

EIDRKARIWARVSRKQKISILVLSSAMGSNLREILDNVYYPEIFLSFLNDKERKKIGS

KENAILEFYQQFACVGGDPVFSESLCKELQKKFFQQRCELGRIGRRNMNRRLNLDIPS

NNTFLLPRDILAAADHLIGLKFGMGTLDDMNHLKNKRIRSVADLLQDQFGLSLVRLEN

VVRGTICGAIRHKLIPTPQNLVTSTPLTTTYESFFGLHPLSQVLDRTNPLTQIVHGRK

LSYLGPGGLTGRTASFRIRDIHPSHYGRICPIDTSEGINVGLIGSLAIHARMGYWGSL

ESPFYKISERSTGLRLLYLSPGRDEYYMLAAGNSLALNQDIQEEQVVPARYRQEFLTI

AWERVHLRSIFPFQYFSIGASLIPFIEHNDANRALMSSNMQRQAVPLSRSEKCIVGTG

LERQAALDSGALAIAERGGKIIYIDTDKILFSGDGDTLSISLVMYQRSNKNTCMHQKT

RVQRGKCIKKGQILADGAATVGGELALGKNVLVAYMPWEGYNSEDAVLISERLVYEDI

YTSFHIRKYEIQTHATSQGPERITNEIPHLEARLLRNLDKNGIVMLGSWVETGDILVG

KLTPQMVKESSYAPEDRLLRAILGIQVSTSKETCLKLPIGGRGRVIDVRWIQKRGGSS

YNPEMIRVYISQKREIKVGDKVAGRHGNKGIISKILPRQDMPYLQDGRPIDMVFNPLG

VPSRMNVGQIFECSLGLAGGLLDRHYRIAPFDERYEQEASRKLVFSELYEASKQTANP

WVFEPEYPGKSRIFDGRAGSPFEQPVIIGKPYILKLIHQVDDKIHGRSSGHYALVTQQ

PLRGRAKQGGQRVGEMEVWALEGFGVAHILQEMLTYKSDHIRARQEVLGTTIIGGIIP

NPEDAPESFRLLVRELRSLALELNHFLVSEKNFQINRKEA"

gene 53502..53572

/gene="trnC-GCA"

tRNA 53502..53572

/gene="trnC-GCA"

/product="tRNA-Cys"

gene 54410..54499

/gene="petN"

CDS 54410..54499

/gene="petN"

/codon_start=1

/transl_table=11

/product="cytochrome b6/f subunit N"

/translation="MDIVSLAWAALMVVFTFSLSLVVWGRSGL"

gene complement(55297..55401)

/gene="psbM"

CDS complement(55297..55401)

/gene="psbM"

/codon_start=1

/transl_table=11

/product="photosystem II subunit M"

/translation="MEVNILAFIATALFILVPTAFLLIIYVKTVSQND"

gene complement(55931..56007)

/gene="trnD-GUC"

tRNA complement(55931..56007)

/gene="trnD-GUC"

/product="tRNA-Asp"

gene complement(55933..56006)

/gene="trnD-GUC"

tRNA complement(55933..56006)

/gene="trnD-GUC"

/product="tRNA-Asp"

gene complement(56119..56204)

/gene="trnY-GUA"

tRNA complement(56119..56204)

/gene="trnY-GUA"

/product="tRNA-Tyr"

gene complement(56120..56203)

/gene="trnY-GUA"

tRNA complement(56120..56203)

/gene="trnY-GUA"

/product="tRNA-Tyr"

gene complement(56263..56335)

/gene="trnE-UUC"

tRNA complement(56263..56335)

/gene="trnE-UUC"

/product="tRNA-Glu"

gene 56922..56995

/gene="trnT-GGU"

tRNA 56922..56995

/gene="trnT-GGU"

/product="tRNA-Thr"

gene 56923..56994

/gene="trnT-GGU"

tRNA 56923..56994

/gene="trnT-GGU"

/product="tRNA-Thr"

gene 58184..59245

/gene="psbD"

CDS 58184..59245

/gene="psbD"

/codon_start=1

/transl_table=11

/product="photosystem II protein D2"

/translation="MTIALGKFTKDEKDLFDIMDDWLRRDRFVFVGWSGLLLFPCAYF

ALGGWFTGTTFVTSWYTHGLASSYLEGCNFLTAAVSTPANSLAHSLLLLWGPEAQGDF

TRWCQLGGLWTFVALHGAFALIGFMLRQFELARSVQLRPYNAIAFSGPIAVFVSVFLI

YPLGQSGWFFAPSFGVAAIFRFILFFQGFHNWTLNPFHMMGVAGVLGAALLCAIHGAT

VENTLFEDGDGANTFRAFNPTQAEETYSMVTANRFWSQIFGVAFSNKRWLHFFMLFVP

VTGLWMSALGVVGLALNLRAYDFVSQEIRAAEDPEFETFYTKNILLNEGIRAWMAAQD

QPHENLIFPEEVLPRGNAL"

gene 59193..60614

/gene="psbC"

CDS 59193..60614

/gene="psbC"

/codon_start=1

/transl_table=11

/product="photosystem II 43 kDa protein"

/translation="MKTLYSLRRFYHVETLFNGTLSVAGRDQETTGFAWWAGNARLIN

LSGKLLGAHVAHAGLIVFWAGAMNLFEVAHFVPEKPMYEQGLILLPHLATLGWGVGPG

GEVIDTFPYFVSGVLHLISSAVLGFGGIYHALLGPETLEESFPFFGYVWKDRNKMTTI

LGIHLILLGLGAFLLVFKALYFGGVYDTWAPGGGDVRKITNLTLSPSIIFGYLLKSPF

GGEGWIVSVDDLEDIIGGHVWLGSICILGGIWHILTKPFAWARRALVWSGEAYLSYSL

GALAIFGFTACCFVWFNNTAYPSEFYGPTGPEASQAQAFTFLVRDQRLGANVGSAQGP

TGLGKYLMRSPTGEVIFGGETMRFWDLRAPWLEPLRGPNGLDLSRLKKDIQPWQERRS

AEYMTHAPLGSLNSVGGVATEINAVNYVSPRSWLATSHFVLGFFFFVGHLWHAGRARA

AAAGFEKGIDRDFEPVLFMTPLN"

gene 59229..60614

/gene="psbC"

CDS 59229..60614

/gene="psbC"

/codon_start=1

/transl_table=11

/product="photosystem II 43 kDa protein"

/translation="METLFNGTLSVAGRDQETTGFAWWAGNARLINLSGKLLGAHVAH

AGLIVFWAGAMNLFEVAHFVPEKPMYEQGLILLPHLATLGWGVGPGGEVIDTFPYFVS

GVLHLISSAVLGFGGIYHALLGPETLEESFPFFGYVWKDRNKMTTILGIHLILLGLGA

FLLVFKALYFGGVYDTWAPGGGDVRKITNLTLSPSIIFGYLLKSPFGGEGWIVSVDDL

EDIIGGHVWLGSICILGGIWHILTKPFAWARRALVWSGEAYLSYSLGALAIFGFTACC

FVWFNNTAYPSEFYGPTGPEASQAQAFTFLVRDQRLGANVGSAQGPTGLGKYLMRSPT

GEVIFGGETMRFWDLRAPWLEPLRGPNGLDLSRLKKDIQPWQERRSAEYMTHAPLGSL

NSVGGVATEINAVNYVSPRSWLATSHFVLGFFFFVGHLWHAGRARAAAAGFEKGIDRD

FEPVLFMTPLN"

gene complement(60848..60940)

/gene="trnS-UGA"

tRNA complement(60848..60940)

/gene="trnS-UGA"

/product="tRNA-Ser"

gene 61265..61453

/gene="psbZ"

CDS 61265..61453

/gene="psbZ"

/codon_start=1

/transl_table=11

/product="photosystem II subunit Z"

/translation="MTLVFQLAVFALIATSSILLISVPVVFASPDGWSSNKNVVFSGT

SLWIGLVFLVGILNSLIS"

gene 61684..61754

/gene="trnG-GCC"

tRNA 61684..61754

/gene="trnG-GCC"

/product="tRNA-Gly"

gene complement(61907..61981)

/gene="trnM-CAU"

tRNA complement(61907..61981)

/gene="trnM-CAU"

/product="tRNA-Met"

gene complement(61908..61981)

/gene="trnfM-CAU | trnM-CAU"

tRNA complement(61908..61981)

/gene="trnfM-CAU | trnM-CAU"

gene complement(62131..62433)

/gene="rps14"

CDS complement(62131..62433)

/gene="rps14"

/codon_start=1

/transl_table=11

/product="ribosomal protein S14"

/translation="MARKSLIQREKKRQKLEQKYHLIRRSSKKEISKVPSLSDKWQIY

GKLQSPPRNSAPTRLHRRCFSTGRPRANYRDFGLSGHILREMVHACLLPGATRSSW"

gene complement(62561..64765)

/gene="psaB"

CDS complement(62561..64765)

/gene="psaB"

/codon_start=1

/transl_table=11

/product="photosystem I P700 apoprotein A2"

/translation="MALRFPRFSQGLAQDPTTRRIWFGIATAHDFESHDDITEERLYQ

NIFASHFGQLAIIFLWTSGNLFHVAWQGNFESWVQDPLHVRPIAHAIWDPHFGQPAVE

AFTRGGALGPVNIAYSGVYQWWYTIGLRTNEDLYTGALFLLFLSAISLIAGWLHLQPK

WKPSVSWFKNAESRLNHHLSGLFGVSSLAWTGHLVHVAIPGSRGEYVRWNNFLDVLPH

PQGLGPLFTGQWNLYAQNPDSSNHLFGTSQGAGTAILTLLGGFHPQTQSLWLTDMAHH

HLAIAFIFLVAGHMYRTNFGIGHSIKDLLDAHVPPGGRLGRGHKGLYDTINNSLHFQL

GLALASLGVITSLVAQHMYSLPAYAFIAQDFTTQAALYTHHQYIAGFIMTGAFAHGAI

FFIRDYNPEQNEDNVLARMLDHKEAIISHLSWASLFLGFHTLGLYVHNDVMLAFGTPE

KQILIEPIFAQWIQSAHGKTAYGFDVLLSSTSGPAFTAGRSIWLPGWLNAVNENTNSL

FLTIGPGDFLVHHAIALGLHTTTLILVKGALDARGSKLMPDKKDFGYSFPCDGPGRGG

TCDISAWDAFYLAVFWMLNTIGWVTFYWHWKHITLWQGNVSQFNESSTYLMGWLRDYL

WLNSSQLINGYNPFGMNSLSVWAWMFLFGHLVWATGFMFLISWRGYWQELIETLAWAH

ERTPLANLIRWRDKPVALSIVQARLVGLAHFSVGYIFTYAAFLIASTSGKFG"

gene complement(64791..67043)

/gene="psaA"

CDS complement(64791..67043)

/gene="psaA"

/codon_start=1

/transl_table=11

/product="photosystem I P700 apoprotein A1"

/translation="MIIRSPEPEVKILVDKDPVKTSFEQWAKPGHFSRTIAKGPDTTT

WIWNLHADAHDFDSHTSDLEEISRKVFSAHFGQLSIIFLWLSGMYFHGARFSNYEAWL

SDPTHIGPSAQVVWPIVGQEILNGDVGGGFRGIQITSGFFQIWRASGITNELQLYCTA

IGALVFAALMLFAGWFHYHKAAPKLAWFQDVESMLNHHLAGLLGLGSLSWAGHQVHVS

LPINQFLNAGVDPKEIPLPHEFILNRDLLAQLYPSFAEGATPFFTLNWSKYAEFLTFR

GGLDPVTGGLWLTDIAHHHLAIAILFLIAGHMYRTNWGIGHGLKDILEAHKGPFTGQG

HKGLYEILTTSWHAQLSLNLAMLGSLTIVVAHHMYSMPPYPYLATDYGTQLSLFTHHM

WIGGFLIVGAAAHAAIFMVRDYDPTTRYNDLLDRVLRHRDAIISHLNWACIFLGFHSF

GLYIHNDTMSALGRPQDMFSDTAIQLQPVFAQWIQNTHALAPGATAPGATASTSLTWG

GGDLVAVGGKVALLPIPLGTADFLVHHIHAFTIHVTVLILLKGVLFARSSRLIPDKAN

LGFRFPCDGPGRGGTCQVSAWDHVFLGLFWMYNSISVVIFHFSWKMQSDVWGSISDQG

VVTHITGGNFAQSSITINGWLRDFLWAQASQVIQSYGSSLSAYGLFFLGAHFVWAFSL

MFLFSGRGYWQELIESIVWAHNKLKVAPATQPRALSIVQGRAVGVTHYLLGGIATTWA

FFLARIIAVG"

gene complement(67769..69705)

/gene="pafI"

CDS complement(join(67769..67921,68643..68872,69582..69705))

/gene="pafI"

/codon_start=1

/transl_table=11

/product="photosystem I assembly factor I"

/translation="MPRSRINGNFIDKTFSIVANILLQIIPTTSGEREAFTYYRDGMS

AQSEGNYAEALQNYYEAMRLEIDPYDRSYILYNIGLIHTSNGEHTKALEYYFRALERN

PFLPQAFNNMAVICHYRGEQAILQGDSEIAEAWFDQAAEYWKQAIALTPGNYIEAHNW

LKITRRFE"

CDS complement(join(67769..67923,68645..68870,69580..69705))

/gene="pafI"

/codon_start=1

/transl_table=11

/product="photosystem I assembly factor I"

/translation="MPRSRINGNFIDKTFSIVANILLQIIPTTSGEREAFTYYRDGMS

AQSEGNYAEALQNYYEAMRLEIDPYDRSYILYNIGLIHTSNGEHTKALEYYFRALERN

PFLPQAFNNMAVICHYRGEQAILQGDSEIAEAWFDQAAEYWKQAIALTPGNYIEAHNW

LKITRRFE"

exon complement(67769..67923)

/gene="pafI"

/number=3

exon complement(67769..67921)

/gene="pafI"

/number=3

intron complement(67922..68642)

/gene="pafI"

/number=2

intron complement(67924..68644)

/gene="pafI"

/number=2

exon complement(68643..68872)

/gene="pafI"

/number=2

exon complement(68645..68870)

/gene="pafI"

/number=2

intron complement(68871..69579)

/gene="pafI"

/number=1

intron complement(68873..69581)

/gene="pafI"

/number=1

exon complement(69580..69705)

/gene="pafI"

/number=1

exon complement(69582..69705)

/gene="pafI"

/number=1

gene 70022..70108

/gene="trnS-GGA"

tRNA 70022..70108

/gene="trnS-GGA"

/product="tRNA-Ser"

gene complement(70393..70998)

/gene="rps4"

CDS complement(70393..70998)

/gene="rps4"

/codon_start=1

/transl_table=11

/product="ribosomal protein S4"

/translation="MSRYRGPRFKKIRRLGALPGLTNKRPKAGSDLRNQSRSGKKSQY

RIRLEEKQKLRFHYGLTERQLLKYVRIAGKARGSTGQVLLQLLEMRLDNILFRLGMAS

TIPAARQLVNHRHILVNGRIVDIPSYRCKPRDIITGKDEQKSRTLIQNSLNSSPQAEV

PNHLTLHPFQYKGLVNQIIDSKWVGLKINELLVVEYYSRQT"

gene complement(71387..71461)

/gene="trnT-UGU"

tRNA complement(71387..71461)

/gene="trnT-UGU"

/product="tRNA-Thr"

gene complement(71388..71460)

/gene="trnT-UGU"

tRNA complement(71388..71460)

/gene="trnT-UGU"

/product="tRNA-Thr"

gene 72128..72700

/gene="trnL-UAA"

tRNA join(72128..72164,72651..72700)

/gene="trnL-UAA"

/product="tRNA-Leu"

tRNA join(72128..72162,72651..72700)

/gene="trnL-UAA"

/product="tRNA-Leu"

exon 72128..72164

/gene="trnL-UAA"

/number=1

exon 72128..72162

/gene="trnL-UAA"

/number=1

intron 72163..72650

/gene="trnL-UAA"

/number=1

intron 72165..72650

/gene="trnL-UAA"

/number=1

exon 72651..72700

/gene="trnL-UAA"

/number=2

gene 72978..73051

/gene="trnF-GAA"

tRNA 72978..73051

/gene="trnF-GAA"

/product="tRNA-Phe"

gene 72978..73050

/gene="trnF-GAA | trnP-GAA"

tRNA 72978..73050

/gene="trnF-GAA | trnP-GAA"

gene complement(73745..74221)

/gene="ndhJ"

CDS complement(73745..74221)

/gene="ndhJ"

/codon_start=1

/transl_table=11

/product="NADH dehydrogenase subunit J"

/translation="MQGRLSAWLVKHGIIHRSLGFDYQGIETLQIKPEDWHSIAVILY

VYGYNYLRSQCAYDVAPGGLLASVYHLTRIEYGVDQPEEVCVKVFTSRRNPRIPSVFW

VWKSVDFQERESYDMLGISYDNHPRLKRILMPESWIGWPLRKDYIAPNFYEIQDAH"

gene complement(74322..75062)

/gene="ndhK"

CDS complement(74322..75062)

/gene="ndhK"

/codon_start=1

/transl_table=11

/product="NADH dehydrogenase subunit K"

/translation="MVLAPEYSDNKKKGEKKIEKVMNPIEFPLLNRTAKISVISTTLN

DLSNWSRLSSLWPLLYGTSCCFIEFASLIGSRFDFDRYGLVPRSSPRQADLILTAGTV

TMKMAPSLVRLYEQMPDPKYVIAMGACTITGGMFSTDSYSTVRGVDKLIPVDVYLPGC

PPKPEAVIDAITKLRKKISREIYENRIRSQQANRCFTTNHKFHVGRSMNTGNYDQRFL

YQPPSTSEIPTENFFEYKSSVSSHELVN"

gene complement(74322..74999)

/gene="ndhK"

CDS complement(74322..74999)

/gene="ndhK"

/codon_start=1

/transl_table=11

/product="NADH dehydrogenase subunit K"

/translation="MNPIEFPLLNRTAKISVISTTLNDLSNWSRLSSLWPLLYGTSCC

FIEFASLIGSRFDFDRYGLVPRSSPRQADLILTAGTVTMKMAPSLVRLYEQMPDPKYV

IAMGACTITGGMFSTDSYSTVRGVDKLIPVDVYLPGCPPKPEAVIDAITKLRKKISRE

IYENRIRSQQANRCFTTNHKFHVGRSMNTGNYDQRFLYQPPSTSEIPTENFFEYKSSV

SSHELVN"

gene complement(75053..75415)

/gene="ndhC"

CDS complement(75053..75415)

/gene="ndhC"

/codon_start=1

/transl_table=11

/product="NADH dehydrogenase subunit C"

/translation="MFLLYEYDIFWAFLIISSLIPILAFFISGILAPIRKGPEKLSSY

ESGIEPMGDAWLQFRIRYYMFALVFVVFDVETVFLYPWAMSFDVLGVSVFIEALIFVL

ILIVGLVYAWRKGALEWS"

gene complement(76364..77011)

/gene="trnV-UAC"

tRNA complement(join(76364..76401,76974..77011))

/gene="trnV-UAC"

/product="tRNA-Val"

exon complement(76364..76401)

/gene="trnV-UAC"

/number=2

gene complement(76365..77011)

/gene="trnV-UAC | trnC-ACA"

tRNA complement(join(76365..76399,76974..77011))

/gene="trnV-UAC"

/product="tRNA-Val"

tRNA complement(join(76365..76420,76974..77011))

/gene="trnC-ACA"

exon complement(76365..76420)

/gene="trnC-ACA"

/number=2

exon complement(76365..76399)

/gene="trnV-UAC"

/number=2

intron complement(76400..76973)

/gene="trnV-UAC"

/number=1

intron complement(76402..76973)

/gene="trnV-UAC"

/number=1

intron complement(76421..76973)

/gene="trnC-ACA"

/number=1

exon complement(76974..77011)

/gene="trnV-UAC | trnC-ACA"

/number=1

gene 77200..77272

/gene="trnM-CAU"

tRNA 77200..77272

/gene="trnM-CAU"

/product="tRNA-Met"

gene complement(77490..77891)

/gene="atpE"

CDS complement(77490..77891)

/gene="atpE"

/codon_start=1

/transl_table=11

/product="CF1 subunit epsilon"

/translation="MTLNLCVLTPNRIVWDSEVKEIILSTNSGQIGVLPNHAPVATAV

DIGILRIRLKDQWLTMALMGGFARIGNNEITVLVNDAEKGSDIDPQEAQQTLEIAEAN

LRKAEGKRQIIEANLALRRARTRVEAVNVIS"

gene complement(77888..79384)

/gene="atpB"

CDS complement(77888..79384)

/gene="atpB"

/codon_start=1

/transl_table=11

/product="CF1 subunit beta"

/translation="MVSNPTTSSSGVSTLEKKNQGNIIQIIGPVLDVAFPPGKMPNIY

NALVVKGQDTAGQPINVTCEVQQLLGNNRVRAVAMSATDGLMRGMEVIDTGAPLSVPV

GGATLGRIFNVLGEPVDNLGPVDTRTTFPIHRSAPAFIQLDTKLSIFETGIKVVDLLA

PYRRGGKIGLFGGAGVGKTVLIMELINNIAKAHGGVSVFGGVGERTREGNDLYMEMKE

SGVINEENIEESKVALVYGQMNEPPGARMRVGLTALTMAEYFRDVNEQDVLLFIDNIF

RFVQAGSEVSALLGRMPSAVGYQPTLSTEMGSLQERITSTKEGSITSIQAVYVPADDL

TDPAPATTFAHLDATTVLSRGLAAKGIYPAVDPLDSTSTMLQPRIVGEEHYEIAQRVK

QTLQRYKELQDIIAILGLDELSEEDRLTVARARKIERFLSQPFFVAEVFTGSPGKYVG

LAETIRGFQLILSGELDGLPEQAFYLVGNIDEATAKAMNLEMESNLKK"

gene 80193..81638

/gene="rbcL"

CDS 80193..81638

/gene="rbcL"

/codon_start=1

/transl_table=11

/product="ribulose-1,5-bisphosphate carboxylase/oxygenase

large subunit"

/translation="MSPQTETKASVGFKAGVKEYKLTYYTPEYETKDTDILAAFRVTP

QPGVPPEEAGAAVAAESSTGTWTTVWTDGLTSLDRYKGRCYHIEPVLGEKDQYICYVA

YPLDLFEEGSVTNMFTSIVGNVFGFKALRALRLEDLRIPTAYTKTFQGPPHGIQVERD

KLNKYGRPLLGCTIKPKLGLSAKNYGRAVYECLRGGLDFTKDDENVNSQPFMRWRDRF

LFCAEAIYKAQAETGEIKGHYLNATAGTCEDMMKRAVFARELGVPIVMHDYLTGGFTA

NTTLAHYCRDNGLLLHIHRAMHAVIDRQKNHGMHFRVLAKALRLSGGDHIHAGTVVGK

LEGEREITLGFVDLLRDDYIEKDRSRGIYFTQDWVSLPGVIPVASGGIHVWHMPALTE

IFGDDSVLQFGGGTLGHPWGNAPGAVANRVALEACVQARNEGRDLASEGNAIIREACK

WSPELAAACEVWKEIKFEFKPVDTLDPEKTN"

gene 82334..83800

/gene="accD"

CDS 82334..83800

/gene="accD"

/codon_start=1

/transl_table=11

/product="acetyl-CoA carboxylase subunit beta"

/translation="MERCWFNSMVFKKELERRYGIKKLTDNLGPMENTSEGEDPNRKG

RAKNMHSWSGRDNSSYSNVDPLFGAKDIRNFISDDTFLVRDNNGDSYSIYFDIENHIF

EIDNEHCFRSELESSFSSYRNSNYMNNGSTNEDSLYTRYMYDTQSSWNNHITSCIDSY

LQSQICIDTPTVSDSSDSYISRCVFQKRKTSSEPSIRTRAKSSDLTLRASDLDETQKY

KQLWVQCENCYALNYKKLLKSQINICEQCGYHLKMSSSERIEVLIDPGTWDPMDEDMV

SGDPIGFHSEEETYKDRIDFYQRNIGLTEAVQTGVGQLNGIPVAIGVMDFKFMGGSMG

SVVGEKITRLIEYATNKFIPLIIVCASGGARMQEGSLSLMQMAKISSALYDYQSNKKL

LYVSILTSPTTGGVTASFGMLGDIIIAEPNSYIAFAGKRVIEQTLNKTIPEGSQASEY

LFQKGLFDLIVPRNLLKSVLSELFKLHAFFPLNSNSMH"

gene 84513..84623

/gene="psaI"

CDS 84513..84623

/gene="psaI"

/codon_start=1

/transl_table=11

/product="photosystem I subunit I"

/translation="MTTFQFPSIFVPLVGLVFPAIAMASLFLHVQKNKIV"

gene 85056..85610

/gene="pafII"

CDS 85056..85610

/gene="pafII"

/codon_start=1

/transl_table=11

/product="photosystem I assembly factor II"

/translation="MSWRSEHIWIELITGSRKLSNFCWALVVFLGSLGFLLVGTSSYL

GRNLISFVPSQQILFFPQGIVMSFYGIAGLFISSYLWCTISWNVGSGYDQFDRKEGMV

CIFRWGFPGKNRRIFLRFRIKDIQSVRIEVKEGIYARRILYMDIRGRGAIPLTRTDEN

LTPREIEQKAAELAYFLRVPIEVF"

gene 86171..86860

/gene="cemA"

CDS 86171..86860

/gene="cemA"

/codon_start=1

/transl_table=11

/product="envelope membrane carbon uptake protein"

/translation="MAKKKAFTPLLYLASIVFLPWWISLSFTKSMESWVTSWSNTVQS

ENFVNDMQEKSILEKFIELEEMLFLEEMIKEYSETHLQKLPIEIHKETIQLIKIHNED

RIHTILHFSTNLICFVILAGYSILGNQELVILNSWTQEFLYNLSDTVKAFLILLLTDL

CIGFHSPHGWELMIGSVYKDFGFDHNDQIISGLVSTFPVILDTILKYWIFHYLNRVSP

SLVVIYHSMND"

gene 87077..88039

/gene="petA"

CDS 87077..88039

/gene="petA"

/codon_start=1

/transl_table=11

/product="cytochrome f"

/translation="MQTRKTFSWIKEHITRSISVSLMIYIITRTSISSAYPIFAQQGY

ENPREATGRIVCANCHLANKPVDIEVPQAVLPDTVFEAVVRIPYDKQVKQVLANGKKG

GLNVGAVLILPEGFELAPPDRISPEMKEKIGNLSFQSYRPNKQNILVIGPVPGQKYSE

ITFPILSPDPATKKDVHFLKYPIYVGGNRGRGQIYPDGSKSNNTVYNATGAGIVSKIL

RKEKGGYEITITDPSDGREVVDIIPPGPEVLVSEGESIKLDQPLTSNPNVGGFGQGDA

EIVLQDPLRVQGLLVFLASVILAQIFLVLKKKQFEKVQLAEMNF"

gene complement(89035..89157)

/gene="psbJ"

CDS complement(89035..89157)

/gene="psbJ"

/codon_start=1

/transl_table=11

/product="photosystem II subunit J"

/translation="MADTTGRIPLWIIGTVAGILVIGLIGIFFYGSYSGLGSSL"

gene complement(89295..89411)

/gene="psbL"

CDS complement(89295..89411)

/gene="psbL"

/codon_start=1

/transl_table=11

/product="photosystem II subunit L"

/translation="MTQSNPNEQNVELNRTSLYWGLLLIFVLAVLFSNYFFN"

gene complement(89435..89554)

/gene="psbF"

CDS complement(89435..89554)

/gene="psbF"

/codon_start=1

/transl_table=11

/product="cytochrome b559 subunit beta"

/translation="MTIDRTYPIFTVRWLAVHGLAVPTVFFLGSISAMQFIQR"

gene complement(89569..89820)

/gene="psbE"

CDS complement(89569..89820)

/gene="psbE"

/codon_start=1

/transl_table=11

/product="cytochrome b559 subunit alpha"

/translation="MSGSTGERSFADIITSIRYWVIHSITIPSLFIAGWLFVSTGLAY

DVFGSPRPNEYFTESRQGIPLITGRFDPLEQLDEFSRSF"

gene 90745..90840

/gene="petL"

CDS 90745..90840

/gene="petL"

/codon_start=1

/transl_table=11

/product="cytochrome b6/f subunit L"

/translation="MLTITSYFGFLLVALTITSALFIGLSKIRLI"

gene 91020..91133

/gene="petG"

CDS 91020..91133

/gene="petG"

/codon_start=1

/transl_table=11

/product="cytochrome b6/f subunit G"

/translation="MIEVFLFGIVLGLIPITLAGLFVTAYLQYRRGDQLDL"

gene complement(91254..91327)

/gene="trnW-CCA"

tRNA complement(91254..91327)

/gene="trnW-CCA"

/product="tRNA-Trp"

gene complement(91488..91563)

/gene="trnP-UGG"

tRNA complement(91488..91563)

/gene="trnP-UGG"

/product="tRNA-Pro"

gene complement(91490..91563)

/gene="trnP-UGG"

tRNA complement(91490..91563)

/gene="trnP-UGG"

/product="tRNA-Pro"

gene 91837..91971

/gene="psaJ"

CDS 91837..91971

/gene="psaJ"

/codon_start=1

/transl_table=11

/product="photosystem I subunit J"

/translation="MRDLKTYLSVAPVLSTLWFGALAGLLIEINRFFPDALTFPFFSF

"

gene 92444..92644

/gene="rpl33"

CDS 92444..92644

/gene="rpl33"

/codon_start=1

/transl_table=11

/product="ribosomal protein L33"

/translation="MAKRKDARVTVILECTGCIQNGVKKVSTGISRYITQKNRHNMPN

RLELRKFCPYCYKHMIHVEIKK"

gene 92792..93097

/gene="rps18"

CDS 92792..93097

/gene="rps18"

/codon_start=1

/transl_table=11

/product="ribosomal protein S18"

/translation="MDKSKRSFLKSKRSFRRRLPPIQSGDRIDYRNMSLISRFISEQG

KILSRRVNRLTLKQQRLITIAIKQARILSLLPFLNNEKQFERMESTTRTTGLRTRNK"

gene complement(93342..93728)

/gene="rpl20"

CDS complement(93342..93728)

/gene="rpl20"

/codon_start=1

/transl_table=11

/product="ribosomal protein L20"

/translation="MTRIKRGYIARRRRTKIRLFASSFRGSHSRLTRTITQQKIRALV

SAHRDRDKQKRNFRRLWITRINAVIREGRVFYSYSKLINDLYKKQLLLNRKILAQIAI

SNRNCLYMISNEIRKKVDWKEYTGII"

exon complement(94488..94601)

/gene="rps12"

/number=1

gene complement(94728..96597)

/gene="clpP1"

CDS complement(join(94728..94955,95577..95868,96527..96597))

/gene="clpP1"

/codon_start=1

/transl_table=11

/product="ATP-dependent Clp protease proteolytic subunit

1"

/translation="MPIGVPKVPFRSPGEEDASWVDVYNRLYRERLLFLGQEVDSEIS

NQLIGLMVYLSIEDDTKDLYLFINSPGGWVIPGIAIYDTMQFVRPEVHTICMGLAASM

GSFILVGGEITKRLAFPHARVMIHQPASSFYEAQTGEFILEAEELLKLRETLTRVYVQ

RTGKPLWVVSEDMERDVFMSATEAQAYGIVDLVAVE"

CDS complement(join(94728..94952,95574..95868,96527..96597))

/gene="clpP1"

/codon_start=1

/transl_table=11

/product="ATP-dependent Clp protease proteolytic subunit

1"

/translation="MPIGVPKVPFRSPGEEDASWVDVYNRLYRERLLFLGQEVDSEIS

NQLIGLMVYLSIEDDTKDLYLFINSPGGWVIPGIAIYDTMQFVRPEVHTICMGLAASM

GSFILVGGEITKRLAFPHAWVMIHQPASSFYEAQTGEFILEAEELLKLRETLTRVYVQ

RTGKPLWVVSEDMERDVFMSATEAQAYGIVDLVAVE"

exon complement(94728..94955)

/gene="clpP1"

/number=3

exon complement(94728..94952)

/gene="clpP1"

/number=3

intron complement(94953..95573)

/gene="clpP1"

/number=2

intron complement(94956..95576)

/gene="clpP1"

/number=2

exon complement(95574..95868)

/gene="clpP1"

/number=2

exon complement(95577..95868)

/gene="clpP1"

/number=2

intron complement(95869..96526)

/gene="clpP1"

/number=1

exon complement(96527..96597)

/gene="clpP1"

/number=1

gene 97029..98555

/gene="psbB"

CDS 97029..98555

/gene="psbB"

/codon_start=1

/transl_table=11

/product="photosystem II 47 kDa protein"

/translation="MGLPWYRVHTVVLNDPGRLLSVHIMHTALVAGWAGSMALYELAV

FDPSDPVLDPMWRQGMFVIPFMTRLGITNSWGGWSITGATVTNPGVWSYEGVAGAHIF

FSGLCFLAAIWHWVYWDLEIFSDERTGKPSLDLPKIFGIHLFLSGVACFGFGAFHVTG

LYGPGIWVSDPYGLTGKVQPVNPAWGVEGFDPFVPGGIASHHIAAGTLGILAGLFHLS

VRPPQRLYKGLRMGNIETVLSSSIAAVFFAAFVVAGTMWYGSATTPIELFGPTRYQWD

QGYFQQEIYRRVSAGLAENQSLSEAWSKIPEKLAFYDYIGNNPAKGGLFRAGSMDSGD

GIAVGWLGHPIFRDKEGRELFVRRMPTFFETFPVVLVDGDGIVRADVPFRRAESKYSV

EQVGVTVEFYGGELNGVSYSDPATVKKYARRAQLGEIFELDRATLKSDGVFRSSPRGW

FTFGHASFALLFFFGHIWHGARTLFRDVFAGIDPDLDAQVEFGAFQKLGDPTTRRQAV

"

gene 98746..98847

/gene="psbT"

CDS 98746..98847

/gene="psbT"

/codon_start=1

/transl_table=11

/product="photosystem II subunit T"

/translation="MEALVYTFLLVSTLGIIFFAIFFREPPKVPTKK"

gene complement(98921..99052)

/gene="pbf1"

CDS complement(98921..99052)

/gene="pbf1"

/codon_start=1

/transl_table=11

/product="photosystem biogenesis factor 1"

/translation="METATLVAIFISGLLVSFTGYALYTAFGQPSQQLRDPFEEHGD"

gene 99156..99377

/gene="psbH"

CDS 99156..99377

/gene="psbH"

/codon_start=1

/transl_table=11

/product="photosystem II subunit H"

/translation="MATQTVENSSKSGPRRTVVGDLLKPLNSEYGKVAPGWGTTPLMG

VAMALFAVFLSIILEIYNSSVLLDGISMN"

gene 99502..100877

/gene="petB"

CDS join(99502..99507,100236..100877)

/gene="petB"

/codon_start=1

/transl_table=11

/product="cytochrome b6"

/translation="MSKVYDWFEERLEIQAIADDITSKYVPPHVNIFYCLGGITLTCF

LVQVATGFAMTFYYRPTVTEAFASVQYIMTEANFGWLIRSVHRWSASMMVLMMILHVF

RVYLTGGFKKPRELTWVTGVVLGVLTASFGVTGYSLPRDQIGYWAVKIVTGVPEAIPV

IGSPVVELLRGSASVGQSTLTRFYSLHTFVLPLLTAVFMLMHFPMIRKQGISGPL"

exon 99502..99507

/gene="petB"

/number=1

intron 99508..100235

/gene="petB"

/number=1

exon 100236..100877

/gene="petB"

/number=2

gene 101072..102291

/gene="petD"

CDS join(101072..101079,101817..102291)

/gene="petD"

/codon_start=1

/transl_table=11

/product="cytochrome b6/f subunit IV"

/translation="MGVTKKPDLNDPVLRAKLAKGMGHNYYGEPAWPNDLLYIFPVVI

LGTIACNVGLAVLEPSMIGEPADPFATPLEILPEWYFFPVFQILRTVPNKLLGVLLMV

SVPAGLLTVPFLENVNKFQNPFRRPVATTVFLIGTAVALWLGIGATLPIDKSLTLGLF

"

exon 101072..101079

/gene="petD"

/number=1

intron 101080..101816

/gene="petD"

/number=1

exon 101817..102291

/gene="petD"

/number=2

gene complement(102454..103467)

/gene="rpoA"

CDS complement(102454..103467)

/gene="rpoA"

/codon_start=1

/transl_table=11

/product="RNA polymerase subunit alpha"

/translation="MVREKVTVSTRTLQWKCVESRTDSKRLYYGRFILSPLMKGQADT

IGIAMRRALLGEIEGTCITRVKSENVPHEYSTITGIQESVHEIIMNLKEIVLRSNLYG

TCGASICARGPGYVTAQDMILPPYVEIVDNTQHIASLAEPINLCIGLEIERNRGYLIK

MPHTFQDGSYPIDAVFMPVRNVNHSIHSYENGTEKQEILFLEIWTNGSLTPKEALHEA

SRNLIDLFIPFLYKEEKNLPLEDNQYRLPLSPFTFHDKLDKVRKNKNKIALKSIFIDQ

SELSPRVYNCLKRSNIYTLLDLLNNSQEDLMKIEHFRLEDVKHILGILEKNFAIDLPK

NKF"

gene complement(103539..103955)

/gene="rps11"

CDS complement(103539..103955)

/gene="rps11"

/codon_start=1

/transl_table=11

/product="ribosomal protein S11"

/translation="MAKAIPRIGSRKNVRIGSRKSARRIPKGVIHVQASFNNTIVTVT

DVRGRVVSWSSSGTCGFKGTRRGTPFAAQTAATNAIRTVVDQGMQRAEVMIKGPGLGR

DAALRAIRRSGLLLTFVRDVTPMPHNGCRPPKKRRV"

gene complement(104058..104171)

/gene="rpl36"

CDS complement(104058..104171)

/gene="rpl36"

/codon_start=1

/transl_table=11

/product="ribosomal protein L36"

/translation="MKIRASVRKICEKCRLIRRRGRIRVICSNPRHKQRQG"

gene complement(104267..104500)

/gene="infA"

CDS complement(104267..104500)

/gene="infA"

/codon_start=1

/transl_table=11

/product="translational initiation factor 1"

/translation="MKEQKWIHEGLITESLPNGMFRVRLDNEDLIIGYVSGKIRRSFI

RILPGDKVKIEVSRYDSTRGRIIYRLRNKDSKD"

gene complement(104623..105036)

/gene="rps8"

CDS complement(104623..105036)

/gene="rps8"

/codon_start=1

/transl_table=11

/product="ribosomal protein S8"

/translation="MGRDTIAEIITSIRNADMDRKRVVRIASTNITENIVKILFREGF

LENVRKHREKNQNFLVLTLRHNRRNRKRPHTCRNFLNLKRISRPGLRIYSNSQRIPRI

LGGMGIVILSTSRGIMTDREARLERIGGEILCYIW"

gene complement(105209..105577)

/gene="rpl14"

CDS complement(105209..105577)

/gene="rpl14"

/codon_start=1

/transl_table=11

/product="ribosomal protein L14"

/translation="MIQPQTHLNVADNSGARKLMCIRILGASNRRYAHIGDIIVAVIK

EAVPNMPLEKSEVVRAVIVRTCKELKRDSGMIIRYDDNAAVVIDQEGNPKGTRIFGAI

PRELRQFNFTKIVSLAPEVL"

gene complement(105704..107007)

/gene="rpl16"

CDS complement(join(105704..106102,106999..107007))

/gene="rpl16"

/codon_start=1

/transl_table=11

/product="ribosomal protein L16"

/translation="MLSPKRTRFRKQHRGRMKGISYRGNHICFGKYALQALEPAWITS

RQIEAGRRAMTRNARRGGKIWVRIFPDKPVTIRPAETRMGSGKGSPEYWVAVVKPGRI

LYEMGGVTENIARRAILIAASKMPIRTQFIISG"

exon complement(105704..106102)

/gene="rpl16"

/number=2

intron complement(106103..106998)

/gene="rpl16"

/number=1

exon complement(106999..107007)

/gene="rpl16"

/number=1

gene complement(107163..107825)

/gene="rps3"

CDS complement(107163..107825)

/gene="rps3"

/codon_start=1

/transl_table=11

/product="ribosomal protein S3"

/translation="MGQKINPLGFRLGTTQSHHSLWFAQPKNYSEGLQEDQKIRDFIT

NYVQKNMRISSGAEGIARIEIQKRIDLIQIIIFMGFPKLLIESRPRGIEELQMNLQNE

FHYVNRKLNIAITRIAKPYGNPNILAEFIAGQLKNRVSFRKAMKKAIELTEQADTKGI

QIQIAGRIDGKEIARVEWIREGRVPLQTIRAKIDYCSYTVRTIYGVLGIKIWIFIDKG

EE"

gene complement(107810..108274)

/gene="rpl22"

CDS complement(107810..108274)

/gene="rpl22"

/codon_start=1

/transl_table=11

/product="ribosomal protein L22"

/translation="MLRKKKTEVYALGRHISLSADKARRIIDQIRGCSYEETLMILEL

MPYKACYPIFKLVYSAAANASLIMGSDEANLVIRKAEVNEGPSAKKLKPRARGRSYAI

KKATCHITIVVKDISLPEYEEMYFYSLKNPRWQKTTMVYDDAYNSEVVWDKK"

gene complement(108334..108612)

/gene="rps19"

CDS complement(108334..108612)

/gene="rps19"

/codon_start=1

/transl_table=11

/product="ribosomal protein S19"

/translation="MIRSLKKNPFVANHLLRKIDKLNKKAEKEIIVTWSRASTIIPTM

IGHTIAVHNGKEHLPIYITDRMVGHKLGEFVPTLNFQGHAKSDNRSRR"

repeat_region 108571..134221

/note="inverted repeat B (IRB)"

/rpt_type=inverted

gene complement(108671..110162)

/gene="rpl2"

CDS complement(join(108671..109104,109772..110162))

/gene="rpl2"

/codon_start=1

/transl_table=11

/product="ribosomal protein L2"

/translation="MAIHLYKTSTPSTRNGTVDSQVKSNPRNNLIYGQHHCGKGRNAR

GIITSRHRGGGHKRLYRKIDFRRNEKDIYGRIVTIEYDPNRNAYICLIHYGDGEKRYI

LHPRGAIIGDTIVSGTEVPIKMGNALPLTDMPLGTAIHNIEITLGKGGQLVRAAGAVA

KLIAKEGKSATLKLPSGEVRLISKNCSATVGQVGNVGANQKSLGRAGSKRWLGKRPVV

RGVVMNPVDHPHGGGEGRAPIGRKKPTTPWGYPALGRRSRKRNKYSENLIVRRRSK"

exon complement(108671..109104)

/gene="rpl2"

/number=2

intron complement(109105..109771)

/gene="rpl2"

/number=1

exon complement(109772..110162)

/gene="rpl2"

/number=1

gene complement(110181..110462)

/gene="rpl23"

CDS complement(110181..110462)

/gene="rpl23"

/codon_start=1

/transl_table=11

/product="ribosomal protein L23"

/translation="MDGIKHAVFTDKSIRLLGKNQYTSNVESGSTRTELKHWVELFFG

VRVIAMNSHRLPGKGRRMGPIMGHTMHYRRMIITLQPGYSIPPLRKKRT"

gene complement(110627..110701)

/gene="trnM-CAU"

tRNA complement(110627..110701)

/gene="trnM-CAU"

/product="tRNA-Met"

gene complement(110628..110701)

/gene="trnI-CAU"

tRNA complement(110628..110701)

/gene="trnI-CAU"

/product="tRNA-Ile"

gene 110790..117629

/gene="ycf2"

CDS 110790..117629

/gene="ycf2"

/codon_start=1

/transl_table=11

/product="Ycf2 protein"

/translation="MKGHQFQSWIFELREILREIKNSHCFLDSWTQFNSVGSFIRIFF

HQERFLKLFDPRILSILLSRNSQGSTSNRYFTIKGVLLFVVAVLIYRINNRNMVERKN

LYLRGLLPIPMNSIGPRNDTLEESVGSSNINRLIVSLLYLPKGKKISESCFLNPKEST

WVLPITKKCSMPESNWGSRWWRNWIGKKRDSSCKISNETVTGIGILFKEKDLKYLEFL

FVYYMDDPIRKDHDWELFDRLSLRKRRNRINLNSGPLFEILVKHWISYLMSAFREKIP

IEVEGFFKQQRAGSTIQSNDIEHVSHLLSRNKRAISLQNCAQFHMWQFRQDLFVSWGK

NPHESDFLRNVSRENWIWLDNVWLVNKDRFFRKVRNVSSNIQYDSTRSSFVQVTDSSQ

LKGSSDQSRDHLDSISNEDSEYHTLINQREIQPLKERSILWDPSFLQTEGTEIESDRF

PKCLSGYSSMSRLFTEREKQMINHLLPEEIQEFLGNPTRSVRSFFSDRWSELHLGSNP

TERSTRDPKLLKKQQDLSFVPPRRSENKELVNIFKIITYLQNTVSIHPISSDPGCDRV

LKDDPDMDSSNKISFLNKNPFFDLFHLFHDRNRGGYTLHHDFESEERFQEMADLFTLS

ITEPDLVYHKGFPFSIDSYGLDQKQFLNEARDESKKKSLLVLPPIFYEENESFSRRIR

KKWVRISCGNDLEDPKPKIVVFASNNIMEAVNQYRLIRNLIQIQYSTYGYIRNVLNRL

FLMNRSDRNFEYGIQRDQIGKDTLNHRTIMKYTINPHLSNLKKSQKKWLDPLILISRT

ERSMNRAPDAYRYKWSNGSKNFQEHLEHFVSEQKSRFQIVFDRLRINQYSIDWSEVID

KKDLSKPLRFFLSKSLLFLSKLLFFLSNSLPFFCVSFGNIPIHRSEIYIYELKGPNDQ

LCNQLLESIGLQIVHLKKWKPFLLDEHDTSRKSKFLINGGTPFLFNKIPKWMIDSFHT

RNNRRKSFDNADSYFSMIFHNQDNWLNPVKPFHISSLISSFYKANRLRFLNNPHHFCF

YCNTRFPFSVEKARINNYDFTYGQFLNILFIRNKIFSLCVGKKKHAFGGRDTISPIES

QVSNIFIPNDFPQSGDETYNLYKSFHFPSQHDPFVRRTIYSIADMFGTPLTEGQIVHF

ERTYCQPLSDMNLSDSEGKNLHQYLNSNVGLIHTPCSGKYLPSEKRKRWSLCLKKCVE

KGQMYRTFQRDGAFSTLSKWNLFQTYIPWFLTSTGYKYLNLIFLDTFSDLLPILSSSQ

KFVSIFHDIMHGSGIAWRILQKKWCLPQWNLISEISSKCFHNLLLSEEMIHRNNESPS

THLRSPNVREFLYSILFLLLVAGYLVRTHLLFVSRASSELQTEFEKVKSLMIPSSMIE

LRKLLDRYPTSAPNSFWLKNLFLVALEQLGDSLEEIRASGGNMPGPAYGVKSIRSKKK

YLSINLIDLIPNPINQITFSRNTRHLSHTSKEIYSLIRKRKNVNGDWIDDKIESWVAN

SDSIDDEEREFLVQFSALTTEKRIDQILLSLTHSDHLSKNDSGYQMIEQPGAIYLRYL

VDIHKKYLLNYEFNTSSLAERRVFLAHYQTITYSQTSCGTNTLHFPSHGKPFSLRLAL

SPSRGILVIGSIGTGRSYLVKYLATNSYVPFITVFLNKFLDNKPKGFIFDDIDIDASD

DIDASDDIDASDDIDRDLDTELELLTMDRMPEIDRFYITLQFELAKAISPCIIWIPNI

HDLDVNESNYLSLGLLVNHLSERCSTRNILVIASTHIPQKVDPALIAPNKLNTCIKIR

RLLIPQQRKHFFTLSYTKGFHLEKKMFHTNGFGSITMGSNARDLVALTNEALSISITQ

KKSIIDTKTIRSALHRQTWDLRSQVRSVQDHGILFYQIGRAVAQNVLLSNCPIDPISI

YMKKKSCNEGDSYLYKWYFELGTSMKKLTILLYLLSCSAGSVAQDLWSLPGSDEKNGI

TSYGLVENDSDLVHGLLEVEGALVGSSRTEKDCSPFDNDRVTLLLRPELRNPLDMMQN

GSCSIFDQRFLYEKYESEFEEGKGEAALDPQQIEEDLFNHIVWAPRIWRPWAFLFDCI

ERPNELGFPYWSRSFRGKRIIYDEEDELQENDSEFLQSGTMQYQTRDRSSKEQGLFQI

SQFIWDPADPLFFLFKDQPPGSVFSHRELFADEEMSKGLLTSQTDPPTSIYKRWFIKN

TQEKHFELLINRQRWLRTNSSLSNGSFRSNTLSESYQYLSTLFLSNGTLLDQMTKTLV

RKRWLFPDEMKIGFMEQEKDFPFLSRKDMWL"

gene complement(118175..118257)

/gene="trnL-CAA"

tRNA complement(118175..118257)

/gene="trnL-CAA"

/product="tRNA-Leu"

gene complement(118176..118256)

/gene="trnL-CAA"

tRNA complement(118176..118256)

/gene="trnL-CAA"

/product="tRNA-Leu"

gene complement(118801..121007)

/gene="ndhB"

CDS complement(join(118801..119556,120231..121007))

/gene="ndhB"

/codon_start=1

/transl_table=11

/product="NADH dehydrogenase subunit B"

/translation="MIWHVQNENFILDSTRIFMKAFHLLLFDGSLIFPECILIFGLIL

LLMIDSTSDQKDIPWLYFISSTSLVMSITALLFRWREEPIISFSGNFQTNNFNEIFQF

LILLCSTLCIPLSVEYIECTEMAITEFLLFVLTATLGGMFLCGANDLITIFVAPECFS

LCSYLLSGYTKKDVRSNEATMKYLLMGGASSSILVHGFSWLYGLSGGETELQEIVSGL

INTQMYNSPGISIALIFITVGIGFKLSPAPSHQWTPDVYEGSPTPVVAFLSVTSKVAA

SASATRILDIPFYFSSNEWHLLLEILAILSMILGNIIAITQTSMKRMLAYSSIGQIGY

VIIGIIVGDSNDGYASMITYMLFYISMNLGTFACIVLFGLRTGTDNIRDYAGLYTKDP

FLALSLALCLLSLGGLPPLAGFFGKLYLFWCGWQAGLYSLVLIGLLTSVVSIYYYLKI

IKLLMTGRNQEITPHVRNYRRSPLRSNNSIELSMIVCVIASTIPGISMNPIIAIAQDT

LF"

exon complement(118801..119556)

/gene="ndhB"

/number=2

intron complement(119557..120230)

/gene="ndhB"

/number=1

exon complement(120231..121007)

/gene="ndhB"

/number=1

gene complement(121271..121738)

/gene="rps7"

CDS complement(121271..121738)

/gene="rps7"

/codon_start=1

/transl_table=11

/product="ribosomal protein S7"

/translation="MSRRGTAEEKTAKSDPIYRNRLVNMLVNRILKHGKKSLAYQIIY

RAMKKIQQKTETNPLSVLRQAIRGVTPDIAVKARRVGGSTHQVPIEIGSTQGKALAIR

WLLAASRKRPGRNMAFKLSSELVDAAKGSGDAIRKKEETHKMAEANRAFAHFR"

exon complement(121792..121817)

/gene="rps12"

/number=3

intron complement(121817..122352)

/gene="rps12"

/number=1

intron complement(121818..122353)

/gene="rps12"

/number=1

exon complement(122354..122585)

/gene="rps12"

/number=2

gene 124201..124272

/gene="trnV-GAC"

tRNA 124201..124272

/gene="trnV-GAC"

/product="tRNA-Val"

gene 124495..125985

/gene="rrn16"

rRNA 124495..125985

/gene="rrn16"

/product="16S ribosomal RNA"

gene 126305..127313

/gene="trnI-GAU | trnE-UUC"

tRNA join(126305..126346,127279..127313)

/gene="trnI-GAU"

/product="tRNA-Ile"

tRNA join(126305..126341,127279..127313)

/gene="trnI-GAU"

/product="tRNA-Ile"

tRNA join(126305..126336,127274..127313)

/gene="trnE-UUC"

/product="tRNA-Glu"

exon 126305..126346

/gene="trnI-GAU"

/number=1

exon 126305..126341

/gene="trnI-GAU"

/number=1

exon 126305..126336

/gene="trnE-UUC"

/number=1

intron 126337..127273

/gene="trnE-UUC"

/number=1

intron 126342..127278

/gene="trnI-GAU"

/number=1

intron 126347..127278

/gene="trnI-GAU"

/number=1

exon 127274..127313

/gene="trnE-UUC"

/number=2

exon 127279..127313

/gene="trnI-GAU"

/number=2

gene 127378..128256

/gene="trnA-UGC"

tRNA join(127378..127415,128220..128256)

/gene="trnA-UGC"

/product="tRNA-Ala"

tRNA join(127378..127415,128222..128256)

/gene="trnA-UGC"

/product="tRNA-Ala"

tRNA join(127378..127414,128221..128256)

/gene="trnA-UGC"

/product="tRNA-Ala"

exon 127378..127415

/gene="trnA-UGC"

/number=1

exon 127378..127414

/gene="trnA-UGC"

/number=1

intron 127415..128220

/gene="trnA-UGC"

/number=1

intron 127416..128221

/gene="trnA-UGC"

/number=1

intron 127416..128219

/gene="trnA-UGC"

/number=1

exon 128220..128256

/gene="trnA-UGC"

/number=2

exon 128221..128256

/gene="trnA-UGC"

/number=2

exon 128222..128256

/gene="trnA-UGC"

/number=2

gene 128453..131263

/gene="rrn23"

rRNA 128453..131263

/gene="rrn23"

/product="23S ribosomal RNA"

gene 128453..128764

/gene="rrn23-fragment"

rRNA 128453..128764

/gene="rrn23-fragment"

gene 129011..131263

/gene="rrn23"

rRNA 129011..131263

/gene="rrn23"

/product="23S ribosomal RNA"

gene 131362..131464

/gene="rrn4.5"

rRNA 131362..131464

/gene="rrn4.5"

/product="4.5S ribosomal RNA"

gene 131720..131840

/gene="rrn5"

rRNA 131720..131840

/gene="rrn5"

/product="5S ribosomal RNA"

gene 132075..132149

/gene="trnR-ACG"

tRNA 132075..132149

/gene="trnR-ACG"

/product="tRNA-Arg"

gene 132075..132148

/gene="trnR-ACG"

tRNA 132075..132148

/gene="trnR-ACG"

/product="tRNA-Arg"

gene complement(132720..132793)

/gene="trnN-GUU"

tRNA complement(132720..132793)

/gene="trnN-GUU"

/product="tRNA-Asn"

gene complement(132721..132792)

/gene="trnN-GUU"

tRNA complement(132721..132792)

/gene="trnN-GUU"

/product="tRNA-Asn"

CDS 133118..134236

/gene="ycf1"

/codon_start=1

/transl_table=11

/product="Ycf1 protein"

/translation="MIFQSFLLGNLVSLCMKIINSVVVVGLYYGFLTTFSIGPSYLFL

LRAQVMEEGTEKKVSATTGFITGQLMMFISIYYAPLHLALGRPHTITVLALPYLLFHF

FWNNHKHFFDYGSTTRNSMRNFSIQCLFLNNLIFQLFNHFILPSSMLARLVNIYMFRC

NNKMLFVTSSFVGWLIGHILFMKWLGLVLVWIRQNHSIRSNVLIRSNKYLVSELINSK

ARILSILLFITCVYYLGRIPSPLFTKKLKETSKTGAGERVESAEERDVEIETASEMKG

TKQEQEGSTEEDPSPYFFSEERADPNKIDETEEIQVNGKEKEFHFRFTETDYQNRPVS

EESYLMNINENQDNSRLKIVDQKTENKELIKKIDTKEK"

gene complement(134182..136404)

/gene="ndhF"

CDS complement(134182..136404)

/gene="ndhF"

/codon_start=1

/transl_table=11

/product="NADH dehydrogenase subunit F"

/translation="MEQTYQYACILPFVPLLVPILIGVGLVIFPTATKNLHRMWAFPS

ILLLSIVMIFSTNLSIQQINSSYIYQYVWSWTLDNDFSLEFGCLIDPLTSIMLMLITT

VGIMVLIYSDNYMAHDQGYLRFFAYMSFFSTSMLGLVTSSNLIQIYIFWELVGMCSYL

LIGFWFTRPPAANACQKAFVTNRVGDFGLLLGILGFYWITGSFEFRDLFEILNNLISN

NEVNFPFVILCAALLFAGAVAKSAQFPLHVWLPDAMEGPTPISALIHAATMVAAGIFL

VARLLPLFVVIPYIMNLIALIGIITLLLGATLALAQKDIKRSLAYSTMSQLGYMMFAL

GIGSYRSALFHLITHAYSKALLFLGSGSVIHSMETLVGYSPDKSQNMVLMGGLTKHVP

ITKTSFLLGTLSLCGIPPLACFWSKDEILNDSWLYSPIFAIIAWATAGLTAFYMFRIY

LLTFEGHLNINFQNYSGNQNNSFYSISIWGKGYSKRINQNFALLRNESSYFFWKKTCR

SDENARKKRGGRPFINILHFDNQKAFSYPSESANTMLFSLLLLVLFTLVVGSLGISFN

QKRTDLDLLSKWLAPSINLLHQKSKDSASWYEFFKDSLLSVGIAYCGIFLASFLYKPI

YSSLKNFDLINSFVKLGPKRKRLDKIINALYDWSYNRAYIDSFYTIFFARGVRELAQL

TNFFDRRIIDGITNGVGVMSFFLGEGIKYLGGGRISSYLFFYFSFVSIFLISSLFSVF

"

misc_feature 134222..151589

/note="small single copy (SSC)"

gene 136923..137099

/gene="rpl32"

CDS 136923..137099

/gene="rpl32"

/codon_start=1

/transl_table=11

/product="ribosomal protein L32"

/translation="MAVPKKRTSASKKRIRKNFWKKKGYRAALKAFSLGKSLSTGNSK

SFFVRQTNKLSNKT"

gene 137577..137656

/gene="trnL-UAG"

tRNA 137577..137656

/gene="trnL-UAG"

/product="tRNA-Leu"

gene 137741..138712

/gene="ccsA"

CDS 137741..138712

/gene="ccsA"

/codon_start=1

/transl_table=11

/product="cytochrome c biogenesis protein"

/translation="MIFSILEHILTHISFSIVSIIITIHLITFLVDEIVKLDDSSKTG

MIMTFFCITGLLISRWIYSRHFPLSDLYESLIFLSWSFSFIYIVSYFKKNQNILSTII

GPSALFSQGFATSGLLTEIHESTILVPALQSEWLIMHVSMMILGYAALLCGSLLSVSL

LVITVRKKLSLFSTSNHLLNLNESFFLGEIEYMNEQRDFLQKTSFFFARNYYRSQLIQ

KLDYWSYRVISLGFIFLTIGILSGAVWANEAWGSYWSWDPKETWAFITWIIFSIYLHT

RTNIKRKSINSAIVASIGFLIIWICYFGVNLLGIGLHSYGSFTLTSN"

gene complement(140515..140760)

/gene="psaC"

CDS complement(140515..140760)

/gene="psaC"

/codon_start=1

/transl_table=11

/product="photosystem I subunit C"

/translation="MSHSVKIYDTCIGCTQCVRACPTDVLEMIPWDGCKAKQIASAPR

TEDCVGCKRCESACPTDFLSVRVYLWHETTRSMGLAY"

gene complement(141042..141347)

/gene="ndhE"

CDS complement(141042..141347)

/gene="ndhE"

/codon_start=1

/transl_table=11

/product="NADH dehydrogenase subunit E"

/translation="MMLEHILVLSAYLFSIGIYGLITSRNMVRALMCLELILNSVNIN

FVTFSDIFDNRQLRGDIFSIFVIAIAAAEAAIGSAIVSSIYRNRKSIRINQSNLLNK"

gene complement(141563..142093)

/gene="ndhG"

CDS complement(141563..142093)

/gene="ndhG"

/codon_start=1

/transl_table=11

/product="NADH dehydrogenase subunit G"

/translation="MDLPGPIHDFLLVFLGLGLILGSLAVVLLPNPIYSAFSLGWVLF

CISLFYILSNSYFVAAAQLLIYVGAINVLIIFAVMFMNGSEYYKDFNLWTVGDGVTSI

VCTSLFISLITTIPDTSWYGIVWTTKSNQILEQDLISNSQQIGIHLATDFFLPFELIS

IILLVALIGAIAIARQ"

gene complement(142483..142989)

/gene="ndhI"

CDS complement(142483..142989)

/gene="ndhI"

/codon_start=1

/transl_table=11

/product="NADH dehydrogenase subunit I"

/translation="MFPMLTQFLNSGQQTIRAARYIGQGFMITLSHANRLPVTIQYPY

EKLITSERFRGRIHFEFDKCIACEVCVRVCPIDLPVVDWKLQTDIRKKRLLNYSIDFG

ICIFCGNCVEYCPTNCLSMTEEYELSAYDRHELNYNQIALGRLPVSIIEDYTIRTISS

NLPQIKNV"

gene complement(143076..145235)

/gene="ndhA"

CDS complement(join(143076..143614,144683..145235))

/gene="ndhA"

/codon_start=1

/transl_table=11

/product="NADH dehydrogenase subunit A"

/translation="MIIDTTKIQAINSFFRLGSLKEVYGIIWMLIPILILVLGITLGV

LVIVWLEREISAGIQQRIGPEYAGPFGILQALADGTKLLFKENILPSRGDTRLFSLGP

SIAVISILLSYSVIPFSYRFILADLSIGVFLWIAVSSLAPVGLLMSGYGSNNKYSFLG

GLRAAAQSISYEIPLTLCVLSISLLSNSLSTVDIVEVQSKYGFWGWNLWRQPIGFIVF

LISSLAECERLPFDLPEAEEELVAGYQTEYSGIKFGLFYVASYLNLLVSSLFVTVLYL

GGWNLSIPYLFFPELFDINKRSQVFGTIIGIFITLAKTYLFLFISIATRWTLPRLRMD

QLLNLGWKFLLPISLGNLLLTTSSQLLSL"

exon complement(143076..143614)

/gene="ndhA"

/number=2

intron complement(143615..144682)

/gene="ndhA"

/number=1

exon complement(144683..145235)

/gene="ndhA"

/number=1

gene complement(145237..146418)

/gene="ndhH"

CDS complement(145237..146418)

/gene="ndhH"

/codon_start=1

/transl_table=11

/product="NADH dehydrogenase subunit H"

/translation="MIPPTTRKDLMIVNMGPHHPSMHGVLRLIVTLDGEDVINCEPVL

GYLHRGMEKIAENRTIIQYLPYVTRWDYLATMFTEAITVNGPEQLGNIQVPKRASYIR

AIMLELSRIASHLLWLGPFMADIGAQTPFFYIFRERELIYDLFEAATGMRMMHNFFRI

GGVAADLPHGWIDKCLDFCDYFLTGVAEYQKLITRNPIFLERVEGVGIIGGEEALNWG

LSGPTLRASGIQWDLRKVDRYECYEEFDWEIQWQKEGDSLARYLVRIGEMTESIKIIQ

QALEGIPGGPYENLESRRFDRIKDPEWNDFEYRFISKKPSPTFELSKQELYVRVEAPK

GELGIFLIGDRSVFPWRWKIRPPGFINLQILPQLVKRMKLADIMTILGSIDIIMGEVD

R"

gene complement(146519..146791)

/gene="rps15"

CDS complement(146519..146791)

/gene="rps15"

/codon_start=1

/transl_table=11

/product="ribosomal protein S15"

/translation="MVKNSSISVIFQNENKETRGSVEFQVFSFTTKIRKLTSHLELHK

KDFSSQRGLRKILGKRQRLLAYLSKINRGRYKELIGELDIREIKTR"

gene complement(147224..149620)

/gene="trnI-AAU"

tRNA complement(join(147224..147284,149587..149620))

/gene="trnI-AAU"

exon complement(147224..147284)

/gene="trnI-AAU"

/number=2

intron complement(147285..149586)

/gene="trnI-AAU"

/number=1

exon complement(149587..149620)

/gene="trnI-AAU"

/number=1

ORIGIN

1 tcaatcttga attatcctga ttttcattga tattcattag ataagactct tcagaaacgg

61 gtctattttg atagtctgtc tctgtaaatc taaagtggaa ttctttttcc tttccattca

121 cttggatttc ttccgtttca tcgattttgt tcggatccgc cctttcttcc gaaaaaaagt

181 aaggagaagg atcttcttcg gtggatccct cttgttcctg tttagtcccc ttcatttcgg

241 aagctgtttc tatttctaca tctctttctt ccgcactttc caccctttct cccgctcccg

301 tttttgaggt ttctttcagt ttcttagtaa aaaggggtga cggtattctg cctaaatagt

361 agacacaggt aataaataag agaatactca agatccgagc cttagaattt atcaattctg

421 atacaaggta cttattagat cgaataagta cattcgatct aatagaatga ttttgccgta

481 tccagactaa taccaatcca agccatttca tgaataaaat gtgaccaatt aaccaaccaa

541 caaaactact tgttacaaat aacatcttgt tgttgcatcg aaacatataa atgttgacta

601 atctggctaa cattgaactt ggtaaaatga aatggttgaa taattgaaaa atgagattat

661 ttagaaataa acattgaatg ctgaaattac gcattgaatt tctggtagta gatccataat

721 caaaaaagtg tttgtgattg ttccagaaga aatgaaacaa aagatatggt agagctagga

781 cagttattgt atgaggtcta cccaatgcta gatgcagagg cgcataatag atcgatatga

841 acatcatgag ctgtcccgta ataaaaccag ttgttgctga taccttcttc tcggttcctt

901 cttccataac ctgagctcgg agaaggaaga gataagaggg ccctatagag aatgtggtca

961 gaaatccata atagagtccg accacaacga ccgaattgat tatcttcatg cataaggata

1021 ctagattacc tagtagaaaa gattgaaaaa tcatcacaaa cctccccctt ttcttttcta

1081 ttgcaacttc tggattatta tatgatgatt tttgaacttt ccatatatag aaaagaaata

1141 gaaagagata aactagaaat tatgtcaatg acaccaaagg gggtattaaa tgaatggaat

1201 tgggatatgg atggaatata atataatgaa atagagccgc tttgaggttc cctccgaaat

1261 gaggcatgga agggagccac tacgaagaag ttccgggagt tacgaaggag gattcgagct

1321 catattgttc atgggttgag aacgggaatt gaactctatg agatctaatc tcccgttgtt

1381 cctcagtagc tcagtggtag agcggtcggc tgttaaccga ttggtcgtag gttcgaatcc

1441 tacttgggga gatttgattc attctgaatt aacgaattca gaatagaata aaggggctca

1501 ctttgaccgt taagagtagg gaacctgttc cctgtctttg tttctatctc atcgtatacc

1561 attccgttct gcgagatttg aaaataaccg tcaatacctc ggtgtagtcc ccggataatc

1621 cacagccctg gggctattta caactagcca attaagaatt ctcagatgta ttagcactgc

1681 atcaaaatca aagatgcagt catcgattct cccgagagtt cacaattgcc gcgagcaaac

1741 atattaatga cgaggaagtt tgttatgcta ctaatacttg tacttgctcg cctattctgc

1801 ccaagcctgg ctgaggaaga gttacagggc gtaaaacaaa aaaatacgct gatggcgggg

1861 gggcatacta tgtgtaatga ttccatcatt tacgataaat aagaaataaa aagaaaaagc

1921 cattccaaag acccacaccc aagttccata gcttttggtc cgctatcccg atcatgattt

1981 tcctaccccc ggggggaaag gtacttccct tttgggccgg ttgtgggcga ggagggattc

2041 gaacccccga caccgtggtt cgtagccacg tgctctaatc ctctgagcta caggccccac

2101 cccgtctcca ctggatctgt tcccgggagt accctcaaaa aaaggaacct ttcctctccc

2161 cagccatttc gggttaagaa gatgtgaaag cgcgtttatc tcggtacgtt ccgaggtgtg

2221 aagtgggaga gaagggatgt cataattggg gttttgaata agacgacctt tttatttttc

2281 attttattcg ttgaaaaagt aataagaatg agaggtgtta agctttttat catcctggcg

2341 tcgagctatt tttccgcagg acctccccta cagtatcgtc accgcagtag agtttaacca

2401 ccaagttcgg gatggattgg tgtggttcct ctacgcctag gacaccagaa tatcgaacca

2461 tgaacgaaga agggcatgag agaaaagcct attggctagt gattgtgagg ccccaattct

2521 tgactggaag ggacaccaaa ggcctctgcc cttccatccc ttggatggat agaaggggag

2581 ggcggggctt ttggtttttt catgttgtca aagagttgaa caatggtttt tcgtgttgtc

2641 aaagagttga acaatgaaaa tagatggcga gtgcctgatc gaattgatca ggtcatgcag

2701 gaacaaggtt caagtctacc ggtctgttag gatgcctcag ctgcatacat cactgcactt

2761 ccacttgaca cctatcgtaa tgataaacgg ctcgtctcgc cgtgaccttc tcttgaattc

2821 tcaaaacttc tgtcgctcca tccccgcagg ggcagagaac ccgtcgttgt ctcggctgtg

2881 ctgccggaag ctctggggaa gtcggaatag gagagcactc atcttggggt gggcttacta

2941 cttagatgct ttcagcagtt atccgctccg cacttggcta cccagcgttt accgtgggca

3001 cgataactgg tacaccagag gtgcgtcctt cccggtcctc tcgtactagg gaaaggtcct

3061 ctcaatgctc taacgcccac accggatatg gaccgaactg tctcacgacg ttctgaaccc

3121 agctcacgta ccgctttaat gggcgaacag cccaaccctt ggaacatact acagccccag

3181 gtggcgaaga gccgacatcg aggtgccaaa ccttcccgtc gatgtgagct cttggggaag

3241 atcagcctgt tatccctaga gtaactttta tccgttgagc gacggccctt ccactcggca

3301 ccgtcggatc actaaggccg actttcgtcc ctgctcgacg ggtgggtctt gcagtcaagc

3361 tcccttctgc ctttgcactc gagggccaat ctccgtccgg cccgaggaaa cctttgcacg

3421 cctccgttac cttttgggag gcctacgccc catagaaact gtctacctga gactgtccct

3481 tggcccgtag gtcctgacac aaggttagaa ttctagctct tccagagtgg tatctcactg

3541 atggctcggg cccccccgga aggaggcctt cttcgccttc cacctaagct gcgcaggaaa

3601 ggcccaaacc caatcccagg gaacagtaaa gcttcatagg gtctttctgt ccaggtgcag

3661 gtagtccgca tcttcacaga catgtctatt tcaccgagcc tctctccgag acagtgccca

3721 gatcgttacg cctttcgtgc gggtcggaac ttacccgaca aggaatttcg ctaccttagg

3781 accgttatag ttacggccgc cgttcaccgg ggcttcggtc gccggctccc ctgtcatcag

3841 gtcaccaact tccttgacct tccggcactg ggcaggcgtc agcccccata catggtctta

3901 cgactttgcg gagacctgtg tttttggtaa acagtcgccc gggcctggtc actgcgaccc

3961 cctttgtgag gaggcacccc ttctcccgaa gttacggggc tattttgccg agttccttag

4021 agagagttgt ctcgcgcccc taggtattct ctacctaccc acctgtgtcg gtttcgggta

4081 caggtaccct tttgtttaaa ggtcgttcga gcttttcctg ggagtatggc gtgggttact

4141 tcagcgccgt agcgcctggt gctcgaacat cggctcgagg cattttctct accccttctt

4201 accctgaaaa agcagggaca ccttacgttc ttgaaccgat aaccatcttt cggctaacct

4261 agcctgctcc gtccctcggg accaacaagg ggtagtacag gaatattcac ctgttgtcca

4321 tcgactacgc ctttcggcct gatcttaggc cctgactcac cctccgtgga cgaaccttgc

4381 ggaggaaccc ttaggttttc ggggcattgg attctcacca atgtttgcgt tactcaagcc

4441 gacattctcg cttccgcttc gtccaccgcc gctcgcgcgg ccgcttcccc ctaaggcgga

4501 acgctcccct accgatgtat ttttacatcc cacagcttcg gcagatcgct tagccccgtt

4561 catcttcggc gcaagagcgc tcgatcagtg agctattacg cactctttca agggtggctg

4621 cttctaggca aacctcctgg ctgtctctgc acccctacct cctttatcac tgagcgatca

4681 tttaggggcc ttagctggtg atccgggctg tttccctctc gacgatgaag cttatccccc

4741 atcgtctcac tggtcgactt tgacccctgt tattttgagg tcatatctag tattcagagt

4801 ttgcctcgat ttggtaccgc tctcgcggcc cgcaccgaaa cagtgcttta cccctagatg

4861 tccagtcaac tgctgcgcct caacgcattt cggggagaac cagctagctc tgggttcgag

4921 tggcatttca cccctaacca caactcatcc gctgattctt caacatcagt cggttcggac

4981 ctccacttag tttcacccaa gcttcatcct ggtcatggat agatcaccca ggttcgggtc

5041 cataagcagt gaccattgcc ctatgaagac tcgctttcgc tgtggctccg gtgggttccc

5101 ttaaccaagc cactgcctat gagtcgccgg ctcattcttc aacaggcacg cggtcagagc

5161 catggctcct cccactgctt gggagcttac ggtttcatgt tctatttcac tccccgatgg

5221 gggttctttt cacccttccc tcacggtact acttcgctat cggtcaccca ggagtattta

5281 gccttgcaag gtggtccttg ctgattcaca cgggattcca cgtgtcccat gctactcggg

5341 tcagagcgta agctagtgat gctttcggct actggacttt cgccatctag ggtgcagcat

5401 ttatgctgct tcgcctagca gcacgacgct tgtattgctc tcccacaacc ccggtttcac

5461 ggtttaggct gctcccattt cgctcgccgc tactacggga atcgcttttg ctttcttttc

5521 ctctggctac taagatgttt cagttcgcca ggttgtctct tgcctgccca tggattcagc

5581 agcagtttga aaggttgccc tattcgggaa tctccggatc tatgcttatt ttcaactccc

5641 cgaagcattt cgtcgattac tacgcccttc ctcgtctctg ggtgcctagg tatccaccgt

5701 aagcctttcc tcgtttgaac ctcgccctta ttgtattgcc cttataaaaa tcttataaat

5761 tagaaaataa attataaata aggctatgcc atcctaaggt gctgctaaat ggatggatct

5821 tatcaacgtc catgaatgat aaatcataga tcgaaccgcc gaatcggaaa aattgggtgc

5881 tatcatatag ctttgtatcg gctaagttca tgagttggag ataagcggac tcgaaccgct

5941 gacatccgcc gcagggtaaa ccaccgtctc tcatgccccc gactgattct accatagagg

6001 ccaacgatag acaataaccc cctccgaaca cagcttacaa ctttcatcgt actgtgctct

6061 ccaaagagca actcttctca aaatctcact caaagggtgc tgagttggaa tcccattcta

6121 actcaggatt cttgcggttc cggaggttcc agctacagga gaaccaggaa cggagagccc

6181 ccctttttcc gcccgactct ttggtcttaa gaacgctggt tttaagaatg agtgattgcc

6241 cttctccgac ccttactgcc caacctgaga acggacagct aatgcgttcc acttattgaa

6301 cagggttcta tggtcggtcc gtgacccctg gatgccgaag gcgcccttgg ggtgatctcg

6361 tagttcctac ggggtggaga tgatggggtc ggtccatgga ttttccttcc ttttcttttg

6421 ccgcatttcg ctcaaagggt tgaagggaga tagtgcatca agctgttcgc aagggccaac

6481 ttgatcctct tccccaggga tctcagatga gggaccctgg gggagccgcc gactccaacg

6541 accgtccatg tatgatccat actagatctg accaactgcc catcctacct cctctacgtt

6601 cttgacagcc catcttcgtc tcagtagagt ctttcagtgg catgtttcgg tcctcttccc

6661 cattacttag aaaaagtgag ccaccggttc aggtacaaga tactatcatt actgcctgga

6721 caattagaca tccaacccgt aatcgcaacg acccaattgc aagagcggag ctctaccaac

6781 tgagctatat ccccccgagc caagtggagc atgcatgaag tagtcagatg cttcttctat

6841 tcttttccct ggcgcagctg ggccatcctg gacttgaacc agagacctcg cccgtgaagt

6901 aaatcatcgc acctacggtc caaccaattg ggagagaatc aatagattcc ttttcggtag

6961 cgattcatcc ttcccgaacg cagcatacaa ctctccgttg tactgcgctc tccaagtgtg

7021 cttgttcccc ccttcttcct taccccggca agtctttgtg aaataactct gatgagaaga

7081 aaaaagaagg cgttaagaga ccctcccggc ccaaccctag acactctaag atcctttttc

7141 aaacctgctc ccatttcgaa tcaagagata gataaataga cacatcccat tgcactgatc

7201 gggggcgttc gtagtgactg agggggtcga agaccaagaa gtgagttatt tataccaagc

7261 attcttctta tggctagatc caatctcctg gtccctgcgg aaaggaaaaa gaatttcacg

7321 ctcttccttt cgggaaggga ggattcggga aatgcagctt tctccagacc tccgggaaaa

7381 gcatgaaaaa aacggctcga atggtacgat ccctccgtca ccccagaatg aagggggtga

7441 tctcgtagtt cttggtctgt gaagatgcgt tgttaggtgc tccattttct tttcccattg

7501 aggccgaacc taaacctgtg ctcgagagat agctgtccat acactgataa gggatgtatg

7561 gattctcgag aagagaggag ccgcggtggt ccccccccgg accgcccgga tcccacgagt

7621 gaatcgaaag ttggatctac attggatctc acccgaatcg ccccatcgat cctcctgagg

7681 agaggtttgg tttcaaaccc ctgttcgaac aggaggagta cgccatgcta atgtgccttg

7741 gatgatccac atctcagggt caggcgccga tgagcacatt gaactatcca tgtggctgag

7801 agccctcaca gcccaggcac aacgacgcaa ttatcagggg cgcgctctac cactgagcta

7861 atagcccgtc gtgcgagcct cccgcagggg gcccgctatc gtgcgagcct cccgcagggg

7921 gcccgctatg ccaaaagcga gagaaacccc atccctctct ttcctttttt cgcccccatg

7981 cccccacacg gggggcatgg ggacgtaaca aaggggatcc tatcaacttg ttccgaccta

8041 ggataataag ctcatgggct tggtcttact tcaccgtcga gaaacgaaag aagacttcct

8101 ttaactcaga cgcagctccc ttctttttgt tttgagtgtg aagcagtggc aaaccaaaat

8161 acccaacaag cattagctct ccccgaaaag gaggtgatcc agccgcacct tccagtacgg

8221 ctaccttgtt acgacttcac tccagtcact agccctgcct tcggcatccc cccccttgcg

8281 gttaaggtaa cgacttcggg catggccagc tcccatagtg tgacgggcgg tgtgtacaag

8341 gcccgggaac gaattcaccg ccgtatggct gaccggcgat tactagcgat tccggcttca

8401 tgcaggcgag ttgcagcctg caatccgaac tgaggacggg tttttggggt tagctcaccc

8461 tcgcgggatc gcgacccttt gtcccggcca ttgtagcacg tgtgtcgccc agggcataag

8521 gggcatgatg acttgacgtc atcctcacct tcctccggct tatcaccggc agtctgttca

8581 gggttccaaa ttcaacgatg gcaactaaac acgagggttg cgctcgttgc gggacttaac

8641 ccaacacctt acggcacgag ctgacgacag ccatgcacca cctgtgtccg cgttcccgaa

8701 ggcacccctc tctttcaaga ggattcgcgg catgtcaagc cctggtaagg ttcttcgctt

8761 tgcatcgaat taaaccacat gctccaccgc ttgtgcgggc ccccgtcaat tcctttgagt

8821 ttcattcttg cgaacgtact ccccaggcgg gatacttaac gcgttagcta cagcactgca

8881 cgggtcgata cgcacagcgc ctagtatcca tcgtttacgg ctaggactac tggggtatct

8941 aatcccattc gctcccctag ctttcgtctc tcagtgtcag tgtcggccca gcagagtgct

9001 ttcgccgttg gtgttctttc cgatctctac gcatttcacc gctccaccgg aaattccctc

9061 tgcccctacc gtactccagc ttggtagttt ccaccgcctg tccagggttg agccctggga

9121 tttgacggcg gacttaaaaa gccacctaca gacgctttac gcccaatcat tccggataac

9181 gcttgcatcc tctgtcttac cgcggctgct ggcacagagt tagccgatgc ttattcccca

9241 gataccgtca ttgcttcttc tccgggaaaa gaagttcacg acccgtgggc cttctacctc

9301 cacgcggcat tgctccgtca ggctttcgcc cattgcggaa aattccccac tgctgcctcc

9361 cgtaggagtc tgggccgtgt ctcagtccca gtgtggctga tcatcctctc ggaccagcta

9421 ctgatcatcg ccttggtaag ctattgcctc accaactagc taatcagacg caagcccctc

9481 ctcgggcgga ttcctccttt tgctcctcag cctacggggt attagcagcc gtttccagct

9541 gttgttcccc tcccaagggc aggttcttac gcgttactca cccgtccgcc actggaaaca

9601 ccacttcccg tccgacttgc atgtgttaag catgccgcca gcgttcatcc tgagccagga

9661 tcgaactctc catgagattc atagttgcat tacttatagc ttccttgttc gtagacaaag

9721 cggattcgga atttcattcg aaggcataac ttgtattcat gcgcttcata ttcgcccgga

9781 gttcgctccc agaaatatag ccatccctgc cccctcacgt caatcccacg agcctcttat

9841 ccattctcat tgaatcacgg cgggggggca aatctaaaca gaaaaactca cattgggttt

9901 agggataatc aggctcgaac tgatgacttc caccacgtca aggtgacact ctaccgctga

9961 gttatatccc ttccctgccc ccatcgagaa atagaactga ctaatcctaa gtcaaagggt

10021 cgagagactc aacgccacta ttcttgatct tgaacaactt agagccgggt cttcttttcg

10081 gactatttcg gatatgaaaa taatggtcaa aataggattc aattgtcaac tgcccctagg

10141 attgactagc gattccgaag gaactggagt tacatccttt ttccatttcc attcaagagt

10201 tcttatgtgt ttccacgccc ctttgagacc ccgaaaaatg gacaaattcc ttttttcctt

10261 ttcttaagaa cacatacaag attcgtcact acaaaaagga taatggtaac cccaccatta

10321 actacttcat ttatgaattt catagtaata gaaatacatg tcctaccgag acagaatttt

10381 gaatttgcta gtctcttgcc tagcaggcaa agaaaagatt tatccgcgtg gaaaggatga

10441 ttcgtttgga tcgacatgag agtccaactg cattgccaga atccatgttg tctatttgaa

10501 agaggttgac ctccttgctt ctctcatgtc cacgccgagc cccttttctc ctcggcccgc

10561 agagacaaaa tgtaggactg gtgccaacag ttcatcacgg aagaaaggac tcactaagcc

10621 gggatcatta actaatctaa atcactaact aatactaatc taatataatc gaaaatacta

10681 atataataga aaagaactgt cttttcagta tactttcctc ggttccgttg ctatcgcggg

10741 ctttacgcaa tcgatcggat tagatagata tcccttcaac ataggtcatc gaaaggatct

10801 cggagaccca ccaaagtgcg aaagccagga tctttcagaa aacggattcc tattcgagaa

10861 gagtgcataa ctgcatggat aagcttacac taacccgtca atttgggatc caattcgata

10921 ttttcctggg gaggtatcgg gaaggaattg gaatggaata agatcgattc atacagaaga

10981 aaaggttctc tattgattct gtacctatgg gatagggata gaggaagagg aaaaaaccga

11041 agatttcaca tagtactttt gatcgaaaaa gaaatctgat tgatttcgta cccttcgttc

11101 aatggtcaaa ttctacagga tcaaacctat gggacttaag gaatgatata aaaaaaagag

11161 agggaaaaag aaatgaatat taaataaaaa tgaagtagaa gaacccagat tccaaatgaa

11221 caaattcaaa cttgaaaagg aaaggacctt tctgattctc gaagaatgag gggcaggggg

11281 gttggtcgag aaagatctct tgttcttatt ataggatcgt gattggatcc gcatatgttt

11341 ggtaaagaga ataatcttct cctttgagaa taatcaaaaa tggaaagtgt tcaattggaa

11401 catgaaaacg tgactaaatg ggtcctagtt actattcggg acagagtgga agaggggagt

11461 ggattctcga gcgcggaaag ggatccaatg aattcgaaag aattgaacga ggagccgtat

11521 gaggtgaaaa tctcatgtac ggttctgtag agtggcagta agggtgactt atctgtcaac

11581 ttttccacca tcacccccaa aaaaccaaac tctgccttac gtaaagttgc cagagtacga

11641 ttaacctctg gatttgaaat cactgcttat atacccggta ttggccataa ttcacaagaa

11701 cattcttcag tcttagtaag agggggaagg gttaaggatt tacccggtgt aagatatcac

11761 attgttcgag gaaccctaga tgctgtcgga gtaaaggatc gtcaacaagg gcgttctagt

11821 gcgttgtaga ttcttatcca agacttgtat catttgatga tgccatgtga atcgctagaa

11881 acatgtgaag tgtatggcta acccaataac gaaagtttcg taaggggact ggagcaggct

11941 accatgagac aaaagatctt ctttctaaag agattcgatt cggaactctt atatgtccaa

12001 ggttcaatat tgaaataatt tcagaggttt tcccttactt tgtccgtgtc aacaaacaat

12061 tcgaaatacc tcgacttttt tagaacaggt ccgagtcaaa tagcaatgat tcgaagcact

12121 tctttttaca ctatttcgga aacccaagga ctcaatcgta tggatatgta aaatacagga

12181 tttccaatcc tagcaggaaa gggggggaaa cggatactca atttaaagtg agtaaacaga

12241 attccatact cgatctcata gatacatata gaattctgcg gaaagccgta ttcgatgaaa

12301 gtcgtatgta cggcttggag ggagatcttt catatctttc gagatccacc ctacaatatg

12361 gggtaaaaaa gccaaaataa gtgattttag cccttataaa aagaaaactg attcttaaac

12421 ccctttcacg ctcatgtcac gtcgaggtac tgcagaagaa aaaacagcaa aatccgatcc

12481 aatttatcgt aatcgattag ttaacatgtt ggttaaccgt attctgaaac acggaaaaaa

12541 atcattggct tatcaaatta tctatcgagc catgaaaaaa attcaacaaa agacagaaac

12601 aaatccacta tctgttttac gtcaagcaat acgtggagta actcccgata tagcagtaaa

12661 agcaagacgt gtaggtggat cgactcatca agttcccatt gaaataggat ccacacaagg

12721 aaaagcactt gccattcgtt ggttattagc ggcatcccga aaacgtccgg gtcgaaatat

12781 ggctttcaaa ttaagttccg aattagtgga tgctgccaaa gggagtggcg atgccatacg

12841 caaaaaggaa gagactcata aaatggcaga ggcaaataga gcttttgcac attttcgtta

12901 atccatgaac aggatctata catctcgatc ggaaaagaat caagagaaaa agaaagaatc

12961 ggaattgatc gatagatttc tcgaaacaaa cgaaagatga aacataaatc atggatcaac

13021 taagccctct cggggacttt cttaaagagg aacctcatgt aaataccatg gaataaggtt

13081 tgatcttgat cctattccat tccaaaaacg gaaagttcga cacaattggg attttttttg

13141 gaaattggat gcagttacta attcatgatc tggcatgtac agaatgaaaa cttcattctc

13201 gattctacga gaatttttat gaaagccttt catttgcttc tcttcgatgg aagtttgatt

13261 ttcccagaat gtatcctaat ttttggccta attcttcttc tgatgatcga ttcaacctct

13321 gatcaaaaag atataccttg gttatatttc atctcttcaa caagtttagt aatgagcata

13381 acggccctat tgttccgatg gagagaagaa cctataatta gcttttcggg aaatttccaa

13441 acgaacaatt tcaacgaaat ctttcaattt cttattttac tatgttcaac tctatgtatt

13501 cctctatccg tagagtacat tgaatgtaca gaaatggcta taacagagtt tctcttattc

13561 gtattaacag ctactctagg aggaatgttt ttatgcggtg ctaacgattt aataactatc

13621 tttgtagctc cagaatgttt cagtttatgc tcctacctat tatctggata taccaagaaa

13681 gatgtacggt ctaatgaggc tactatgaaa tatttactca tgggtggggc aagctcttct

13741 attctggttc atggtttctc ttggctatat ggtttatccg ggggagagac cgagcttcaa

13801 gaaatagtga gtggtcttat caatacacaa atgtataact ccccaggaat ttcaattgcg

13861 ctcatattca tcactgtagg aattgggttc aagctttccc cagccccttc tcatcaatgg

13921 actcctgacg tatacgaagg agtgcggttc gttcgagaaa ttcctacctc tctatctatc

13981 tctgagatgt ttggattttt caaaactcca tggacatgca gaagagaaat gctatcccca

14041 ctcggaccaa gacagaactt ttacttgttc aaataacaat taaggtgaag cagggtcagg

14101 aacgacgaat ctctttatga taaacagatc cattttgcaa gttcgttatt acgggtagtt

14161 cctacaaagg atccgactaa tgacgtatac aatacttgaa ttctcgatgt agatgctaca

14221 tagttggttc tcatccttca gagactacga gtgtaatagg agcatccgtc gacaaaagga

14281 tcaccctaag atgatcattt catggctatt gagaacgaat taaatcagat ggttctattt

14341 ctcaatcttt ctgacttgct cctacggaac caaggttgaa aagattgaaa aaataagtca

14401 ttcacctgat gaaggattcc tcgaaaagtt aaggattagt aatctttttt agaaatcgaa

14461 tggattcggt cttatacata cgcgaggaag gtaatcaaaa aagaaagaaa acgagttctt

14521 ctttctttta tcacttagga gccgtgtgag atgaaagtct catgcacggt tttgaatgag

14581 agaaagaagt gaggaatcct cttttcgact ctgactctcc cactccagtc gttgcttttc

14641 tttctgttac ttcgaaagta gctgcttcag cttcagccac tcgaattttg gatattcctt

14701 tttacttctc atcaaacgaa tggcatcttc ttctggaaat cctagctatt cttagcatga

14761 tattgggaaa tatcattgct attactcaaa caagcatgaa acgtatgctt gcatattcgt

14821 ccataggtca aatcggatat gtaattattg gaataattgt tggagactcc aatgatggat

14881 atgcaagcat gataacttat atgctgttct atatctccat gaatctagga acttttgctt

14941 gcattgtatt atttggtcta cgtaccggaa ctgataacat tcgagattat gcaggattat

15001 acacgaaaga tccttttttg gctctctctt tagccctatg tctcttatcc ctaggaggtc

15061 ttcctccact agcaggtttt ttcggaaaac tctatttatt ctggtgtgga tggcaggcag

15121 gcctatattc cttggtttta ataggactcc ttacaagcgt tgtttctatc tactattatc

15181 taaaaataat caagttatta atgactggac gaaaccaaga aatcacccct cacgtgcgaa

15241 attatagaag atctccttta agatcaaaca attccatcga attgagtatg attgtatgtg

15301 tgatagcatc tactatacca ggaatatcaa tgaacccgat tattgcaatt gctcaggata

15361 ccctttttta gcttctaggg tctatttctt agttcaagat ccctcttact aactggaatc

15421 aaagaattag tagatctgtt ccgaccaaaa cgggaatggg ctggggttat gaacttataa

15481 tctgatgatc gagtcgattc catgattata agttcattcc ataccggacc gggccggaat

15541 agggttatat acattctcaa ggggtcattc gcgcgtatct aaatagatac tatgtttaca

15601 tatgggtccc tacgccgttc catttaggat taggaatagg cgtaatcgga cctgcttttt

15661 ccccatattt ctcgttattt ttgggaccct attcacccct ttgagcttct attgaatcga

15721 gaaatcaaat aggtttgatt ggccatcttt ttgatatata cataaggcat tctccggata

15781 attcaaatcg aagcaattgg atgtccaact cgggcctata tgaccgatca atagaaatac

15841 tacacccttg tcatatattc catacatcac actagataga tatcatattc atggaatagg

15901 atgcactttc aagatgcctt ggtggtgaaa tggtagacac gcgagactca aaatctcgtg

15961 ctaaagagcg tggaggttcg agtcctcttc aaggcataat attgagaatg ctcattgaat

16021 aagcaattca ataagggagc tcggatcaaa tcggtattga tataccgacc tgcacccgcc

16081 ccagagtata cgattcaaca ggaatcacac aagggtagat tagaaacctc tggtaaaatg

16141 cccgccagtc acccagcgga taaagtacat tacatagtcc agggattggc gacttaccca

16201 ttcagtgact ttggcactgg acgtgcccaa aaggtatcgg gtcgggtcaa ttcaataata

16261 gacgcctgtt agcattccac ccttccttct cctttcaggg cctatccgaa agagaatcca

16321 gtacttcttg gtcgtgaata tctgaactgg ttgttcgctg ttcaagaatt cttgtttggg

16381 cagttcatac catccataca tagtgttttg atctaagatt ttaattcttc cgtgtttcag

16441 cagtaacata ttgttccatg gagctaaggt ccaaaatagg gaagaaagaa gcgtttccac

16501 cactctacca cccagtccat tctgttccac tgaatccctc tttcatagcc acatatcttt

16561 ccggctaagg aatgggaaat ctttctcctg ttccatgaat ccaattttca tttcatccgg

16621 gaaaagccat ctttttctta ccaatgtctt tgtcatttga tccaatagcg ttccgttaga

16681 taggaacaga gttgataaat actgataact ctcggataga gtattagaac ggaaagatcc

16741 attagataat gaactattgg ttctaagcca tctctgacga ttaatcaaca attcgaagtg

16801 cttttcttgt gtattcttga taaaccagcg tttatatata gatgtaggag gatccgtttg

16861 ggaagtaaga agcccctttg acatctcttc atctgcaaat aattctcgat gtgaaaacac

16921 agagccaggg ggctgatctt tgaataggaa aaagagcgga tccgcagggt cccaaatgaa

16981 ttggcttatt tgaaaaaggc cttgttcttt ggaagatcta tctcgtgtct ggtactgcat

17041 ggttccactc tgcaagaact ccgaatcatt ctcttgaagc tcatcctctt catcataaat

17101 gatccgcttg ccccggaatg acctggacca atagggaaat cccaattcat tgggcctttc

17161 gatacaatca aatagaaagg cccaagggcg ccatattcta ggagcccaaa ctatgtgatt

17221 gaataaatcc tcctctatct gttgcgggtc gagggctgct tcccccttcc cttcttcaaa

17281 ctccgattcg tatttttcat agagaaatct ctgatcaaag atagaacaag atccattttg

17341 catcatatct aagggattcc tcagttcggg ccgaagaagc aatgtaactc gatcattatc

17401 aaacggactg caatcttttt ctgtccgtga agatcccacc agagcgcctt ctacttctaa

17461 taggccatga actagatccg aatcattctc aacgagtcca taagaagtga tcccattttt

17521 ttcatcggat ccgggtagag accaaagatc ttgagcgacc gatccggcag aacaactcaa

17581 aagataaaga agtatcgtta atttcttcat gctcgttcca agttcgaagt accatttgta

17641 caaataagaa tccccttcgt tacatgattt cttcttcata tagatagata taggatctat

17701 ggggcaatta cttagaagta cattttgtgc tacagccctt cctatttgat agaaaaggat

17761 cccatgatcc tgaaccgatc ttacctggga tcgcaaatcc caagtttgtc tatgaagagc

17821 ggatctaatt gtcttagtgt ctataatgga tttcttctgt gtaatactaa tcgatagggc

17881 ctcattggta agtgctacaa gatctcttgc attggaaccc atggttatgg acccgaatcc

17941 gttagtatgg aacattttct tttccaagtg aaatccctta gtatatgaaa gagtgaaaaa

18001 gtgctttcgt tgttgtggaa taagaagcct tcttatctta atgcacgtat ttaatttatt

18061 cggagctatt agagcgggat ccactttttg gggaatatga gtcgaagcaa taacaagaat

18121 atttctagtg gaacatcttt cagagagatg gttcactaat agaccgaggg ataagtaatt

18181 cgactcattc acatccagat catgaatgtt tggaatccat attatgcaag gagatattgc

18241 ttttgccaat tcgaattgaa gggtgatata aaatcggtct atttccggca tcctatccat

18301 agttagcagt tccagctccg tatcaaggtc acgatcaata tcgtcactag catcaatatc

18361 gtcactagca tcaatatcgt cactagcatc aatatcgata tcatcaaaaa taaaaccttt

18421 aggcttgtta tccaggaact tgttcagaaa taccgtaatg aaaggaacat aggagtttgt

18481 tgctaggtat ttgaccaaat aggatcgtcc agttcctata gaacctatca ctaaaatccc

18541 cctagagggg gataaggcta agcggagcga aaagggtttt ccatgagatg ggaaatgcaa

18601 agtattagtc ccacacgaag tttgtgaata agtgattgtc tgataatgag caaggaatac

18661 ccgtctttct gctaaagagg atgtattgaa ctcataattc aatagatact ttttatgaat

18721 gtcaactaag tatcgtaagt aaattgctcc cggttgttca atcatttgat aaccagagtc

18781 attctttgat aaatgatcac tatgagtcag actcaataga atttgatcaa tccttttttc

18841 tgtcgttaag gcggagaact gaaccaagaa ttctctttct tcatcatcaa tcgaatcact

18901 gttcgcgacc caggattcga ttttatcatc aatccaatcc ccattcacgt tttttctttt

18961 tcttatcaat gaatagatct ctttacttgt atgacttaga tgtctcgtat ttctcgaaaa

19021 agtgatttga ttgatgggat ttggtatgag atcgatgaga ttgatactca aatatttctt

19081 cttagaacgt attgatttga ccccataagc gggaccaggc atgttgccgc cagaagcccg

19141 tatttcttct agagaatctc ctaattgttc cagagcaact agaaagagat tctttaacca

19201 gaaagaattc ggtgcagatg taggatacct atccagaagt tttcgtaact caatcataga

19261 tgatggaatc atcaaagatt tgaccttttc gaactctgtc tgtaactcac tagaggcccg

19321 ggaaacaaag agaagatgtg tacgaacgag atatccagca acaagaagaa ggaaaaggat

19381 tgaatagagg aactcccgaa catttggcga tctcagatgt gtcgatggtg actcattatt

19441 tcgatgaatc atttcttcgg acagaagaag attatggaaa cacttactcg aaatctcact

19501 tatcagattc cattgtggaa gacaccattt tttctgaaga attcgccatg ctataccgga

19561 tccatgcata atatcatgaa aaatggatac aaatttttga ctgctactta gtatcggcaa

19621 taggtctgaa aaagtatcta aaaatatcaa atttagatat ttgtaccctg tcgaagtaag

19681 gaaccatggt atatatgttt ggaatagatt ccattttgag agagttgaaa aagcaccatc

19741 tcgttgaaag gttctataca tctgcccttt ctcaacgcat ttctttagac aaagactcca

19801 ccttttcctc ttttcggatg gtaaatattt cccagaacat ggagtgtgaa tcaaacccac

19861 gtttgaattg agatactgat gcaagttctt cccttctgaa tcagatagat tcatatctga

19921 aagaggttga caataagttc tttcaaaatg gactatttgt ccctctgtta gaggtgttcc

19981 aaacatgtct gcgatcgagt aaatagttct acgaacgaat ggatcgtgtt gacttggaaa

20041 atggaaagat ttgtacaagt tatacgtttc gtcaccactt tgtggaaaat cgttaggtat

20101 gaatatgtta gatacctgtg actcgattgg tgaaatagta tctctccccc caaaagcatg

20161 ttttttttta ccgacgcaca aagaaaatat tttgttgcga atgaacaaga tattgaggaa

20221 ttgtccatac gtaaaatcat aattattgat acgggccttt tccacagaaa aggggaatct

20281 tgtgttacag tagaagcaga agtgatgtgg attattcaag aatcgaagtc gatttgcttt

20341 ataaaaagaa gatatcaatg aacttatatg aaatggtttc acgggattca gccaattgtc

20401 ttgattgtgg aatatcattg agaaatagga atccgcgtta tcaaaggatt tcctgcgatt

20461 atttctagta tggaatgagt caatcatcca ctttggtatc ttattgaaca aaaagggcgt

20521 tcctccattg atcaagaatt tcgattttcg ggaagtatca tgctcatcca ataagaaggg

20581 tttccatttt ttcaaatgaa caatttgaag acctattgat tctaacaact gattgcagag

20641 ttgatcgttt ggacctttca attcatagat gtagatctcg gacctatgaa tggggatatt

20701 cccgaaactc acacagaaaa aaggaagtga gttcgacaaa aagaaaagca acttggacaa

20761 aaaaagaagt gacttggaca aaaagaaacg aagtggctta gacaaatctt ttttgtcgat

20821 aacctcagac caatcaatcg aatattgatt aatacgtaat cgatcgaaca ctatttgaaa

20881 acggctcttc tgctcagaaa cgaaatgttc caaatgttcc tggaaattct tgctcccgtt

20941 ggaccatttg tatctatatg catcaggagc ccgattcatg gatctctcgg ttcgagaaat

21001 caagataaga ggatcgagcc atttcttctg actctttttc aaattcgata aatgtgggtt

21061 gatcgtatat ttcattatag ttctatgatt cagagtatcc tttcctattt gatccctttg

21121 aattccatat tcgaagttgc gatcggatcg attcattaaa aagagtcgat tcaatacatt

21181 tcttatgtac ccataggtac tatattggat ttgaatcaga tttcggatca atctatattg

21241 attgactgcc tccattatgt tgttgctagc aaataccact atttttggtt ttggatcttc

21301 caaatcattc ccgcaagaga tccggaccca tttttttctg atccttcgag aaaaagattc

21361 attctcttca taaaaaatag gaggtagaac caataaagat ttctttttcg attcatccct

21421 ggcctcattc aagaattgtt tttgatccaa tccgtaggaa tcaatagaaa agggaaatcc

21481 cttatgatac accagatccg gctcggttat tgatagagtg aatagatctg ccatttcttg

21541 aaatctctct tctgattcaa aatcgtggtg taacgtgtat cctcccctgt tccggtcatg

21601 gaatagatga aataaatcaa aaaatggatt tttgttcaag aatgaaatct tattggaact

21661 gtccatatcc gggtcatcct tcagaaccct atcacacccc ggatctgatg aaataggatg

21721 aattgagaca gtattttgta aatacgtaat tatcttgaat atattaacca gttctttatt

21781 ttccgatcgc ctgggaggga caaaagaaag atcttgttgt ttcttcaaca atttcggatc

21841 tctagtggac ctctcagtag gattcgaacc cagatgaagt tctgaccatc tatcagagaa

21901 aaaagaacga accgatcttg taggattccc aagaaattct tggatttctt ccggaagcag

21961 atgattaatc atctgcttct cacgttccgt gaatagccgg gacattgagg aatatccaga

22021 aaggcatttc gggaatcggt ctgattctat ctctgttcct tccgtttgaa gaaaggaagg

22081 atcccaaaga atcgatcttt cttttagtgg ttgaatctct ctttgattga tcaatgtgtg

22141 atattccgaa tcctcattac taatggaatc caaatgatct ctggattgat cagaagatcc

22201 tttcagttgg ctagaatccg ttacttgaac gaaactggat cttgtggaat catattgaat

22261 atttgacgat acattccgta cctttctaaa aaaccgatcc ttgtttacca accacacatt

22321 gtctaaccaa atccaattct ctctcgatac gttcctcaaa aaatccgatt cgtgcggatt

22381 cttcccccaa ctaacgaaga gatcttggcg aaattgccac atatgaaatt gagcacaatt

22441 ttgcaaagaa atagcccgct tgtttctcga gaggagatgg gaaacatgct caatatcatt

22501 tgattgaata gttgacccag ccctttgttg tttgaagaaa ccctccactt caattggtat

22561 tttttcacga aaagcagaca tgagataaga aatccagtgt ttcactaaga tttcgaatag

22621 cgggcccgaa ttcaagttga ttctatttcg cctcttcctc agagaaagac gatcaaacaa

22681 ttcccaatca tggtccttgc ggatcggatc atccatataa tatacaaaaa gaaactccag

22741 atatttgaga tctttctctt tgaataagat cccaattcca gtgacggttt cattagatat

22801 cttacaacta gaatccctct tttttccgat ccagttcctc caccaccgcg aaccccagtt

22861 agattcaggc atgctacact ttttagttat tgggagaacc caagtactct ctttcggatt

22921 caggaaacaa ctctcagaga tcttttttcc ttttggaaga tacaggagcg aaacaatcaa

22981 cctattgata ttggaagacc caaccgattc ttccaatgta tcatttctgg gtccaatgga

23041 attcataggt ataggaagaa gccccctcaa atagagattt tttctttcga ccatatttcg

23101 attgttaata cgatatataa ggaccgctac tacaaatagt agtacaccct tgatcgtgaa

23161 atatcgattg cttgttgaac cctgtgaatt gcgtgaaagt aggatactca aaattcgggg

23221 gtcaaagagt tttagaaaac gttcttggtg gaaaaaaatg cgaatgaaag accccactga

23281 attgaattgg gtccatgaat ctaagaaaca gtgagaattc ttgatctctc tcaatatctc

23341 tctcaattcg aaaatccagg attggaattg atgtcctttc attgattcct cctaaattgc

23401 attgatttat cctaaagatt tcatttcaat tggaatttgg ttattcacca tgtacgagga

23461 tcccccctaa gcatccatgg ctgaatggtt aaagcgccca actcataatt ggcgaattcg

23521 taggttcaat tcctactgga tgcacgccaa tggaaccctc caataagtct attggaattg

23581 gctctgtatc aatggaatct catcatccat acataacgaa ttggtgtagt atattcatat

23641 catagtatat gaacagtaag aaatagcatt cttattgaga ctagaactca tagggaagaa

23701 attcgattta tggatggaat caaacatgca gtatttacag acaaaagtat tcggttattg

23761 gggaaaaatc aatatacttc taatgtcgaa tcaggatcaa ctaggacaga actaaagcat

23821 tgggtcgaac tcttctttgg tgtcagggta atagctatga atagtcatcg acttccggga

23881 aagggtagaa gaatgggacc cattatggga catacaatgc attacagacg tatgatcatt

23941 acgcttcaac cgggttattc tattccacct cttagaaaga aaagaactta aatcaaaata

24001 cttaatagca tggcgataca tttatacaaa acttctaccc cgagcacacg caatggaacc

24061 gtagacagtc aagtgaaatc caatccacga aataatttga tctatgggca gcatcattgt

24121 ggtaaaggtc gtaatgccag aggaatcatt acctcaaggc atagaggggg aggtcataag

24181 cgtctatacc gtaaaatcga ttttcgacgg aatgaaaaag acatatatgg tagaatcgta

24241 accatagaat acgaccctaa tcgaaatgca tacatttgtc tcatacacta tggggatggt

24301 gagaagagat atattttaca tcccagaggg gctataattg gagataccat tgtttctggt

24361 acagaagttc cgataaaaat gggaaatgcc ctacctttga gtgcggtttg aactattgat

24421 ttacgtaatt ggaagtaacc aattaggttt acgacgaaac ctagaaatcg atcactgatc

24481 caatttgagt acctctacag gatagacctc aacagaaaac tgaagagtaa cggcagcaag

24541 tgattgagtt cagcagttcc tcatataaaa ttattgactc tagagatata gtaatatgga

24601 gaagacaaaa tagtttcaag caccgacaga accggaagcg ccccttcttt caaagagagg

24661 aggacgggtt attcacattt catttgatgg tcagaggcga attgaaagct aagcagtggg

24721 aattctaaag attccccggg ggaaaaatag agatgtctcc tacgttaccc ataatatgtg

24781 gaagtatcga cgtaatttca tagagtcatt cggtctgaat gctacatgaa gaacataagc

24841 cagatgacgg aacgggaaaa cctaggatgt agaagatcat accatgagtg attcggcaga

24901 tttggattca tatagatatc cacccatgcg gtacttcatt gtaccatata tataagatcc

24961 gtctgtatag atatcatcat ctacatccag aaagccgtat gctttggaag aagcttgtac

25021 agtttgggaa ggggttttga ttgatcaaaa agaggaatct acttcaaccg atatgccctt

25081 aggcacggcc atacataaca tagaaatcac acttggaaag ggtggacaat tagttagagc

25141 agcgggtgct gtagcgaaac tgattgcaaa agaggggaaa tcggccacat taaaattacc

25201 ttctggggag gtccgtttga tatccaaaaa ctgctcagca acagtcggac aagtggggaa

25261 tgttggggcg aaccagaaaa gtttgggtag agccggatcc aagcgttggc taggtaagcg

25321 tcctgtagta agaggagtcg ttatgaaccc tgtagaccat ccccatgggg gtggtgaagg

25381 gcgagcccca attggtagaa aaaaacccac aaccccttgg ggttatcctg cacttggaag

25441 aagaagtaga aaaaggaata aatatagtga aaatttgatt gttcgtcgcc gtagtaagta

25501 gtaaataggc gagaaaatag aatttctttc ttcgtcttta aaaaaaatag gagtaaactg

25561 tgatacgttc actaaaaaaa aatccttttg tagccaatca tgggcgaacg acgggaattg

25621 aacccgcgca tggtggattc acaatccact gccttgatcc acttggctac atccgcccct

25681 ctactattac tataatgaaa atgaatttac tttaaaatgc aaaatcaatt gaattaaaat

25741 catttttttc ttctttcttt ttctgaatcc tgtaatggaa agaaaaaaag taattcaaaa

25801 gaaaattaag gagcaataac ccctcttgat aaaacaagaa agagtttatt gctccttata

25861 tttatctatt ctattttcaa aaactcctat acactaagac caagtcttat ccatttgttg

25921 gagcttcgat agaagctagg tctagaggga aattatgagc attacgttca tgcataactt

25981 ccataccaag gttagcacgg ttgatgatat cagcccaagt attaattaca cggccttgac

26041 tatcaactac agattggttg aaattgaaac catttaggtt gaaagccata gtgctaatac

26101 ctaaagcagt gaaccagata cccactacag gccaagcagc taggaagaag tgtaatgaac

26161 gggaattgtt gaaactagca tattggaaga tcaatcggcc gaagtaaccg tgagcagcta

26221 cgatattata agtttcttcc tcttgaccga atctgtaacc ttcattagca gattcatttt

26281 ctgtggtttc cctgatcaaa ctagaagtta ccaaggaacc atgcatagca ctgaataggg

26341 agccgccgaa gacaccagcc acgcctaaca tgtgaaacgg gtgcataagg atgttgtgct

26401 cagcctggaa tacaatcatg aagttgaaag taccagaaat tcctagaggc ataccatcag

26461 aaaaacttcc ttgaccgatt gggtaaatca agaaaacggc agtagccgct gcaacaggag

26521 ctgaatatgc aacagcaatc caaggtcgca tacccaaacg gaaactaagc tcccactcac

26581 gacccatgta acaagctaca ccaagtaaga agtgtagaac aattagttca taaggaccgc

26641 cgttgtataa ccattcatca acggatgctg cttcccagat tgggtaaaag tgcaaaccga

26701 tggctgcaga agtaggaata atggcacctg agataatatt gtttccgtaa agtagagatc

26761 cagaaacagg ctcacgaata ccatcaatat ctactggagg agcagcaatg aaggcaataa

26821 taaatacaga agttgcggtc aataaggtag ggatcatcaa aacaccaaac catccaatgt

26881 aaagacggtt ttcggtgcta gttatccagt tacagaagcg accccacagg ctttcgcttt

26941 cgcgtctctc taaaattgca gtcatggtaa aatcttggtt tatttaatta tcagggactc

27001 ccaagcacca aaattttcta taccataaat agaaatagaa aacggaaggc ttgttattca

27061 acagtataac atgacttata tgttcgtgtc aaccaatccg aataaatatc tagatctatt

27121 cgaatttatt ataactaaat gaagtggatt acaaaaagaa aatatgaatt gctatacata

27181 gaatttctat atggtggtgg gttgcccggg attcgaaccc ggaactagtc ggatggagta

27241 gataatttct ttgttatctt aattaaataa agaaaaaccc ctccccaagc cgtgcttgca

27301 tttttcattg cacacggctt tccctatgta tacatcattt cctttcttag aaatactttc

27361 aaaacaaaag ttgaatactc agttgattta acccttatta catactacat caacatttca

27421 gaatagtgga aatccaattt tgatcttttt atctctcgat ccattttttt aggaaaaatg

27481 gatattttaa agattttaga atttggattt ttgattcgcc agatcattga tagaaatcat

27541 atccaaatac caaatccgac ttctatatac tcctcgcaaa gtgtaagagg tttttttgaa

27601 gttcaaaaaa aggacgtgtt cttccgacat aagaaattct tctaaaaatt ccgagcctaa

27661 tcttttcaaa aaagcacgta cagtactttt gtgttttcga gccaaagttc tagcgcaaga

27721 aagtcgaagt atatacttta ttcgatacaa actctttttt tcggaagatc cactatgata

27781 acgagaaata tttctgcata tacgcccaaa tcggtcaata atattagaat ctgataaatc

27841 agcccgaatc ggcttactaa caggatgccc caatacgtta caaaattttg ctttagccaa

27901 tgacgcaatc agaggaataa ttggaacaag agtctcaaac ttattaatag cattattgat

27961 tagaaatgaa ttttctatac tttgactgcg tactacggaa gggctgattc gcacgcttga

28021 aagatagccc aaaaactcaa gggaatgctt ggataattgg tttatataaa tgcttcttgg

28081 atgaaaccac accgaaaaat gccattgcca aaaagtgaca aggtaaaatt tccatttctt

28141 catgaaaata aatgtccctt ttgaagccag aatggatctt ttttgatacc taatataatg

28201 catgaaaggt tccttgacca accgcaggtt tgttcccaaa tccttaatct taacaaagac

28261 gttcacaaga cgttccattt ttatatagaa atagattcgt tcaagaaaaa ctccagaaga

28321 tgttgatcgt aaataaaaag attggttacg tagaaagacg aaaatggatt catattcaca

28381 tacgtgagaa ttatataaga ataagaataa tcttttattt ttttttgaag aaggggaact

28441 ggctttcttt ggaataataa gactattcca attacaatat tcattgagaa agactcgtaa

28501 taaatgcaag gaggaagcat cttttacgca atcgcgaagg atttgaacca agatttccac

28561 atgaacaggg cgaggtatta ccatatctaa cacaaaatta aaatgtgaaa aagtgtcctc

28621 gaaaaaggga aatattgaat gaattgatcg taaattctga gattttccta tcttgttcgt

28681 ttccccttct agacaagata ggaattgtaa agaaaatgga atttcgacaa taaaagcaaa

28741 cccctctgat atgatttgag aatacaaatt attgttgcgc gcccaaaacg gattttggtt

28801 agaatcatta ggagaaatca gaaaatggtt ctgttgatac agtcgagtaa ttaaccgttt

28861 cacaatcagt aaactggatt tattgtcata acccagattt tctgacaaaa tcaatttact

28921 caaagcacga ttatgagcaa atgcataaat atactcctga aagataagtg gatataggaa

28981 gtcatgttgt tgagatctct ccagctgtaa atatctttgg atttcctcca tttgaaattc

29041 gattttaatt aaaggtagaa gattgggggg gttatcaaat gatacatagt gcgatacagt

29101 caaaacaagg tattctgtaa caatcgatac ctcgggaaca gataaactta tcaacagatt

29161 ctctaccctc tctttttcca tccatttcat ttaatttgtc tatgtttatg ttatagacca

29221 taaagatggt tagaaatctt ttatttgtta aaccgaatcg ctcttttgat ttcggaaaaa

29281 actttcttta tcaatatact gcttctttta cacacgcatc ttcagtccat aatggagaat

29341 gtcaatagtt aggattcatt aaaaaaaaat agaatccact cgtggagtga taagtgcttc

29401 ccgtatcagg caccaattta tttttaacgt ctagttagat cgggaaatta ttcaaattaa

29461 gaacagaagc cggttgcttt ttctttctga taattaattg aagccacaag gcccctatcc

29521 atttattcat tcgacccaac tttcttttgt tccgttccaa gaattcgaaa aaggttttat

29581 accaatccga taagaatgaa atatcctcaa aactctccat tgatacgaca tgctattttt

29641 tccttttatt ccctttcagg atcagtcgcg gtcttccaaa gtttaccaag gttacggacg

29701 aattcgtcac ttcatccaaa tgtgtaaaag attctagccg cacttaaaag ccgagtactc

29761 taccgttgag ttagcaaccc gaaaaaaaca aacatagatt aaacaaaacc tcaacaaagg

29821 gtgtatagat acaatcaaat tcaattaaat tgatttgaaa caaagataaa aaatattata

29881 caaactaatc aaacccttga gttagcaaat ctaaaaaaaa acagattgga taatggattc

29941 aaaacataca aatgaagtga tttgatgaaa atacaattag attaaaatac aagaaatttt

30001 cagataaaat aaaagaaaaa aaagatacag cattgtgaaa atggatagag catctcttat

30061 gttatctagc tttctttcaa tcaaaaaaac ctcttgtatc aaagaaaaca ttatttgaat

30121 aagataaagt aacaaaacta tgggacaaaa ataaatagac ccggatcgct tatcacgatg

30181 aattatattt gttcaataca ctgttgtcaa tataaatgtt gagaaaagaa tacattggaa

30241 aagagaaatt gcatgaaaga aaatcctttt tgaaatcctt tgaatttcaa tttaacaatg

30301 aaatacaata atattcaaaa ttgtcttgga ttgacattac atatctaatc tacagagata

30361 gaaaaagtct aaatcgaaaa aaggaagaat tcaaaaaaaa tatcggattt ttgagtccct

30421 aatgtatttc atcagcagaa actctgataa ataaatatga atacggaaga aaaaaagaaa

30481 aaggggggca agtagaaaaa agttagacaa agttatatac aagtcgctac ccccctggta

30541 tttctttaat tgaatttcat ttaattaggc gtaagttcct taaaaacttc ggctttcttt

30601 aaaagattct gaacagttcc cgtgggttga gcaccccttt caaggaaata tagaataaga

30661 ggaacattta aaaaagtttg attcttcatt ggatcataaa agcccaccct tctaagatct

30721 tttccttctc ttcgggatcg aacatcaatt gcgacgattc gatagatggc tcattgggat

30781 agatgtagat gaacaatacc ccccctagaa ccgtatagga agttttctcc tcgtacagct

30841 cgcgaaaaat gatttgattc gaagttttgt ctatatatgc aattctaata aattaaatga

30901 caaatgcgct cataaactaa attagacttt gatttcagtc tttttttctt cctgaaaatc

30961 aaaaataaac cattcgtact cataactcaa gttggataac tctcaaatag ttcaaaggaa

31021 aaatctttag tcaatttcat ttattgagtc gtctttactc ccttttgttt gtctcgttta

31081 aaccatttca aattctattt ggattcttta gtctgatcca gttattgaga cgattgagac

31141 aattaaaagg gtgtttcctt gttcttagat cctttttttt ttaatcattg ggtttagaca

31201 ttacttcggt gtttcttaat tctttcaaaa taaaatggca gcaacatacc cccttttgat

31261 ttctttcaat caaaaaatcc tatggacagt cgattcccgc gtgatacact ttgaatcaaa

31321 aattggaatc aatttcaaca agttttgctt ttgacttgga aacttgctcg aattggatcc

31381 tttcgattcc tatatatgtt cgatatattt acgaagttgt tcctccaatt gattggcatt

31441 aaccctagat ccttgccccc gagaaatgaa ttaatacttt ctattcgagc tccagcgtgc

31501 actatttaca acccaacaaa aaacgaaggg ttcgggtgta acagaacaaa caatgtcgag

31561 ccaagagcac ccttattcct agacaaatca aaaatggtgg atgtaaaaat ccacaccgga

31621 tcgtgtcctt caagtcgcac gttgctttct accacatcgt ttcaaacgaa gttttaccat

31681 aacattcctc taattaattt ggaaccggta tgaaattgat tcaattatgg aatcatgaat

31741 agtcactggt tcagctgtcc atagacatcg taatctagac ttttcttcta tatatgaata

31801 tattggaacg atttttctga aaaagtctta gcaagacatc atttttaaca tacataaata

31861 atatatagat ccgacaaaaa aatagaaata gagaaacgaa taactttttg aatctgcatc

31921 ttcaagtgac tcaataggat agattaaacg atttcttact ttttcaaagt ttccacgtac

31981 aagtaatctt tctaattgaa attgaaaagg tttcctattt ctacatcatt attattatta

32041 aatggaattt gaagaaaatg ggacaataag catcaccttt gatcttcata ggaggatcgg

32101 tttttctttt tgaattgact catcactgat tgaatttcaa atagaaatgt gaaagggaag

32161 gttcttcgta tctatcagat agactgaaca attgtcactc ttatagatat tctagattat

32221 cgaatatcta taattttagt ttaaataatt taaggattac aaatcttttt cacaaagaac

32281 caaaaatttt cttgtcattg aaatagaatg atacgtaaca tttatatata tatattatac

32341 gattgatatg gatttggttt aaatggggtt gaggaatatt gactcttgtg agaaagtgaa

32401 tacaaaggga taggataaat caccaccgcc tcaagcctta aatagataat agatttccca

32461 tttttcattc gagtcaaaca cgaaatacct aaatcaaatt agattcaaag aaaaaacatc

32521 agctccctaa catattttta tataatatgg aacttgatca tttattaatg aaattgagaa

32581 caaacacaga tatgaaaatg aatatggatt aactcctacc cactccctta tttctttcga

32641 taggaataat ggagcataaa aaatggactg cgctgggacg gaaggattcg aacctccgaa

32701 tagcgggacc aaaacccgtt gccttaccgc ttggctacgc cccatttcaa tttctaatcg

32761 acactaagaa acaataatat tggtattgct tgtttgtcaa ctccaatcca aatatctata

32821 gatctataga atcgattggt actaggattt tgatacatat agatatagaa ttcaacttaa

32881 tttattgatc attacataga attcaattaa gatattgtat gaaagtatga tttcttctat

32941 cctcctttga gaattggagg atttttggtt gggtggattc aaagaaaaag aaaggttttt

33001 gtctacctta ctgactttct ttttccttat atcaaaaacg caatcaaaat gcaattatct

33061 ccaataacaa aatgtctgtt atgcttaata tcgttagttt gatctgtatt tgtattaatt

33121 ctcccctttt ttcgagtcat tttttcttcg ggaaattgcc cgaagcctat gcttttttga

33181 atccaatcgt ggattttatg ccagtcatac ctttgttttt ttttctctta gcttttgttt

33241 ggcaagctgc tgtaagtttt cgatgagatc ctgaataata atatcctaga aaatgtatga

33301 tttcctcgat aaaaaaattc taacaattga aaaaaaaaaa aataagataa gttttagagt

33361 atgaaccctc gattcacacg ctgaaattcg tgtatagtcg acaaaaccca ggcttaccct

33421 catttcctca tttttgaccc cctttccagt gaaagaccct aaccctatta gggtctccca

33481 caatataata cctaatttgg atataaacca aaatttgggt taatgaaaaa caaatgaatt

33541 tgtgacaatg catttctaga aaaacctcat ttctttagga tatgtggtag aaaaatagaa

33601 gatctattct cttttttttt tcaaaaaaac catcttggag attgtgtaat gcttactctg

33661 aaactcttcg tttataccgt agtgatattt tttgtgtctc tctttatctt tggattccta

33721 tctaatgatc cagggcgaaa tcctggacgt gaagaataaa atacaggggt ttttccttgg

33781 ttttagtgaa tttgaaaatt ttcttaggat tttatctatt ctacacgttt cactaagatt

33841 ttcaaaattt gaaaaaaacc aaataaattc atcaacggaa agagagggat tcgaaccctc

33901 ggtacgaata actcgtacaa cggattagca atccgacgct ttagtccact cagccatctc

33961 tcccaattga aaaagataat tactacatta cacataatgt aaagaacaca taatgtaaag

34021 aatctttctt ctttctctat tctattatat atatagagat ccacaaatcg ggaatttcct

34081 ttatctaaaa gatgggctca accgcccgaa caatcgaact aacaaaaaat cagcaagacc

34141 catttgtgtt gattttgttc gaaaggccct tcttattctc atggcccggc ccggtcagta

34201 cctagccggg ctcttttttt tgttccaacg aattctaatg attcatctga tagctgattt

34261 gaaaacaaaa agactttttt gattatatta ttatattcaa ttgaatattt tatattcaag

34321 caacaagaac aaagactatt tacattcttt ttcttgttat attttaccga actcaaaaag

34381 aaactatcga ttcttccaca attttaattt tgatctcccc gcaaaaaagt gcaacgttct

34441 cattttgatg attctttgat gatcctatct tgatcatgct caacgatctt gttcgacaaa

34501 agatccattt gtatacaata atcatattgt agcgggtata gtttagtggt aaaagtgtga

34561 ttcgttcgat taatcgttaa tagttaaagg gtctttcggt tttattcata ttccgaccaa

34621 aaactttatt tcttaaaaag attgaatcct ttacctctca atgagaaatt cgagaaaaaa

34681 aatacgaatt ctcgtgattt gtatccatga gtcacttata aattgaaaag ttggattatc

34741 aaattgcgaa acataatttt tgacttggac caaaaattcc aattgaataa gtatgagtaa

34801 aggatccatg gcagaagata gaaaggcaat ttcgaatcgt aactaaatct tccattttta

34861 tttctaaaga aataaattgg agcaaaatgg ctattcaacg acgactttgg tttactagag

34921 acatcgacat attgttttag ctcggtggaa acaaaatcct tttccttagg atcctctcaa

34981 atagaaatag agaacgaagt aactagaaag attgttagaa tcaccttctt ctagaaggat

35041 catctagaaa gcgattcatt ttgtttgtat tcaaacaaaa agctgacgta ggtgttatgg

35101 gtatcgttca ttttgttcac atcttagatc tatgaattta ctcatattcc ataaaggagc

35161 cgaatgaaac caaagtttca tgttcggttt tgaattagag acgttcaaag cactgaattg

35221 agcgtcgact ataaccccta gccttccaag ctaacgatgc gggttcgatt cccgctaccc

35281 gcttatttcc ttttttctaa ctattctatt cgaattctat tctagaatct agaaaatcta

35341 ttttaattac gattagtgaa tcattgaata tacaattcca aaaatgatct cacatataat

35401 cctatttttt ttgttttcaa ttcaataaaa atcaaaatac gaaaaaatcg gaatgaacgg

35461 cgtccattgt ctaatggata ggacagaggt cttctaaacc tttggtatag gttcaaatcc

35521 tattggacgc aatttatttc catctatttt tgatttctaa agactttttg cataattgga

35581 atccgagaca cttgagtcct ttttgaaaag aaaagtgaac agtttgttta tgcttgttcc

35641 tgaagtagaa accgatccat ttgttcctga atagcttctt tcaaaagggc ttccgcttct

35701 tcagtaaata tcttagtaga agatatgatt tcttggaact gaggtttatt actttttaag

35761 taagtacgta attcaacaat aaatttcctt acctgtccaa tttctaatga atcaagataa

35821 ccatttgttc cggtataaat agtcattatc tgttcttcta ccgcaagagg ggcggcttgg

35881 gattgtttaa gcaattcacg caatcgttga cctcttgcca attgattctg agtagcttta

35941 tcaagatcgg aagcaaattg tgcaaaggct tctaattctg caaattgcgc caattccaat

36001 tttaatttac cagctacttg tttcatggct ttaatttgag ccgcagaccc tactctggaa

36061 acggagatac ccacattaat ggcgggtcta attccagcat tgaatagatc ggcagataaa

36121 aatatttgtc catcagtaat ggaaattaca ttagtaggaa tataagccga aacatctccc

36181 gattgggttt caactattgg taaggcggtc atacttcctt cgcctaaact agaacttgac

36241 ttagcggctc tttccaaaag gcgtgaatgc aaataaaaaa catcccctgg ataagcttcg

36301 cgaccgggtg gtcttcgtaa tagaagagac atttggcgat aagcttgggc ttgtttggag

36361 ggatcatcat aaatgattaa agtgtgttgt ttacggtaca taaaaaattc agccaaagcg

36421 gctcctgtat aaggagcgag atattgtaat gtagcggggg aatccgccat ttcggctacc

36481 acaatagtgt attccatcgc ccccctttcc tgtaaagtcg ttactacctg agccaccgaa

36541 gatgcttttt gcccaatagc tacataaaca catattacat tttgaccttg ttgattgaga

36601 atcgtatctg tggctactgc tgttttaccg gtctgtctgt ctccaataat taattctcgc

36661 tgaccacgtc ctatagggat catcgaatca atagcaataa gcccggtttg aagaggctcg

36721 tatacggaac gccgggaaat aatacccgga gcgggagatt caattaatcg cgattcagaa

36781 gccgaaattt cccccctacc atcaataggt ttagccaggg catttataac acggcccaaa

36841 taagcctcac tcacgggtat ctgagcaatt cttcctgttg cttttacaga acttccttct

36901 tgtatcatca aaccatcacc cattaataca acaccaacat tatttgattc caaattcaga

36961 gcaataccta ttgtaccttc ttcaaattcg actaattcac ccgccattac ttcatcaaga

37021 ccataaatac gagcaatacc gtcgcctact tgaagtacgg taccggtatt tacaatctta

37081 acttctctat tatattgttc aatacgttca cggagaatat tactaatttc gtcggcttga

37141 atggttacca tgagtttttc ttaatttaat tattttttga aaagaaaaaa ataatgcctt

37201 ggagtagata atactaaata aagtgaaagg agaaaggcta atcagttctt tctttcattg

37261 ctcccaacag gccaatattg gcgctaatgg tacgtaaatg taactcatta ttcaaacaac

37321 tattcagagt ttctatagct ccttgtaagg cttgttggaa aacccgttgt cggacttgat

37381 taattgccct ttgctgttca aactgaatcg tttcattttt gtaattttct aattgttcca

37441 aagtgttata agttgaatta atcaaattcc atttttcttg ttctatctca gagtatccat

37501 tcacccgaaa ctgatctgcc tccatttcta ctttccgtaa gcgggcccgg gctttttcca

37561 tctgttcaat ggccccccca cgcagttctt ctgaatttcg aatagtattc aagatcctct

37621 gttttcgatt atctaataaa tcacttaatg aaagtagatt atctttccgt tcattttaaa

37681 acttccataa tcccttcccg aaccaaacat gaatctttcg attcatttgg ctctcgcgct

37741 caattactta gggtcaattc tcatagcttt ttatgaatgt aatgagccta tcctcttcat

37801 attaaaaaaa aatgaaaaaa cgaatctaat gcaaaacaaa aatacttaga ggactcttct

37861 gacaaaatca aaaatatcta attgtcagca aagttgtttc tttttttttt cttcagttca

37921 aatccaaaaa ttatacaata aggcataggt cgtcgattca gcattggata aaagggtaaa

37981 atacccgagc ggttccattt tttttatcga gatgagtgtt ctatatcgag aaagtattcc

38041 ttttaaatga aaaccacctc tatattaaca tagcggtaga aagagtacca tgctgcgtct

38101 agacttcaaa cgatttgatt taaccatctt aacagcccca cgttattggt tgctagagaa

38161 tcaaagtgga tttaccaatt aatcacgaaa tgctgtggtt cttacatata atttcttaag

38221 aagtaattcg cgtgatcatg cacccatcca ggggatacag ctgattggat acagcctatt

38281 cttgaaataa acaacccaca cacactccct ttccaaaaaa gatcaataca ccaatcacta

38341 cacttagatt tattggattt gttgctaaaa tatcggtatt aaatccgaaa ctcccggcag

38401 atggccagtg gaccaaagaa acgaaagaat cggttacatt tttcatataa tctcctctta

38461 tagatagact aaaaaatcga cgagagttct ttttctacca ctttgcccct ctttttgatt

38521 tatgcttttt atgaggtgtt gcaataatga aggggtagtt catactttaa ctagaagatt

38581 ttttgactcg atttcataaa aaaaaagagt ttcccttgga ctacgaacgg aaaggatgaa

38641 cgcgagtcgg catactaatt ccgcacctgc gaatcagcct ttcccgtagg ttattttctc

38701 aacgaataag gaattgtcgg agggaaatcg tgatctaatt tgaaaaagca aacgacaagc

38761 ccaaggcaat aaaataggaa aatttgtatt tttcctattt cgaagattaa acaaaaggat

38821 tcgcaaataa aagtgctagt gctacaacca atccataaat cgttaaagct tccataaaag

38881 ctaaactaag caatagagta cctcgtattt ttccctccgc ctcgggctgt ctcgcgatac

38941 cctctacggc ttgacccgca gcagtccctt gaccaactcc aggtccaata gaagcaagcc

39001 ctacagccaa tccagcagca ataacggaag cggcagaaat cagtggattc atgataaatt

39061 cctcatacca acaaaaagaa atggttaatg atacaatcaa ccaatgaact aggcaggact

39121 tccatcgata ggattcatcc agtggaaata actaagaact tagaatgtaa gtaagaatat

39181 tgttgaatca tcagaactac ttcgatatct cttttttgtt tctagttcta tccacagagt

39241 ttttgtgact ccgtagaact ttatttcatg atttcgaact gttatttcaa ttcttccatc

39301 ttttctggat ttcatctctt ttttcagcca atttacagcc acaacgatat gaaaggactt

39361 ctagtagaac tcacacacag gagtagggca actaaaatat atctttagtt catatatata

39421 acgagtcaat atctaatatc acatatacat gtctttcttc cataacgtaa accattagat

39481 tcaatcggat tcgacaatca atccactttt ttaggaatcc cgcgtaaatt cttttctcac

39541 ttgcgctttg tttatcggag agtacaatct aaatgggtaa attcaattga ttagcgcgaa

39601 ttattgcata gaaatttgat tatttcaaat ttgattaatc taagttcatg caatttctta

39661 taattttctt ttggtcaatt gttgaataaa atcaactgta aagtagaacc ccccttcttt

39721 ttagttaatc acatgtcgta aaatagatct tattcaaatt atgatttgaa taagaatatt

39781 ggctgaccaa tataatagaa agtgaatatt cgtcaaggaa agataggata ttaagtgtac

39841 aaaaagataa ttttaggaaa aagatctttt agatctcttt cctttttttt tttttttaca

39901 actctataat atcgtaattt ttgaattttt tgttcctatt gctttctatc gtatatagag

39961 tcttgtatat ttctattcgt taagtcaatc atcttgagcc atgcatacat tactttgcgc

40021 taagcgaaaa aaacgatttc aatgatgacc ctccatggat tcacctatat aagccgcggc

40081 taacgttgca aaaataagag cttgaatacc acttgtaaat aatccaagga acatgacggg

40141 gataggaatc accgaaggta ctaaagaaac aagaacaaca actactaatt catccgctaa

40201 gatattcccg aaaagccgaa aactaagcga taacggcttt gtaaaatctt ctaagatgtt

40261 aatgggtaaa aggattgggg ttggttgaat atatttcccg aaataactta atcccttttt

40321 gctaagaccc gcatagaaat atgccgctga cgtgagtaaa gccaaagcaa cagtagtatt

40381 tatatcattc gtaggtgcgg ctaactctcc atgaggtaat tctatgattt tccaaggtaa

40441 aagggctcct gaccaattcg aaacaaaaat aaatagaaac atagttccaa taaaaggaac

40501 ccaagggcca tattcttctc caatttgggt tttactcaca tctcgaatga attcaagaac

40561 atattcgaag aaattctgac ctccggtcgg gatggtttgt gggttccgaa cagctatagt

40621 agctgaacct aataagatgg caattacaac ccaagaagta ataagtactt ggccgtgtac

40681 ttggaaaccc cctatttgcc aatagaaatg ttggcctact tccacaccgg atatatcata

40741 taatcctttt agtgtgttgc tggaacatga tatcacattc atattgccct ctgaaaaaaa

40801 tcgaacttaa aacaaaatta ttttgattca accatctctt tctctttgtc tactttaatc

40861 ggatattttg aataccaact agtattttga atactaacta atcacataat atccccaggt

40921 atttttatct atttgagaaa attcataaat aatacccgat tccataaatc tttcgaggaa

40981 aagtgatttc gcttattagt aatcattatt aatcaaggat ttcttatata gctagaatgg

41041 ccctcactaa ttgcgaatac taatttatta agaattaatc ggattgaaga tatagcgtca

41101 tcgttcgctg gaatcgaaat atctgcaaga tcggggtcac aatttgtatc gattaaacaa

41161 atcgttggaa ttcccaaagt gatacactct cgcagggccg tatattcttc atgctgatcc

41221 acgatgatta caatatcggg taaccctgtc atatatttaa tcccgcccag atatgtttgt

41281 aagcgatata attgtctttt caccacagcc gcatctcttt tcggaagacg gttgagtctt

41341 cccgtttttt gttccatcct caagtccctg aacttatgaa gtctcgtttc tgtagtggac

41401 caatttgtta acatcccgcc gagccatttt ttattaacac aatgacaccg ggcctttatt

41461 gcagcccggg ctactgaatc agctgctttt tttttggtac caacaatcaa gaattgtttt

41521 cccctgcttg ctgcatcaaa aaccaaatcg caggcttccg ataaaaaacg agcagtttta

41581 gtcagatttg taatatgaat acctttacgc tttgcagaga tataaggtgc cattttagga

41641 ttccatttcc tagtaccgtg gccaaaatga actcctgcct ccagcatctc gtccaagttg

41701 atgttccaat atcttcttgt catttctccc cacacccccc ccttttcaaa aaacaaagag

41761 acgaggaccc ccgaactgaa ataaaaaatt gttccgatgg aaccttctct tctaccgtag

41821 attggctgta gctagacgaa taagccatca atgagtcttt tctattgctt actattttga

41881 ttaccgaatc aaatggctgc accaaataca tatactgata gtcaaaaaga tgaatctgct

41941 cttaggaatc attaaatcct ataaatgatt gttctggtct atcatccaaa ttatttgaaa

42001 gaaaagaatc aaataatttt ctatggtgaa acaaaatatc tctcatttcc ccttcaaata

42061 gatttttttt ttttgtttcc atttccaagg ggctcctgtt atgttgtttt gaagggggca

42121 cgaatccttt caatccggta ccgacgggta tcataccccc caaaacaaca ttctctttca

42181 ggcctttcaa ccaatcgatt cgaccccgga gagctgcttt tgctaaaact cgagcagttt

42241 cttgaaaact cgcctcagaa atgaaacttt gagtattgag agatgctctt gttattccca

42301 ataaaagggc tcgataacaa accgcttctt ccaacgcacg ccccattcgt tccgcacgca

42361 ccaatccaat tagttctccc ggcgaaaaaa cattagacat tccatcttct gaaaccaaga

42421 cccttgatgt tatttgacgt acaataattt ctatatgcct attatgaatc tgcaccccct

42481 gggatcgata aaccttttgg atcttattaa ccaaagagat acgactttgt actatagtta

42541 gctcagcacc aatcaagaat gcccacggca ttccaagaat tcttgttatg cgttcgttcc

42601 aaccctcaat cctcttgtct agattcatcg atattgaatc aattgaacgc acttctaaca

42661 cctgttctac ttttggaagc ccttgggtta tatcaccgga tctcgatttt tcatatataa

42721 atgtaattaa agtatctcct tcgtacagga tttccccata atggccatga acagttgctc

42781 ccggagtggc caaatacgtc ttagctgatc gtattactac agagtcaact tgaacaagta

42841 taacttgacc cgattttagg tgtggtgcgc tttttgatat atatatattt tcacaaataa

42901 actgcccaag actcattatt gtagatgttt cttgacaata attgggatgg aaaaaatacc

42961 aattcaaatt gcaaataatg ttactgcatg gatcggggtt ataaattttc tccttttcat

43021 cgattaaata atatttcaat ttaattactt gaaaggtctg tttaaaattg tcaagttgca

43081 aatagttctt taccaagatc tgattatgag ttattaaatg gtaaaatgaa taaacattcg

43141 caattcgaat tagagggact gttcctaaag gccccagcga attctgaatt ggaattacag

43201 gatcttttgt aatgttaatt gattctttta tcccattgtg tatcacattg tgattgtgat

43261 attttacatc gttgaatgga cccattcgaa aacaattgga tgatgacaaa attatcaacg

43321 attggaatcc cgcatttcta ttcaacaacg tatgaatagt tcgtttattt tgattaaggg

43381 gttgttgaat tattgccttg gaataaatcg aagaaaacgg atttattttt gtgcaatctg

43441 atccattatc cgagaacaat cctgagcccg agggatcatt cctttttccg atatatgaaa

43501 aagtggattt cagcaagtcg attcttagta aatgtcgaat caaaccattt acccttattt

43561 caacaaagga agcacgggct tcttgactag aagaactttt tttgtcttgg tcccaattca

43621 atactaaaca agtccgaact aattgaatat ttgtatcaga aattcctcga atcggtttac

43681 catttccata aaggatataa ttgacaaccc gaagtttcac attatccctt tcctgcaaca

43741 gatccggagg gaaaagtgtt cctaaagtta taccgtccgt tatttcatat gtgacgacag

43801 gccgaaccaa aacaaaatac tttttcttac taggtgtgat ccgttgaaca tagatccaat

43861 tttcccgttt tttcgattcc ttagaatttc gttttcctgt tcttagtggt atcaaaacgc

43921 cgctatgtcg ggatatctta tctgtctctc cgggaaaatg gatatctcca gaaaagattt

43981 taagttcaat tctttttttt tttctctcca ctcggaccaa cccggctacg cggcttctca

44041 tatttaaagt aatttgtgta tctaccccaa tgatactatt gttccgtacc attatggaag

44101 aagatccggg taatatatgc acttcctcgg gaatgaaaaa aaaccgatcg actttcattt

44161 ggtattttgg actaaattcc ttgcctcctc gatactcaat caaatcctct tttttgacga

44221 ttgaatgcat ttctagagtc ccatatttag taatgcccga actctttctt ctgtatctag

44281 gatcgtccaa ataagcaaga atactatttc tgcggaaaat gccgttgatg gggatttcaa

44341 tcaagatatc tgaagggggc gttagcccgt tctcgcgttc ttgaatcgat tggagtggga

44401 tgatgaatct atttcttcgc ctctttgaga ataaatcaga attatggtag agaagggtcg

44461 gatctacgag attacaacaa ccagtacata taattcgact aaggtctgaa taatcaggaa

44521 tcctatcttc ttttttaccc gaaaaatctg aagtaaagaa tttttctctc aactgatcat

44581 ccgtccctga aaggttaaaa ctcttgacag aacgagaatt cgcactcatt tgatcttgat

44641 ccttgtggat cgaaagtgaa actagactgg atccgcgtgg ctctcctaat aatatccata

44701 aatgacttgt ttttggtaat agatgaacac tgccgtatgt aaattcgggt gcatgatata

44761 catcggtgct ccagtgcatt tctccgtctg agtcagaata aatatgtttt cgaatcttct

44821 ctttaaaatt aaaagtggat gttcccgcgc gaatctcggc aatcacttgt tctgattcga

44881 catattgatc gttttgaact aaaagaaaac tttggggtgg aatatttaca ttatgtcgaa

44941 tatcttcact ctcaatagtt acatacaagt ttatagaaca gagaagggcg ggatgcccat

45001 ggcgtgtacg tgtcggatga actaaatcct cattgaattt gatttttcca ttagaagggg

45061 ctcgcacgtg ttctgcagta cccccggtga atactccacc ggtatgaaaa gttcttaatg

45121 ttaattgagt gcccggttct ccaattgatt ggcctgcaat aatacctacc gcttctccca

45181 attcaaccag gtcgccatga gtagagctcc ggccataaca taatcgacag atccaagatg

45241 cactcctaca agtaaagggc gttcgaatag ctattggttg tgctcgaaag gttatgaatc

45301 gatttacaag tccaatccca atgtcttgat ttctagtggc aatacagcgt gtgcccatat

45361 atatatcatc ggctaataca cgaccaatta atgtttggat aaaaatcctt tctggcatca

45421 taccattctg aggactcaca gaaatgccac gggcggttcc acaatctatt cgacgtacaa

45481 caatgtgttg aactacttca acaagtctgc gcgtgagata tccagcatct gatgttcgta

45541 cagcagtatc tacaacccct ttacgggctc catagcaaga aattatatat tctgttaaag

45601 aaagcccttc gcgtaaattg ctttgaatgg gtaaatcaat catttgtcct tgagggtccg

45661 acattaatcc cctcatacct actaattgat gtacctggga tgcatttcct ctagctcccg

45721 aaaaagacat tatatgaact ggattaaggg ggtcagtcat cctaaaatta ggattcattt

45781 cttggcgcaa atattcactt gtggcatacc atatttcaat ggattggcgt aatttttcta

45841 ccgcgtgtac attcccataa tggtggtgtt tttccaaaat caaactttgt tgttcggcat

45901 cttgaactag ccatttcttc gagggtattg ttaaaagatc atcaattcct aatgaaatgg

45961 atgtagcagt agcttgttga aaacccaaag tctttacttg atccaggatg tgtgatgtat

46021 atgccattcc gaagtgatct attaatctac taataagtcg tttcatggca gttccatcta

46081 tcactttatt gtgaaatacc agattggccc gttctgccat aagtacctcc atattctgct

46141 gagtatgatt tgacaatggg tttgagtcag tggttggaaa acttcccctt ctcgatcttg

46201 attcgcgtag aaattccgga actacgggcc tggttgaagt gaagagatct gaattcctat

46261 tggtatcgta gaattcctta gcctagttag gtaccatatg aacaagcccg ggaaaacccc

46321 tgtatggctt cttcgatttc tcgataaaga gaaatatgac caacagtggt tcgaatgtat

46381 agaaaaagaa tttctttttt tatacttctt agtattaaat agtgtccata aatctcataa

46441 taattaccca aagattcata gtgaacttcg atgggagttt ctcttgaagc aataacccgt

46501 tggtctagtc gccaccgaag ccacaaagga ctatctaaat tgattcgttt ctgccgataa

46561 gccccaattg catcatagga attagaaaaa aaaggttctt tcgtatactt atatttatta

46621 ttgtcacttc ttttattttg atagtttttg agattccatg gattatacct atttatacaa

46681 atacctcgac gattcccgct cgttaataca tagagtccaa taagcatatc ttgagttggt

46741 acggaaatgg gatctccaat agctggagac aaaagattca tatgagaaaa cataagtaaa

46801 cgggcctctg cttgagcctc caaagataag ggtacatgaa cagccatttg atccccgtca

46861 aaatctgcat tgaatccctt acaaactaat ggatgtaaac aaatagcgcg tccctccact

46921 aaaatgggct ggaatgcttg tatgcccagt ttatgcagag tgggtgctct attcagcaat

46981 acaggatgcc cctgcataac ttcctgcagt atttcccata caatcggttc tttttctcga

47041 attttactct tagcaactcc tatgtttgaa gcaagatgtt gtctaattag accacgaatt

47101 acaaatgtct ggaaaagctc tattgctatt tcgcgcggca atccacatcg atgtaatgaa

47161 agtgaaggac ctacgacaat gacggaacgc cctgaataat caacccgttt cccaaggaga

47221 gtctcacgaa atcttccctc tttgccttca attacatctg aaaatgactt gtaagcttta

47281 ttatgaccgt ccctcgttgg ttgaccccgg attccattat caagaagtgt atccacagct

47341 tcttgtacta atttctcctg acacattact aattctcccg gcgtagatct acttgttgtt

47401 aatagatcgg taagagtatt gttccgatag ataactcttc tatagagttc attaatatcc

47461 gagctcatta atttaccccc atctatctga atgatgggtc ttaactcagg aggaagaact

47521 ggtaatagac acaaaaccat ccattctggt tctatatttg ttcgaagaaa atgcttagcc

47581 aattccatgc gtctaaccaa aaaatccttt cttcttccaa cctttcgatc ttcccattca

47641 ttccccgtgg gcccttcttc ccctaattct ttccattcta ccaaagaatt atgtagaata

47701 gtgcgtaaat ctaggtcggc tagttgttct cgaatagcac ttgcgccggt cgagatttct

47761 cgatttcgaa atgtatcgaa gccctgggta gtaaaaaaaa gcgggatgct gtatttccaa

47821 gattggattt catattcgaa taaacctcgt aatcgcaaga aagtgggttt tttcgttatg

47881 ggcctagcaa aagaaaaatt gagataggat cctataggat cgcccccctt caaaatcgga

47941 cgtgaaagtt tcctttcatc cggctcaagt aggtacacca attcaagaaa ggagttcccc

48001 ctttcaaatt atagaaaaac tcaaaacaaa aagatctact ctttactcaa gttaccagtg

48061 aagactaagc aaaatttcat tgattccgcc ttctttgtat tttttattta ttcatattat

48121 ttcgatttga ttaattcgtg ctattcaatt acgacagaaa tgaaatgtga aattcttgag

48181 tagtctactt cccttcgaat gatgaatccc ttaattctta attagaatga aagtgaaatt

48241 aaaataaaaa aataaaggag taccttgtaa ttcataaggg atttacttgt ctatgtattg

48301 ttccattcga tctgttaggt ccctacttcg cctcgacggt tatgccacga tgcccttatt

48361 aaagcctata cgcgatggat agactcctgt aaccacgaca tatttgcttg cttgaacata

48421 aaatttcttt ccaaaagaaa gtaaatggtt aatctcacaa aacaaataaa tattttttac

48481 gaggtacaaa tcaaattttt tgtatttgtt ccaaaatcga ccatagatca atcccccttt

48541 tgatttggga gtattgacta cacccctaag tctgagcttc atggtactct taccaagtca

48601 catgttagat ccagggcatc ccaattggat tgattaggat gacagtttct cattccgaat

48661 ctgtaaaata agaattttta tcaaatcaca catcgcagta tactaggcct tctaattctt

48721 taagaggttt atctaaaaga ttcgcaatat aactgggaag acgttttaaa taccacacgt

48781 gggttactgg gcatgcgagt ttgatgtagc ccatttgata tcttcgtatc cgagaatcaa

48841 caaattcgac tccgcattgt tcacaaaatt tcgggtcttc tttttcatct ccgattacgc

48901 gataatttcc acaagcacaa attccacttt tgataggccc aaaaattctt tcacaaaata

48961 atccaccttt ttccggttta ttggttttgt aatgaaaagt atagggtttt gtcacctctc

49021 caacgatctc cccattgggc aggattttag tagcccaagc gcttatttgt tgaggagaaa

49081 ctaagccaat tcggagctgt tggtgtttat accgatcgat catagaataa aaattcggat

49141 ttattctgat taagcttcct tcctattaat ctggaagttt ttctcagata caaggaaatg

49201 attcagttcc agagctaaag atcgtagttc tcgaacgagc aatcgaaagg attctggagc

49261 atcctcggga ttaggtataa ttcctccaat gatcgtagta ccaagtactt cttggcgcgc

49321 tctaatatga tcagatttat aagtaagcat ctcttgtaaa atatgagcaa caccaaatcc

49381 ttctagagcc caaacctcca tttctcctac tcgctgtccc ccctgttttg cccttccccg

49441 aaggggttgt tgtgtaacaa gcgcataatg tccactggaa cgtccatgga ttttatcatc

49501 aacttgatga attaatttca agatataagg ttttcctatt ataacgggtt gttcaaaagg

49561 actccccgcc cttccatcaa atattctact ttttcctgga tactcgggtt caaataccca

49621 tggattagct gtttgcttac tggcttcata taattcagaa aacactagtt ttctggaagc

49681 ttcttgttca tatctctcat caaaaggcgc tattcgatag tgtctgtcta gcaaaccccc

49741 tgctaacccg agcgaacatt caaatatctg tcctacattc attcgtgagg gtactcctaa

49801 tgggttgaaa accatatcaa taggtcttcc atcttgcaga taaggcatat cttgtctagg

49861 caaaattttt gaaatgatac ctttatttcc gtgtcttcca gctactttat cgcctacttt

49921 gatttcacgt ttctgtgaaa tatatacacg aatcatttct ggattataac tagaacctcc

49981 tcttttctgg atccacctca catcaataac ccggccccta ccacctatag ggagttttaa

50041 acaagtttct tttgaagtag atacctgaat gccgagtata gctcgtaaca atctatcttc

50101 gggggcatac gaggattctt tcaccatttg gggcgttaat ttacctacta aaatatcacc

50161 cgtctctacc caagatccca acatcacaat tccattttta tctaaattgc gaagtaaacg

50221 ggcttctaaa tgtggtattt cgttagtgat cctttcgggg ccttgacttg tcgcatgagt

50281 ctgaatctcg tatttccgta tgtgaaaaga agtataaata tcttcatata ccaaacgctc

50341 actaatgagt accgcatctt cagaattgta accttcccac ggcatataag ctactaatac

50401 attttttccc aaagcaagtt cgccaccaac cgtagcagca ccatctgcta aaatttgtcc

50461 ctttttaatg catttacccc gctgaacgcg ggttttttga tgcatacaag tatttttgtt

50521 ggaacgttga tacataacta atgaaatgct tagagtatct ccatcccccg agaaaaggat

50581 cttgtcagta tctatataaa tgatctttcc cccgcgttcg gctatagcaa gagcccctga

50641 atctagagct gcttgtcgtt ccaacccagt cccaacaata catttctcgg accgagaaag

50701 cggaactgcc tgacgttgca tattagaact cattaaagcc ctattcgcat cattatgttc

50761 aataaaagga atgagggaag ctccaataga aaaatattgg aagggaaaaa tacttcgaag

50821 atgaacccgt tcccatgcaa tagtcaggaa ttcttggcgg tatcgagctg gaacaacctg

50881 ttcttcctga atatcctgat tcaacgccaa agaatttcct gccgctaaca tatagtattc

50941 atctctgccg ggtgataaat aaagcagccg taaccctgtt gatctctccg aaattttata

51001 aaacgggctt tctagagatc cccaataacc catcctcgca tgaattgcta aggatccaat

51061 aagcccaaca ttgattccct ccgatgtgtc aattgggcaa atacgcccat agtgactagg

51121 atggatatct cgtatccgaa aactagcagt tcgccctgtt agtcctccag gacctaaata

51181 acttaatttt ctaccatgaa ctatttgtgt caacggatta gttcgatcta aaacttgaga

51241 taaagggtgt aaaccaaaaa aagattcata agtagttgtt aagggagttg aagttaccaa

51301 attttgagga gttggtatca atttatgccg aattgctcca catatagttc ctcgaaccac

51361 attttctaaa cgaaccagag acaatccgaa ttgatcttgt aaaagatccg ctacagaacg

51421 aatgcgctta tttttcaaat gattcatatc gtcaagtgta cccattccaa atttcagtcc

51481 aattaaatga tcagcggccg ccagtatgtc tcgtggtaac aaaaatgtat tgttcgaggg

51541 gatatcaagg ttcagccttc ggttcatatt tcgtcgacca atccttccta attcacatct

51601 ttgttgaaag aacttctttt gtaattcctt acataaggat tcagaaaata ccggatcccc

51661 gcctacacaa gcaaattgtt gataaaactc caaaatggcg ttttcttttg atccaatttt

51721 ttttctctcc ttatcattca gaaaagacaa aaaaatttca ggataataaa cattgtctag

51781 aatttctctt agattcgaac ccatagctga tgatagaact agaatagata ttttttgttt

51841 cctactaacg cgagcccata tccttgcttt tctatcaatc tctaattctg atcttcctcc

51901 ccaatctgat attatggtgc cggtatagac cgcaattccg ttatggtcca attctgaccg

51961 gtaataaata ccgggacttt gcagtatttg attgatcaca attctatata ttccatttac

52021 tatagaagtt cctagggaat tcattagagg aatgtttcca accaaaatag tttgttcttg

52081 catatcccta ctggttttcc aaattaatcc cgcggataca tataattcag aagaatatgt

52141 gagtgattca tatacagcat ttctttcctt tatcaacggt tctaccaatt gatatctttc

52201 caaaaataat tgaaattcga tttcatgatc tgtatcttca atttttggaa acttgtaaag

52261 ttcttctgtc aaaccccgat tgatgaacct acaaaatcct tcaaattgga tctgatgaaa

52321 tccaggtatt gtagacattg cctcatttgc atccccgagc attttgaatt tcccatttat

52381 caaaaaatcc cattatattt tatattatta agtacattct tcatcgaatt agatcgaggc

52441 ttctagtact gatggaattt ctattctgtt tactgaatca catgaaattt tagccaactc

52501 catatttgaa atgaaatgta tgaacgaaga aataaagata attttctact aaaattggaa

52561 gttgcgacag atcaaaattg aaaacagctg atagaacaat cctagaaaca gaattctgtc

52621 acttaggctt attaaggtca tagggttttg tatagaatcg caaaacaaaa aaaatgattc

52681 aaattagacc attattatga tattacatat tcgaatttga gaaaaagaaa aagattcggt

52741 atttgggaga caaagataaa aaataagata gaatccattt tttagcactt aaccgcctta

52801 gctggggatt tccttgttca aaaaacgatt caaagagaag agagatattt gtactttact

52861 tatttttgag tagaatacaa ttggaagtgt attgtataag aagggttgca tttattaaac

52921 acgtatcgag atagctagaa tatccgactt ctcttttatt gccggttcta ttcggaatag

52981 ccctggctgt gctctatcaa aagataattt ctattcaatg catgaagtag gatcatttta

53041 tgataattcc ctatcgacaa catatctaaa taaacatatc taaataatag atctctgtgt

53101 aacaatttat gttctggggt ttacatagac tcatatgttt tgttataatt gaagtggaga

53161 aaggtttttt gattgaaaaa atcaatactg aattggttcg gtctcaattt ttattttctt

53221 atgtcattag gaaaacacaa tttgaaattc aaatcccaga atcgttcatg aattcgaact

53281 aaggagtcaa tagttaatgg ttccaattta tagttaatgg ttccaatttt tcggctgaaa

53341 atacacaatg ctaaaaacat tctctcgctt tcctattgag caatcaaatg tgtattttca

53401 gatactaaat atgtattttc agatactata caataaattg aaggggtgga tcaaatacaa

53461 ccaaaagggg gtttggaaga aaagtatttt tttatcattt tggcggcatg gccgagtggt

53521 aaggcggggg actgcaaatc ctttttcccc agttcaaatc cgggtgtcgc ctgattacca

53581 aaaactcgaa atctcttctt cttttctgtt cttctgatag aactcgaaaa atgcttcctc

53641 cgtagaagca taggaaacgg gctgttgata ctttgtttga ttctaaatat gtggtctaaa

53701 ggttttctaa aagaaaagat tgtgaagccc cgttcgcatc taggattcaa ggaaatattc

53761 gaatctactg gtagaggggt tatcaagact tcatgattcc cttctattac taaggaagtg

53821 gctaatgact ggatcttgaa tcaaattgta gagcctggaa attaaattac taattttggg

53881 aggggtcgga agttgcttta tataagacga acttgcgaga atctctgagt gctcaggtat

53941 ccaatcaata ttagattgga tgaataattg acttttctag aaaagagagt aaaaagaaat

54001 gctttctttt cggtaacgcc tacccaagcc ccctgtttcg cagggataat cagaaaaggg

54061 ggtacgggtt cgatcaaatg gggaaataga gatctgtaat agatagtgat tgctcgtttt

54121 tagtgattcc cccccccctt ctgtttttac ttagaatgta aacaaaacaa aaaagaatag

54181 accttattat tcttattcct acatgttccc atttccttga gatgttacta tgtattttgc

54241 ttgtgttttt aatctttgga ttcgaaatca taatcgattt attggattgc tttctcaata

54301 gtgttcggtc agaacccctt tttgactctg cggcattgat tccactatta ttagtgagga

54361 ataatggaat aattcctttg tatttataga gataggggac ataatgcaca tggatatagt

54421 cagtctcgct tgggctgctt taatggtagt ctttacattt tccctttcac tcgtagtgtg

54481 gggaagaagt gggctctaga agtactacta attgagttca ggaatccaaa ataaaaccgt

54541 atcaattgtt ttatagatcg ttctgcaacg catttgaaac tatttcaaat caaaactctg

54601 aatttggaat ctcattggac tccaatcgag taatctatga tatgaatcat actctttcaa

54661 ttttcaatta aagagatatt tcatcgattc ccgcccttgt attgtatttc gaaaagaaag

54721 ggattcagat gattggaaat ttattccaac ctcaaattct tccaaaattt tctatttcaa

54781 tcaaggggaa tgggctctta ctatttttat agatggaaga tggatactga ggaagacgga

54841 cccccccttt tttatttctt tccctcttta ctcttcaaag aagaagtcgt tttgttacgt

54901 gtctacgcac tttctagaag aaatgatata gggatggtgg ttgtctaccg agagactagg

54961 caataaataa taagatcttg actggggcga gtcatgggca tttacccttt gttttctaat

55021 tttataaagg cgatttttgc tttctcgact tatttcatat cagggtttgg gcgttaaaaa

55081 taggcaaggt ttccccttac ttattaggta atagtatggt agaaagatgc atctttctac

55141 catactatct atctcgtaga aaactgctga ttatagtgcg ctcgtttcat ttaagttaag

55201 acgtaaaatg ggaatccttt tcatttgact tcgtcaattt ttgataagaa ctcagaagtc

55261 aagtttaatt caaatgaatt tgaaaattca tttgaattaa tcattttggc tgactgtttt

55321 tacgtaaatg ataagcagaa aggcggtagg aactagaatg aatagtgcag tagcaataaa

55381 tgcaagaata ttgacttcca taatctcatc ggttttttta cttcgcaata actcgggatt

55441 taatccccta gagatgataa atctttcggc tgtaaattca acgaatgaat tacctctcga

55501 tgatcttgaa tcggatcaat atcatgaata acaatatctg atctagcaaa tcaacccgtc

55561 gtcaagactt gaatagtata acataggaag ttcttttatc cataccgaat ccaaattttg

55621 attcctgact caatcaatcc aaaattcctt tatttctcat ctgtttcctt gttttttcta

55681 taacctgcct tgcgtcttcc ttggacaatc gtctgataaa ggatcatcag attgcctttc

55741 cacttcgatt aattacatag ttctaaacgt aaataaaagc caaatggaaa aaataggaaa

55801 gcaaaaagaa ataaagactc ctttttgctt tgggaatgtt tgctttggaa atgaaagtat

55861 gccccttgcc accctttact agttcataga agggaaaaaa tgaattgatg tatttatttg

55921 attttgatcc gtcgggactg gcggggctcg aacccgcagc ttccgccttg acagggcggt

55981 gctctaaccg attgaactac aatcccgggg aaaggaaata ggggatctcg cataaaattg

56041 gattcttttt tttatcttcg ttgggtcttt ctgaaggata gggggatttg taccatctca

56101 tgttagatta gcggattatt gggccgagct ggatttgaac cagcgtagac atattgccaa

56161 cgaatttaca gtccgtcccc attaaccgct cgggcatcga cccaggaaga atccatttta

56221 gccttattag taatccatga tcaacttcct ttcatagtac cctaccccca ggggaattcg

56281 aatccccgct gcctccttga aagagagatg tcctaaacca ctagacgatg ggggccaact

56341 tgaccaaccg ccctcatact atgatcatag tatgatcagt tttttgaaat tgtcaatata

56401 atggaatgat cagatccggg gatccttccg tttttcataa ttgtatagaa ttttttgctt

56461 cgtcattcat attcatgaat cgttcattag aatcgacatt ctctagataa atcgaatcta

56521 gtaagcggga gcaaaatcaa aaagttatca aaaatgttct ttaagacttt agttccaggg

56581 gggagtagaa tctcttcatg acatgattcg atcaaatgtc ttgaaattta ttttagatca

56641 aattagtaag tgtacgtgta tgttatgtat caattaagtg aattttgttt tcattaagga

56701 tcatctcaat aaaagaaatt agggccggtc ttgaaacaat tcattttacc ctagattttc

56761 taggaaaatc cattggatta ttcaaaaata agctactagt cactatgagt atactgtgta

56821 tatatatatg tatataatct atttatatat atatgtatat aatatatttg catatagaga

56881 ttttatctac atagtgactc gtcctggaat gaaataaaac aagccctttt aactcagtgg

56941 tagagtaacg ccatggtaag gcgtaagtca tcggttcaaa tccgataagg ggcttttttt

57001 gtagttggaa taaaaaagga actaaccgga taatccgtta tcattatact gagttagagt

57061 tgagttagag tatagtagtt ctagttaaaa aatggaacat ttgttcggaa aattttcatt

57121 attatgaata atcataagcc gcctcttgaa tcgccaaaga tccctatttt ccattatacc

57181 aagcaaatgc accggaaaga ttcgaaatca acaaaacaaa agaaaaagta agtggacctg

57241 acccatttaa ttagaactat atccgctatt ctgatattca aattagatag agatcgaatt

57301 tgaattagaa cagccggcca cctccccttt taaatttcat ttctttggac ttggaaagga

57361 aagaatttgt cgatatttcc gattcaatct tcttgttcct atctttcaag tcacaaaata

57421 agagccattt ccactattta gatgtatagc tatcagatca cgacttcatg taccaaacat

57481 ttctatattg ttgcatccga tattttggtt acgacagtgt aatggatgtg agaaagaaac

57541 tttcatttca agtcttctat ttttttttta ttgaatttaa cgaaaaaaaa ggtgaaaggg

57601 ggaggaaagt ctctttcttt ctaagagata agactcaata gaaaaatact ggaagggtct

57661 ttgttgcttt gacccgtggg aagatatact ctggaatttt tgatttatct gacaaaaaaa

57721 atgaaaaaaa gaaatttata aattatgtat atggaattgg aatcgtttag tatacataga

57781 aattcaaaca atctagaaat atctttcttt ttcgcaatct caagaacaag atctaagaat

57841 aacacgagtt aatcgaagaa gaggggggat agatctggga atcggttagt cgtagggggg

57901 gtcgcttgtt ccttgaaaag ttctttgaaa agattcatgt atctgattga tgagtcataa

57961 gaagacaatt catggttcat atacttagta acaaagaata atcaaattga gttcatggat

58021 ttcctcaggt cagtttgtgg gccaataaag gatttttatc ttcgaaaccc gtcggtaggg

58081 accgtgcaag agaaatcata gagaaatgat cgaatcttca gacgccccga aaatactatg

58141 aggtgctcgg aaatggttga agtagttgaa taggaggatc actatgacta tagcccttgg

58201 taaatttacc aaagacgaaa aggatttatt tgatattatg gatgactggt tacggaggga

58261 ccgtttcgtt tttgtaggct ggtccggcct attgctcttt ccttgtgcct atttcgcttt

58321 agggggttgg ttcacaggta caacctttgt aacttcatgg tatacccatg gcttagccag

58381 ttcctatttg gaaggctgta atttcttaac agccgcagtt tcgactcctg ctaatagttt

58441 agcgcattct ttattgttac tatggggtcc tgaagcacaa ggagatttta ctcgttggtg

58501 tcaattaggc ggtctgtgga cttttgttgc tctgcatggc gctttcgccc taataggttt

58561 catgttacgt caatttgagc ttgctcgatc tgttcaattg cgaccttata atgcaatcgc

58621 attctctggt ccaattgctg tttttgtttc tgtattcctg atttatccgc tgggacagtc

58681 tggttggttc tttgcaccta gttttggtgt agcagctata tttcgattca tcctattttt

58741 tcaaggattt cataattgga cgctgaaccc atttcatatg atgggagttg ccggcgtatt

58801 gggggctgct ttgctatgcg ctattcatgg tgctaccgta gaaaatactt tatttgaaga

58861 tggtgatggt gcaaatacat tccgtgcttt taacccaact caagccgaag aaacttattc

58921 catggtgaca gctaaccgct tttggtccca aatctttggg gttgcttttt ccaataaacg

58981 ttggttacat ttctttatgt tatttgtacc agtaaccggt ttatggatga gtgctcttgg

59041 agtagtcggt ttggccctga acctacgcgc ctatgacttc gtttctcagg aaattcgcgc

59101 agcggaagat ccggaatttg agactttcta caccaaaaat attctattaa acgaaggtat

59161 tcgtgcttgg atggcggctc aagatcagcc tcatgaaaac cttatattcc ctgaggaggt

59221 tctaccacgt ggaaacgctc tttaatggaa ctttatctgt agctggtcgt gaccaagaaa

59281 ccaccggttt tgcttggtgg gccggaaatg cccgacttat taatttatcc ggtaaactac

59341 taggagctca tgtagcccat gccggattaa tcgtattctg ggccggagca atgaacctat

59401 ttgaagtggc tcatttcgta ccagagaagc ctatgtatga acaaggatta attttacttc

59461 cccatctagc tactctaggt tggggggtag gccctggggg ggaagttata gatacctttc

59521 catactttgt atctggagta cttcatttaa tttcctctgc agtattgggt tttggcggta

59581 tttatcatgc acttctggga cccgagacac tggaagaatc ttttccattc ttcggttatg

59641 tgtggaaaga tagaaataaa atgaccacaa ttttaggtat tcacttaatc ttattgggtc

59701 taggtgcttt tcttctagtc ttcaaagctc tttattttgg gggcgtatat gatacttggg

59761 ctccgggagg gggagatgta agaaaaatta ccaacttgac ccttagccca agtatcatat

59821 ttggttattt actaaaatct ccctttgggg gagaagggtg gattgttagt gtggacgatt

59881 tagaagatat aatcggagga cacgtatggt taggttccat ttgtatactt ggtggaatct

59941 ggcatatctt aactaaaccc ttcgcgtggg ctcgacgcgc ccttgtatgg tctggggagg

60001 cttacttatc ttatagttta ggggctttag ccatcttcgg tttcactgct tgttgttttg

60061 tctggttcaa taatacggcc tatcctagcg agttttacgg tcctactgga ccagaagctt

60121 ctcaggctca agcatttact tttctagtta gagaccaacg tcttggggct aatgtaggat

60181 ccgctcaagg acctactggt ttaggtaaat acctaatgcg ctccccgacc ggagaagtca

60241 tttttggagg agaaactatg cgtttttggg atctgcgtgc tccttggtta gaacctttaa

60301 gaggtccaaa tggattggac ttgagtaggt tgaaaaaaga catacaacct tggcaagaac

60361 ggcgttccgc agaatatatg actcatgctc ctttaggttc tttaaattct gtgggtggcg

60421 tagctaccga gattaatgca gtcaattatg tctctcctag aagttggtta gctacctctc

60481 attttgttct aggattcttc ttcttcgtag gtcatttgtg gcacgcggga agggctcgtg

60541 cagctgcagc aggatttgaa aaaggaattg atcgtgattt tgaacctgtt cttttcatga

60601 cccctcttaa ttgagatgag acaggagatc cgatgcttga attgaagtag gaatcacttt

60661 gattccatca tacatattgg gatcaggtta tacttgaaaa gtattccttt tttttttttc

60721 aactcactaa tcaatttttc tattttttat ggcttggcta ggtgggatag ccgagccatt

60781 cccctttctt tatgatacca atctcggcaa aactaataga aacaaatcga ttcaatgagc

60841 aaactaaagg agagagaggg attcgaaccc tcgatagttc tttgttcaaa actataccgg

60901 ttttcaagac cggggctatc aaccactcag ccatctctcc gaaagacaat ttttatttta

60961 ttcctccgaa tagaacatgg ccataggggg ggataacact actatctgta gaaagatctc

61021 cggtgtgaat ctaccgatcg atctatccat atatagaatc cagcatgccc catttgtgaa

61081 ataaaaaata aaattccatt tccaccgact ccatgtatga atagagtggt caaaggggta

61141 gtaataagtc caattgattc gtggtaaact aaaatccctt gatggtgtat tttcttacaa

61201 tttttggctg atagagggat caaatggtat agtttatttg ttggtatctt ggaggattaa

61261 aaggatgact cttgttttcc aattggctgt ttttgcatta attgctactt catcaatctt

61321 attgattagc gtacctgtcg tatttgcttc tcccgacggt tggtcaagta acaaaaatgt

61381 tgtattttcc ggtacatcat tatggattgg gttagtcttt ctggtgggta tccttaattc

61441 tctcatctct tgaacctatt tgtcccagat ccaaaaccaa aacgaccccc caaaaaaaga

61501 aaatgaaaaa aaatgaaatt taaaattaga ggggggtcaa acttcttgga tgaaaaaaat

61561 acaattaatg agattaataa aatgattgga ataactctga agagagtctc cggaccgata

61621 ctgcataaat atgatacagg ttatcatata tgtgtggata tattgtgtat cacaaacaaa

61681 aatgcggata tggtcgaatg gtaaaatttc tctttgccaa ggagaagatg cgggttcgat

61741 tcccgctatc cgccaaagat aaagtaattt tttgatttta ataggctaaa gggttgggta

61801 tagttggtcg tgatagtata gtaagtctat cccccctccc cttcctactc cccccaaaag

61861 ggggtaagag ccaaacctca caattcgata taaaataaaa aaagatgttg cggagacagg

61921 atttgaaccc gtgacctcaa ggttatgagc cttgcgagct accaaactgc tctaccccgc

61981 gctgaagaga agaagtaaaa actaatagac aaagaaggat tgaatgtgcc cctctaccat

62041 atctgtacaa atagaatagt acatttatac agaatggtaa agaggccctt tatgatcatc

62101 gaccgtataa ataaaatgaa agattaatcc ttaccaactt gatcttgttg ctcctggcaa

62161 caaacatgca tgaaccattt cgcgaagtat gtgtccggat agtccaaagt ctcgatagtt

62221 agctctcggc cttccggtcg aaaaacaacg gcgatgaagg cgtgtaggtg cactatttcg

62281 tggtggggat tgtaactttc cataaatttg ccatttgtca ctcaatgatg gaactttact

62341 tatttctttt tttgaggatc gacgaatcaa atgatatttc tgttccaatt tttgcctctt

62401 cttctccctc tgaatcaaac ttttccttgc cataatggtt gaggtcctat tagtatccat

62461 gatacaagtc aaatcctaga tgtagaaata aaatagaaga aggcaggcct tctccccagc

62521 gggaaaaatg agattatcgc ggatataaca cattccaaaa ttagccaaat tttcccgacg

62581 tagaagcaat caagaaagct gcataagtga atatataacc tacagaaaaa tgggctaatc

62641 caaccaatct tgcttgcaca atggaaaggg ccacaggttt atctctccat cgaatcaaat

62701 tggccaaagg tgtgcgttca tgagcccatg ctaaagtttc aatcaattcc tgccaatacc

62761 cacgccagga gattaagaac ataaatccag tagcccaaac aagatgtcca aataagaaca

62821 tccacgccca gacagataaa ctattcatac caaaagggtt atatccattg ataagttgtg

62881 aagagtttaa ccacaaataa tctcttaacc agcccatcaa ataagtagaa gattcattaa

62941 actgtgaaac gttaccctgc cataatgtga tatgcttcca atgccaataa aaagtaaccc

63001 atccaatagt atttaacatc caaaaaactg ccaaataaaa tgcgtcccat gccgaaatat

63061 cacaagtacc ccctcgtcca ggaccatcgc atggaaaact ataaccaaaa tcctttttat

63121 ctggcattaa cttggaacca cgtgcatcta aagcaccttt tactaagatc aatgtagttg

63181 tatgtaaacc cagagcaata gcatgatgaa ccaaaaagtc tccaggacct attgttaaaa

63241 ataatgaatt agtattttca ttaacagcat ttaaccagcc gggtaaccag atgcttcgac

63301 ccgcagtgaa tgccgggcca ctcgttgaag ataaaagtac atcaaaccca tatgcagttt

63361 taccatgagc cgattgtatc cattgagcaa atatgggttc gatcaaaatt tgcttttccg

63421 gagtaccaaa agcaagcatg acatcattat gaacataaag acccaaggta tggaatccca

63481 gaaagaggct ggcccaactt aaatgagata tgatagcttc cttatgatct aacattcttg

63541 ccaatacatt atcttcattt tgctccgggt tgtaatctct aatgaaaaag atagctccat

63601 gagcaaaagc ccctgtcatg atgaatcctg caatatattg gtgatgagta tataatgcag

63661 cttgagtagt aaagtcttgt gctatgaatg cataagcagg taaagagtac atgtgttgag

63721 ctactaagga ggtaataacc cctaaagaag ctagagcaag gcctaattga aaatgaaggg

63781 aattgttaat tgtatcataa agacccttat gcccacgtcc caaccgccct cctggaggaa

63841 cgtgtgcatc taaaagatcc ttgatactat gtccaatccc gaaattagtt ctatacatat

63901 gaccagcaac aagaaaaata aatgcaatag ctaaatgatg atgagccata tcagtcagcc

63961 ataaactttg tgtttgtgga tggaatcctc caagaagggt tagaatggca gttcctgctc

64021 cttgggaggt cccaaataaa tgattacttg aatcagggtt ttgagcataa agattccact

64081 gaccggtaaa aagcgggcct aacccttggg gatgcggtaa tacatctaag aaattattcc

64141 atcgaacgta ctcccctctg gatccaggaa tagcgacatg tactaaatgt cctgtccaag

64201 ccaaggaact tacgccgaag agtcctgaca aatgatgatt cagccgagat tcggcatttt

64261 tgaaccacga aacgctcggt ttccatttcg gttgtaagtg tagccaacct gctattaagg

64321 atatggcaga aagaaataat agaaaaagag ctccagtata aagatcttca ttagtgcgta

64381 aaccgattgt ataccaccac tgataaacac cagaataagc gatattcact gggccaagag

64441 caccccctcg agtaaaagct tccacggccg gttgaccaaa atgaggatcc caaattgcat

64501 gagcaatagg tcttacgtgt aaagggtcct gtacccatga ttcaaaattt ccttgccaag

64561 ctacatgaaa cagatttccg gaagtccaca gaaaaatgat tgctaattga ccgaagtgag

64621 aagcaaaaat attctgataa agacgttcct cagtaatatc atcatgactt tcgaagtcat

64681 gtgcggtagc aataccaaac caaatacgac gagtagtggg gtcctgagct aacccttggc

64741 taaaccttgg aaatcttaat gccataatgc ctttcaaatc ctcctagcca ttatcctact

64801 gcaataattc ttgctaagaa gaacgcccat gttgtggcaa ttccacccag aaggtaatgg

64861 gttactccta cagcgcgtcc ttgtacaatg ctcaaggctc tcggctgagt agcaggagca

64921 acttttaatt tattatgagc ccaaacgatg gattcaataa gttcttgcca ataaccccgt

64981 ccgctgaata gaaacattaa actaaaagcc catacaaaat gagcacctag gaaaaaaagg

65041 ccatatgcag ataatgaaga accataagac tgaattacct gggatgcctg tgcccataag

65101 aaatcccgga gccacccatt aatagtaata gaactctgcg caaagtttcc tcccgtgata

65161 tgagttacta ccccttgatc acttatacta ccccaaacat ctgactgcat tttccaactg

65221 aaatggaata ttactaccga aatcgaattg tacatccaga atagtcctaa gaagacatga

65281 tcccaagccg atacttgaca tgtaccccct cttccaggtc catcacaagg aaaacgaaaa

65341 ccaagatttg ctttatctgg tatcaaacgg gagctgcgag caaagagaac acctttcagg

65401 agtatcaata ccgtgacatg aatcgtaaat gcatggatat gatgtaccaa aaaatccgcg

65461 gttcctaatg gaataggtaa caaagccact ttgccgccca ctgctactaa atcaccaccc

65521 ccccaggtta aactggtgct tgccgttgca ccaggtgccg tcgcgccggg tgctaaagcg

65581 tgggtatttt gtatccattg agcaaaaacg ggttgtaatt gtatagcggt atctgaaaac

65641 atatcttgag gacgccccaa ggcactcatg gtatcgttat gaatatacaa accaaaactg

65701 tgaaagccca gaaatataca tgcccagttg agatgtgata tgattgcatc acgatgtcta

65761 aggacacgat ccaatagatc gttgtatcga gtagttggat cataatctct taccataaaa

65821 atggctgcat gcgcagcagc accaactatg agaaatccac caatccacat gtgatgtgtg

65881 aacaatgaca gttgtgtacc atagtcagta gctagatatg gataaggggg catggaatac

65941 atatggtgag cgacaacaat ggttaacgag cccaacatgg ctaggttaag agataattga

66001 gcatgccatg acgttgttag gatctcatat aggcctttat ggccctggcc tgtaaatgga

66061 cctttatgag cttctaaaat atcttttagt ccatgaccaa tgccccagtt ggtcctatac

66121 atgtgacccg ctatcaggaa caaaatagca atagctaaat gatggtgcgc aatatcggtc

66181 aaccacagac ctccagttac tggatctaat cctccacgaa acgtaagaaa ttccgcatat

66241 tttgaccaat tcaaggtgaa aaatggggtt gctccctcgg caaaactcgg ataaagttgg

66301 gccaaaaggt cgcgattcaa gataaattca tgaggaagcg gtatctcttt aggatctact

66361 ccagcgttta gaaattggtt aatcggtaaa gatacatgta cttgatgccc cgcccaagag

66421 agagacccaa gtcctagtag ccccgctaaa tggtgattca acatagattc tacatcttga

66481 aaccaagcca attttggggc cgctttatga taatgaaacc aaccagcaaa aagcattaac

66541 gctgcaaaga ccaatgcacc gattgcggta caatagagtt gtaattcatt agttattccg

66601 gatgctcgcc aaatctgaaa aaaaccagag gttatttgta ttcctcggaa acccccgccc

66661 acatcaccat tcaatatttc ttgtcccact attggccaaa ccacctgggc acttggccca

66721 atgtgagttg gatcacttag ccacgcttca taattggaaa aacgagcacc gtggaaatac

66781 atgccgctca gccaaagaaa gatgatggag agttgcccaa aatgggcact aaatactttt

66841 cgagagatct cctccaaatc actggtatgg ctatcgaaat cgtgagcatc agcatgtagg

66901 ttccagatcc aagtggtagt atcaggcccc ttagctattg ttcttgagaa atgaccaggt

66961 ttggcccatt gctcgaatga agtttttacg ggatccttat ccaccaaaat ttttacttct

67021 ggttccggcg aacgaataat cattgagtcc tcctctttcc ggacaacaca tacacagaga

67081 cctgcctgcc aacagtcaaa caattagtaa acctttgaga gatatttcta gaattagttt

67141 gtttcttttc tatttttcta tctcccagtt atctatttga tttagttatt tactagagca

67201 attatgatct ggaagtcgat ccggggcaag tgttcggatc tattatgaca tagtcatgag

67261 gcgctcaacg gaccttttga atctcataaa acccttttgt cgctttggat tgatacaaaa

67321 acgatctttt tgtacaacct ggtgatatga attcatctat tccaaatcac gagaacagcc

67381 gttgctaaga aacattactg tatatatata tatattgaaa tatattttca taatttagtt

67441 gttaattttg attttcgata tttaatttag gtttctttat attagtttga attcaaattg

67501 ttttaattta agaaaatgga aataaaaaaa agaagtctat tttgtactct atcccttcat

67561 tttatcctta cgaaatatca gatgaaatag aatgcttaga agggatataa tgaaattctt

67621 tgattggctc tttccacaaa acaacggaat gatccatttt tttatttgac caatgaggcc

67681 aaaaaacaat tcatattaat tataacaaag atatcaaata tatatattat actaaataag

67741 agattaataa actaaatagc gccttctctt attcgaaacg cctcgtgatc ttcaaccaat

67801 tatgtgcttc aatataatta ccaggagtaa gcgctatagc ttgtttccaa tactcagcag

67861 cttgatcaaa ccaagcctcc gcaatttcag aatctccctg gagaatggcc tgttctcccc

67921 ggtcggaata ggtggattaa tcccttccct tagaaccgta cttgagagtt tcctacctca

67981 tacggctcct cgttcaattc ttttggtgta ccgttatcat atctaacaaa tgggatttct

68041 catggatcta tcccaccttt cgggttaacc aaagaggtgc attacatgag ttttaaactg

68101 aaatttggat tgttttcttt cgttttatct tttttcccac cttcagaaca aaaaattccc

68161 cctatcgtta gaattttctg aaaggtaact atctcggttt cgtatagaaa tttatataga

68221 atctttgaaa aagactttcc ttcctaagaa agaaaagact tactatcttt gggatctgat

68281 cctacaccgc tgctcaagac tttagtggat cgactctatt acataagtgg attcctaatt

68341 tttagctcac atcatgagat aagtatatct accatcatca catgacataa gtacgcagtt

68401 attattgtat cggccccaaa ccccgctaat tcatctttac ggtgcttctt ttaccaatta

68461 gattcttttt tttatccata gaaaaaagta gctaggtata tctatttctt catatttcca

68521 cttttatgaa tatgaagttt ctttcttttc tacagctgat aaaatcgttg ttttagacga

68581 tgcatatgta gaaagccctt ttgtttttct tttttttact tctatagtga agatagtcgc

68641 acgtaatgac agatcacggc catattatta aaagcttgtg gtaagaacgg atttcgctct

68701 agtgctcgaa aataatattc cagagctttc gtatgttctc cattacttgt atggataaga

68761 cctatattat agagtatata acttcgatca tagggatcga tttctagtcg catagcttca

68821 taataattct gtaaagcttc cgcataattt ccttcggatt gagctgacat ccgttacggt

68881 cgccattcaa ttaagagaat ctccgttcca gaaccgtacg tgagattttc acctcatacg

68941 gctcctccct tatgttcata aggagaataa tacatgaaat caaaaaagat gaaaatattc

69001 tcattatgga ctgagcaggg ctagtgtttt tacaagaaat ctctagccaa ccttcctgca

69061 agagatcttt tcttaacatc aagcgtgttg ggactagata taaatgagac ctcgaacaat

69121 ttctttgttt tcaacgcctc cgaatttcca ggaattagtc acttcaacaa ccttcgatgg

69181 ttctattggt atccaaagta cgaacgagat ggatatttgt tgtcccaacc attcttttag

69241 tcccgagccc aataaggaaa ggggtaattt ctaacaaggt tttcgtgttg ttgattccta

69301 ggtgtagtgc ttcttccctt atgctgtccg ttggtactag tggagtagga ttgccccata

69361 atccagaacc tctaggtgta acctttcgct caatactaga atcaaaaatt gaaacatagc

69421 atctgaggtt gcattaatcg aggatacacg acagaaggaa ttgttctatt tccaaacttc

69481 accttcaaca agcgtagatt tatttcaaaa agtttcccga atcgcgtgtc tttctcgtaa

69541 gaccgagaga tctgcaataa aaaaaacgaa tcaaatcaca ccatctctgt aataggtaaa

69601 tgcctccctt tctcctgaag ttgtcggaat tatttgcaat aagatattgg ctacaattga

69661 aaaggtctta tcaataaaat ttccatttat tcgcgatcta ggcatagcca gcaatccatt

69721 ttataattct tctcattccc tttctttgga aaaatgatcc cacaaagaaa agaattgtac

69781 agtacgaaat aacataaaaa cagattgatt taaaaaaaat ttcaagaaat aataaaacaa

69841 ttgtctcttt atcttggagc ctcgaaagaa agatctttct ttactttgat gaaaaatttt

69901 ctatttttat ttgtaatatt aatatctagt taaaatggat tggtcgtacc tatccaataa

69961 tctagcatgg aatatgaaga acccactatt aactagggtt ttggttcata atcattatgt

70021 aggagagatg gccgagtggt tcaaggcgta gcattggaac tgctatgtag gcttttgttt

70081 accgagggtt cgaatccctc tctttccgta ccttcactta atagacccgt tttttgaatt

70141 tattaaaaac accggatcga ctagcaatgg agacccttaa tcccaccagt tagacctatg

70201 gattctctat tccgaattgc cgtgactagg aaaaatactg gaaaaaaata gaaaaatatc

70261 cccgcgggct atgatacata aatgggatcc aatcggggtc acccccacat ttttcttact

70321 taaggttcat tttatttgtt gatgaagggg aagaaaattg cccgaaccct tgtattttat

70381 ttcagtttag gtttaagtct gacgagaata atattctacg actagcaatt catttatttt

70441 caaaccgacc cacttactat ctattatttg attgactaat cctttatatt ggaatgggtg

70501 aagggtcaaa tggtttggca cttccgcctg aggggaagag ttgagagaat tttgaatcag

70561 agttctggat ttttgttcat ctttccccgt aataatatct ctgggtttgc agcgataact

70621 tggtatatct actatacgcc cattcactaa aatatgtcta tggttaacta attggcgggc

70681 tgcgggaata gtcgaagcca tacccaatcg aaaaaggatg ttatccaaac gcatttcaag

70741 taattgtagt aaaacttgac ccgttgaccc cctagctttt ccggcaatac gaacgtattt

70801 aagtaattgt cgttctgtaa gaccataatg aaaccgcaat ttttgttttt cttctaggcg

70861 aattcgatat tgagattttt tcccggaacg cgattggttt ctaagatcac ttccggcttt

70921 aggcctttta ttcgttagtc ctggtaaagc cccaagacgg cgtatttttt tgaaacgagg

70981 tcctcggtaa cgcgacataa agactcctta ttcttatttc gaattcattt cattcttttt

71041 ttgaaaattg gattttacag aataaaccta aactaaaact gaactaaatg aagcgaagtt

71101 tgctgaagta gtgtacttgt actataaaga agaatgagat aaattggata aatattcaaa

71161 ctttcgatta ttatatatat actattatta tatattattc tattattaca tatataagat

71221 ccttttcctg acatagttgg gatttcccta taacttaacc tataacttaa gaaattcatg

71281 gtttttggaa agaggggaat acgctttttc aatattctgt gatttcaaac aaaggaagat

71341 tgcccgtttt tcttcaactt gaataaaaaa aatgaaaaat aggaaaaagc cggctatcgg

71401 aatcgaaccg atgaccatcg cattacaaat gcgatgctct aacctctgag ctaagcgggc

71461 ccgcataata gaaattttct atgcatagga attcaataca ctatagtata agtatagtgt

71521 ctatagaaat atagaaaaga atagaatcta taattcccaa taaatattgg atattataga

71581 acataacgat ttctatagcg aatatagaat tttcatttct ttctcacaat tcgaaattcg

71641 aatattattc tattcgatag tgaatcttaa atatttttcg agagtctagt ccaatttttt

71701 tattatttta aattcacatg acatttgaaa ttctttttgt tacacttcta tattttctat

71761 cctatatatt tccaattcta tatttctttt gaattcgatt gagtagttat aactaatcag

71821 acattcctcg gcttttattc gtaaaggtaa aaatcgaccc ttcaagtatc gacccttgaa

71881 tgggaaaatg ggaggaatag aaagatatat atggggtata tatcaatcta tattgaattg

71941 ccgatacaga aatgataaaa tccaattgga ttggatccaa tatgggcttc ctataggtaa

72001 gaaagaaaag acttttttcg agatagtaat ccctatctaa taaattgaag ggttccggta

72061 taaatgaaag aaaaaaggga atgatatcat aataagattc taagctcaaa acaaaagaaa

72121 agtcagaggg gatatggcga aatcggtaga cgctacggac ttaattataa ttaaattgag

72181 ccttagtgtg ggaaacctac taagtgataa ctttcaaatt cagagaaacc ccggaattaa

72241 taaaaatggg cgatcctgat ccaaatcctg tttttttaaa acaaaggttc aaaaaacgaa

72301 ataaaggata ggtgcagaga ctcaatggaa gctgttctaa caaatggagt tgactgcgtt

72361 ggtagaggaa tcctttctac ggaaacttca gaaaggatga aggataaacg tatctatcga

72421 atactttatc aaatgattaa tgatggaccg aatccgtatt tttaatatga aaaatagaag

72481 aattggtgtg aattgattct atattgaaga aaaaatcgaa tattcattca tcaaatcatt

72541 cactccatag ttcgatagat cttttaaaga actgattaat cggacgagaa taaagataga

72601 gtcccattct acatgtcaat actggcaaca atgaaattta tagtaagagg aaaatccgtc

72661 gacttttaaa atcgtgaggg ttcaagtccc tctatcccca gcctatttga ctcctaaaat

72721 agttatcccg cccccccctt tttcgttagc ggttcgaaat tcctgattct ttgactttca

72781 tattttttgg taaataactt tctcttatca catgtgatat agaatacaca tccacattaa

72841 gcaaggaatc cctatttgca taattcacaa tgaatagctc aggacttgga gaaaactttg

72901 taatcccccc ggtcccttta attgacagag actccagtca tcccataaaa tgaagatggg

72961 atgctacatt gggaatggtc gggatagctc agctggtaga gcagaggact gaaaatcctc

73021 gtgtcaccag ttcaaatctg gttcctggca catggttaaa ttggatgagg tctttctttt

73081 agaaattaat tgatatgaag cgagatacat attgattttg aatctggatc ataatagata

73141 cttttatcta tcttagcgag atatacccca tctattttat agatggggta gagtttctta

73201 aattcttaaa taaataaaaa aataaataaa atgaaatttg attttctctt ttttttcttt

73261 cgtgcaaaaa gaatattaat actttataca tatttcctat ttgaaaggtt caattagttg

73321 gttgaaagaa caaaaagtca aaatttaaat agacaagatt cggctcagat acaaaaaaaa

73381 gaaatcaaat tggataccct ttcatttcat gattaatctt ttcatctcta ttcgatttcc

73441 taattcgtct cgacatgact ttctacaacc ggtctaagga agaataggcg cagtacaagg

73501 ttcatgacgc agaactcatt tgattcatcc tattggttcg gctcatacga acgaaataag

73561 tatcttgcaa ataaatctaa gaatcccaac gaatccccaa ttcaaccttt tttgttagcc

73621 tatccacaat tccctaatac aaatacaaaa gagaccccaa ttattctagt taatgttaat

73681 ctagaacaga aagccaatcc ttgaatctct gaaattttat aagtggaaat gactttctta

73741 tcattcagtg agcatcttgt atttcataaa aattgggggc aatataatcc ttacgtaagg

73801 gccatcctat ccaactttca ggcattaaga tacgtttcag gcgcggatga ttatcataag

73861 agattcccaa catatcataa gattctcgtt cttgaaaatc cacgcttttc caaacccaga

73921 aaacggacgg aattctagga ttcctccttg aggtaaacac ttttacgcat acctcttctg

73981 gttgatccac accatactct attctggtaa gatgatacac actagctaac agtccgccgg

74041 gtgctacatc ataggcacat tgggagcgta gataattgta accatataca tataaaatga

74101 cagcaatgga gtgccaatcc tcgggcttta tttgtaaagt ctctattcct tgataatcaa

74161 agcccaaaga tctatgaatt atcccatgct tgactaacca agcagacaaa cgaccctgca

74221 tcttttttat ctcccacatt tttatttaga atatttcaca ttctcgatca aattgatgaa

74281 gattgacccg ctgcttgtta ttctgtacaa aggagtccgg cctaattcac caattcatga

74341 gaagatactg aacttttata ttcgaaaaag ttttccgtag ggatctctga agtagatggc

74401 ggttgataga ggaatctttg atcataattt ccagtattca tactgcgtcc aacatgaaac

74461 ttgtgattag tcgtaaaaca ccgatttgct tgttgcgacc taattctatt ttcatagatt

74521 tctcgcgata ttttcttacg aagttttgtt atagcatcta taaccgcttc cggtttaggg

74581 ggacaacctg gcaaatagac atccacagga attagtttat cgactccccg aacagtacta

74641 taagaatcgg tactgaacat ccctcctgta attgtacacg ctcccatagc aataacatat

74701 tttgggtcgg gcatttgctc atataatctc actaaggagg gggccatttt cattgttact

74761 gttccggctg ttaaaattag atccgcttgt ctaggactcg atcttggtac tagtccataa

74821 cgatcaaagt caaaccgtga tcctattagt gaagcaaatt caataaaaca acaactggta

74881 ccatagagaa gcggccataa actagagagt cttgaccaat ttgaaagatc atttaatgta

74941 gttgaaataa ctgaaatttt ggctgttcga ttaagtaaag gaaactcgat gggattcata

75001 actttttcaa tctttttttc ccctttcttt ttattgtctg aatattcagg agctaagacc

75061 attccaatgc cccctttcgc catgcataaa ctaaaccaac aattaagata agcacgaaaa

75121 ttaaagcttc tataaatacg gatacgccca atacatcgaa actcattgcc catggataaa

75181 gaaaaaccgt ttcaacatca aaaacaacaa aaactagagc aaacatataa taacggattc

75241 taaattgtaa ccaagcatcg cccatcggtt ctatacccga ttcataactc gaaagtttct

75301 ccggcccttt cctaatcggg gctaaaatcc cggaaataaa aaatgccaaa ataggaataa

75361 gacttgatat tattagaaat gcccaaaaaa tatcatattc gtaaagcaaa aacatagacg

75421 cactcctatg aacgtggaaa ttgtgccgga ttggttgatt cgaattgaat ttcgaaagtc

75481 atccataatt ggttggtcca aataaaatga attcttgttt tgaccaaatc ttctagtctc

75541 ctttgattat tgcggggctc atttcaagat ttatcgactg acttgacagg gatcctattt

75601 tcagcctatt ttatttttga tttccatttt ctttaattgc tttagttagt ataactctat

75661 atgttacgct tagacaaatt ttcttgtttt cgcctaggat tcttgctaaa gaaagcgact

75721 tcaaaataaa atcgaattct tcttttgatt atttgaattt ttttttataa aaattcgatt

75781 tataaatttt tcttttttag gaatagaatc gggaggtctt tttgtcgttt ttttttctta

75841 gtgatttaga atagaacgag tattcaaatg gaagaaaatg ggtaggtatt tccatctcag

75901 gatttcgaat atcttagttt gagtgttggt atattccgtt tatgaaaata aggatggggt

75961 tttctcttga ttgactagag aaaaagaaaa ccatcccctt tttgttttgg tgtgattcgc

76021 taggtctagt ttttagatat accaaaaagg ggagtccggc tagcttaacc ccttgattat

76081 ggacaggaaa attcatatag aatttccctc cgaaaattaa tgacgaaggg ttggtttgtt

76141 tattcacgat tggcacgatt ggaaaaggat cgattcgatc tcttgaaatg cgtttttctt

76201 tgcgttttcg gttcggcttt aaacgattcc tgtgagtgag tttctaggac aaattgggtt

76261 tggatgaacc aaccggtcag ttacaagcaa caaacaagaa tgaagaaatg aaaattatac

76321 aatttttgat ttccaatttt gattttattc attttatagg gctctagggc tatacggact

76381 cgaaccgtag accttctcgg taaaacagat caaacttatt attatcaaaa tgatctgaac

76441 tgtttcaaag acccaacatg catttttttg cattgggctc tttcattaac tgatagaaat

76501 atcagccaat ccgccatatt ttttcttgac agaaaaataa gataagggta tggctccacg

76561 tgttctgatt cattatttgg gattctgatt cagaagcact accaaagtgt ttcaagggtg

76621 gggttatctt gacgtaggtc tgcctctggc ctagatcgtt ttaagttaaa tcaagtctct

76681 attcttcggc ttaaaaaatc caatatgaaa cttcatacac cttcaagttc ataggacgaa

76741 aagagatttt ttgagggcct tgtactcatt atgcctagca ttgaatgtac tggtattcac

76801 cttatcaata tctcaaatca atcatggggt ctgtttggta cctaaacggg cactcaaatc

76861 gaaccctttg tcaggctatt gttctcttaa agttatggag taagacatcg atttctcaat

76921 aagatccatt ttttggattg tatgatggac tcccctgaaa aacattggcg cgcgtgtaaa

76981 cgaggtgctc taccaactga gctatagccc ttagtgcttg tgatgcatat tttatcatgt

77041 atataatttg ttgtcaagat gaatattcta tgatccaaca tcctaaattt gtgagtggtt

77101 gggtggtatt gcttagatta ttattgctta taagcaatat gatatttata atccatcgat

77161 gggatgggtt ccgtttggtt ctctttggga tgataaatga cctacttaac tcagtggtta

77221 gagtattgct ttcatacggc gggagtcatt ggttcaaatc caatagtagg tagaacttat

77281 tagataccat tgactccgac atctaataag ttttttgccc caccctcttc tatttttaga

77341 gatttttttt attgaatcta gttcaatttc atttttgaat tgcgttgtat caaaatggga

77401 ttctgcttga ttggattcac tcgacagaat ccaatcaaaa taggatttag aaacataact

77461 tcttttgatt atttgaacgc accaactagt tatgaaataa cattgacagc ctcgactctt

77521 gtcctagctc gccggagagc tagatttgcc tcaattattt gtctctttcc ttcagctttt

77581 ctcaaattag cttccgctat ttcaagagtt tgctgagctt cttgtggatc aatatcacta

77641 cccttttccg catcatttac taaaacagtg atttcattat tacctattct agcaaaaccg

77701 cccattagag ccatcgttaa ccattggtcc ttaaggcgta ttcttaaaat ccctatatct

77761 acagctgtag caacaggagc atgatttggt aatacgccaa tttgaccact atttgtagat

77821 aaaatgattt ctttcacttc tgaatcccaa acaattcgat taggggtcag tacacaaaga

77881 tttaaagtca tttcttcaaa ttgctctcca tttctaagtt catagccttc gcggtagctt

77941 catcgatatt accgactaaa taaaaggcct gctcaggaag accgtctaat tctcccgaaa

78001 ggatcaattg aaaccctcta atggtttctg ctagaccaac atatttcccc ggagaaccgg

78061 taaatacttc ggctacaaaa aagggttggg ataagaaacg ctcaattttg cgcgctcttg

78121 ctacggttaa acgatcctct tcggataatt cgtccaaccc aaggatagct ataatgtcct

78181 gaagctcttt ataccgttgt aaagtttgct taactctttg cgcaatttca taatgttcct

78241 cgccaacgat ccgaggttga agcatggttg aagttgaatc taaaggatct actgctggat

78301 agatcccttt ggcagccaat cctcttgata gtacggtagt agcatctaaa tgtgcaaatg

78361 tcgtagcagg ggcaggatcg gtcaaatcgt ctgcgggtac ataaactgct tgaatagaag

78421 ttatggaccc ttctttggta gaagtaattc tttcttgtaa agagcccatt tcggtactca

78481 gggtgggttg ataaccgaca gcagaaggca ttctacccaa caaggccgat acttcggatc

78541 ctgcttggac gaaacggaag atattgtcga taaatagaag tacgtcttgt tcattaacat

78601 ctcggaaata ttccgccatc gttagggcag tcaaaccaac cctcatgcga gctcccggcg

78661 gctcattcat ctggccgtaa actagagcca cttttgattc ttcaatattt tcttcattaa

78721 tcactccaga ttctttcatt tccatgtaaa gatcatttcc ttcacgagta cgttcaccca

78781 ctccgccaaa tacggatacg cccccatggg ctttcgcaat attgttaatc aattccataa

78841 tgagtaccgt tttaccaacc ccagctcccc caaatagtcc gatttttccc ccacggcgat

78901 aaggtgctaa aagatcgact actttaattc ccgtttcaaa aatagataat tttgtatcta

78961 actgtataaa ggcgggcgca gatcgatgaa taggaaatgt ggtacgagta tctacaggac

79021 ctaaattatc aacaggctcc cccagcacgt tgaaaattcg tcccagagtc gctccaccga

79081 cgggaacgct tagaggagct cccgtatcaa tcacttccat tcctctcatc agaccatctg

79141 tagcactcat agctaccgcc ctaacccgat tatttcctaa taattgctgt acctcacaag

79201 tcacattaat tggttgacca gcagtatctt ggcctttaac taccagggcg ttataaatat

79261 tgggcatctt gcccggcgga aaggctacat ctagtaccgg accgattatt tggatgatat

79321 tcccctggtt ttttttttca agcgtggaaa ccccagaact agaagtagta ggattactta

79381 ccataataat aataaaaata tatgtcgaaa ttttttttgt gaaaattatc gaattcaaaa

79441 aagaaatgtc cgctaacacg tcgatcggtt aattcaataa caaatgagag ttagcattcg

79501 atttcgttag tgacatccaa tcgaatccaa ttcaattgtt tacttgttca atttcaatga

79561 gtgaatttga aagggtcaac caagcgattt tgaaaatttc aagtggatga atgatgaata

79621 aggatcttga gagcgttttt catttgtcta taatgataga caatctcatc tatattatag

79681 aaaatattct atggaattcg aacccgaact ctatttacat tacgattcat tatttctatg

79741 ttattggctc ttcttcattt tgtattttta tttcagcatt taggactata ctatagtcgc

79801 ctattccttt cttttcgttt ttttataccc ttccatcatc atttcataga tgaattgcgc

79861 ctattttcac atctaggatt tacatataca acatatacac tgtcaagggg aaatttctta

79921 ttagttaggt taggtatttc gattccaaaa aagggcaaaa agaaaaatgg ggttgcgcta

79981 tatatatgaa agagtataca ataatgatgt gtttggtaaa tcaaatacca tggtctaata

80041 atcaaacatt ctgattagtt gataatatta gtattagttg ggaagtttgt gaaagattcc

80101 tgtgaaaagt ttcattaacg cccaattcat tcgtgtcgag tagaccttgt tgttgtcaga

80161 attcttaatt catgagttgt agggagggat ttatgtcacc acaaacagag actaaagcaa

80221 gtgttggatt caaagcgggt gttaaagagt acaaattgac ttattatacc cctgaatacg

80281 aaaccaaaga tactgatatc ttggcagcat tccgagtaac tcctcaacct ggagttccgc

80341 ccgaagaagc aggggccgcg gtagctgccg aatcttcgac tggtacatgg acaactgtgt

80401 ggaccgatgg acttaccagc cttgatcgtt acaaagggcg atgctaccac atcgagcccg

80461 ttcttggaga aaaagatcaa tatatctgtt atgtagctta ccctttagac ctttttgaag

80521 aaggttctgt tactaacatg tttacttcca ttgtaggaaa tgtatttgga ttcaaagccc

80581 tacgtgctct acgtctggaa gatctgcgaa tccctactgc ttatactaaa actttccaag

80641 gcccacctca tgggatccaa gttgagagag ataaattgaa caagtatggt cgtcctctgt

80701 tgggatgtac tattaaacca aaattggggt tatctgctaa aaactatggt agagcagttt

80761 atgaatgtct tcgcggtgga cttgatttta ccaaagatga tgaaaacgtg aactcccagc

80821 catttatgcg ttggagagat cgcttcttat tttgtgccga agcaatttat aaagcacaag

80881 ctgaaacagg tgaaatcaaa gggcattatt tgaatgctac tgcgggtaca tgcgaagaca

80941 tgatgaaaag ggctgtattt gctagagaat tgggagttcc tatcgtaatg catgactact

81001 taacaggagg attcactgca aatactacct tggctcatta ttgccgagat aatggcttac

81061 ttcttcacat tcaccgtgca atgcatgcag ttattgatag acagaagaat catggtatgc

81121 acttccgtgt actagctaaa gcgctacgtc tgtccggtgg agatcatatt cacgccggta

81181 ccgtagtagg taaacttgaa ggagaaagag aaattacttt aggctttgtt gatttactgc

81241 gtgatgatta tattgaaaaa gatcgaagtc gcggtattta tttcactcaa gattgggtct

81301 ctctaccggg tgttattccc gtggcttcag ggggtatcca cgtttggcat atgcctgctc

81361 tgaccgagat ctttggggat gattccgtac tacagttcgg cggaggaact ttaggacacc

81421 cgtggggtaa tgcgccaggt gctgtagcta accgagtcgc tctagaagca tgtgtacaag

81481 ctcgtaatga aggacgtgat cttgcttctg agggtaatgc aattatccgc gaggcttgca

81541 agtggagtcc tgaactagct gccgcttgtg aggtatggaa agagatcaaa tttgaattta

81601 aaccagtgga tactttggat ccggaaaaga caaactaatt actctccgtt ctcttaattg

81661 aattgcaatt ttactcggcc caatctttta ctaaaaggat tgagccgaat ataaagattc

81721 tatatgcatg tattttggat aaatacaggt atctctagat aatagaaaga tttgaaatcg

81781 aaattgaaga ctcaaatttt tctattgttg tcttggatcc acaattaaac ccatggatcc

81841 ttaggattgg tatattcttt tctatatcct gtagtttccc cgaatcaagc gaagtatcac

81901 aaatcgttcg acccatcctg tatattgtct ttttcgttcc gtgttggaac agaaccttaa

81961 tttcttactt gttattagtt agttagtaga cgagattttg cgaaaaatat tcgttctctc

82021 ctagaacaaa tatttccttt ttttgttcga tgcgaagaca gaggaaaaac cactttttat

82081 cattatatgc aatttcgaat caaaggggat tccatcctat tcatatatat atagtgaagt

82141 cttactatag tgaagtctta cccctggatt cccacaaaag agaattattt ttcacacttc

82201 ctatttagta gtgattgaat ttcgatggtt agtttgatag gaaataagat attcaaataa

82261 aaataaagaa ttgtggatcg aataactatt catctattgt attttttata caaatagggg

82321 gcaagaaaac tctatggaaa ggtgttggtt taattcgatg gtctttaaga aggagttaga

82381 acgcaggtat gggataaaga aattaacgga caatcttggg cctatggaaa atactagtga

82441 aggcgaagat ccgaatagaa aaggtagggc taaaaacatg catagttgga gcggtcgtga

82501 caattctagt tatagtaatg tcgatccttt atttggcgcc aaagacattc gaaatttcat

82561 ctctgatgat acttttttag ttagggataa taatggagac agttattcta tctattttga

82621 tattgaaaat catatttttg agattgacaa tgagcattgt tttcggagtg aactggaaag

82681 ttctttttcc agttatcgca attcgaatta tatgaataat ggatctacga atgaagattc

82741 cttatacact cgttacatgt acgatactca atctagttgg aataatcata ttactagttg

82801 cattgacagt tatcttcagt ctcaaatctg tattgatacg cccactgtaa gtgatagcag

82861 tgacagttac atttctaggt gcgtttttca gaaacgtaaa actagtagtg aacccagtat

82921 acgaacccgc gcgaagagta gtgatttaac tctaagagct agcgatctcg atgaaactca

82981 aaaatacaag cagttgtggg ttcaatgcga aaattgttat gcattaaatt ataagaaact

83041 tttgaaatca caaattaata tttgtgaaca atgtggatat catttgaaaa tgagtagttc

83101 agaaagaatc gaagttttga tcgaccccgg cacttgggat cctatggatg aagacatggt

83161 ctcaggggat cccattggat ttcattcgga ggaggagact tataaagatc gtattgattt

83221 ttatcaaaga aacataggat taaccgaggc tgttcaaaca ggcgtaggtc aactaaatgg

83281 tattcccgta gcaattgggg ttatggattt taaatttatg gggggtagca tgggatccgt

83341 agtcggggag aaaataaccc gtttgattga gtacgctacc aataaattta tacctcttat

83401 tatagtgtgt gcttcggggg gggcgcgcat gcaagaagga agtttgagct tgatgcaaat

83461 ggctaaaatc tcgtctgcct tatatgatta ccaatcaaat aaaaagttat tgtatgtatc

83521 aatccttaca tctcctacta ccggcggggt aacagctagt tttggtatgt tgggagatat

83581 tattattgca gaaccaaatt cctacattgc atttgcgggt aaaagagtaa ttgaacaaac

83641 attgaataaa acgatacccg agggttcaca agcttctgaa tatttattcc agaagggctt

83701 atttgaccta atcgtaccac gtaatctttt aaaaagtgtt ctgagtgagt tatttaagct

83761 ccacgccttc tttcccttga attccaattc aatgcactag gttcaattat tttatttgta

83821 gcaaacaagt agtttgctta tcggaatcaa agtaactaag aatgaagttt tctttggtga

83881 cctaagatct aattgtaaaa agaataaaaa gtggcggata actctttttt ttacctggat

83941 tcccgattac taattaagaa gtctctatca acaagataaa aacacgagtt cttcctttcg

84001 tgaaattagg caaataaaac gcattttgtc ttaggtatag aatcaaattc aaatatagaa

84061 aaaaatagat atatggtttt gtatctttct tcatcccccg aaaatcctat tttcactaaa

84121 aatcccggtt gtgccgcatt ctaatgaatc tttccaaaat ttgtaagaaa ctcctattaa

84181 tattaaattc gaagacaaga acaaaacaca aagaaatgaa gaaaaataat caaatggatt

84241 agcatatgta tctttcatgt cgatagacga ataagtccat tttttgagtt ctacatttct

84301 tggacttatt ctttcctatt ctttccttgg acttattcta tatactcaat taaatacgta

84361 tatctgtaat aagaattttc aaattgaatg aattcataat aactacgaat tcatactaat

84421 tacaattatt aattataatt agtataaata ataaatatga tattaaaaaa aaataagaac

84481 aggtacaaat agtaaatcga ggtacccatt ttatgacaac tttccaattt ccctctattt

84541 ttgtgccttt agtaggccta gtatttccgg caattgcaat ggcttcttta tttcttcatg

84601 ttcaaaaaaa caagattgtt tagatctgag gggacccaat ctcatccgtt tttttgaaac

84661 ttagacttgt agcataaccc atatatttat ttcaggaaat aaggtagaac acgtgatttc

84721 tgccgaacat aaaggaaaaa gctcttatgc ctgcagaaaa tgatctatgg ttaactgaat

84781 tccagctggt ttgaaataga tcaggactgc tggataccca aaatgtaaag ttggcggatc

84841 tatatgtata tctgtatatt gggatcatag gaagggtgtg ttattatttt agatctaacc

84901 aatttgagga attactccta aaggttccca tcaaactagt gctagttcac attaaactag

84961 tgctagttga tgaaagttca ttcggaaaca aaaaagtgaa gtcaaaacca tttggggtat

85021 tctctcaatt ccaataaaat gcaatcggat caagtatgag ttggcgatca gaacatatat

85081 ggatagaact tataacgggg tctcgaaaac taagtaattt ttgctgggca cttgtcgttt

85141 ttttaggttc attaggattc ttgttggttg gaacttccag ttatcttggt agaaatttga

85201 tatcttttgt tccgtctcag caaatccttt tttttccaca agggatcgtg atgtctttct

85261 acgggatcgc gggtctcttt attagttcct atttgtggtg cacaatttcc tggaatgtag

85321 gtagtggtta tgatcaattc gatagaaagg aaggaatggt gtgtattttt cggtggggat

85381 ttcctggaaa aaatcgtcgc atcttcctcc gattccgtat aaaagatatt caatccgtta

85441 gaatagaagt taaagagggt atttatgctc gccgtatcct ttatatggac atcagaggcc

85501 ggggggctat tccgttgacc cgtactgatg agaatttgac tccacgagaa attgaacaaa

85561 aagccgcgga attggcctat ttcttgcgcg taccaattga agtcttttga gaaagggatt

85621 gggctgaaga acgaatgctt tctccgcagg agggaaaaat acaagaatct tgtttttttc

85681 tataactaag tgatacttta gatgcagata aatcatatct tgtaaattct tttctttttg

85741 tcaatcgatt ttttgatcat cccaatatct ttctctcaat tattctattc ttgactatgg

85801 aaactccttg gaattttttt gaaatattgg atattttgat agaaaaagag ttctttacgt

85861 ctccaaatct caacaatatt cattccttaa aggacttctt ttgttcattg atcgaaagga

85921 gacacgtgtt ttgtttggaa tttgatgaat tgagatatct ggaaacacaa ttttgtatta

85981 tttcttcagt cgaatttcaa gtggagtctt agtctatttc tgtattcttt ctagattcaa

86041 acaaaatcac aaagaaaata gattcatagg tttaatatct tgtctagaac tcatgtttgg

86101 tttgaaagaa atattggatc acatagagtc gacaagtgaa gtgggttaat taaaaattca

86161 caggttaaaa atggcaaaaa agaaagcatt cacccctctt ttgtatcttg catctatagt

86221 atttttgccc tggtggattt ctctctcatt tactaaaagt atggaatctt gggttactag

86281 ttggtcgaat acggttcaat ccgaaaattt tgtgaatgat atgcaagaaa aaagtattct

86341 agaaaagttc atagaattag aggaaatgct cttcttggaa gaaatgatca aggaatactc

86401 ggagacacat ctacaaaagc ttcctataga aatccacaaa gaaacgatcc aattaatcaa

86461 gatacacaat gaggatcgta tccatacgat tttgcacttc tcaacaaatc taatctgttt

86521 tgttattcta gccggttatt ctattttagg taatcaagaa cttgttattc ttaactcttg

86581 gactcaagaa ttcctatata acttaagtga tacagtcaaa gcttttttga ttcttttatt

86641 aaccgattta tgtatcggat ttcattcacc ccatggttgg gaactaatga ttggttctgt

86701 ctacaaagat tttggatttg atcataatga tcaaattata tctggtcttg tttccacttt

86761 tccagttatt ctcgatacta ttttaaaata ttggattttt cattatttaa atcgtgtatc

86821 tccgtcgctt gtagttattt atcattcaat gaatgattga taaaagatcc gtcaatatta

86881 atccaattag aatgtttctt actttgtagt tctacagaag tattaaaaat aggcgatttt

86941 catactattt tcatagtata cttatactat agtattctag taaaatgatt ccaataaata

87001 gcagaatcgt ggacagggaa ctagactaga gacctgccaa atttattgta gaaattttcg

87061 gatcaatgat tagaccatgc aaactagaaa gactttttct tggataaagg aacatattac

87121 tcgatctatt tccgtatcgc tcatgatata tatcataact cggacatcca tttcaagtgc

87181 atatcccatt tttgcgcagc agggttatga aaatccacga gaagcgactg ggcgtattgt

87241 atgtgccaac tgccatttag ctaataagcc cgtggatatt gaggttccac aggcggtact

87301 ccctgatact gtatttgaag cagttgttcg aattccttat gataagcaag tgaaacaagt

87361 tcttgctaat ggtaagaaag ggggtttgaa tgtgggggct gttcttattt taccggaggg

87421 gtttgaatta gctcctcccg atcgtatttc tcccgagatg aaagaaaaga taggcaattt

87481 gtcttttcag agctatcgcc ctaataaaca aaatattctt gtgatagggc ctgtccccgg

87541 tcagaaatat agtgaaatca cctttcctat tctttccccc gaccccgcta ctaagaaaga

87601 tgttcacttc ttaaaatatc ctatatacgt aggggggaat aggggaaggg gacagattta

87661 tcccgacgga agcaagagta acaatacagt ttataatgct acaggggcgg gcatagtaag

87721 taaaatctta cgaaaagaaa aagggggata tgaaataacc ataacagatc catcggatgg

87781 acgtgaagtg gttgatatta tccctccggg accagaagtt cttgtttcag agggtgaatc

87841 catcaaattg gatcaaccat taacaagtaa tcctaatgtg ggtggatttg gtcagggaga

87901 tgcagaaata gtacttcaag atccattacg tgtccaaggt cttttggtct tcttggcatc

87961 tgttattttg gcccaaatct ttttggttct taaaaagaaa cagttcgaga aggttcaatt

88021 ggccgaaatg aatttctaga ctcgcggatt tattgacatc aggttcgtaa aaagaacaaa

88081 attgttgttt aaaattatgg atgatcaaaa aaaaaaatca attctaaaaa accttttatt

88141 tacctgctct tttgctacga gcttcgggga atcgaatccc ttgtaccgca ttcctagtcc

88201 taataggtat atttttgttt gaagaaagct attttatttg actttaccac cctcttcttt

88261 gtttttttta gccaagtcat aaaacagatc cattttattc tatttcaaat agaataaaat

88321 ggggatagat ataagtaggc gggtaataga acgaactcaa aatgcgtgtg acaaagaatt

88381 ctaggagaca ctatttggct ccctagtctt cgacacaaga aaggggtgta gatcaaaaaa

88441 agttcccctt cttgtgtcga aagagtaatg atttttgatc ctggtcgtca aatcatcaaa

88501 aattcctagt cttggtttcg ggtttccagg tgtatcagaa cttttttcga tttattacgt

88561 acaagaaaat caaaatgcaa ataaatataa aattgttaaa ttcaaataaa gtagtggaca

88621 aaaaaaagaa aacttattca acgaactttc catttttctg agatactcaa aaaaaaatag

88681 ggtaagagta tagaaataat ttgtacgata tttaatctac agaaatatat atatataatc

88741 caggaattgg gcgattcccc cctttgttct aagtacccat gaatactatc aagatcgagg

88801 aagaggtgtt ctgaatcaat caataaagtt catttttcaa aagtatcctc agaaaaaaga

88861 gaatggagtc ttttgaacca gcagaaaaag aaatccactt tgttagttag atgaaaaaaa

88921 gactctgctt attaagaacc caacggggcc tttcccctca aatcagacaa actaaagaag

88981 ggaatcccgt tgagttccta cgctttcatg tctacaactc cattcatccg attactacag

89041 ggatgaacct aatccggaat atgaaccata aaagaaaata cctattaaac caatcacaag

89101 aataccagct acagtaccta ttatccaaag aggaatcctt ccagtagtat cggccattta

89161 ctccgtgtcc ctccaatttc tttcatcaag tggtcatgct agagacataa acagtcgtgg

89221 ataattatga gatgagatcc ttccgaatgg gctaagagaa tgcctagaat tattatttat

89281 tttctttcat tttcctaatt gaagaaataa ttggaaaata aaacagcaag tacaaaaatg

89341 agtaataacc cccagtagag actggtacga ttcaattcaa cattttgttc attcgggttt

89401 gattgtgtca tagctctata attcggaatt aggtttatcg ttggatgaac tgcattgctg

89461 atattgaccc caaaaaaaag acggtaggta cagctaggcc gtgaacagcc aaccagcgta

89521 ctgtaaaaat tggataggtt cgatctatag tcatttgatt agggcctcct aaaacgatct

89581 actaaattca tcgagttgtt ccaaaggatc aaaacggcca gttattaatg gaattccttg

89641 tcggctctct gtgaaatact catttggccg agggcttcca aacacatcgt aagctaaacc

89701 ggtactgacg aataaccaac ccgcaatgaa tagggaaggt atagtaatgc tatgaatgac

89761 ccaatatcga atactggtaa taatatcagc aaaagaacgt tctcctgtgc ttccagacat

89821 gttcagctcc acatattctt gtacagtcaa aggggatcga ttccgtaaaa gatgagatca

89881 gtaaaaggca atcactgaaa ttggatcttt gtaggatcgt caatattgta ccaagggcat

89941 ctttagagta taccgaatca gtatagctat ccttcttctg acacagcaat gcaatttgaa

90001 tcagtatcga aaggaagtgc taaagaattt atttattcct ttcctttacc tgttgatgta

90061 aaatggtgct gtatttaatc gaaaattctt acaatgaaaa agagtatcct atttccacaa

90121 ttcaatttaa gtagatgaga gatagataaa tttccttcaa ttttcttttc atggttgtat

90181 agacagtttt ttgtttgaac ccccctttat ttttcatttt caggagttgg ttaatcgtcc

90241 cgtaacaaat acgagtagta tatcctattc gatgaaagaa agcgaagaaa gaataaaata

90301 attggaatca atagttgtaa tgaattgttg gattggatct caatccaaat cttgatctag

90361 ctacaaggat gatactttat ttgaaaatat cgaaaaaaag agtcttccct aaaaatttca

90421 ttcacaaata agaattcaaa aagagtttca tttgtgaatg aagttacacg ctccagttcg

90481 tattattagt ttatgctcaa cgactcgttt gataagaatc gcgagatggc tagatgttag

90541 aaatgattaa gcaatttcat tttatatgcc cgtcactttc tctccgttcg tgctatctat

90601 aatgatagat gaatcaaaaa ctttcaattg aacttattct ttcaatgggt tttttggata

90661 ttttcccatt ttgaaaaaat ggaaacttag gtaaatgctt tagaaacata tgtataaaaa

90721 gaacatattt catttagccc cttcatgctt actataacta gttatttcgg ttttctacta

90781 gtggctttaa ctataacttc agctctcttt attggtctga gcaagatacg acttatttaa

90841 aatttctatt tgaaagaaac aattcagaaa gtaaatcctt ctttgctttg aaattccgtc

90901 tattcgagag ttagttacca cgttaattgt caatttttgg tcattgagat tcgcgtcaat

90961 tcggattaat atttaggtat agatattacc tctttttttc tccttttcaa aaaatttaaa

91021 tgattgaagt ttttctattt ggaatcgtgt taggtctaat tcctattact ttggctgggt

91081 tattcgtaac tgcatattta caatacaggc gtggcgatca gttggacctt tgattaatta

91141 acatttcttt ttttgattgg cctcttcctt tatccacaag aggtcaaatt ggaattgttg

91201 tgcaagtgat tgctgaatct atttcagtct aattcgactc atgaagaaaa aatcacgctc

91261 tgtaggattt gaacctacga catcgggttt tggagaccca cgttctaccg aactgaacta

91321 agagcgcttt cttataagaa tagaaaagaa tgtaaacata aaaaaaagga ttcttttttt

91381 aacactttta tatgcatata ttctatagtt tcaaaaaaat gaatgattct gtgcaatttg

91441 aatcgatctc gattgattcc tcgttactgc tcaacgaagc agtaataggt agggatgaca

91501 ggatttgaac ccgtgacatt ttgtacccaa aacaaacgcg ctaccaagct gcgccacatc

91561 ccttcaattg tcctacagtg tcattgtaga gaattcctgt cttgttttcc acacccctat

91621 ttcctgcatt gagaaacaca aattttctac ccatttcgtc tttttggtct catttaacat

91681 ataataagaa ggggaaagtc ttagagagtg ttttacattt caattggaat tgcggattcc

91741 gtgtacaact acaagcggcc cttaactaca tatgcatctg atcatatatg cattatcttt

91801 ttctgtatta caataaataa aaagggaggt ttttcaatgc gagatctaaa aacatatctc

91861 tccgtggccc cagtactaag tacgctatgg ttcggagctt tagcaggtct attgatagag

91921 attaatcgtt ttttcccgga tgcgttgaca tttccctttt tttcatttta gttattgaca

91981 tcggaaagaa tgaagaagaa tagagataca atttcaaatc tgtgactaac ccccccattc

92041 cttttctctt ttgtcccttt ttttttctaa tttttagact aagggacgaa agagaaagaa

92101 gaagagtaga ttcaacgtct ccgaaactca ggttccaatt cgaattaaat caatattaat

92161 aataaataat agagtcacgg ggggtagagt aggaaaatcg gggtctaggg caagaataca

92221 agatctttaa ctgatgtcct tcgggtttaa atagagtttc taaatcattg tattacttct

92281 tattctttac tatgtctttg ctttgattta ttattacttt attgattttg atcttttaga

92341 gttggatttc aagttggtaa cttctatttt ttccttcctt tctttttctt catttcgaat

92401 caaaatggaa gagttgagca aatcaaaaat caaaaggagg ttcatggcta agaggaaaga

92461 tgcgcgggta acggtgattt tggaatgtac cggttgtatc caaaacggtg ttaagaaggt

92521 atcaacgggc atttccagat atattactca aaaaaaccgg cacaatatgc cgaatcggct

92581 agaattaaga aaattctgtc cctattgtta caaacatatg attcatgtgg aaatcaagaa

92641 atagatcgaa ccgagtatgt cttatcctta aaaggggaaa aaatgccatt atatagaaca

92701 tattgaaata taaatagaag atatttcatt taaataaaaa attggagtaa aaaaaaaatg

92761 acaattaaga aataaactaa acaaaaaaat catggataaa tccaagcgat cttttctgaa

92821 atccaagcga tcttttcgta ggcgtttgcc cccgattcaa tcgggggatc gaattgatta

92881 tagaaacatg agtttaatta gtcgatttat tagtgaacaa ggaaaaatat tatctagacg

92941 agtcaataga ttgaccttga aacaacaacg attaatcact attgctataa aacaagctcg

93001 tattttatct ttgttacctt ttctcaataa tgagaaacaa tttgaaagaa tggagtcgac

93061 caccagaacg actggtctta gaaccagaaa taagtaggct tattcttttt ctttccgaac

93121 tcaaacagtt ctttttcttt ccgaactcaa acgcgggttg gtgttttgtt tgacaaattg

93181 gagaatccag attgtattat gctttcgtaa gaaaaaaaac gaaccagaaa aaaaagattt

93241 tttttattga acgtgttcat tcattttgac tactttaagc atattttctt atagtcattt

93301 tgaccccacc ttcccggagt tcattctccg gggaactcca tttaaattat tccggtgtat

93361 tctttccaat ctactttttt tcttatttcg ttggaaatca tataaagaca attcctattt

93421 gatatagcga tttgggccaa aattttacga ttaagaagca actgcttctt gtatagatca

93481 tttattaatt tactataact atagaatact ctcccttctc gaattactgc gtttatccga

93541 gtgatccaca aacgacgaaa atttctcttt tgcttatccc tatcccgatg agccgaaacc

93601 aaagctctta ttttctgttg agtaatagtt cgagtaagtc ttgaatgaga cccccgaaaa

93661 cttgatgcaa ataaacgaat ttttgttcta cgcctccggg ctatatatcc gcgtttaatt

93721 ctggtcattg aataaataaa attttgacaa ataactaatc gatttcgttt ctttcagtta

93781 ttcttttacc cgtcctggtc tattaataac caaacggatt tttccaatgt ataaaataaa

93841 aattccaatg gctttggcta ctataacctc cccaaccacg attttttctt ttttttttta

93901 ttttaaaaga aatagaaatt aaatagataa ataaatagtg ggtttcctcg tttctatggt

93961 tacttcttaa acggtgaggt cttctctata caccggagcc tttacttcat ttatttaata

94021 tttcatcaat gttattggta acttgtatag ttcacatcac actctttggc tctactcatg

94081 aattatccag taacaggtct ttcacaataa gacccgccta tacagtaacg gtattgaatt

94141 atgaaatttc gctgggtagc tgaccctcgt agtccgttct tgaccgagcg cgaacttcat

94201 ttttatgttt ttttttgaat ttcaataaga tttcctccgc ttaatggata acgacttgct

94261 accaatggag aattggttct catcttaaat tcaggtgatt ggatttacac cagtggaaac

94321 cataaatttt atacacaata gagggatcga gtggcttatt tttagatagt aagtagatag

94381 taaatggagt tccttcttcc attcgatcct attcactgga ctaatcattc atactggaag

94441 cggtttcttg cttttttgta tcagctcatg atctaaacga gtcgcacata caccctagta

94501 catgttcctc gacgctgagg gcatccccca agagcggggg atttcgtgac atttcgaatg

94561 ggctgtcttg tatttctaat aagttgttta atggttggca tgttgaatat atacataatg

94621 ggctggttta gattgatccg aaccggatga ttatgaattt ttttatttaa tagttaaact

94681 cggagataaa atatcacatc acggattttc acaaaatcca ttttttttca ttcaactgct

94741 acaagatcaa caattccata agcttgggct tctgttgctg acataaaaac gtctctttcc

94801 atgtcttcag atacaaccca taagggtttg cccgtccttt gtacataaac ccttgtgagg

94861 gtttcgcgta gtttaagcag ttcttccgct tccaggataa attctcccgt ctgcgcctca

94921 taaaaagaac tcgcgggttg atggatcatt accctgatga tagaaggttt ctctatctga

94981 tgtgatgaaa ggaacagaaa aggaagatca agaataatag gaaaaaaaga tagaattgaa

95041 caaccgtacg ggcatcatct tttgtgcatt gcatacggct atacaatcaa attgaccctt

95101 actttccaga taaaaataga atctatcagc cccaggaggg taaatggtca aattgccacc

95161 cttccttttg taggagttca aaaatactat gatggctccg ttgcttttct atttgaaaat

95221 ggattttttc tttgattcag caatcccaaa gtttcttttt gatccaacca aaaagtgaaa

95281 aatttggttt tttttttaac tctttttttt ataacttaaa aattgttaag agactttcgg

95341 tgtgaaaaca accaagtttg taacgctgaa ttggacttcc gatagataag agaaaatcgg

95401 aaatatcttt tatctcatac tactctctcg atacataatc gaatcttttg aaaaaaaaaa

95461 acaatgcacg aatttttcat atcgaattcg aagtgccatg ctattattac ttaatattca

95521 tatggcgaag gcatagtttt actttttctc tcaaataaaa aaccccattg gcgccaagcg

95581 tgagggaatg ctagacgttt ggtaatttct cctccaacca ggataaaaga tcccattgac

95641 gcggccaatc ccatgcatat tgtatggacc tccggtcgca caaattgcat agtatcataa

95701 atagcgattc cgggtattac ccatccgcca ggagagttta taaacaaata aagatctttg

95761 gtatcatcct cgatactgag atataccata agaccgataa gttgattcga gatctcgcta

95821 tcaacttctt ggcctaaaaa aagtaatctt tctcgataaa gtcggttgag tagggcaaaa

95881 ttgtatccct taggaaccgt acatgcatct tttggtgcat acggttcaaa aaacaaatag

95941 cgaaaaaaaa agaatcaatg tatagattaa gggctttttc ttcgattttt caataaagac

96001 gaattttttt cttctttatt atataataga aaaacttatc gaattcactt ttcattgatg

96061 tattgtttca tcgagattca atccaaatca cgatggtatt ttcttgttcc tgaatgggtc

96121 tctttcatct ttttaggttt atgctctact ccgggtaaag attcaccccc ttttgatttg

96181 cacatatagg acaaatcttc tcaccaccat ttctttttgg tatgactttc caaataacaa

96241 acaggaatga tttaggatac acgtagtaat cctagatata ttaccaattg ggttttttct

96301 aaacggagcc tggatacttc atttttttag tccaatcaca gtcaaccata aattattcga

96361 attgataata gtactatgaa ttcctccaaa caatgcatct aattgcactt cacgctccaa

96421 tttttgtatg attccatttc tacttcttgg gcgaaacaga ggatatctcg atcgggcgag

96481 agaacgggta aatcccatat gacccaatat atctcacaag tcgcactata cgtcaaccca

96541 agatgcatct tcctctccag gacttcggaa aggtactttt ggaacaccaa taggcataaa

96601 ttgaaagaaa aaagaactaa atactatatt tcactttgat gtggaaacgt aataatgtgg

96661 ttttattgtc tttagaatat tgtctttatc atatttattt tctccataga ttagaaaaat

96721 tcagaaagaa agaaaaaata aataaaggca attttttacg agtaagcctt tcgaatgata

96781 aacgcattta ctcatagaaa gtggtatcaa tccaccattg cgtattggta cttatcgagt

96841 atagaataaa tctgcttctc tttgttctta cgaacagaat tcttccatta tttggaacac

96901 aatagaataa ataacccttg ttaggaaata atccactgaa caggggaagt tcgcagcata

96961 gtcctagtat attccaatgc aataaagtta cgtagtgttt atttttcttt gataaagggg

97021 tattttccat gggtttgcct tggtatcgtg ttcataccgt tgtattgaat gatcccggcc

97081 gattgctttc cgttcatata atgcatacag ctctggttgc tggttgggcg ggttcgatgg

97141 ctctctatga attagcagtt tttgatcctt ctgaccccgt tcttgatcca atgtggagac

97201 aaggtatgtt cgttataccc ttcatgactc gtttaggaat aaccaattcg tgggggggtt

97261 ggagtatcac aggagcgact gtaacgaatc caggggtttg gagttacgaa ggtgtagctg

97321 gggcacatat tttcttttct ggcttatgct ttttggcagc tatctggcat tgggtctatt

97381 gggatctaga aatattttct gatgaacgta caggaaaacc ttctttggat ttgcccaaga

97441 tctttggaat tcatttattt ctctccgggg tggcttgctt tggttttggt gcatttcatg

97501 taacaggcct atatggtcct ggaatatggg tgtctgatcc ttacggacta acgggaaaag

97561 tacaacctgt aaatccggcg tggggcgtgg agggttttga tccttttgtt ccgggaggaa

97621 tagcttctca tcatattgca gccggtacat tgggcatatt agcgggtcta ttccatctta

97681 gcgttcgacc gccacaacgt ctatacaaag gattacgtat gggaaatatt gaaaccgtac

97741 tttccagtag tatcgcggct gtcttttttg cagcttttgt agttgctgga actatgtggt

97801 atggttcagc aactaccccc atcgaattat ttggtcccac tcgttatcaa tgggatcagg

97861 gttacttcca gcaagagata tatcgaagag ttagcgccgg gctagcagaa aatcaaagtt

97921 tatcagaagc ctggtctaaa attcctgaaa aattggcttt ttatgattat attggcaata

97981 atccggcaaa aggaggatta ttcagggcgg gctcaatgga tagcggagat ggaatagcgg

98041 ttggttggtt aggacaccct atctttagag ataaagaagg acgtgagctt tttgtacggc

98101 gtatgcccac tttttttgaa acatttccgg tcgttttggt agacggcgac ggaattgtta

98161 gagcggatgt tccttttaga agggcagaat ctaagtatag tgttgaacaa gtaggtgtaa

98221 ccgttgagtt ctatggcggc gaactcaatg gagtcagtta tagtgatcct gctactgtga

98281 aaaaatatgc tagacgcgcc caattgggtg aaatttttga attagatcgt gcgactttga

98341 aatccgatgg tgtttttcgt agcagtccaa ggggttggtt tacttttggg catgcttcgt

98401 ttgctttact cttctttttc ggacacattt ggcatggtgc tagaaccttg ttcagagatg

98461 tttttgctgg tattgaccca gatttggatg ctcaagtaga gtttggagca ttccaaaaac

98521 ttggggatcc aactacaaga agacaggcag tctgatccaa gacccctttg gtctctttca

98581 tctctatttt cttttttgag ggaattttac atcgaatacc ggagtgaatt tgcatcaccg

98641 ccttttttga ttcttgcttt ttttgagatg attcccaaaa ataaaatgaa aaaaaaaaca

98701 aacaaacagg taggaaagct ataattgtaa accacgatca aatttatgga agcattggtt

98761 tatacattcc ttttagtctc gactctaggg ataatttttt tcgctatttt ttttcgagaa

98821 ccacctaaag ttccaactaa aaagtaaaaa gataaaataa tttttcatta tctcaattga

98881 agtaatgagc ctcccaatat tggaaggctc attacttcaa ctagtccccg tgttcctcga

98941 atggatctct tagttgttga gaaggttgcc caaaagcggt atataaggcg tacccggtaa

99001 aacttacaag taaaccagat ataaagatgg cgactagggt tgctgtttcc attatgatta

99061 tataatttca agaccccaac ggatctatca taagatcgtt tatttacaac gcaatggtat

99121 acaaagtcaa cagatcttaa tgaatacaat aatttatggc tacacaaact gttgagaaca

99181 gttctaaatc gggtccaaga cgaactgtcg taggggattt attaaaacca ttgaattcgg

99241 aatatggtaa agtagctccc ggttggggaa cgactccttt gatgggtgtc gcaatggccc

99301 tatttgcggt atttctatct attattttgg aaatttataa ttcttccgtt ttattggatg

99361 gaatttcaat gaattaaatc tctacgaatc acaaagtcct ggcttttgaa taaaaaatca

99421 atcagttaga actcaaattt ctggcctatt gtattttggt agttcgatcg tggaatttat

99481 ttctttctgt atttccggaa tatgagtgtg cgacttgtta taatggattc tattgatagt

99541 acagagaata agtctgtcgc cttgatggag atggttctac ttcgtcggat atttattcga

99601 gtatctggaa cacgaactat atgaactaga ttacgaaata tttgaactat gattcatact

99661 taatattcga cctcgtgccc ggactgcaaa aaaaattgca aactgttgaa aaaaaaatga

99721 aaatctttct ttttttttta gtcggtttat ctaatttatg gaatatgtga tgaaccaacg

99781 gttcttactc agggaatcct tgggttagct tatgattttt tattgaatcg tcgtggttct

99841 agtatgaatc tgaggtttta atcgattcat agggtcttaa caagagaatt cctatcaagt

99901 caataataaa gaaaggaaaa aagctagatt agagacaaaa ccaaattaaa aagaaaaaat

99961 aggtaaagag aggattcaag aggcccgtaa ggatcaacat aaagacgatt gagccaactt

100021 gatattttgg tagtaccacc acaaagaaga gctttcggat tttttcttct ttcgtacatt

100081 tagagaagat tgaatcggag attcagaagt ttcaaacttt ctatttcatt tacatatccg

100141 actattcttt tttttattgg ggtgtttttg cttgagctgt acgagatgaa agtctcatat

100201 acggttctgg gaggggggtt ttcgcctatc tcaataaagt ctatgattgg ttcgaagaac

100261 gtctcgagat tcaagcgatt gcggatgata taactagtaa atatgttcct ccccatgtca

100321 atatatttta ttgtttaggg ggaattacgc ttacctgttt tttagtacaa gtagctacag

100381 ggtttgctat gactttttac tatcgtccga ccgttactga agcttttgcc tctgttcaat

100441 acataatgac ggaagctaac tttggttggt taatccgatc agttcatcga tggtcggcaa

100501 gtatgatggt cctaatgatg attctacatg tttttcgtgt gtatctgacc ggcggattta

100561 aaaaaccccg cgaattgact tgggttacgg gtgtggttct gggagtattg accgcatctt

100621 ttggcgtaac tggttattcc ttacctcggg accaaattgg ttattgggcg gtgaaaattg

100681 taacaggtgt acctgaggct attcctgtaa taggatcccc tgtggtagaa ttattgcgcg

100741 gaagtgcaag tgtgggacaa tccaccttga ctcgttttta tagtttacac acttttgtat

100801 tgccgcttct tactgctgta tttatgttaa tgcactttcc aatgatacgt aaacaaggta

100861 tttctggtcc tttatagaga agatatataa tagatagttg gagttgtaat caatcattta

100921 tcacttagag gaggaataag aggattttat tgctacaagt atggattatt gaaaataata

100981 agacatgaat ttggatattt cccttcaact aatcatgtca aataaataat attttctagt

101041 tgaagggaat tctacgaaga gaaaatggat tatgggagtg tgtgacttga actattgatt

101101 ggtctatgta gatatctatc tgccgtattg gaatttacaa ccaaatgtgt ctttgttaca

101161 accactgtgt aacctctata cagaagatag gctggttcgc ttgaagagaa tcttttctat

101221 gatcagatat aaatcgtgtt gtacatgagt gggctccgta aaatctagta taattaagtg

101281 aaatagatca aataaaacag aatccttatc tatatctatt tcacttaatg aatttcctac

101341 ttatttaaaa tggaatagta tgtaaatgca ttcatttcct ctgcattgat atgatcgata

101401 atactatcgg ggtgaaacaa gagatctaaa gaaaaatatc ggctagacta aattagtaac

101461 aagtaaacct tttgtgtgtg tagctccaac tattttggag ataacacagt aatcgtaaag

101521 tctgagacga cccaaaaagc acttgatcat atcatgatcc actttgtaag ccaacttggg

101581 tattgagtat ttacttagac ttagagctga attctttgca gcggggtagt tgcaattccg

101641 gaaaagagtc aaatttttct tacattgaat cattcatata tgtgtagata tagccaacac

101701 atagattttt tatggattca tttggttctt ttgagtcttg ctcgagctgg atgataaaaa

101761 attatcatgt ccggttcctt cgggggatgg atctataaga attcacctat cccaataaca

101821 aaaaaacccg atttgaatga tcctgtatta agagctaaat tggctaaagg tatgggtcat

101881 aattattacg gagaacccgc atggcccaac gatcttttgt atatttttcc agtagtaatt

101941 ctaggtacta ttgcatgtaa cgtaggctta gcggttctag aaccgtcaat gattggtgaa

102001 ccggcagatc catttgcaac ccctctggaa atattgcctg aatggtattt ctttcccgta

102061 tttcaaatac ttcgtacagt gcccaataaa ttattgggtg ttcttttaat ggtttcagta

102121 cccgcgggat tattaacagt acccttttta gagaatgtta ataaattcca aaatccattt

102181 cgccgtccag tagcgacaac cgtctttttg attggtaccg cagtcgctct ttggttgggt

102241 attggtgcaa cattacctat tgataaatcc ctcactttag gtctttttta atttgattca

102301 attgtgaaat aacacgacgt atgtatctag ggaatagttg cttcaaagcg aattttccct

102361 agatacatct attcaataaa attatcaatt tatttcgaat atatgaattg tgctgcagat

102421 tcaactctta tatcttaatt aaatccaatt gatttaaaac ttgttttttg gtaaatcaat

102481 tgcgaaattt ttttctagaa tgcccaatat atgttttaca tcttctaggc gaaaatgttc

102541 aattttcata agatcttctt gactgttatt caaaaggtcc aataatgtat atatattgga

102601 ccttttgagg caattataaa ccctgggaga caattcggat tgatcaataa aaatcgattt

102661 caatgctatt ttatttttgt tttttcgtac tttatccaat ttatcgtgaa aagtaaaagg

102721 ggataaggga agcctgtatt gattgtcctc taaaggtaag tttttttctt ccttatataa

102781 aaagggaata aacaaatcaa tcaaattccg ggaagcttca tgaagtgctt ctttcggagt

102841 taaactccca tttgtccata tttcgagaaa tagtatctct tgtttttcag tcccattttc

102901 ataggaatga atactatgat tcacgtttcg aacaggcatg aatacagcat ctataggata

102961 acttccatct tgaaaggtat gtggcatttt gataagatat ccccgatttc tctcgatttc

103021 taatccaata cacaaattaa tgggttctgc taagctagct atatgttgtg tattgtcgac

103081 gatttccaca taaggtggta agatcatatc ttgagcagtt acatatccgg ggcccctggc

103141 gcaaatcgac gcgccacaag ttccatatag attacttctc aatacaattt ctttcaaatt

103201 cattataatt tcgtggaccg attcttgaat acccgttata gtcgaatatt catgcgggac

103261 attctcagat tttacgcgtg tgatacatgt tccttctatt tctccaagca aagctcttct

103321 catcgcaata cctattgtgt cggcctgccc tttcataagt ggagacagaa taaagcgccc

103381 gtaataaaga cgtttactgt ctgttcttga ttcaacacac ttccactgca gcgtccgagt

103441 agatactgtt actttctctc gaaccatagt aataatattt tatttgattg aattatttat

103501 ttctcttgtt tctcttgaaa tttcttcact cgtcatttct acacacgtct ttttttgggg

103561 ggtctgcagc cattatgtgg catgggggtt acatcccgca caaaagttaa tagtagacca

103621 cttctacgaa tagctcgcaa tgccgcgtct cttccgagac cgggaccttt tatcatgact

103681 tcggctcgtt gcataccttg atctactact gtacgaatag catttgtcgc tgcagtttga

103741 gcggcaaagg gcgtccccct tctcgtaccc ttgaatccac aagtaccgga cgaggaccag

103801 gaaacgaccc gaccccgtac atctgtaacc gtgacaatag tattattgaa acttgcttga

103861 acatgaataa ctccctttgg tattctacgt gcacttttac gtgaaccaat acgtacattt

103921 ttacgtgaac caattctcgg tatagctttt gccatattgt agcatctcat aaatatgagt

103981 cagaaatata tggaatatat ccatttgatg tcaaaacaga ttctttattt gtacgtttat

104041 tcctttggtg agtctgatta tccttgtctt tgtttatgtc tcgggttaga gcaaattact

104101 ctaatgcgcc cccgtctgcg gattagtcga catttttcac aaattttacg gacggaagct

104161 cttattttca tatttgtcat tccttatctt aattctgaat ctacttcttg gtagaaaata

104221 agtttcttgc aatttttcat ctcgaatcgt attgaataaa aaccacctaa tctttcgaat

104281 ccttgtttcg gagtcgataa attatacgtc ctctagttga atcataacga cttacttcaa

104341 ttttgacttt atctcctggc agtatccgta taaaactacg tcggatcttt cctgaaacat

104401 aacctataat cagatcttca ttatctaacc gaacccggaa catgccattg ggaagcgatt

104461 cggtaattaa accctcatga atccattttt gttctttcat ctcaggtaaa gcccccttga

104521 agtatcaact aatgtaggag aaacgatatt agacaactca cccctttctt ttttttttac

104581 aaataagaag aggtttcgga tcccatttgg atattaaaag gattaccata tataacataa

104641 aatttctccg ccgattcttt ctagtcgagc ctcccggtct gtcattatac ctcgagaagt

104701 agaaagaatt acaatcccca ttccacctaa aattctagga attcgttgag agttagaata

104761 gattcgtaga ccgggtcggc tgattcgttt taaatttaaa aaatttctac aggtatgtgg

104821 tcttttccta ttccttctat tatgtcgcag ggttaaaacc aaaaaatttt ggtttttttc

104881 tcgatgtttt ctgacgtttt cgagaaaacc ttctcggaaa agtattttaa caatattttc

104941 ggtaatatta gtagatgcta tgcgaacaac tctttttcta tccatatccg catttcgtat

105001 agaggttatt atctcagcaa tagtgtctct acccatgatg aagtaaaatt tttggtgcct

105061 caaaattgga tctaattaac atgttttttt attggtttat gaatatgaat ttttcaaggt

105121 atatgcgtga gacataatct acgaattaat ctagttctta agctatttct tttcaaagat

105181 ccaaaagata tcaggatccc attttatttt ataatacctc gggagctaat gaaactattt

105241 tagtaaaatt gaattgtctc aattcgcggg ggattgcacc aaaaatgcga gttccttttg

105301 gatttccttc ttgatcaatc acaactgcag cattgtcatc atatcgtatt atcataccgc

105361 tgtcacgttt aagttcttta caggtacgaa caattacagc tcttactact tctgattttt

105421 ctaggggcat gtttggtact gcttctttga tcacagcaac aataatgtca ccaatatgag

105481 catatcggcg attactagct cctaggattc gaatacacat taattttcga gccccgctgt

105541 tatccgctac atttaaatgg gtctgaggtt gaatcatatg aagttttgag tctattcttc

105601 caatgcaaag gatgaagaaa ataaaagaaa tattgtttgt caaaaaaaat aaacctgcgg

105661 ttttctttca ttcccaagac tcatttgtgt tggttctaca tttttatccc gaaataataa

105721 attgagttcg tataggcatt tttgatgctg ctattaaaat agcccttcta gctatatttt

105781 cagttacgcc gcccatttca tatagtattc gccccggttt aacaacagct acccaatatt

105841 caggggatcc tttccccgaa cccatacgtg tttcggcggg tcgtattgta actggtttgt

105901 ctggaaatat tcggacccat atttttccac cgcggcgtgc atttcgtgtc attgctcgcc

105961 gacccgcttc gatttgtcta gatgtgatcc aagcaggctc aagcgcctga agagcatatt

106021 taccgaaaca aatatgatta cctcgataag atattccctt cattcgtcct ctatgttgtt

106081 tacggaatct agttcttttg gggttataat tgatggttgt ttcggaagtc catctctact

106141 acagaactgg acgtgagagt ttcttctcat ccagctcctc gcgaataaaa ggattcaaaa

106201 tggaattgca attaatttga atagatatta gaacattttt agaaaaaatt tgataaaaaa

106261 tcgttttttt tttggaacgt cctattaaat ctttcgtttc ttttgtcttt tagccaggtt

106321 taccaattca aattaaaaaa aaattatcgc gggcgaatat tgactcttgc aatctctatt

106381 tcagtcctag cacttgttcg tcatttctcc aaatagatga attgatttcc ggttcatttc

106441 gccatcccaa cgagtgaatc attaagattc atttgttcaa taaaatcttt tgcattcaca

106501 ggttccttcg tccccaccac ttcgtactaa atggttaggt cagaattcta caacgaagct

106561 tctaatccaa tttatccttg agtcaatctt cttagtcttt attggctcga agctcttgag

106621 ttttttcttc tataatctat aagaaggatt catttaatat ttcattatgg atgaatcaat

106681 tagtattgat gctttattac actgtctttt atgagaggac tcatagacct tacatattgg

106741 aattctatat cattcatatt tttttctttc tttctctcac ccttccattt atccacactc

106801 ttgttctgta ttttatttta aacttagaat tcattaaagt ttaattgtaa aaaaatttca

106861 gttgctacaa agatatgacc gatttggcat atcttgacag gttctttaga accagataat

106921 atgaagcgat ggattggttt ttatactacc gggccggtct ttttttaatc ccaaccctct

106981 aaaaccaacg agtcacacac taagcatagc aattatatca aaacaaaggg tcaatctaat

107041 ttttattcaa ccctatagaa ttaaaagaat taaagaattc ggaatttagt tgattggcca

107101 gaaaaagaat gaatgagttt cccccttttt gttctatcga cggaaaaaca atgaaagttt

107161 tgttattcct ctcccttgtc tataaaaatc caaattttga tgcctaatac accatagata

107221 gtccgaactg tataggaaca ataatcaatt ttagcgcgaa tggtttgtag gggaacccga

107281 ccttctctga tccattcgac acgtgcaatt tcttttccgt cgatacgccc tgcaatttgt

107341 atttgaattc cttttgtatc tgcttgttca gtcaattcaa tagccttttt cattgctttt

107401 cgaaatgaaa ctcgattctt taattgtccg gctataaatt ctgcaagaat attaggattt

107461 ccatagggtt ttgcaattct tgtgatagca atgttaagtt ttcggttcac ataatgaaat

107521 tcgttttgta gattcatctg taattcttcg attcctcgcg gtctactttc gattaataac

107581 ttcgggaacc ccataaagat tatgatctga atcaaatcga ttcttttttg aatctctatg

107641 cgggcaattc cctcggcacc ggaggatatt ctcatattct tttgaacata atttgtgata

107701 aaatctctta ttttttgatc ttcttgtaga ccctcagaat aattttttgg ttgtgcaaac

107761 caaagggaat gatggctttg ggttgtacca agtcggaaac caagtggatt tattttttgt

107821 cccatactac ctcactatta tacgcatcat catataccat agttgtcttt tgccatctag

107881 ggttttttaa cgaatagaaa tacatctctt catattcagg taaagatata tctttcacta

107941 caatagttat atgacaggta gcttttttta tcgcataact acgtcctcga gcccggggtt

108001 ttaatttctt cgcggaagga ccctcgttaa cctcagcttt acgaattact aaattggcct

108061 cgtcggaacc cataatcaaa ctcgcatttg ctgctgcaga ataaaccaat ttgaaaatag

108121 gataacatgc tttatagggc atgagttcta gtatcataag tgtttcctcg taggaacagc

108181 cgcgaatttg atcaattatt cttcttgctt tgtcagcgga taaagatata tgtcgaccca

108241 aagcgtatac ttctgttttt ttcttcctta gcataagggt tctctcctag taatgaatca

108301 taagtatcta tctatatgtt ttttaagata agattaacgg cgggatctat tatcgctttt

108361 tgcgtgtcct tggaaattca aagtaggtac aaattctccc aacttgtgac ctaccatacg

108421 atctgttata taaataggca aatgctcctt accattatga acagcaatcg tatggccgat

108481 cattgtgggt ataatggtag atgcacggga ccaagttacg attatttctt tttctgcttt

108541 tttattaagc ttgtcaattt ttcttaataa atgattggct acaaaaggat ttttttttag

108601 tgaacgtatc acagtttact cctatttttt ttaaagacga agaaagaaat tctattttct

108661 cgcctattta ctacttacta cggcgacgaa caatcaaatt ttcactatat ttattccttt

108721 ttctacttct tcttccaagt gcaggataac cccaaggggt tgtgggtttt tttctaccaa

108781 ttggggctcg cccttcacca cccccatggg gatggtctac agggttcata acgactcctc

108841 ttactacagg acgcttacct agccaacgct tggatccggc tctacccaaa cttttctggt

108901 tcgccccaac attccccact tgtccgactg ttgctgagca gtttttggat atcaaacgga

108961 cctccccaga aggtaatttt aatgtggccg atttcccctc ttttgcaatc agtttcgcta

109021 cagcacccgc tgctctaact aattgtccac cctttccaag tgtgatttct atgttatgta

109081 tggccgtgcc taagggcata tcggttgaag tagattcctc tttttgatca atcaaaaccc

109141 cttcccaaac tgtacaagct tcttccaaag catacggctt tctggatgta gatgatgata

109201 tctatacaga cggatcttat atatatggta caatgaagta ccgcatgggt ggatatctat

109261 atgaatccaa atctgccgaa tcactcatgg tatgatcttc tacatcctag gttttcccgt

109321 tccgtcatct ggcttatgtt cttcatgtag cattcagacc gaatgactct atgaaattac

109381 gtcgatactt ccacatatta tgggtaacgt aggagacatc tctatttttc ccccggggaa

109441 tctttagaat tcccactgct tagctttcaa ttcgcctctg accatcaaat gaaatgtgaa

109501 taacccgtcc tcctctcttt gaaagaaggg gcgcttccgg ttctgtcggt gcttgaaact

109561 attttgtctt ctccatatta ctatatctct agagtcaata attttatatg aggaactgct

109621 gaactcaatc acttgctgcc gttactcttc agttttctgt tgaggtctat cctgtagagg

109681 tactcaaatt ggatcagtga tcgatttcta ggtttcgtcg taaacctaat tggttacttc

109741 caattacgta aatcaatagt tcaaaccgca ctcaaaggta gggcatttcc catttttatc

109801 ggaacttctg taccagaaac aatggtatct ccaattatag cccctctggg atgtaaaata

109861 tatctcttct caccatcccc atagtgtatg agacaaatgt atgcatttcg attagggtcg

109921 tattctatgg ttacgattct accatatatg tctttttcat tccgtcgaaa atcgatttta

109981 cggtatagac gcttatgacc tccccctcta tgccttgagg taatgattcc tctggcatta

110041 cgacctttac cacaatgatg ctgcccatag atcaaattat ttcgtggatt ggatttcact

110101 tgactgtcta cggttccatt gcgtgtgctc ggggtagaag ttttgtataa atgtatcgcc

110161 atgctattaa gtattttgat ttaagttctt ttctttctaa gaggtggaat agaataaccc

110221 ggttgaagcg taatgatcat acgtctgtaa tgcattgtat gtcccataat gggtcccatt

110281 cttctaccct ttcccggaag tcgatgacta ttcatagcta ttaccctgac accaaagaag

110341 agttcgaccc aatgctttag ttctgtccta gttgatcctg attcgacatt agaagtatat

110401 tgatttttcc ccaataaccg aatacttttg tctgtaaata ctgcatgttt gattccatcc

110461 ataaatcgaa tttcttccct atgagttcta gtctcaataa gaatgctatt tcttactgtt

110521 catatactat gatatgaata tactacacca attcgttatg tatggatgat gagattccat

110581 tgatacagag ccaattccaa tagacttatt ggagggttcc attggcgtgc atccagtagg

110641 aattgaacct acgaattcgc caattatgag ttgggcgctt taaccattca gccatggatg

110701 cttagggggg atcctcgtac atggtgaata accaaattcc aattgaaatg aaatctttag

110761 gataaatcaa tgcaatttag gaggaatcaa tgaaaggaca tcaattccaa tcctggattt

110821 tcgaattgag agagatattg agagagatca agaattctca ctgtttctta gattcatgga

110881 cccaattcaa ttcagtgggg tctttcattc gcattttttt ccaccaagaa cgttttctaa

110941 aactctttga cccccgaatt ttgagtatcc tactttcacg caattcacag ggttcaacaa

111001 gcaatcgata tttcacgatc aagggtgtac tactatttgt agtagcggtc cttatatatc

111061 gtattaacaa tcgaaatatg gtcgaaagaa aaaatctcta tttgaggggg cttcttccta

111121 tacctatgaa ttccattgga cccagaaatg atacattgga agaatcggtt gggtcttcca

111181 atatcaatag gttgattgtt tcgctcctgt atcttccaaa aggaaaaaag atctctgaga

111241 gttgtttcct gaatccgaaa gagagtactt gggttctccc aataactaaa aagtgtagca

111301 tgcctgaatc taactggggt tcgcggtggt ggaggaactg gatcggaaaa aagagggatt

111361 ctagttgtaa gatatctaat gaaaccgtca ctggaattgg gatcttattc aaagagaaag

111421 atctcaaata tctggagttt ctttttgtat attatatgga tgatccgatc cgcaaggacc

111481 atgattggga attgtttgat cgtctttctc tgaggaagag gcgaaataga atcaacttga

111541 attcgggccc gctattcgaa atcttagtga aacactggat ttcttatctc atgtctgctt

111601 ttcgtgaaaa aataccaatt gaagtggagg gtttcttcaa acaacaaagg gctgggtcaa

111661 ctattcaatc aaatgatatt gagcatgttt cccatctcct ctcgagaaac aagcgggcta

111721 tttctttgca aaattgtgct caatttcata tgtggcaatt tcgccaagat ctcttcgtta

111781 gttgggggaa gaatccgcac gaatcggatt ttttgaggaa cgtatcgaga gagaattgga

111841 tttggttaga caatgtgtgg ttggtaaaca aggatcggtt ttttagaaag gtacggaatg

111901 tatcgtcaaa tattcaatat gattccacaa gatccagttt cgttcaagta acggattcta

111961 gccaactgaa aggatcttct gatcaatcca gagatcattt ggattccatt agtaatgagg

112021 attcggaata tcacacattg atcaatcaaa gagagattca accactaaaa gaaagatcga

112081 ttctttggga tccttccttt cttcaaacgg aaggaacaga gatagaatca gaccgattcc

112141 cgaaatgcct ttctggatat tcctcaatgt cccggctatt cacggaacgt gagaagcaga

112201 tgattaatca tctgcttccg gaagaaatcc aagaatttct tgggaatcct acaagatcgg

112261 ttcgttcttt tttctctgat agatggtcag aacttcatct gggttcgaat cctactgaga

112321 ggtccactag agatccgaaa ttgttgaaga aacaacaaga tctttctttt gtccctccca

112381 ggcgatcgga aaataaagaa ctggttaata tattcaagat aattacgtat ttacaaaata

112441 ctgtctcaat tcatcctatt tcatcagatc cggggtgtga tagggttctg aaggatgacc

112501 cggatatgga cagttccaat aagatttcat tcttgaacaa aaatccattt tttgatttat

112561 ttcatctatt ccatgaccgg aacaggggag gatacacgtt acaccacgat tttgaatcag

112621 aagagagatt tcaagaaatg gcagatctat tcactctatc aataaccgag ccggatctgg

112681 tgtatcataa gggatttccc ttttctattg attcctacgg attggatcaa aaacaattct

112741 tgaatgaggc cagggatgaa tcgaaaaaga aatctttatt ggttctacct cctatttttt

112801 atgaagagaa tgaatctttt tctcgaagga tcagaaaaaa atgggtccgg atctcttgcg

112861 ggaatgattt ggaagatcca aaaccaaaaa tagtggtatt tgctagcaac aacataatgg

112921 aggcagtcaa tcaatataga ttgatccgaa atctgattca aatccaatat agtacctatg

112981 ggtacataag aaatgtattg aatcgactct ttttaatgaa tcgatccgat cgcaacttcg

113041 aatatggaat tcaaagggat caaataggaa aggatactct gaatcataga actataatga

113101 aatatacgat caacccacat ttatcgaatt tgaaaaagag tcagaagaaa tggctcgatc

113161 ctcttatctt gatttctcga accgagagat ccatgaatcg ggctcctgat gcatatagat

113221 acaaatggtc caacgggagc aagaatttcc aggaacattt ggaacatttc gtttctgagc

113281 agaagagccg ttttcaaata gtgttcgatc gattacgtat taatcaatat tcgattgatt

113341 ggtctgaggt tatcgacaaa aaagatttgt ctaagccact tcgtttcttt ttgtccaagt

113401 cacttctttt tttgtccaag ttgcttttct ttttgtcgaa ctcacttcct tttttctgtg

113461 tgagtttcgg gaatatcccc attcataggt ccgagatcta catctatgaa ttgaaaggtc

113521 caaacgatca actctgcaat cagttgttag aatcaatagg tcttcaaatt gttcatttga

113581 aaaaatggaa acccttctta ttggatgagc atgatacttc ccgaaaatcg aaattcttga

113641 tcaatggagg aacgcccttt ttgttcaata agataccaaa gtggatgatt gactcattcc

113701 atactagaaa taatcgcagg aaatcctttg ataacgcgga ttcctatttc tcaatgatat

113761 tccacaatca agacaattgg ctgaatcccg tgaaaccatt tcatataagt tcattgatat

113821 cttcttttta taaagcaaat cgacttcgat tcttgaataa tccacatcac ttctgcttct

113881 actgtaacac aagattcccc ttttctgtgg aaaaggcccg tatcaataat tatgatttta

113941 cgtatggaca attcctcaat atcttgttca ttcgcaacaa aatattttct ttgtgcgtcg

114001 gtaaaaaaaa acatgctttt ggggggagag atactatttc accaatcgag tcacaggtat

114061 ctaacatatt catacctaac gattttccac aaagtggtga cgaaacgtat aacttgtaca

114121 aatctttcca ttttccaagt caacacgatc cattcgttcg tagaactatt tactcgatcg

114181 cagacatgtt tggaacacct ctaacagagg gacaaatagt ccattttgaa agaacttatt

114241 gtcaacctct ttcagatatg aatctatctg attcagaagg gaagaacttg catcagtatc

114301 tcaattcaaa cgtgggtttg attcacactc catgttctgg gaaatattta ccatccgaaa

114361 agaggaaaag gtggagtctt tgtctaaaga aatgcgttga gaaagggcag atgtatagaa

114421 cctttcaacg agatggtgct ttttcaactc tctcaaaatg gaatctattc caaacatata

114481 taccatggtt ccttacttcg acagggtaca aatatctaaa tttgatattt ttagatactt

114541 tttcagacct attgccgata ctaagtagca gtcaaaaatt tgtatccatt tttcatgata

114601 ttatgcatgg atccggtata gcatggcgaa ttcttcagaa aaaatggtgt cttccacaat

114661 ggaatctgat aagtgagatt tcgagtaagt gtttccataa tcttcttctg tccgaagaaa

114721 tgattcatcg aaataatgag tcaccatcga cacatctgag atcgccaaat gttcgggagt

114781 tcctctattc aatccttttc cttcttcttg ttgctggata tctcgttcgt acacatcttc

114841 tctttgtttc ccgggcctct agtgagttac agacagagtt cgaaaaggtc aaatctttga

114901 tgattccatc atctatgatt gagttacgaa aacttctgga taggtatcct acatctgcac

114961 cgaattcttt ctggttaaag aatctctttc tagttgctct ggaacaatta ggagattctc

115021 tagaagaaat acgggcttct ggcggcaaca tgcctggtcc cgcttatggg gtcaaatcaa

115081 tacgttctaa gaagaaatat ttgagtatca atctcatcga tctcatacca aatcccatca

115141 atcaaatcac tttttcgaga aatacgagac atctaagtca tacaagtaaa gagatctatt

115201 cattgataag aaaaagaaaa aacgtgaatg gggattggat tgatgataaa atcgaatcct

115261 gggtcgcgaa cagtgattcg attgatgatg aagaaagaga attcttggtt cagttctccg

115321 ccttaacgac agaaaaaagg attgatcaaa ttctattgag tctgactcat agtgatcatt

115381 tatcaaagaa tgactctggt tatcaaatga ttgaacaacc gggagcaatt tacttacgat

115441 acttagttga cattcataaa aagtatctat tgaattatga gttcaataca tcctctttag

115501 cagaaagacg ggtattcctt gctcattatc agacaatcac ttattcacaa acttcgtgtg

115561 ggactaatac tttgcatttc ccatctcatg gaaaaccctt ttcgctccgc ttagccttat

115621 ccccctctag ggggatttta gtgataggtt ctataggaac tggacgatcc tatttggtca

115681 aatacctagc aacaaactcc tatgttcctt tcattacggt atttctgaac aagttcctgg

115741 ataacaagcc taaaggtttt atttttgatg atatcgatat tgatgctagt gacgatattg

115801 atgctagtga cgatattgat gctagtgacg atattgatcg tgaccttgat acggagctgg

115861 aactgctaac tatggatagg atgccggaaa tagaccgatt ttatatcacc cttcaattcg

115921 aattggcaaa agcaatatct ccttgcataa tatggattcc aaacattcat gatctggatg

115981 tgaatgagtc gaattactta tccctcggtc tattagtgaa ccatctctct gaaagatgtt

116041 ccactagaaa tattcttgtt attgcttcga ctcatattcc ccaaaaagtg gatcccgctc

116101 taatagctcc gaataaatta aatacgtgca ttaagataag aaggcttctt attccacaac

116161 aacgaaagca ctttttcact ctttcatata ctaagggatt tcacttggaa aagaaaatgt

116221 tccatactaa cggattcggg tccataacca tgggttccaa tgcaagagat cttgtagcac

116281 ttaccaatga ggccctatcg attagtatta cacagaagaa atccattata gacactaaga

116341 caattagatc cgctcttcat agacaaactt gggatttgcg atcccaggta agatcggttc

116401 aggatcatgg gatccttttc tatcaaatag gaagggctgt agcacaaaat gtacttctaa

116461 gtaattgccc catagatcct atatctatct atatgaagaa gaaatcatgt aacgaagggg

116521 attcttattt gtacaaatgg tacttcgaac ttggaacgag catgaagaaa ttaacgatac

116581 ttctttatct tttgagttgt tctgccggat cggtcgctca agatctttgg tctctacccg

116641 gatccgatga aaaaaatggg atcacttctt atggactcgt tgagaatgat tcggatctag

116701 ttcatggcct attagaagta gaaggcgctc tggtgggatc ttcacggaca gaaaaagatt

116761 gcagtccgtt tgataatgat cgagttacat tgcttcttcg gcccgaactg aggaatccct

116821 tagatatgat gcaaaatgga tcttgttcta tctttgatca gagatttctc tatgaaaaat

116881 acgaatcgga gtttgaagaa gggaaggggg aagcagccct cgacccgcaa cagatagagg

116941 aggatttatt caatcacata gtttgggctc ctagaatatg gcgcccttgg gcctttctat

117001 ttgattgtat cgaaaggccc aatgaattgg gatttcccta ttggtccagg tcattccggg

117061 gcaagcggat catttatgat gaagaggatg agcttcaaga gaatgattcg gagttcttgc

117121 agagtggaac catgcagtac cagacacgag atagatcttc caaagaacaa ggcctttttc

117181 aaataagcca attcatttgg gaccctgcgg atccgctctt tttcctattc aaagatcagc

117241 cccctggctc tgtgttttca catcgagaat tatttgcaga tgaagagatg tcaaaggggc

117301 ttcttacttc ccaaacggat cctcctacat ctatatataa acgctggttt atcaagaata

117361 cacaagaaaa gcacttcgaa ttgttgatta atcgtcagag atggcttaga accaatagtt

117421 cattatctaa tggatctttc cgttctaata ctctatccga gagttatcag tatttatcaa

117481 ctctgttcct atctaacgga acgctattgg atcaaatgac aaagacattg gtaagaaaaa

117541 gatggctttt cccggatgaa atgaaaattg gattcatgga acaggagaaa gatttcccat

117601 tccttagccg gaaagatatg tggctatgaa agagggattc agtggaacag aatggactgg

117661 gtggtagagt ggtggaaacg cttctttctt ccctattttg gaccttagct ccatggaaca

117721 atatgttact gctgaaacac ggaagaatta aaatcttaga tcaaaacact atgtatggat

117781 ggtatgaact gcccaaacaa gaattcttga acagcgaaca accagttcag atattcacga

117841 ccaagaagta ctggattctc tttcggatag gccctgaaag gagaaggaag ggtggaatgc

117901 taacaggcgt ctattattga attgacccga cccgatacct tttgggcacg tccagtgcca

117961 aagtcactga atgggtaagt cgccaatccc tggactatgt aatgtacttt atccgctggg

118021 tgactggcgg gcattttacc agaggtttct aatctaccct tgtgtgattc ctgttgaatc

118081 gtatactctg gggcgggtgc aggtcggtat atcaataccg atttgatccg agctccctta

118141 ttgaattgct tattcaatga gcattctcaa tattatgcct tgaagaggac tcgaacctcc

118201 acgctcttta gcacgagatt ttgagtctcg cgtgtctacc atttcaccac caaggcatct

118261 tgaaagtgca tcctattcca tgaatatgat atctatctag tgtgatgtat ggaatatatg

118321 acaagggtgt agtatttcta ttgatcggtc atataggccc gagttggaca tccaattgct

118381 tcgatttgaa ttatccggag aatgccttat gtatatatca aaaagatggc caatcaaacc

118441 tatttgattt ctcgattcaa tagaagctca aaggggtgaa tagggtccca aaaataacga

118501 gaaatatggg gaaaaagcag gtccgattac gcctattcct aatcctaaat ggaacggcgt

118561 agggacccat atgtaaacat agtatctatt tagatacgcg cgaatgaccc cttgagaatg

118621 tatataaccc tattccggcc cggtccggta tggaatgaac ttataatcat ggaatcgact

118681 cgatcatcag attataagtt cataacccca gcccattccc gttttggtcg gaacagatct

118741 actaattctt tgattccagt tagtaagagg gatcttgaac taagaaatag accctagaag

118801 ctaaaaaagg gtatcctgag caattgcaat aatcgggttc attgatattc ctggtatagt

118861 agatgctatc acacatacaa tcatactcaa ttcgatggaa ttgtttgatc ttaaaggaga

118921 tcttctataa tttcgcacgt gaggggtgat ttcttggttt cgtccagtca ttaataactt

118981 gattattttt agataatagt agatagaaac aacgcttgta aggagtccta ttaaaaccaa

119041 ggaatatagg cctgcctgcc atccacacca gaataaatag agttttccga aaaaacctgc

119101 tagtggagga agacctccta gggataagag acatagggct aaagagagag ccaaaaaagg

119161 atctttcgtg tataatcctg cataatctcg aatgttatca gttccggtac gtagaccaaa

119221 taatacaatg caagcaaaag ttcctagatt catggagata tagaacagca tataagttat

119281 catgcttgca tatccatcat tggagtctcc aacaattatt ccaataatta catatccgat

119341 ttgacctatg gacgaatatg caagcatacg tttcatgctt gtttgagtaa tagcaatgat

119401 atttcccaat atcatgctaa gaatagctag gatttccaga agaagatgcc attcgtttga

119461 tgagaagtaa aaaggaatat ccaaaattcg agtggctgaa gctgaagcag ctactttcga

119521 agtaacagaa agaaaagcaa cgactggagt gggagagtca gagtcgaaaa gaggattcct

119581 cacttctttc tctcattcaa aaccgtgcat gagactttca tctcacacgg ctcctaagtg

119641 ataaaagaaa gaagaactcg ttttctttct tttttgatta ccttcctcgc gtatgtataa

119701 gaccgaatcc attcgatttc taaaaaagat tactaatcct taacttttcg aggaatcctt

119761 catcaggtga atgacttatt ttttcaatct tttcaacctt ggttccgtag gagcaagtca

119821 gaaagattga gaaatagaac catctgattt aattcgttct caatagccat gaaatgatca

119881 tcttagggtg atccttttgt cgacggatgc tcctattaca ctcgtagtct ctgaaggatg

119941 agaaccaact atgtagcatc tacatcgaga attcaagtat tgtatacgtc attagtcgga

120001 tcctttgtag gaactacccg taataacgaa cttgcaaaat ggatctgttt atcataaaga

120061 gattcgtcgt tcctgaccct gcttcacctt aattgttatt tgaacaagta aaagttctgt

120121 cttggtccga gtggggatag catttctctt ctgcatgtcc atggagtttt gaaaaatcca

120181 aacatctcag agatagatag agaggtagga atttctcgaa cgaaccgcac tccttcgtat

120241 acgtcaggag tccattgatg agaaggggct ggggaaagct tgaacccaat tcctacagtg

120301 atgaatatga gcgcaattga aattcctggg gagttataca tttgtgtatt gataagacca

120361 ctcactattt cttgaagctc ggtctctccc ccggataaac catatagcca agagaaacca

120421 tgaaccagaa tagaagagct tgccccaccc atgagtaaat atttcatagt agcctcatta

120481 gaccgtacat ctttcttggt atatccagat aataggtagg agcataaact gaaacattct

120541 ggagctacaa agatagttat taaatcgtta gcaccgcata aaaacattcc tcctagagta

120601 gctgttaata cgaataagag aaactctgtt atagccattt ctgtacattc aatgtactct

120661 acggatagag gaatacatag agttgaacat agtaaaataa gaaattgaaa gatttcgttg

120721 aaattgttcg tttggaaatt tcccgaaaag ctaattatag gttcttctct ccatcggaac

120781 aatagggccg ttatgctcat tactaaactt gttgaagaga tgaaatataa ccaaggtata

120841 tctttttgat cagaggttga atcgatcatc agaagaagaa ttaggccaaa aattaggata

120901 cattctggga aaatcaaact tccatcgaag agaagcaaat gaaaggcttt cataaaaatt

120961 ctcgtagaat cgagaatgaa gttttcattc tgtacatgcc agatcatgaa ttagtaactg

121021 catccaattt ccaaaaaaaa tcccaattgt gtcgaacttt ccgtttttgg aatggaatag

121081 gatcaagatc aaaccttatt ccatggtatt tacatgaggt tcctctttaa gaaagtcccc

121141 gagagggctt agttgatcca tgatttatgt ttcatctttc gtttgtttcg agaaatctat

121201 cgatcaattc cgattctttc tttttctctt gattcttttc cgatcgagat gtatagatcc

121261 tgttcatgga ttaacgaaaa tgtgcaaaag ctctatttgc ctctgccatt ttatgagtct

121321 cttccttttt gcgtatggca tcgccactcc ctttggcagc atccactaat tcggaactta

121381 atttgaaagc catatttcga cccggacgtt ttcgggatgc cgctaataac caacgaatgg

121441 caagtgcttt tccttgtgtg gatcctattt caatgggaac ttgatgagtc gatccaccta

121501 cacgtcttgc ttttactgct atatcgggag ttactccacg tattgcttga cgtaaaacag

121561 atagtggatt tgtttctgtc ttttgttgaa tttttttcat ggctcgatag ataatttgat

121621 aagccaatga tttttttccg tgtttcagaa tacggttaac caacatgtta actaatcgat

121681 tacgataaat tggatcggat tttgctgttt tttcttctgc agtacctcga cgtgacatga

121741 gcgtgaaagg ggtttaagaa tcagttttct ttttataagg gctaaaatca cttattttgg

121801 cttttttacc ccatattgta gggtggatct cgaaagatat gaaagatctc cctccaagcc

121861 gtacatacga ctttcatcga atacggcttt ccgcagaatt ctatatgtat ctatgagatc

121921 gagtatggaa ttctgtttac tcactttaaa ttgagtatcc gtttcccccc ctttcctgct

121981 aggattggaa atcctgtatt ttacatatcc atacgattga gtccttgggt ttccgaaata

122041 gtgtaaaaag aagtgcttcg aatcattgct atttgactcg gacctgttct aaaaaagtcg

122101 aggtatttcg aattgtttgt tgacacggac aaagtaaggg aaaacctctg aaattatttc

122161 aatattgaac cttggacata taagagttcc gaatcgaatc tctttagaaa gaagatcttt

122221 tgtctcatgg tagcctgctc cagtcccctt acgaaacttt cgttattggg ttagccatac

122281 acttcacatg tttctagcga ttcacatggc atcatcaaat gatacaagtc ttggataaga

122341 atctacaacg cactagaacg cccttgttga cgatccttta ctccgacagc atctagggtt

122401 cctcgaacaa tgtgatatct tacaccgggt aaatccttaa cccttccccc tcttactaag

122461 actgaagaat gttcttgtga attatggcca ataccgggta tataagcagt gatttcaaat

122521 ccagaggtta atcgtactct ggcaacttta cgtaaggcag agtttggttt tttgggggtg

122581 atggtggaaa agttgacaga taagtcaccc ttactgccac tctacagaac cgtacatgag

122641 attttcacct catacggctc ctcgttcaat tctttcgaat tcattggatc cctttccgcg

122701 ctcgagaatc cactcccctc ttccactctg tcccgaatag taactaggac ccatttagtc

122761 acgttttcat gttccaattg aacactttcc atttttgatt attctcaaag gagaagatta

122821 ttctctttac caaacatatg cggatccaat cacgatccta taataagaac aagagatctt

122881 tctcgaccaa cccccctgcc cctcattctt cgagaatcag aaaggtcctt tccttttcaa

122941 gtttgaattt gttcatttgg aatctgggtt cttctacttc atttttattt aatattcatt

123001 tctttttccc tctctttttt ttatatcatt ccttaagtcc cataggtttg atcctgtaga

123061 atttgaccat tgaacgaagg gtacgaaatc aatcagattt ctttttcgat caaaagtact

123121 atgtgaaatc ttcggttttt tcctcttcct ctatccctat cccataggta cagaatcaat

123181 agagaacctt ttcttctgta tgaatcgatc ttattccatt ccaattcctt cccgatacct

123241 ccccaggaaa atatcgaatt ggatcccaaa ttgacgggtt agtgtaagct tatccatgca

123301 gttatgcact cttctcgaat aggaatccgt tttctgaaag atcctggctt tcgcactttg

123361 gtgggtctcc gagatccttt cgatgaccta tgttgaaggg atatctatct aatccgatcg

123421 attgcgtaaa gcccgcgata gcaacggaac cgaggaaagt atactgaaaa gacagttctt

123481 ttctattata ttagtatttt cgattatatt agattagtat tagttagtga tttagattag

123541 ttaatgatcc cggcttagtg agtcctttct tccgtgatga actgttggca ccagtcctac

123601 attttgtctc tgcgggccga ggagaaaagg ggctcggcgt ggacatgaga gaagcaagga

123661 ggtcaacctc tttcaaatag acaacatgga ttctggcaat gcagttggac tctcatgtcg

123721 atccaaacga atcatccttt ccacgcggat aaatcttttc tttgcctgct aggcaagaga

123781 ctagcaaatt caaaattctg tctcggtagg acatgtattt ctattactat gaaattcata

123841 aatgaagtag ttaatggtgg ggttaccatt atcctttttg tagtgacgaa tcttgtatgt

123901 gttcttaaga aaaggaaaaa aggaatttgt ccatttttcg gggtctcaaa ggggcgtgga

123961 aacacataag aactcttgaa tggaaatgga aaaaggatgt aactccagtt ccttcggaat

124021 cgctagtcaa tcctaggggc agttgacaat tgaatcctat tttgaccatt attttcatat

124081 ccgaaatagt ccgaaaagaa gacccggctc taagttgttc aagatcaaga atagtggcgt

124141 tgagtctctc gaccctttga cttaggatta gtcagttcta tttctcgatg ggggcaggga

124201 agggatataa ctcagcggta gagtgtcacc ttgacgtggt ggaagtcatc agttcgagcc

124261 tgattatccc taaacccaat gtgagttttt ctgtttagat ttgccccccc gccgtgattc

124321 aatgagaatg gataagaggc tcgtgggatt gacgtgaggg ggcagggatg gctatatttc

124381 tgggagcgaa ctccgggcga atatgaagcg catgaataca agttatgcct tcgaatgaaa

124441 ttccgaatcc gctttgtcta cgaacaagga agctataagt aatgcaacta tgaatctcat

124501 ggagagttcg atcctggctc aggatgaacg ctggcggcat gcttaacaca tgcaagtcgg

124561 acgggaagtg gtgtttccag tggcggacgg gtgagtaacg cgtaagaacc tgcccttggg

124621 aggggaacaa cagctggaaa cggctgctaa taccccgtag gctgaggagc aaaaggagga

124681 atccgcccga ggaggggctt gcgtctgatt agctagttgg tgaggcaata gcttaccaag

124741 gcgatgatca gtagctggtc cgagaggatg atcagccaca ctgggactga gacacggccc

124801 agactcctac gggaggcagc agtggggaat tttccgcaat gggcgaaagc ctgacggagc

124861 aatgccgcgt ggaggtagaa ggcccacggg tcgtgaactt cttttcccgg agaagaagca

124921 atgacggtat ctggggaata agcatcggct aactctgtgc cagcagccgc ggtaagacag

124981 aggatgcaag cgttatccgg aatgattggg cgtaaagcgt ctgtaggtgg ctttttaagt

125041 ccgccgtcaa atcccagggc tcaaccctgg acaggcggtg gaaactacca agctggagta

125101 cggtaggggc agagggaatt tccggtggag cggtgaaatg cgtagagatc ggaaagaaca

125161 ccaacggcga aagcactctg ctgggccgac actgacactg agagacgaaa gctaggggag

125221 cgaatgggat tagatacccc agtagtccta gccgtaaacg atggatacta ggcgctgtgc

125281 gtatcgaccc gtgcagtgct gtagctaacg cgttaagtat cccgcctggg gagtacgttc

125341 gcaagaatga aactcaaagg aattgacggg ggcccgcaca agcggtggag catgtggttt

125401 aattcgatgc aaagcgaaga accttaccag ggcttgacat gccgcgaatc ctcttgaaag

125461 agaggggtgc cttcgggaac gcggacacag gtggtgcatg gctgtcgtca gctcgtgccg

125521 taaggtgttg ggttaagtcc cgcaacgagc gcaaccctcg tgtttagttg ccatcgttga

125581 atttggaacc ctgaacagac tgccggtgat aagccggagg aaggtgagga tgacgtcaag

125641 tcatcatgcc ccttatgccc tgggcgacac acgtgctaca atggccggga caaagggtcg

125701 cgatcccgcg agggtgagct aaccccaaaa acccgtcctc agttcggatt gcaggctgca

125761 actcgcctgc atgaagccgg aatcgctagt aatcgccggt cagccatacg gcggtgaatt

125821 cgttcccggg ccttgtacac accgcccgtc acactatggg agctggccat gcccgaagtc

125881 gttaccttaa ccgcaagggg ggggatgccg aaggcagggc tagtgactgg agtgaagtcg

125941 taacaaggta gccgtactgg aaggtgcggc tggatcacct ccttttcggg gagagctaat

126001 gcttgttggg tattttggtt tgccactgct tcacactcaa aacaaaaaga agggagctgc

126061 gtctgagtta aaggaagtct tctttcgttt ctcgacggtg aagtaagacc aagcccatga

126121 gcttattatc ctaggtcgga acaagttgat aggatcccct ttgttacgtc cccatgcccc

126181 ccgtgtgggg gcatgggggc gaaaaaagga aagagaggga tggggtttct ctcgcttttg

126241 gcatagcggg ccccctgcgg gaggctcgca cgatagcggg ccccctgcgg gaggctcgca

126301 cgacgggcta ttagctcagt ggtagagcgc gcccctgata attgcgtcgt tgtgcctggg

126361 ctgtgagggc tctcagccac atggatagtt caatgtgctc atcggcgcct gaccctgaga

126421 tgtggatcat ccaaggcaca ttagcatggc gtactcctcc tgttcgaaca ggggtttgaa

126481 accaaacctc tcctcaggag gatcgatggg gcgattcggg tgagatccaa tgtagatcca

126541 actttcgatt cactcgtggg atccgggcgg tccggggggg gaccaccgcg gctcctctct

126601 tctcgagaat ccatacatcc cttatcagtg tatggacagc tatctctcga gcacaggttt

126661 aggttcggcc tcaatgggaa aagaaaatgg agcacctaac aacgcatctt cacagaccaa

126721 gaactacgag atcaccccct tcattctggg gtgacggagg gatcgtacca ttcgagccgt

126781 ttttttcatg cttttcccgg aggtctggag aaagctgcat ttcccgaatc ctcccttccc

126841 gaaaggaaga gcgtgaaatt ctttttcctt tccgcaggga ccaggagatt ggatctagcc

126901 ataagaagaa tgcttggtat aaataactca cttcttggtc ttcgaccccc tcagtcacta

126961 cgaacgcccc cgatcagtgc aatgggatgt gtctatttat ctatctcttg attcgaaatg

127021 ggagcaggtt tgaaaaagga tcttagagtg tctagggttg ggccgggagg gtctcttaac

127081 gccttctttt ttcttctcat cagagttatt tcacaaagac ttgccggggt aaggaagaag

127141 gggggaacaa gcacacttgg agagcgcagt acaacggaga gttgtatgct gcgttcggga

127201 aggatgaatc gctaccgaaa aggaatctat tgattctctc ccaattggtt ggaccgtagg

127261 tgcgatgatt tacttcacgg gcgaggtctc tggttcaagt ccaggatggc ccagctgcgc

127321 cagggaaaag aatagaagaa gcatctgact acttcatgca tgctccactt ggctcggggg

127381 gatatagctc agttggtaga gctccgctct tgcaattggg tcgttgcgat tacgggttgg

127441 atgtctaatt gtccaggcag taatgatagt atcttgtacc tgaaccggtg gctcactttt

127501 tctaagtaat ggggaagagg accgaaacat gccactgaaa gactctactg agacgaagat

127561 gggctgtcaa gaacgtagag gaggtaggat gggcagttgg tcagatctag tatggatcat

127621 acatggacgg tcgttggagt cggcggctcc cccagggtcc ctcatctgag atccctgggg

127681 aagaggatca agttggccct tgcgaacagc ttgatgcact atctcccttc aaccctttga

127741 gcgaaatgcg gcaaaagaaa aggaaggaaa atccatggac cgaccccatc atctccaccc

127801 cgtaggaact acgagatcac cccaagggcg ccttcggcat ccaggggtca cggaccgacc

127861 atagaaccct gttcaataag tggaacgcat tagctgtccg ttctcaggtt gggcagtaag

127921 ggtcggagaa gggcaatcac tcattcttaa aaccagcgtt cttaagacca aagagtcggg

127981 cggaaaaagg ggggctctcc gttcctggtt ctcctgtagc tggaacctcc ggaaccgcaa

128041 gaatcctgag ttagaatggg attccaactc agcacccttt gagtgagatt ttgagaagag

128101 ttgctctttg gagagcacag tacgatgaaa gttgtaagct gtgttcggag ggggttattg

128161 tctatcgttg gcctctatgg tagaatcagt cgggggcatg agagacggtg gtttaccctg

128221 cggcggatgt cagcggttcg agtccgctta tctccaactc atgaacttag ccgatacaaa

128281 gctatatgat agcacccaat ttttccgatt cggcggttcg atctatgatt tatcattcat

128341 ggacgttgat aagatccatc catttagcag caccttagga tggcatagcc ttatttataa

128401 tttattttct aatttataag atttttataa gggcaataca ataagggcga ggttcaaacg

128461 aggaaaggct tacggtggat acctaggcac ccagagacga ggaagggcgt agtaatcgac

128521 gaaatgcttc ggggagttga aaataagcat agatccggag attcccgaat agggcaacct

128581 ttcaaactgc tgctgaatcc atgggcaggc aagagacaac ctggcgaact gaaacatctt

128641 agtagccaga ggaaaagaaa gcaaaagcga ttcccgtagt agcggcgagc gaaatgggag

128701 cagcctaaac cgtgaaaccg gggttgtggg agagcaatac aagcgtcgtg ctgctaggcg

128761 aagcagcata aatgctgcac cctagatggc gaaagtccag tagccgaaag catcactagc

128821 ttacgctctg acccgagtag catgggacac gtggaatccc gtgtgaatca gcaaggacca

128881 ccttgcaagg ctaaatactc ctgggtgacc gatagcgaag tagtaccgtg agggaagggt

128941 gaaaagaacc cccatcgggg agtgaaatag aacatgaaac cgtaagctcc caagcagtgg

129001 gaggagccat ggctctgacc gcgtgcctgt tgaagaatga gccggcgact cataggcagt

129061 ggcttggtta agggaaccca ccggagccac agcgaaagcg agtcttcata gggcaatggt

129121 cactgcttat ggacccgaac ctgggtgatc tatccatgac caggatgaag cttgggtgaa

129181 actaagtgga ggtccgaacc gactgatgtt gaagaatcag cggatgagtt gtggttaggg

129241 gtgaaatgcc actcgaaccc agagctagct ggttctcccc gaaatgcgtt gaggcgcagc

129301 agttgactgg acatctaggg gtaaagcact gtttcggtgc gggccgcgag agcggtacca

129361 aatcgaggca aactctgaat actagatatg acctcaaaat aacaggggtc aaagtcgacc

129421 agtgagacga tgggggataa gcttcatcgt cgagagggaa acagcccgga tcaccagcta

129481 aggcccctaa atgatcgctc agtgataaag gaggtagggg tgcagagaca gccaggaggt

129541 ttgcctagaa gcagccaccc ttgaaagagt gcgtaatagc tcactgatcg agcgctcttg

129601 cgccgaagat gaacggggct aagcgatctg ccgaagctgt gggatgtaaa aatacatcgg

129661 taggggagcg ttccgcctta gggggaagcg gccgcgcgag cggcggtgga cgaagcggaa

129721 gcgagaatgt cggcttgagt aacgcaaaca ttggtgagaa tccaatgccc cgaaaaccta

129781 agggttcctc cgcaaggttc gtccacggag ggtgagtcag ggcctaagat caggccgaaa

129841 ggcgtagtcg atggacaaca ggtgaatatt cctgtactac cccttgttgg tcccgaggga

129901 cggagcaggc taggttagcc gaaagatggt tatcggttca agaacgtaag gtgtccctgc

129961 tttttcaggg taagaagggg tagagaaaat gcctcgagcc gatgttcgag caccaggcgc

130021 tacggcgctg aagtaaccca cgccatactc ccaggaaaag ctcgaacgac ctttaaacaa

130081 aagggtacct gtacccgaaa ccgacacagg tgggtaggta gagaatacct aggggcgcga

130141 gacaactctc tctaaggaac tcggcaaaat agccccgtaa cttcgggaga aggggtgcct

130201 cctcacaaag ggggtcgcag tgaccaggcc cgggcgactg tttaccaaaa acacaggtct

130261 ccgcaaagtc gtaagaccat gtatgggggc tgacgcctgc ccagtgccgg aaggtcaagg

130321 aagttggtga cctgatgaca ggggagccgg cgaccgaagc cccggtgaac ggcggccgta

130381 actataacgg tcctaaggta gcgaaattcc ttgtcgggta agttccgacc cgcacgaaag

130441 gcgtaacgat ctgggcactg tctcggagag aggctcggtg aaatagacat gtctgtgaag

130501 atgcggacta cctgcacctg gacagaaaga ccctatgaag ctttactgtt ccctgggatt

130561 gggtttgggc ctttcctgcg cagcttaggt ggaaggcgaa gaaggcctcc ttccgggggg

130621 gcccgagcca tcagtgagat accactctgg aagagctaga attctaacct tgtgtcagga

130681 cctacgggcc aagggacagt ctcaggtaga cagtttctat ggggcgtagg cctcccaaaa

130741 ggtaacggag gcgtgcaaag gtttcctcgg gccggacgga gattggccct cgagtgcaaa

130801 ggcagaaggg agcttgactg caagacccac ccgtcgagca gggacgaaag tcggccttag

130861 tgatccgacg gtgccgagtg gaagggccgt cgctcaacgg ataaaagtta ctctagggat

130921 aacaggctga tcttccccaa gagctcacat cgacgggaag gtttggcacc tcgatgtcgg

130981 ctcttcgcca cctggggctg tagtatgttc caagggttgg gctgttcgcc cattaaagcg

131041 gtacgtgagc tgggttcaga acgtcgtgag acagttcggt ccatatccgg tgtgggcgtt

131101 agagcattga gaggaccttt ccctagtacg agaggaccgg gaaggacgca cctctggtgt

131161 accagttatc gtgcccacgg taaacgctgg gtagccaagt gcggagcgga taactgctga

131221 aagcatctaa gtagtaagcc caccccaaga tgagtgctct cctattccga cttccccaga

131281 gcttccggca gcacagccga gacaacgacg ggttctctgc ccctgcgggg atggagcgac

131341 agaagttttg agaattcaag agaaggtcac ggcgagacga gccgtttatc attacgatag

131401 gtgtcaagtg gaagtgcagt gatgtatgca gctgaggcat cctaacagac cggtagactt

131461 gaaccttgtt cctgcatgac ctgatcaatt cgatcaggca ctcgccatct attttcattg

131521 ttcaactctt tgacaacacg aaaaaccatt gttcaactct ttgacaacat gaaaaaacca

131581 aaagccccgc cctccccttc tatccatcca agggatggaa gggcagaggc ctttggtgtc

131641 ccttccagtc aagaattggg gcctcacaat cactagccaa taggcttttc tctcatgccc

131701 ttcttcgttc atggttcgat attctggtgt cctaggcgta gaggaaccac accaatccat

131761 cccgaacttg gtggttaaac tctactgcgg tgacgatact gtaggggagg tcctgcggaa

131821 aaatagctcg acgccaggat gataaaaagc ttaacacctc tcattcttat tactttttca

131881 acgaataaaa tgaaaaataa aaaggtcgtc ttattcaaaa ccccaattat gacatccctt

131941 ctctcccact tcacacctcg gaacgtaccg agataaacgc gctttcacat cttcttaacc

132001 cgaaatggct ggggagagga aaggttcctt tttttgaggg tactcccggg aacagatcca

132061 gtggagacgg ggtggggcct gtagctcaga ggattagagc acgtggctac gaaccacggt

132121 gtcgggggtt cgaatccctc ctcgcccaca accggcccaa aagggaagta cctttccccc

132181 cgggggtagg aaaatcatga tcgggatagc ggaccaaaag ctatggaact tgggtgtggg

132241 tctttggaat ggctttttct ttttatttct tatttatcgt aaatgatgga atcattacac

132301 atagtatgcc cccccgccat cagcgtattt ttttgtttta cgccctgtaa ctcttcctca

132361 gccaggcttg ggcagaatag gcgagcaagt acaagtatta gtagcataac aaacttcctc

132421 gtcattaata tgtttgctcg cggcaattgt gaactctcgg gagaatcgat gactgcatct

132481 ttgattttga tgcagtgcta atacatctga gaattcttaa ttggctagtt gtaaatagcc

132541 ccagggctgt ggattatccg gggactacac cgaggtattg acggttattt tcaaatctcg

132601 cagaacggaa tggtatacga tgagatagaa acaaagacag ggaacaggtt ccctactctt

132661 aacggtcaaa gtgagcccct ttattctatt ctgaattcgt taattcagaa tgaatcaaat

132721 ctccccaagt aggattcgaa cctacgacca atcggttaac agccgaccgc tctaccactg

132781 agctactgag gaacaacggg agattagatc tcatagagtt caattcccgt tctcaaccca

132841 tgaacaatat gagctcgaat cctccttcgt aactcccgga acttcttcgt agtggctccc

132901 ttccatgcct catttcggag ggaacctcaa agcggctcta tttcattata ttatattcca

132961 tccatatccc aattccattc atttaatacc ccctttggtg tcattgacat aatttctagt

133021 ttatctcttt ctatttcttt tctatatatg gaaagttcaa aaatcatcat ataataatcc

133081 agaagttgca atagaaaaga aaagggggag gtttgtgatg atttttcaat cttttctact

133141 aggtaatcta gtatccttat gcatgaagat aatcaattcg gtcgttgtgg tcggactcta

133201 ttatggattt ctgaccacat tctctatagg gccctcttat ctcttccttc tccgagctca

133261 ggttatggaa gaaggaaccg agaagaaggt atcagcaaca actggtttta ttacgggaca

133321 gctcatgatg ttcatatcga tctattatgc gcctctgcat ctagcattgg gtagacctca

133381 tacaataact gtcctagctc taccatatct tttgtttcat ttcttctgga acaatcacaa

133441 acactttttt gattatggat ctactaccag aaattcaatg cgtaatttca gcattcaatg

133501 tttatttcta aataatctca tttttcaatt attcaaccat ttcattttac caagttcaat

133561 gttagccaga ttagtcaaca tttatatgtt tcgatgcaac aacaagatgt tatttgtaac

133621 aagtagtttt gttggttggt taattggtca cattttattc atgaaatggc ttggattggt

133681 attagtctgg atacggcaaa atcattctat tagatcgaat gtacttattc gatctaataa

133741 gtaccttgta tcagaattga taaattctaa ggctcggatc ttgagtattc tcttatttat

133801 tacctgtgtc tactatttag gcagaatacc gtcacccctt tttactaaga aactgaaaga

133861 aacctcaaaa acgggagcgg gagaaagggt ggaaagtgcg gaagaaagag atgtagaaat

133921 agaaacagct tccgaaatga aggggactaa acaggaacaa gagggatcca ccgaagaaga

133981 tccttctcct tacttttttt cggaagaaag ggcggatccg aacaaaatcg atgaaacgga

134041 agaaatccaa gtgaatggaa aggaaaaaga attccacttt agatttacag agacagacta

134101 tcaaaataga cccgtttctg aagagtctta tctaatgaat atcaatgaaa atcaggataa

134161 ttcaagattg aaaatagttg atcaaaaaac tgaaaataaa gaactaatta aaaaaatcga

134221 tacaaaagaa aaatagaaga aaagataaga agaaatacga ccgcctccta gatatttgat

134281 cccttctcct aaaaagaaac tcatgacacc aactccattc gtaattccat caattattcg

134341 tctatcaaaa aaattagtta attgggccaa ttcccttacc cccctggcaa aaaatattgt

134401 ataaaaagaa tcgatataag cacgattata tgaccaatcg tatagggcgt ttataatttt

134461 atccaaacgt tttcttttcg gacctaactt tacaaatgaa tttattaagt caaaattttt

134521 taaagaggaa taaataggtt tatataaaaa ggacgctaga aatattccgc aataagctat

134581 accaactgac aaaagggaat ctttaaaaaa ttcataccaa cttgccgaat ccttcgactt

134641 ttgatgtaaa aggttaatag acggagctaa ccattttgat aatagatcca aatctgttct

134701 tttttgatta aaagaaattc ctagagatcc aacaactaaa gtaaatagga ctaatagaag

134761 taaagaaaat aacatcgtat tcgcggattc agaaggatag gaaaaggcct tttgattatc

134821 aaaatggaga atattaataa aagggcgtcc cccccttttt tttcttgcat tctcatcact

134881 tcgacatgtc tttttccaaa aaaaataaga actttcattt cttaataaag caaaattttg

134941 gttaattctt tttgaatacc ctttacccca tatggatatg gaatagaaag agttattttg

135001 gttgccacta taattttgga aattaatgtt taaatgcccc tcaaaagtaa gtaaataaat

135061 acgaaacata taaaatgccg ttaatcctgc cgtggcccac gctattattg cgaaaatcgg

135121 cgaatacaac caactatcat taagaatttc atcttttgac caaaaacaag caagcggcgg

135181 aataccacaa agagaaagtg tacctaataa aaaagaggtt ttggtaattg gtacatgttt

135241 tgttaaacca cccataagaa ccatattctg acttttatcc ggagaataac caacaagagt

135301 ttccattgaa tgaataacgg atccggaccc caaaaataat aatgctttgg aataagcatg

135361 agtaatcaaa tgaaataaag cacttcgata agaaccaatt cctagagcga acatcatata

135421 acccaattga gacattgtcg aataagctaa actcctctta atgtcttttt gagcaagagc

135481 taaagtagct cctaataata gtgtaattat gcctatcaac gcaattaaat tcattatata

135541 aggtataact acgaaaagag gaagaaggcg agctacaaga aaaatacccg ccgctaccat

135601 agtagcggca tgtataagag ccgaaatagg ggtggggccc tccatagcat caggtaacca

135661 cacatgaagg ggaaattgtg cagatttagc aactgcgccg gcaaataata gagcagcaca

135721 caaaataaca aatggaaaat tgacttcatt attagaaatc aagttattaa gtatttcgaa

135781 taaatctcga aattcaaaac tacctgttat ccaataaaaa cccaaaattc ctaataataa

135841 accaaaatcc cctacacgat tagttacaaa cgctttttga caagcatttg ccgcaggagg

135901 tcgtgtgaac caaaacccta ttaatagata ggaacacatt ccaaccaatt cccaaaaaat

135961 ataaatttgt atcaaatttg aactagtaac taaccccaac atggaagtac tgaaaaaact

136021 catataagca aaaaatctca agtatccttg atcgtgagcc atataattat cactataaat

136081 aagaaccata attccaacag tagtgattaa catcaacata atagaagtaa gtggatcaat

136141 caagcagcca aattctaaag aaaaatcatt atcgagggtc caagaccata catattgata

136201 gatataactg ctatttattt gctgaataga cagattagtt gaaaaaatca tgactatact

136261 taacaataaa atacttggaa aagcccacat acgatgaaga ttttttgttg ctgtcggaaa

136321 aataacaagt cccactccta ttaatatagg aactagaagt ggaacgaaag gtaaaataca

136381 cgcatattga tatgtctgtt ccataaaaaa aaggttttta aaatttttaa ttaatattaa

136441 cggtttccaa ttcacccgac cttccctctt ttgaaaggag ttaagaaaaa aatgaaaata

136501 tgtactaaat atgtactaac ttaaataaca tttttgaatt gttatttctt cttatttatt

136561 cgttctgaat ttctgctaaa tacttcaaat attcaaatca agaagttaca attggtcaaa

136621 tcatatgaaa gaaatagatt aattaccaat ttccttccaa tttgcaaatt caagtcaaat

136681 tgggcatatt tttcccggat ttgacaagtt attaaaaatc ttattttttt ctatttttag

136741 aaagatttta tgctattttg ccatactgtt cacccgtctt atctattctt ttctattttt

136801 gacaaaaaaa tattttctag aaaagaacta catagtgaat cagaaaagct catatttttt

136861 tgaacaatag atgtctttca catccagcta taccaatgag taatctctta atttttattt

136921 aaatggcagt tccaaaaaaa cgtacttctg catcaaaaaa gcgtattcgt aaaaattttt

136981 ggaaaaagaa ggggtatcgg gcagcgttaa aagcattttc attaggtaaa tctctttcta

137041 ccgggaattc aaaaagtttt tttgtgcgac aaacaaataa actaagtaat aaaacataac

137101 acgttggaat aatctaaacc gacctgactc aaaattggag tcatttcatt tcaaaaatca

137161 aattcccgaa ttccattcct cgtcgaagga aatggaattc gttcggctct tatagcatat

137221 ggagaataag aatttttctg tttagcttac ccaattcaaa ttgggtaatt atgtgtcaat

137281 ttttaatgat gtaaaatagg ttcttttttt gttcatcaac cgttaattaa aaaaaatatt

137341 tttggggggg agggttctat tccttaattc ctagtaggat ttacaagagt tgagtcggga

137401 agagtataaa atacaaaata aaacaaaaat actaaatgaa actaatgaac cttcaaatac

137461 ctatttgaac aaatttttat tgatcaattt gaatctttcc taaaaaatat tgcactcaat

137521 taaattcgta aatctagacg attatttatt cataggttat tatgaggtca agacaggccg

137581 ctatggtgaa atcggtagac acgctgctct taggaagcag tgctagagca tctcggttcg

137641 agtccgagtg gcggcatagc atctcttaaa aaaagaaaat agatcctata ataaactcaa

137701 ttgctaattt cgattttata attaaggaac tctttatttt atgatatttt caatcttaga

137761 gcatatatta actcatattt ctttttcgat cgtttcaatt ataattacaa ttcatttgat

137821 aaccttttta gtcgatgaaa tcgtaaaact agatgattca tccaaaacgg gcatgataat

137881 gacttttttc tgtataacag gattattaat ttctcgttgg atttattcga gacattttcc

137941 attaagtgat ttatacgaat cgttaatctt tctttcgtgg agtttttcct ttatttatat

138001 agtttcatat ttcaaaaaaa accaaaatat tctaagcaca ataattggcc caagtgctct

138061 tttttcccag ggctttgcta cttcaggtct tttaactgaa atacacgaat cgacaatctt

138121 agtacccgct cttcaatccg agtggctaat aatgcacgta agtatgatga tattaggcta

138181 tgcggccctt ttatgtggat cattattatc agtatcactt ctagtcatta cagttagaaa

138241 aaagctttct cttttttcta cgagtaatca tttattgaat ttaaatgagt catttttcct

138301 tggtgaaatc gaatacatga atgaacaaag agatttttta caaaaaactt cttttttttt

138361 cgcaaggaat tattatagat cacagttgat tcaaaaattg gattattgga gttatcgagt

138421 tattagtcta ggatttatct ttttaaccat aggaattctt tctggagcag tatgggctaa

138481 cgaagcatgg ggatcctatt ggagttggga tccaaaggaa acttgggctt ttattacttg

138541 gatcatattt tcaatttatt tacatactcg aacaaatata aaacgaaaga gtataaattc

138601 agcaattgtg gcgtctattg gatttcttat aatttggata tgctattttg gagtcaatct

138661 attaggaata ggactacata gttatggttc atttacatta acatctaatt gaattcaaaa

138721 aacgaaaaag gggggttatt acaaatagga ataaaagggt gtacaacgta gatcagataa

138781 aaaaactttg tattaattgg cgagagcctg tcgaatcata tatgattcga caggctcttt

138841 gtcattgtac cattttacaa cgaacgcttt tgtaaataat aagatttata aattatctaa

138901 aaaaagaatt agatagaata acttcaacct tttcaactga tagtgaaaga acgaaatcag

138961 ggtacatacc aataccgagt acgggtaaaa aaatagaaac cgaaagaaat aactctcgcg

139021 gacctgaatc aaaaaaataa gagtcaggcc cattaaatat cttgtatcca taaaacatct

139081 gtcgtaacat agataataaa taaataggag ttaatatcat cccaattgcc attacaaaaa

139141 taattgggag ttttgtcatt aaaagatatt ttggactagt aattagtcca aaaaaaacta

139201 ttaattcagc aacaaaacca ctcatgccag gtaatgcaag ggaggccatc gaaaagctac

139261 tgaacatcgt gaatattttt ggcattggaa tacctattcc gcccatttcg tcaagataaa

139321 caagacgtat tctatcataa gttgttccag ccaagaaaaa aagcgccgcg ccaataaatc

139381 catgagagat tatttgtaaa agggctccgt tgagtcccat atctgtgata gaaccaattc

139441 ctataattat aaaacccata tgagatacag aggaataggc tattcttttt tttaaattcc

139501 gttggccgag agatgttgaa gccgcataga ttatttgcat tgcgcctact accattaacc

139561 aaggggaaaa tatcgaatga gcatgaggaa ataattccat attgatccga actaatccat

139621 acgctcccat ttttaataag attccggcta gaagcataca agtactataa tgtgcttctc

139681 catgggtatc gggtaaccat atatgtaggg gtatgattgg caatttgaca gcaaaagcaa

139741 gaaaaaatcc aatataaaat agtatttcca agttcacagg ataggaccga ttggctaata

139801 tttcaaaatt taatgttggt tcagtagaac catataaacc gatacccaga actcccatta

139861 aaagaaaaac agaacccccc gccgtgtaca aaataaattt tgtagctgag tacagacgtt

139921 tttttcctcc ccacatcgat acaagtagat aaacgggaat taattctaac tcccacatga

139981 ggaaaaaaag taaaagatct ctagaagaaa ataatcctat ttgcccactg tacattgcta

140041 acatcaggaa atgaaataat cgagaatctc gagtaaccgg ccaagccgct aaagtagcta

140101 aagtggtgat gaatcccgtc agtaaaatgg gtcctatgga tagtccgtct attcctaatc

140161 tccaatggaa atccaaaaaa cggatccatt tataatcctc cattagttga attaatggat

140221 catccgattg aaaatgatag cagaatgcat aagtcgttag aagaagttct aatatacaca

140281 tgcatatagt ataccagcgt attactctat ttcccctgtg tggaagaaaa aaaatgaagc

140341 aacccgcaaa tattggtaaa actacaatta ttgttaagca aggaaaatgg ttcgtggtaa

140401 agacaagata cacttgggcc agaaaatccg tgctcaaatt caatttgatt ttgagcacgg

140461 attttttcga taaaaaaaaa gaaaatggat tcaagtagat ttttatggaa tgtatcaata

140521 agctagaccc atactgcgag ttgtttcatg ccataaataa acccgaacgc tcaaaaaatc

140581 cgttggacag gcggattcac atctcttaca accaacacag tcctcggtcc ttggagcaga

140641 ggcgatttgt ttagctttac atccgtccca gggtatcatt tctaatacat ccgtggggca

140701 cgcccggaca cattgagtac atcctataca tgtatcataa atctttactg aatgtgacat

140761 tagatctata cgtttttgaa cgccatcaat tttcggtcta gtaaactaac ttataaatga

140821 atcctatagt tagataccag acgaatcaat gagtgataag aatcaattta cttccaaatt

140881 gatttatgaa ggagggccaa aatactttga tttcttatgt tatttgtttt tgcaactacg

140941 attatatcct actatagaca tatcaaaaga catatcaaac ccgctaatgc gaattttcat

141001 ttatttatta ctattcaaaa ttagcattta tatgattaat actatttatt caacaaattg

141061 gattggttaa tacgaattga ttttctgtta cgataaattg atgaaacaat agccgatcca

141121 atagctgctt cagcggctgc aatagctata acaaaaattg agaaaatatc tcctcttaat

141181 tgacgattat caaaaatatc agaaaatgtg acaaaattga tattaactga atttaatata

141241 agttcaagac acataagggc tctaaccata tttcgacttg tgattaatcc atagatacca

141301 atagaaaata agtaggcact caaaacaagt atatgttcga gcatcattaa ccaactcctt

141361 atcaattttg attgatttta atctgaacaa taatttaatc gattcgattc taacaaatat

141421 gaaataatgg aataggttgg taagagatca atagaaaatt aaagtctttt tattctattt

141481 tttttaaaca aaaatgaaaa ttaacgaatt ataacattcc ttttgcgttg attctatttg

141541 aattactcat tttaaagatt tcttattgac gagctatagc aatagcacct attaaagcca

141601 ctaaaagaat tattgaaata agttcaaatg gaagaaaaaa atctgttgct aaatgaattc

141661 caatttgttg actattactt atcaaatctt gttctagaat ctgatttgat tttgtagtcc

141721 aaacgatccc ataccaagac gtatctggaa tagtagtaat tagtgaaata aaaagacttg

141781 tacaaactat tgaagtaact ccatccccaa cggtccaaag attaaaatct ttgtaatatt

141841 ctgacccatt catgaacatc acagcaaaaa tgattaaaac gtttatagct cctacataaa

141901 taaggagctg cgcagcagct acaaaatagg agttggatag aatatagaat aaggatatac

141961 aaaaaagaac ccatcccaac gaaaacgccg aataaattgg attcggaagt aatactactg

142021 ccaggcttcc taatataaga cccaatccca gaaagactaa aagaaaatca tgtattggtc

142081 caggtaaatc catttgattt tataaaaaaa atcgaaatat ttcatgcctt ttttgacctg

142141 accaggaaaa acgaagttat tcttttttag gatacttctt aattgaatga aatttgaacg

142201 gggttgattt ggattttgga ttttggattg atgtaaatac agttattgga ctactcttat

142261 tgtcgaagca atcgaagcag aggttcaaac tttctatttt caaatcatta ttcgaataga

142321 aatccatttt taatcaaaaa taagccgcga ttttcaccga ccagccgttc ttttgtattg

142381 accaagcaaa aacctcatca aaaacctcat tcctttcttt agatccacaa cctataaagc

142441 ttttgtgatt tgaatttgta aatgttttgt gaatcgaagg ttttatacat tttttatttg

142501 aggtaaattc gaagaaattg ttcgaattgt gtaatcttca attattgata ccggtaaccg

142561 acctaaagca atttgattat aatttaattc gtgacgatca taggcagaaa gttcatattc

142621 ttcagtcatt gataaacaat ttgttggaca atactcaacg caattaccgc aaaatataca

142681 gattccaaaa tcaatactgt aattaagtaa tcgtttcttt cgaatatcag tttgcaattt

142741 ccaatctaca acgggtagat ctataggaca tacacgaaca catacttcac aagcaatgca

142801 tttatcaaat tcaaagtgga ttcggccgcg gaaacgttcg gatgtgatca atttttcgta

142861 ggggtattga atagttacag gtaaacgatt cgcgtgggac aaggtgatca tgaaaccttg

142921 accaatgtac ctggcagctc gtattgtttg ttgaccggag ttcaagaact gagttagcat

142981 agggaacata tcggaatatc tataaataat ttgacttttt tctttctctt gtttgagaca

143041 agttataaag aaacgcctat tctatttcta ttcctttaca gtgaaagaag ttgggacgaa

143101 gttgttaata atagattacc tagagaaatt ggtaaaagga atttccatcc aagatttaaa

143161 agttggtcca ttctcagtct cggtaaagtc catcttgttg caatagaaat gaacaagaac

143221 aaataagttt tagctaatgt aataaagata ccgattattg ttccaaaaac ttgacttctt

143281 ttatttatgt caaaaagctc aggaaagaat aggtatggaa tagaaagatt ccaacccccc

143341 aagtaaagaa ctgttacaaa taatgaagaa acgagtagat ttagatacga agcaacataa

143401 aataaaccaa atttgatacc tgaatattcg gtttgataac ctgctactaa ttcttcttct

143461 gcttctggta aatcaaaagg taatctctca cactcggcta gagaagaaat cagaaaaaca

143521 ataaacccta taggttgacg ccataaattc catccccaaa aaccatattt tgattgcact

143581 tcaactatat caactgtact taaactgtta gataatccta gtcgacgcta acatcagcgt

143641 ttccgtcgct attccaaaac cgtacatgaa attttcacct catacggctc ctcataggtc

143701 acaaataaat ctaaggacct ttcaacatta tttctcttga tgtgtttgta ggatctatat

143761 agaatcaaat tattatccta gggcctagaa ttagaccaat gaaattctgt ctgctagatt

143821 tttaataaaa aagtgcttct gaattgatct catcttttaa gaattttgat ttttctttgt

143881 tgattaataa ctttatcctt gaatgaaaaa atacttttta gagaatatcg catcgatatt

143941 tcaacctatc actttctcac ttttgattac gaaaaagaat tacatattgc attacataag

144001 aataatttta ttgctgtaaa taaaatcgaa atagtcagga ataaaaaaaa gatctctttt

144061 tgcaattctc gtttgtggat tttagttcgt tcctattctc ctttctcaga aaaggcacat

144121 tacaaaaagt aaaagatttc gtcgtttttg atagttattt acttaattag tggataggaa

144181 catactctgg atcaaatcgt ggggagtact tcttaagaat ttctactaac ttaaagcccc

144241 aattcgaatt acttttctat aaaaaaatat cctgttggat aatttacaga atctccatta

144301 ctactccttt gtgtatcttt gtcttcctaa ccatccactc gttttttttt gaatctctca

144361 tttagggtaa tacatctatc gtaatagtat agtaaaaacc ccataaggtt gatcttttga

144421 acccgcttca agataaaatg actaatcaac caatcttggg ataaacagtc tcgactgttt

144481 acttttactt acttcttgta cataggaaat gagattaaat tcctttaaca gcaaaatttt

144541 aagccgtttt atttcactca tataactatc tggtttaatt catcaaccca ataatgaata

144601 aaaaaatgga tattccaacc atttcacttt cttgctagaa agaaaatcaa taggggacat

144661 tttatgtctc accgaatcac acgtagagat attgataata cacatagagt taatggtatt

144721 tcataactaa ttgattgagc agcagccctt aatccaccta aaaaggaata tttattattt

144781 gatccatatc ccgacataag aagtccaacg ggagcaagac ttgaaacagc aatccataaa

144841 aaaacaccaa tactaagatc ggctagaata aagcgatagc taaaaggaat tactgaataa

144901 cttagtaaaa tggatatgac tgctatggat ggcccgagac tgaataaacg agtatctcct

144961 cgagatggaa gaatattttc tttgaaaagt agttttgtac catctgctaa agcttgaaga

145021 attccgaaag gacccgcgta ttcgggtcca atacgttgtt gtatccctgc agatatttct

145081 ctttctaacc aaacaattac tagtacacct agtgtgattc ctaatacaag aattaaaata

145141 gggataagca tccatatgat cccatagact tcttttaagg atcccaatct gaaaaaagaa

145201 ttgatagctt gtatttttgt tgtatcaatt atcatttcaa cgatcaactt ctcccataat

145261 aatatctatg ctacctagta tcgtcataat atcagccaat ttcattcttt taactaactg

145321 cggaagaatt tgcaagttga taaaaccagg cggtctaatt ttccatctcc aaggaaaaac

145381 actccgatct cctatcagaa aaattcctaa ttctcctttt ggtgcttcga ctcttacata

145441 aagttcttgc ttggacaatt caaaagtagg agaaggtttt ttactaataa atcgatattc

145501 aaaatcattc cattccgggt ctttgattct atcaaagcgg cggctttcta aattctcata

145561 gggtcccccc ggaattcctt ccagagcctg ctggataatt tttatagatt ctgtcatttc

145621 gccaattcgt actaaatacc gagctaacga atccccctct ttttgccatt gaatctccca

145681 atcaaattcc tcgtaacact cataacgatc aactttacga agatcccatt gtattccgga

145741 agctcgtagc gttggtcccg ataaacccca gtttagtgcc tcttctcccc caataatgcc

145801 tacgccctca acccgttcta aaaaaatagg attccgtgta ataagctttt gatattcagc

145861 aaccccggtt aaaaaataat cgcaaaaatc caaacattta tctatccagc catgaggtag

145921 atcagcagcg actcctccga tacgaaaaaa attatgcatc atgcgcatgc cggtagccgc

145981 ttcaaatagg tcatatatca attctcgttc tcgaaaaata tagaagaaag gagtctgcgc

146041 cccaatatct gccataaaag gaccaagcca taacaaatgg gaagcgatac gactcaactc

146101 caacataata gctctgatat agctagccct tttaggtact tgaatattcc ctagctgttc

146161 gggcccgttt acggttattg cttctgtgaa catagtagct aaataatccc aacgtgttac

146221 ataaggcaaa tattgtataa ttgttcgatt ttccgcaatt ttctccatcc ctctatgtaa

146281 ataacccaat actggttcac agttaatgac atcttcacca tcgagagtaa cgatcagtcg

146341 aagaacacca tgcattgatg ggtggtgagg gcccatattg actatcatga ggtcttttct

146401 tgtagttggt ggaatcataa gtttttctcg agtcattctt ccatgcattg ctgaaagtaa

146461 aaagaaagaa gttcatcaaa atttaaacga ctaagctcaa aaagtctcac gcttcaaatt

146521 aacgagtttt tatctctcga atatccaact ccccaattaa ttctttatag cgccccctat

146581 ttatttttga caaataggcc agcagtcgtt gacgttttcc caaaattttt cgcaaacctc

146641 gctgagatga aaagtctttt ttgtgcaatt ccaaatgtga agtgagtttc cttattttag

146701 tggtgaaact gaatacttga aattcaacag accccctggt ttctttgttt tcgttttgaa

146761 aaataaccga aatcgatgaa tttttgacca taaaaaaacg ccccctgccc tttttacaga

146821 tatggatttt accgatcagt aataataatg ccattaattt gaatgtggta tatacaagaa

146881 tctaatcaaa atgtttttct gacctcctaa tttcctgaat tcaaatcaat caatccaatt

146941 ttgaattggt tttctatagg aatagaaagg gttggtacat ttttggatcg aagaatttag

147001 acctaaaata aagaaggttc cattgattca tttcgagatc gaattcagac attattcatt

147061 tgatattgga tactagaatg aaatgtgaga taagacatca gatctgtgta tttgtttgct

147121 ttcatatacc ctattatata tatagggtat aggatataca gaaaagtgta ttatcaaaaa

147181 attttcaatc gtggatacat ctgtatcctt aacataccga aacggctacc attattggta

147241 tcaaaccaat accgattcat acaagctaaa tcttcgaatc gataattagg ccaaagaaag

147301 aactttaatt tcattaattg atttttctct ctatcaagat ggttattgtc atgtaaaacc

147361 tggctgctgt tttttacgtt ccaaaatacc ggattttgat ctatataatt tctctttttt

147421 gaattgaaac aaattagaat tctcaatttt ctacgacgtc taaaggataa aatattttcg

147481 ggaacaagta aatcaaaatt attgttagaa gcatgtcctt gctcttggta tttttgatgc

147541 ttattcttat gaactaacga aatacccacg gtttgataca taacaaattg cccatctttt

147601 tgttcagaca aacgaatggg ttctagaatt aatactccct tcttcataaa ttctgaaaga

147661 gttaaattat cattaatcct tattatatcc aaattcattt gtttcttttg aatcgaggat

147721 ataataagtt ttgctgaagg tatcagttga cgcaggaggc aatatagatt gatattattg

147781 atcatttttt cattcaaagt ttcatcccat cgcaattgaa aaagcaaata acgtttcatg

147841 aacaaacggc gttctgcttt cgtgtattgt tttttctttt tccctttttt catgtttgat

147901 cttgcataat tttcttcaat atcttttggg ggtgagagaa cggatctccg ctttcctcca

147961 cttgtcggtt ctttttcttc ttgatttcga tgctttttat ttgatgctat caaaaaattg

148021 cccttttcat tgatcttttt agtttcactt cgatttaaat ttaaaagaag taatttgctt

148081 ggtataaacc aaggtttcat tttatatgtc ttataaatta acaaaaattc tgggaagaac

148141 caaagttcca gattggatat gggatgcttt agcatttttt cattcattcc catccaatcg

148201 taaaacccct tatgagagtt tggtaaattg atctctggga tcttaagata aaaaaaatct

148261 tttttagaaa ttatttgcga atttttagta cgaatttgag tattttgatt tctattggta

148321 tcgattatga tccaggcctc aatatcgact ttttgtataa gatcaaattg aaaaattttc

148381 caatcaaaat attttcgatc ggctggtttt tccatatgga tctttcctag atcattttta

148441 atagggatgt tcctcagcat atcaaaaaag ttttttttag gcgcgttata agaaatttct

148501 tggttcggat ttccttgaag gggagatcca taaaaaaagc attccgtttt cttttcataa

148561 tgaataaatt tatatgataa aagatcagat tgatagtatt ttagaaaatt ctctttttga

148621 ttagataatg aatatacttc caattgtttt tgttttttgg aactcattaa ggggttgttt

148681 tcatatgaat gccgtttgct aaaattttcc tttttagcta tacgatgctg acgaaatgta

148741 tttcgccatt tttctggtat taatctagac catccaatct gagataaatg gtattgataa

148801 tgtcctctta accagctttt ccatggattc atttcataac tcggaagttt gttatctgct

148861 gatttcgaat caagcattcc ttgtgtttca aaagaatcct ttattttagc cttaaggaaa

148921 aaggggattc gttgatattg aaccacaaat cttaacaagt tattaacttg gatttttcct

148981 aatttataaa atacatatgg ttgtggcacg taggataagt cagaaaaaat atgtgaattt

149041 gctttaatat tactaatatt ctcaagttcc tttcttataa ttatactcga attagacgga

149101 attgtagttt tctttttttt attaattctt tcttgttttc tttcattatt gtaaatggat

149161 ttatcaataa ttttttttgt taatttaaga aaaagttttg tatttattct ggcaatatta

149221 atgatatata aaaatatatc cgtgtatatt ttttcaatga aaaattttac aaaaggggat

149281 aatttacaga ttaatcgagc atttcttctt tttaacattt tgaacatttg ctgtttttct

149341 aattttttag cattagaact tgtaggacta agattattta ttcttggagt tacttttttt

149401 ttctcttttg tgattctttc tatttgattt cgaattgtac ttgttctatc agccagatcc

149461 tccatttttt tttctgtcaa tgaagagttt gtccaagtcg gagatgcaat ttgcatttga

149521 ctaaatgatt cgtgaatcat ttgattgttt attatggaat ctttttcttc tttaatttgg

149581 cttgatttat cgactccgac ttctcttaat ctaaataata gaattggatt tacttttgaa

149641 agttctttta ttattctttt tctaaatctt tttctaaaaa aaacactttt gataactcgt

149701 ttttttgttt cttttgaaac ttttcgaagt aattttattt tgattttgaa aactgttaga

149761 actcgaaaat acttcttttt gaattttcca attttttttg cgaattcctt taaaatgggt

149821 ttaaaaaagg aaggtttttt tcggggtgca caaaagggga attccgtttc cattccccaa

149881 actgttaaaa aacaagaatc acctccttct tttgtctttt tcattagatc tttctgagag

149941 gatcgtagtt tcgctttgtg ccaaggtttc agacagaaag gaaatacgat ttttatttga

150001 ataccttctg tcaaccaatt ttttggaaat tcggtttggg ataatggaat accattatag

150061 gtgcatttaa tatagatctc tttattccat tcttggaaat cctcagccca ttcaggacgt

150121 tgcaatagga atatccgtcc aatatttttg gcaattatca atgaagggaa tagaatatat

150181 tttctaaaaa tagattgagt tagtaacacg caacctctta ttccttgcgc aaatggaata

150241 cgattccaat cctctgcgac ttttattcgt gctttttcct ttcttttgtt gttttcttcg

150301 ttttcttttc tttttgtttg ttcttttgta tactctccaa tttggaatgc ttcccctttt

150361 tccaacccat ttttaaaaat tagtttaatc aatccggaaa tattaaaata agagggaggg

150421 gatttgtgta gtctctccaa aaaaagaggg gacttcacat ttgcttgaaa caattcgaaa

150481 ataaccactt tacgcctttg agcccgcata gaacctttga ttataccgcg ccgaaagtct

150541 gattgttgtg aatagcgcat taaagtcacg tcggtttttg tatcggacat tcttctatgc

150601 acagtcttag aagtaccctt ggcagcagtc aaaatgacta cacgcttgcc ccttcttgaa

150661 cgaatttgat gacctattgg tatgtcttct ttatattttc ttgattgttg ttctaaatcg

150721 gtgattaatt tgtatgaccg tcgggggacg tttttagtga tttcgtttat tctaatcgat

150781 tttctgtcaa ttttttgacc attaacatca gttacaattt tatttaaatt taataaatag

150841 ttaaaatatt ttgtttcttt ttgagaatct atttctcttt ctgaaaataa ataaaggcta

150901 ctcggattta gattcgtaga tcccgattct ttaacaaatt tacttatcaa agttaaaaaa

150961 ttaacaattt ctgttgataa tgattttttc tcaaatctat ttattttttg gtcaaattct

151021 tggtaatcag tatccggtag aagtataccg tgaattcgat ttatcccaaa ctgatctagg

151081 aaattttcta tcaaagtttt ttttcgggtt gagggcgaag ggcttttgta gattgttttc

151141 cgatatgatc cgttcaggaa aggatcatac ctttttgata agtattcttt tgtcgaatgg

151201 tcattccaca atcgagtccg tgtttcgagt atattcaaat aactattttc tttgtctaaa

151261 gctttaattc gatttagaaa ctcgttgtta aaactgttct ctttttgttt gttggtataa

151321 acccaagggt tatatagttc attaaatgaa aatttttcga ttgtaggcgg ggatatcctt

151381 gatatccttt tttttatcat ttcaaaaaaa atggataaac tcggaggata tgtaaaagag

151441 attctttctt ttccatcact ttgacatatg tcaaaaaaat attgtgacat ctcatttctg

151501 acagccttgt caaatcgttt attttttatg tatcgaaatg gtcgattcca tcgcttcgaa

151561 tcaaaaagaa tagttacaag aggtttgtca tcgatttttt taattagttc tttattttca

151621 gttttttgat caactattt

//
