# Supplementary material for: Complete chloroplast genome sequences of Phlomis fruticosa and Phlomoides strigosa and comparative analysis of the genus Phlomis sensu lato (Lamiaceae)
Source: Front Plant Sci. 2022 Oct 28;13:1022273. doi: 10.3389/fpls.2022.1022273 (PMC9650320; doi:10.3389/fpls.2022.1022273)
Supplement: Supplementary file 4 [file DataSheet_4.docx]

LOCUS Phlomoides strigosa 152432 bp DNA circular 20-MAY-2022

DEFINITION Phlomoides strigosa chloroplast.

ACCESSION

VERSION

KEYWORDS .

SOURCE chloroplast p.strgosa

ORGANISM p.strgosa

Unclassified.

REFERENCE 1 (bases 1 to 152432)

AUTHORS Author,U.

TITLE Your Publication

JOURNAL Unpublished

REFERENCE 2 (bases 1 to 152432)

AUTHORS Author,U.

TITLE Direct Submission

JOURNAL Submitted (20-MAY-2022) Your Department, Your Institute, Your

Address 1, City, State 12345, Country

FEATURES Location/Qualifiers

source 1..152432

/organism="p.strgosa"

/organelle="plastid:chloroplast"

/mol_type="genomic DNA"

misc_feature 1..83639

/note="large single copy (LSC)"

CDS 1..275

/gene="rpl2"

/codon_start=1

/transl_table=11

gene 328..606

/gene="rps19"

CDS 328..606

/gene="rps19"

/codon_start=1

/transl_table=11

/product="ribosomal protein S19"

/translation="MIRSLKKNPFVANHLLRKIDKLNKKAEKEIIVTWSRASTIVPTM

IGHTIAVHNGKEHLPIYITDRMVGHKLGEFVPTLNFQGHAKSDNRSRR"

gene 658..1039

/gene="rpl22"

CDS 658..1039

/gene="rpl22"

/codon_start=1

/transl_table=11

gene 666..1130

/gene="rpl22"

CDS 666..1130

/gene="rpl22"

/codon_start=1

/transl_table=11

/product="ribosomal protein L22"

/translation="MLKKKKTEVYALGRHISLSADKARRIIDQIRGRSYEETLMILEL

MPYKACYPIFKLVYSAAANASFNMGSNEANLVIRKAEVNESTASKKLKLRARGRSYAI

KKATCHITIVVKDISLDEYEEIAFYSLKNPRWKKTTMVYDDAYNSEVVWDKK"

gene 1115..1777

/gene="rps3"

CDS 1115..1777

/gene="rps3"

/codon_start=1

/transl_table=11

/product="ribosomal protein S3"

/translation="MGQKINPLGFRLGTTQSHHSLWFAQPKNYSEGLQEDQKIRDFIK

NYVQKNMRISSGAEGIARIEIQKRIDLIQVRIFMGFPKLLIESRPRGIEELQMNLQNE

FHYVNRKLNIAITRIAKPYGNPNILAEFIAGQLKNRVSFRKAMKKAIELTEQADTKGI

QVQIAGRIDGKEIARVEWIREGRVPLQTIRAKIDYCSYTVRTIYGVLGIKIWIFIDKG

EE"

gene 1924..3246

/gene="rpl16"

CDS join(1924..1932,2848..3246)

/gene="rpl16"

/codon_start=1

/transl_table=11

/product="ribosomal protein L16"

/translation="MLSPKRTRFRKQHRGRMKGISYRGNHICFGKYALQALEPAWITS

RQIEAGRRAMTRNARRGGKIWVRIFPDKPVTIRPAETRMGSGKGSPEYWVAVVKPGRI

LYEMGGVTENIARRAILIAASKMPIRTQFIISA"

exon 1924..1932

/gene="rpl16"

/number=1

intron 1933..2847

/gene="rpl16"

/number=1

exon 2848..3246

/gene="rpl16"

/number=2

gene 3373..3741

/gene="rpl14"

CDS 3373..3741

/gene="rpl14"

/codon_start=1

/transl_table=11

/product="ribosomal protein L14"

/translation="MIQPQTHLNVADNSGARKLMCIRILGASNRRYAHIGDVIVAVIK

EAVPNMPLEKSEVVRAVIVRTCKELKRDSGMIIRYDDNAAVVIDQEGNPKGTRIFGAI

PRELRQFNFTKIISLAPEVL"

gene 3914..4327

/gene="rps8"

CDS 3914..4327

/gene="rps8"

/codon_start=1

/transl_table=11

/product="ribosomal protein S8"

/translation="MGRDTIAEIITSIRNADMDRKRVVRIASTNITENIVKILFREGF

LENVRKHREKNKNFLVLTLRHNRRNRKRPHTCRNFLNLKRISRPGLRIYSNSQRIPRI

LGGMGIVILSTSRGIMTDREARLERIGGEILCYIW"

gene 4450..4683

/gene="infA"

CDS 4450..4683

/gene="infA"

/codon_start=1

/transl_table=11

/product="translational initiation factor 1"

/translation="MKEQKWIHEGLITESLPNGMFRVRLDNEDLIIGYVSGKIRRSFI

RILPGDKVKIEVSRYDSTRGRIIYRLRNKDSKD"

gene 4779..4892

/gene="rpl36"

CDS 4779..4892

/gene="rpl36"

/codon_start=1

/transl_table=11

/product="ribosomal protein L36"

/translation="MKIRASVRKICEKCRLIRRRGRIRVICSNPRHKQRQG"

gene 4995..5411

/gene="rps11"

CDS 4995..5411

/gene="rps11"

/codon_start=1

/transl_table=11

/product="ribosomal protein S11"

/translation="MAKAIPRIGSRKNVRIGSRKSARRIPKGVIHVQASFNNTIVTVT

DVRGRVVSWSSSGTCGFKGTRRGTPFAAQTAAANAIRTVVDQGMQRAEVMIKGPGLGR

DAALRAIRRSGILLTFVRDVTPMPHNGCRPPKKRRV"

gene 5483..6496

/gene="rpoA"

CDS 5483..6496

/gene="rpoA"

/codon_start=1

/transl_table=11

/product="RNA polymerase subunit alpha"

/translation="MVREKVTVSTRTLQWKCVESRTDSKRLYYGRFILSPLMKGQADT

IGIAMRRALLGEIEGTCITRVKSENVPHEYSTITGIQESVHEIIMNLKEIVLRSNLYG

TCGASICAMGPGYVTAQNMILPPYVEIVDNTQHIASLAEPINLCIGLEIERNRGYLIK

MPHTFQDGSYPIDAVFMPVRNVNHSIHSYENGNEKQEILFLEIWTNGSLTPKEALHEA

SRNLIDLFIPFLYKEEKNLPLEDNQYTLPLSPFTFHDKLDKVRKNKKKIALKSIFIDQ

SELSPRVYNCLKRSNIYTLLDLLNNSQEDLMKIEDFRLEDVKQILGILEKHFAIDLPK

NKF"

gene complement(6659..7872)

/gene="petD"

CDS complement(join(6659..7133,7865..7872))

/gene="petD"

/codon_start=1

/transl_table=11

/product="cytochrome b6/f subunit IV"

/translation="MGVTKKPDLNDPVLRAKLAKGMGHNYYGEPAWPNDLLYIFPVVI

LGTIACNVGLAVLEPSMIGEPADPFATPLEILPEWYFFPVFQILRTVPNKLLGVLLMV

SVPAGLLTVPFLENVNKFQNPFRRPVATTVFLIGTAVALWLGIGATLPIDKSLTLGLF

"

exon complement(6659..7133)

/gene="petD"

/number=2

intron complement(7134..7864)

/gene="petD"

/number=1

exon complement(7865..7872)

/gene="petD"

/number=1

gene complement(8062..9445)

/gene="petB"

CDS complement(join(8062..8703,9440..9445))

/gene="petB"

/codon_start=1

/transl_table=11

/product="cytochrome b6"

/translation="MSKVYDWFEERLEIQAIADDITSKYVPPHVNIFYCLGGITLTCF

LVQVATGFAMTFYYRPTVTEAFASVQYIMTEANFGWLIRSVHRWSASMMVLMMILHVF

RVYLTGGFKKPRELTWVTGVVLGVLTASFGVTGYSLPRDQIGYWAVKIVTGVPEAIPV

IGSPVVELLRGSASVGQSTLTRFYSLHTFVLPLLTAVFMLMHFPMIRKQGISGPL"

exon complement(8062..8703)

/gene="petB"

/number=2

intron complement(8704..9439)

/gene="petB"

/number=1

exon complement(9440..9445)

/gene="petB"

/number=1

gene complement(9570..9791)

/gene="psbH"

CDS complement(9570..9791)

/gene="psbH"

/codon_start=1

/transl_table=11

/product="photosystem II subunit H"

/translation="MATQTVENSSKSGPRRTVVGDLLKPLNSEYGKVAPGWGTTPLMG

VAMALFAVFLSIILEIYNSSVLLDGISMN"

gene 9895..10026

/gene="pbf1"

CDS 9895..10026

/gene="pbf1"

/codon_start=1

/transl_table=11

/product="photosystem biogenesis factor 1"

/translation="METATLVAIFISGLLVSFTGYALYTAFGQPSQQLRDPFEEHGD"

gene complement(10100..10201)

/gene="psbT"

CDS complement(10100..10201)

/gene="psbT"

/codon_start=1

/transl_table=11

/product="photosystem II subunit T"

/translation="MEALVYTFLLVSTLGIIFFAIFFREPPKVPTKK"

gene complement(10396..11922)

/gene="psbB"

CDS complement(10396..11922)

/gene="psbB"

/codon_start=1

/transl_table=11

/product="photosystem II 47 kDa protein"

/translation="MGLPWYRVHTVVLNDPGRLLSVHIMHTALVAGWAGSMALYELAV

FDPSDPVLDPMWRQGMFVIPFMTRLGITNSWGGWSITGGTVTNTGVWSYEGVAGAHIL

FSGLCFLAAIWHWVYWDLEIFSDERTGKPSLDLPKIFGIHLFLSGVACFGFGAFHVTG

LYGPGIWVSDPYGLTGKVQPINPAWGVEGFDPFVPGGIASHHIAAGTLGILAGLFHLS

VRPPQRLYKGLRMGNIETVLSSSIAAVFFAAFVVAGTMWYGSATTPIELFGPTRYQWD

QGYFQQEIYRRVRAGLAENQSLSEAWSKIPEKLAFYDYIGNNPAKGGLFRAGSMDSGD

GIAVGWLGHPIFRDKEGRELFVRRMPTFFETFPVVLVDGDGIVRADVPFRRAESKYSV

EQVGVTVEFYGGELNGVSYSDPATVKKYARRAQLGEIFELDRATLKSDGVFRSSPRGW

FTFGHASFALLFFFGHIWHGARTLFRDVFAGIDPDLDAQVEFGAFQKLGDPTTRRQAV

"

gene 12359..14227

/gene="clpP1"

CDS join(12359..12429,13089..13383,14003..14227)

/gene="clpP1"

/codon_start=1

/transl_table=11

/product="ATP-dependent Clp protease proteolytic subunit

1"

/translation="MPIGVPKVPFRSPGEEDASWVDVYNRLYRERLLFLGQEVDSEIS

NQLIGLMVYLSIEDDTKDLYLFINSPGGWVIPGVAVYDTMQFVRPEVHTICMGLAASM

GSFILVGGEITKRLAFPHAWVMIHQPASSFYEAQTGEFILEAEELLKLRETLTRVYVQ

RTGKPLWVVSEDMERDVFMSATEAQAYGIVDLVAVE"

CDS join(12359..12429,13089..13380,14000..14227)

/gene="clpP1"

/codon_start=1

/transl_table=11

/product="ATP-dependent Clp protease proteolytic subunit

1"

/translation="MPIGVPKVPFRSPGEEDASWVDVYNRLYRERLLFLGQEVDSEIS

NQLIGLMVYLSIEDDTKDLYLFINSPGGWVIPGVAVYDTMQFVRPEVHTICMGLAASM

GSFILVGGEITKRLAFPHARVMIHQPASSFYEAQTGEFILEAEELLKLRETLTRVYVQ

RTGKPLWVVSEDMERDVFMSATEAQAYGIVDLVAVE"

exon 12359..12429

/gene="clpP1"

/number=1

intron 12430..13088

/gene="clpP1"

/number=1

exon 13089..13383

/gene="clpP1"

/number=2

exon 13089..13380

/gene="clpP1"

/number=2

intron 13381..13999

/gene="clpP1"

/number=2

intron 13384..14002

/gene="clpP1"

/number=2

gene 13978..14245

/gene="clpP1"

CDS 13978..14245

/gene="clpP1"

/codon_start=1

/transl_table=11

exon 14000..14227

/gene="clpP1"

/number=3

exon 14003..14227

/gene="clpP1"

/number=3

gene 14354..139185

/gene="rps12"

gene join(14354..14467,138392..139185)

/gene="rps12"

/trans_splicing

CDS join(14354..14467,138392..138624,139161..139185)

/gene="rps12"

/codon_start=1

/transl_table=11

/product="ribosomal protein S12"

/translation="MPTIKQLIRNTRQPIRNVTKSPALGGCPQRRGTCTRVYTITPKK

PNSALRKVARVRLTSGFEITAYIPGIGHNSQEHSSVLVRGGRVKDLPGVRYHIVRGTL

DAVGVKDRQQGRSRYGVKKPK"

CDS join(14354..14467,138392..138623,139160..139185)

/gene="rps12"

/trans_splicing

/codon_start=1

/transl_table=11

/product="ribosomal protein S12"

/translation="MPTIKQLIRNTRQPIRNVTKSPALGGCPQRRGTCTRVYTITPKK

PNSALRKVARVRLTSGFEITAYIPGIGHNSQEHSSVLVRGGRVKDLPGVRYHIVRGTL

DAVGVKDRQQGRSKYGVKKPK"

gene join(complement(96887..97680),14354..14467)

/gene="rps12"

/trans_splicing

CDS join(14354..14467,complement(97449..97680),

complement(96887..96912))

/gene="rps12"

/trans_splicing

/codon_start=1

/transl_table=11

/product="ribosomal protein S12"

/translation="MPTIKQLIRNTRQPIRNVTKSPALGGCPQRRGTCTRVYTITPKK

PNSALRKVARVRLTSGFEITAYIPGIGHNSQEHSSVLVRGGRVKDLPGVRYHIVRGTL

DAVGVKDRQQGRSKYGVKKPK"

exon 14354..14467

/gene="rps12"

/number=1

intron 14468..138391

/gene="rps12"

/number=1

gene 15229..15615

/gene="rpl20"

CDS 15229..15615

/gene="rpl20"

/codon_start=1

/transl_table=11

/product="ribosomal protein L20"

/translation="MTRIKRGYIARRRRTKIRLFASSFRGSHSRLTRTITQQKIRALV

SAHRDRDKQKRNFRRLWITRINAVIREGRVFYSYSKLIHDLYKKQLLLNRKILAQMAI

SNRNCLYMISNEIRKKVDWKEYTGII"

gene complement(15838..16143)

/gene="rps18"

CDS complement(15838..16143)

/gene="rps18"

/codon_start=1

/transl_table=11

/product="ribosomal protein S18"

/translation="MDKSKRSFLKSKRSFRRRLPPIQSGDRIDYRNMSLISRFISEQG

KILSRRVNRLTLKQQRLITIAIKQARILSLLPFLNNEKQFERIESTTRTTGLRTRNK"

gene complement(16291..16491)

/gene="rpl33"

CDS complement(16291..16491)

/gene="rpl33"

/codon_start=1

/transl_table=11

/product="ribosomal protein L33"

/translation="MAKRKDARVTVILECTGCIQNGVKKVSTGISRYITQKNRHNMPN

RLELRKFCPYCYKHMIHVEIKK"

gene complement(16964..17098)

/gene="psaJ"

CDS complement(16964..17098)

/gene="psaJ"

/codon_start=1

/transl_table=11

/product="photosystem I subunit J"

/translation="MRDLKTYLSVAPVLSTLWFGALAGLLIEINRFFPDALTFPFFSF

"

gene 17373..17446

/gene="trnP-UGG"

tRNA 17373..17446

/gene="trnP-UGG"

/product="tRNA-Pro"

gene 17610..17683

/gene="trnW-CCA"

tRNA 17610..17683

/gene="trnW-CCA"

/product="tRNA-Trp"

gene complement(17809..17922)

/gene="petG"

CDS complement(17809..17922)

/gene="petG"

/codon_start=1

/transl_table=11

/product="cytochrome b6/f subunit G"

/translation="MIEVFLFGIVLGLIPITLAGLFVTAYLQYRRGDQLDL"

gene complement(18096..18191)

/gene="petL"

CDS complement(18096..18191)

/gene="petL"

/codon_start=1

/transl_table=11

/product="cytochrome b6/f subunit L"

/translation="MLTITSYFGFLLVALTITSALFIGLSKIRLI"

gene 19122..19373

/gene="psbE"

CDS 19122..19373

/gene="psbE"

/codon_start=1

/transl_table=11

/product="cytochrome b559 subunit alpha"

/translation="MSGSTGERSFADIITSIRYWVIHSITIPSLFIAGWLFVSTGLAY

DVFGSPRPNEYFTESRQGIPLITGRFDPLEQLDEFSRSF"

gene 19388..19507

/gene="psbF"

CDS 19388..19507

/gene="psbF"

/codon_start=1

/transl_table=11

/product="cytochrome b559 subunit beta"

/translation="MTIDRTYPIFTVRWLAVHGLAVPTVFFLGSISAMQFIQR"

gene 19531..19647

/gene="psbL"

CDS 19531..19647

/gene="psbL"

/codon_start=1

/transl_table=11

/product="photosystem II subunit L"

/translation="MTQSNPNEQNVELNRTSLYWGLLLIFVLAVLFSNYFFN"

gene 19785..19907

/gene="psbJ"

CDS 19785..19907

/gene="psbJ"

/codon_start=1

/transl_table=11

/product="photosystem II subunit J"

/translation="MADTTGRIPLWIIGTAAGILVIGLIGIFFYGSYSGLGSSL"

gene complement(20957..21919)

/gene="petA"

CDS complement(20957..21919)

/gene="petA"

/codon_start=1

/transl_table=11

/product="cytochrome f"

/translation="MQTRKTFSWIKEHITRSISVSLMIYIITRTSISSAYPIFAQQGY

ENPREATGRIVCANCHLANKPVDIEVPQAVLPDTVFEAVVRIPYDKQVKQVLANGKKG

GLNAGAVLILPEGFELAPPDRISPEMKEKIGNLSFQSYRPNKQNILVIGPVPGQKYSE

ITFPILSPDPATKKDVHFLKYPIYVGGNRGRGQIYPDGSKSNNTVYNATGAGIVSKII

RKEKGGYEITITDPSDGREVVDIIPPGPEVLVSEGESIKLDQPLTSNPNVGGFGQGDA

EIVLQDPLRVQGLLVFLASVILAQIFLVLKKKQFEKVQLAEMNF"

gene complement(22136..22825)

/gene="cemA"

CDS complement(22136..22825)

/gene="cemA"

/codon_start=1

/transl_table=11

/product="envelope membrane carbon uptake protein"

/translation="MAKKKAFTPLLYLASIVFLPWWISLSFTKSMESWVTSWSNTGQS

ENFLNDMQEKSLLEKFIELEEILFLEEMIKEYSETHLQKLPIEIHKETIQLIKIHNED

RIHTILHFSTNLICFVILTGYSILGNQELVILNSWTQEFLYNLSDTVKAFLILLLTDL

CIGFHSPHGWELMIGSVYKDFGFGHNDQIISGLVSTFPVILDTIFKYWIFHYLNRVSP

SLVVIYHSMND"

gene complement(23386..23940)

/gene="pafII"

CDS complement(23386..23940)

/gene="pafII"

/codon_start=1

/transl_table=11

/product="photosystem I assembly factor II"

/translation="MSWRSEHIWIELITGSRKLSNFCWALVVFLGSLGFLLVGTSSYL

GRNLISFVPSQQILFFPQGIVMSFYGIAGLFISSYLWCTISWNVGSGYDQFDRKEGMV

CIFRWGFPGKNRRIFLRFRIKDIQSVRIEVKEGIYARRILYMDIRGRGAIPLTRTDEN

LTPREIEQKAAELAYFLRVPIEVF"

gene complement(24373..24483)

/gene="psaI"

CDS complement(24373..24483)

/gene="psaI"

/codon_start=1

/transl_table=11

/product="photosystem I subunit I"

/translation="MTTFQFPSIFVPLVGLVFPAIAMASLFLHVQKNKIV"

gene complement(25180..26646)

/gene="accD"

CDS complement(25180..26646)

/gene="accD"

/codon_start=1

/transl_table=11

/product="acetyl-CoA carboxylase subunit beta"

/translation="MERCWFNSMVFKKELERRYGIKKLTDNLGPMENTSEGEDPNRKG

RAKNIHSWSGRDNSSYSNVDLLFGAKDIRNFISDDTFLVRDSNGDSYSIYFDIENQIF

EIDNDHSFRSELESSFSSYRNSNYMNNGSTNEDSLYTRYMYDTQSSWNNHITSCIDSY

LQSQICIDTPTVSDSSDSYISRCVFQKRKTRSEPSIRTRAKSSDLTLRASDLDETQKY

KQLWVQCENCYGLNYKKLLKSQINICEQCGYHLKMSSSERIEVLIDPGTWDPMDEDMV

SGDPIGFHSEEETYKDRIDFYQRNIGLTEAVQTGVGQLNGIPVAIGVMDFKFMGGSMG

SVVGEKITRLIEYATNQFIPLIIVCASGGARMQEGSLSLMQMAKISSALFDYQSNKKL

LYVSILTSPTTGGVTASFGMLGDIIIAEPNSYIAFAGKRVIEQTLNKTIPEGSQASEY

LFQKGLFDLIVPRNLLKSVLSELFKLHAFFPLNSNSMR"

gene complement(27316..28761)

/gene="rbcL"

CDS complement(27316..28761)

/gene="rbcL"

/codon_start=1

/transl_table=11

/product="ribulose-1,5-bisphosphate carboxylase/oxygenase

large subunit"

/translation="MSPQTETKASVGFKAGVKEYKLTYYTPEYETKDTDILAAFRVTP

QPGVPPEEAGAAVAAESSTGTWTTVWTDGLTSLDRYKGRCYHIEPVLGEKDQYICYVA

YPLDLFEEGSVTNMFTSIVGNVFGFKALRALRLEDLRVPPAYIKTFQGPPHGIQVERD

KLNKYGRPLLGCTIKPKLGLSAKNYGRAVYECLRGGLDFTKDDENVNSQPFMRWRDRF

LFCAEAIYKAQAETGEIKGHYLNATAGTCEEMIKRAVFARELGVPIVMHDYLTGGFTA

NTSLSHYCRDNGLLLHIHRAMHAVIDRQKNHGMHFRVLAKALRLSGGDHIHSGTVVGK

LEGEREITLGFVDLLRDDYIEKDRSRGIYFTQDWVSLPGVIPVASGGIHVWHMPALTE

IFGDDSVLQFGGGTLGHPWGNAPGAVANRVALEACVQARNEGRDLAAEGNAIIREACK

WSPELAAACEVWKEIKFEFKPVDTLDEEKKN"

gene 29571..31067

/gene="atpB"

CDS 29571..31067

/gene="atpB"

/codon_start=1

/transl_table=11

/product="CF1 subunit beta"

/translation="MIINPTTSGSGVSTLEKKNQGRIIQIIGPVLDVAFPPGKMPNIY

NALVVKGQDTAGQPINVTCEVQQLLGNNRVRAVAMSATDGLMRGMEVIDTGSPLSVPV

GGATLGRIFNVLGEPVDNLGPVDTRTTFPIHRSAPAFIQLDTKLSIFETGIKVVDLLA

PYRRGGKIGLFGGAGVGKTVLIMELINNIAKAHGGVSVFGGVGERTREGNDLYMEMKE

SGVINEENIAESKVALVYGQMNEPPGARMRVGLTALTMAEYFRDVNEQDVLLFIDNIF

RFVQAGSEVSALLGRMPSAVGYQPTLSTEMGSLQERITSTKEGSITSIQAVYVPADDL

TDPAPATTFAHLDATTVLSRGLAAKGIYPAVDPLDSTSTMLQPRIVGEEHYEIAQRVK

QTLQRYKELQDIIAILGLDELSEEDRLTVARARKIERFLSQPFFVAEVFTGSPGKYVG

LAETIRGFQLILSGELDGLPEQAFYLVGNIDEAAAKAMNLEMESNLQK"

gene 31064..31465

/gene="atpE"

CDS 31064..31465

/gene="atpE"

/codon_start=1

/transl_table=11

/product="CF1 subunit epsilon"

/translation="MTLNLCVLTPNRIVWDSEVKEIILSTNSGQIGVLANHAPVATAV

DIGILRIRLKDQWLTMALMGGFARIGNNEITVLVNDAEKGSDIDPQEAQQTLEIAEAN

LRKAEGKRQIIEANLALRRARTRVEAVNVIS"

gene complement(31688..31760)

/gene="trnM-CAU"

tRNA complement(31688..31760)

/gene="trnM-CAU"

/product="tRNA-Met"

gene 31949..32601

/gene="trnV-UAC"

tRNA join(31949..31984,32565..32601)

/gene="trnV-UAC"

/product="tRNA-Val"

exon 31949..31984

/gene="trnV-UAC"

/number=1

intron 31985..32564

/gene="trnV-UAC"

/number=1

exon 32565..32601

/gene="trnV-UAC"

/number=2

gene 33556..33918

/gene="ndhC"

CDS 33556..33918

/gene="ndhC"

/codon_start=1

/transl_table=11

/product="NADH dehydrogenase subunit C"

/translation="MFLLYEYDIFWAFLIISSLIPILAFFISGILAPIRKGPEKLSSY

ESGIEPMGDAWLQFRIRYYMFALVFVVFDVETVFLYPWAMSFDVLGVSVFIEALIFVL

ILIVGLVYAWRKGALEWS"

gene 33909..34649

/gene="ndhK"

CDS 33909..34649

/gene="ndhK"

/codon_start=1

/transl_table=11

/product="NADH dehydrogenase subunit K"

/translation="MVLAPEYSDNKKKGEKKIEKVMNPIEFPLLNRTAEISVISTTLN

DLSNWSRLSSLWPLLYGTSCCFIEFASLIGSRFDFDRYGLVPRSSPRQADLILTAGTV

TMKMAPSLVRLYEQMPDPKYVIAMGACTITGGMFSTDSYSTVRGVDKLIPVDVYLPGC

PPKPEAVIDAITKLRKKISREIYENRIRSQQANRCFTTNHKFRVGRSMNTGNYDQRFL

YQPPSTSEIPTENFFEYKSSVSSHELVN"

gene 33972..34649

/gene="ndhK"

CDS 33972..34649

/gene="ndhK"

/codon_start=1

/transl_table=11

/product="NADH dehydrogenase subunit K"

/translation="MNPIEFPLLNRTAEISVISTTLNDLSNWSRLSSLWPLLYGTSCC

FIEFASLIGSRFDFDRYGLVPRSSPRQADLILTAGTVTMKMAPSLVRLYEQMPDPKYV

IAMGACTITGGMFSTDSYSTVRGVDKLIPVDVYLPGCPPKPEAVIDAITKLRKKISRE

IYENRIRSQQANRCFTTNHKFRVGRSMNTGNYDQRFLYQPPSTSEIPTENFFEYKSSV

SSHELVN"

gene 34750..35226

/gene="ndhJ"

CDS 34750..35226

/gene="ndhJ"

/codon_start=1

/transl_table=11

/product="NADH dehydrogenase subunit J"

/translation="MQGRLSAWLVKHGIIHRSLGFDYQGIETLQIKPEDWHSIAVILY

VYGYNYLRSQCAYDVAPGGLLASVYHLTRIEYGVDQPEEVCIKVFTSRRNPRIPSVFW

VWKSVDFQERESYDMLGISYDNHPRLKRILMPESWIGWPLRKDYIAPNFYEIQDAH"

gene complement(35898..35970)

/gene="trnF-GAA"

tRNA complement(35898..35970)

/gene="trnF-GAA"

/product="tRNA-Phe"

gene complement(36267..36833)

/gene="trnL-UAA"

tRNA complement(join(36267..36316,36799..36833))

/gene="trnL-UAA"

/product="tRNA-Leu"

exon complement(36267..36316)

/gene="trnL-UAA"

/number=2

intron complement(36317..36798)

/gene="trnL-UAA"

/number=1

exon complement(36799..36833)

/gene="trnL-UAA"

/number=1

gene 37503..37575

/gene="trnT-UGU"

tRNA 37503..37575

/gene="trnT-UGU"

/product="tRNA-Thr"

gene 37975..38580

/gene="rps4"

CDS 37975..38580

/gene="rps4"

/codon_start=1

/transl_table=11

/product="ribosomal protein S4"

/translation="MSRYRGPRFKKIRRLGALPGLTNKRPKAGSDLRNQSRSGKKSQY

RIRLEEKQKLRFHYGLTERQLLKYVRIAGKARGSTGQVLLQLLEMRLDNILFRLGMAS

TIPAARQLVNHRHILVNGRIVDIPSYRCKPRDIITGKDEQKSRTLIQNSLNSSPQAEV

PNHLTLHPFQYKGLVNQIIDSKWVGLKINELLVVEYYSRQT"

gene complement(38865..38951)

/gene="trnS-GGA"

tRNA complement(38865..38951)

/gene="trnS-GGA"

/product="tRNA-Ser"

gene 39268..41209

/gene="pafI"

CDS join(39268..39391,40101..40330,41057..41209)

/gene="pafI"

/codon_start=1

/transl_table=11

/product="photosystem I assembly factor I"

/translation="MPRSRINGNFIDKTFSIVANILLQIIPTTSGEREAFTYYRDGMS

AQSEGNYAEALQNYYEAMRLEIDPYDRSYILYNIGLIHTSNGEHTKALEYYFRALERN

PFLPQAFNNMAVICHYRGEQAILQGDSEIAEAWFDQAAEYWKQAIALTPGNYIEAHNW

LKITRRFE"

CDS join(39268..39391,40101..40328,41055..41209)

/gene="pafI"

/codon_start=1

/transl_table=11

/product="photosystem I assembly factor I"

/translation="MPRSRINGNFIDKTFSIVANILLQIIPTTSGEREAFTYYRDGMS

AQSEGNYAEALQNYYEAMRLEIDPYDRSYILYNIGLIHTSNGEHTKALEYYFRALERN

PFLPQAFNNMAVICHYRGEQAILQGDSEIAEAWFDQAAEYWKQAIALTPGNYIEAHNW

LKITRRFE"

exon 39268..39391

/gene="pafI"

/number=1

intron 39392..40100

/gene="pafI"

/number=1

exon 40101..40330

/gene="pafI"

/number=2

exon 40101..40328

/gene="pafI"

/number=2

intron 40329..41054

/gene="pafI"

/number=2

intron 40331..41056

/gene="pafI"

/number=2

exon 41055..41209

/gene="pafI"

/number=3

exon 41057..41209

/gene="pafI"

/number=3

gene 41945..44197

/gene="psaA"

CDS 41945..44197

/gene="psaA"

/codon_start=1

/transl_table=11

/product="photosystem I P700 apoprotein A1"

/translation="MIIRSPEPEVKILVDKDPVKTSFEQWAKPGHFSRTIAKGPDTTT

WIWNLHADAHDFDSHTSDLEEISRKVFSAHFGQLSIIFLWLSGMYFHGARFSNYEAWL

SDPTHIGPSAQVVWPIVGQEILNGDVGGGFRGIQITSGFFQIWRASGITNELQLYCTA

IGALVFAALMLFAGWFHYHKAAPKLAWFQDVESMLNHHLAGLLGLGSLSWAGHQVHVS

LPINQFLNAGVDPKEIPLPHEFILNRDLLAQLYPSFAEGATPFFTLNWSKYAEFLTFR

GGLDPVTGGLWLTDIAHHHLAIAILFLIAGHMYRTNWGIGHGLKDILEAHKGPFTGQG

HKGLYEILTTSWHAQLSLNLAMLGSLTIVVAHHMYSMPPYPYLATDYGTQLSLFTHHM

WIGGFLIVGAAAHAAIFMVRDYDPTTRYNDLLDRVLRHRDAIISHLNWACIFLGFHSF

GLYIHNDTMSALGRPQDMFSDTAIQLQPVFAQWIQNTHALAPGATAPGATASTSLTWG

GGDLVAVGGKVALLPIPLGTADFLVHHIHAFTIHVTVLILLKGVLFARSSRLIPDKAN

LGFRFPCDGPGRGGTCQVSAWDHVFLGLFWMYNSISVVIFHFSWKMQSDVWGSISDQG

VVTHITGGNFAQSSITINGWLRDFLWAQASQVIQSYGSSLSAYGLFFLGAHFVWAFSL

MFLFSGRGYWQELIESIVWAHNKLKVAPATQPRALSIVQGRAVGVTHYLLGGIATTWA

FFLARIIAVG"

gene 44223..46427

/gene="psaB"

CDS 44223..46427

/gene="psaB"

/codon_start=1

/transl_table=11

/product="photosystem I P700 apoprotein A2"

/translation="MALRFPRFSQGLAQDPTTRRIWFGIATAHDFESHDDITEERLYQ

NIFASHFGQLAIIFLWTSGNLFHVAWQGNFESWVQDPLHVRPIAHAIWDPHFGQPAVE

AFTRGGALGPVNIAYSGVYQWWYTIGLRTNEDLYTGALFLLFLSAISLIAGWLHLQPK

WKPSVSWFKNAESRLNHHLSGLFGVSSLAWTGHLVHVAIPGSRGEYVRWNNFLDVLPH

PQGLGPLFTGQWNLYAQNPDSSNHLFGTSQGAGTAILTLLGGFHPQTQSLWLTDMAHH

HLAIAFIFLVAGHMYRTNFGIGHSIKDLLDAHVPPGGRLGRGHKGLYDTINNSLHFQL

GLALASLGVITSLVAQHMYSLPAYAFIAQDFTTQAALYTHHQYIAGFIMTGAFAHGAI

FFIRDYNPEQNEDNVLARMLDHKEAIISHLSWASLFLGFHTLGLYVHNDVMLAFGTPE

KQILIEPIFAQWIQSAHGKTSYGFDVLLSSTSGPAFTAGRSIWLPGWLNAVNENTNSL

FLTIGPGDFLVHHAIALGLHTTTLILVKGALDARGSKLMPDKKDFGYSFPCDGPGRGG

TCDISAWDAFYLAVFWMLNTIGWVTFYWHWKHITLWQGNVSQFNESSTYLMGWLRDYL

WLNSSQLINGYNPFGMNSLSVWAWMFLFGHLVWATGFMFLISWRGYWQELIETLAWAH

ERTPLANLIRWRDKPVALSIVQARLVGLAHFSVGYIFTYAAFLIASTSGKFG"

gene 46555..46857

/gene="rps14"

CDS 46555..46857

/gene="rps14"

/codon_start=1

/transl_table=11

/product="ribosomal protein S14"

/translation="MARKSLIQREKKRQKLEQKYHLIRRSSKKEISKVPSLSDKWQIY

GKLQSPPRNSAPTRLHRRCFSTGRPRANYRDFGLSGHILREMVHACLLPGATRSSW"

gene 47007..47080

/gene="trnfM-CAU"

tRNA 47007..47080

/gene="trnfM-CAU"

/product="tRNA-Met"

gene complement(47234..47304)

/gene="trnG-GCC"

tRNA complement(47234..47304)

/gene="trnG-GCC"

/product="tRNA-Gly"

gene complement(47534..47722)

/gene="psbZ"

CDS complement(47534..47722)

/gene="psbZ"

/codon_start=1

/transl_table=11

/product="photosystem II subunit Z"

/translation="MTLVFQLAVFALIATSSILLISVPVVFASPDGWSSNKNVVFSGT

SLWIGLVFLVGILNSLIS"

gene 48050..48142

/gene="trnS-UGA"

tRNA 48050..48142

/gene="trnS-UGA"

/product="tRNA-Ser"

gene complement(48374..49795)

/gene="psbC"

CDS complement(48374..49795)

/gene="psbC"

/codon_start=1

/transl_table=11

/product="photosystem II 43 kDa protein"

/translation="MKTLYSLRRFYHVETLFNGTLSVAGRDQETTGFAWWAGNARLIN

LSGKLLGAHVAHAGLIVFWAGAMNLFEVAHFVPEKPMYEQGLILLPHLATLGWGVGPG

GEVIDTFPYFVSGVLHLISSAVLGFGGIYHALLGPETLEESFPFFGYVWKDRNKMTTI

LGIHLILLGLGAFLLVFKALYFGGVYDTWAPGGGDVRKITNLTLSPSIIFGYLLKSPF

GGEGWIVSVDDLEDIIGGHVWLGSICILGGIWHILTKPFAWARRALVWSGEAYLSYSL

GALAIFGFTACCFVWFNNTAYPSEFYGPTGPEASQAQAFTFLVRDQRLGANVGSAQGP

TGLGKYLMRSPTGEVIFGGETMRFWDLRAPWLEPLRGPNGLDLSRLKKDIQPWQERRS

AEYMTHAPLGSLNSVGGVATEINAVNYVSPRSWLATSHFVLGFFFFVGHLWHAGRARA

AAAGFEKGIDRDFEPVLSMTPLN"

gene complement(48374..49759)

/gene="psbC"

CDS complement(48374..49759)

/gene="psbC"

/codon_start=1

/transl_table=11

/product="photosystem II 43 kDa protein"

/translation="METLFNGTLSVAGRDQETTGFAWWAGNARLINLSGKLLGAHVAH

AGLIVFWAGAMNLFEVAHFVPEKPMYEQGLILLPHLATLGWGVGPGGEVIDTFPYFVS

GVLHLISSAVLGFGGIYHALLGPETLEESFPFFGYVWKDRNKMTTILGIHLILLGLGA

FLLVFKALYFGGVYDTWAPGGGDVRKITNLTLSPSIIFGYLLKSPFGGEGWIVSVDDL

EDIIGGHVWLGSICILGGIWHILTKPFAWARRALVWSGEAYLSYSLGALAIFGFTACC

FVWFNNTAYPSEFYGPTGPEASQAQAFTFLVRDQRLGANVGSAQGPTGLGKYLMRSPT

GEVIFGGETMRFWDLRAPWLEPLRGPNGLDLSRLKKDIQPWQERRSAEYMTHAPLGSL

NSVGGVATEINAVNYVSPRSWLATSHFVLGFFFFVGHLWHAGRARAAAAGFEKGIDRD

FEPVLSMTPLN"

gene complement(49743..50804)

/gene="psbD"

CDS complement(49743..50804)

/gene="psbD"

/codon_start=1

/transl_table=11

/product="photosystem II protein D2"

/translation="MTIALGKFTKDEKDLFDIMDDWLRRDRFVFVGWSGLLLFPCAYF

ALGGWFTGTTFVTSWYTHGLASSYLEGCNFLTAAVSTPANSLAHSLLLLWGPEAQGDF

TRWCQLGGLWTFVALHGAFALIGFMLRQFELARSVQLRPYNAIAFSGPIAVFVSVFLI

YPLGQSGWFFAPSFGVAAIFRFILFFQGFHNWTLNPFHMMGVAGVLGAALLCAIHGAT

VENTLFEDGDGANTFRAFNPTQAEETYSMVTANRFWSQIFGVAFSNKRWLHFFMLFVP

VTGLWMSALGVVGLALNLRAYDFVSQEIRAAEDPEFETFYTKNILLNEGIRAWMAAQD

QPHENLIFPEEVLPRGNAL"

gene 52052..52123

/gene="trnT-GGU"

tRNA 52052..52123

/gene="trnT-GGU"

/product="tRNA-Thr"

gene 52749..52818

/gene="trnE-UUC"

tRNA 52749..52818

/gene="trnE-UUC"

/product="tRNA-Glu"

gene 52878..52961

/gene="trnY-GUA"

tRNA 52878..52961

/gene="trnY-GUA"

/product="tRNA-Tyr"

gene 53074..53147

/gene="trnD-GUC"

tRNA 53074..53147

/gene="trnD-GUC"

/product="tRNA-Asp"

gene 53686..53790

/gene="psbM"

CDS 53686..53790

/gene="psbM"

/codon_start=1

/transl_table=11

/product="photosystem II subunit M"

/translation="MEVNILAFIATTLFILVPTAFLLIIYVKTVSQND"

gene complement(54583..54672)

/gene="petN"

CDS complement(54583..54672)

/gene="petN"

/codon_start=1

/transl_table=11

/product="cytochrome b6/f subunit N"

/translation="MDIVSLAWAALMVVFTFSLSLVVWGRSGL"

gene complement(55499..55574)

/gene="trnC-GCA"

tRNA complement(55499..55574)

/gene="trnC-GCA"

/product="tRNA-Cys"

gene 56683..59895

/gene="rpoB"

CDS 56683..59895

/gene="rpoB"

/codon_start=1

/transl_table=11

/product="RNA polymerase subunit beta"

/translation="MLGDANEAMSTIPGFHQIQFEGFCRFINRGLTEELYKFPKIEDT

DHEIEFQLFLERYQLVEPLIKERNAVYESLTYSSELYVSAGLIWKTSRDMQEQTILVG

NIPLMNSLGTSIVNGIYRIVINQILQSPGIYYRSELDHNGISVYTGTIISDWGGRSEL

EIDRKARIWARVSRKQKISILVLSSAMGSNLREILDNVYYPEIFLSFLNDKERKKIGS

KENAILEFYQQFACVGGDPVFSESLCKELQKKFFQQRCELGRIGRRNMNRRLNLDIPP

NNTFLLPRDILAAADHLIGLKFGMGTLDDMNHLKNKRIRSVADLLQDQFGLSLVRLEN

VVRGTICGAIRHKLIPTPQNLVTSTPLTTTYESFFGLHPLSQVLDRTNPLTQIVHGRK

LSYLGPGGLTGRTASFRIRDIHPSHYGRICPIDTSEGINVGLIGSLAIHARMGYWGSL

ESPFYKISERSTGLRLLYLSPGRDEYYMLAAGNSLALNQDIQEEQVVPARYRQEFLTI

AWERVHLRSIFPFQYFSIGASLIPFIEHNDANRALMSSNMQRQAVPLSRSEKCIVGTG

LERQAALDSGALAIAERGGKIIYIDTDKILFSGNGDTLSISLVMYQRSNKNTCMHQKT

RVQRGKCIKKGQILADGAATVGGELALGKNVLVAYMPWEGYNSEDAVLISERLVYEDI

YTSFHIRKYEIQTHATSQGPERITNEIPHLEARLLRNLDKNGIVMLGSWVETGDILVG

KLTPQMVKESSYAPEDRLLRAILGIQVSTSKETCLKLPIGGKGRVIDVRWIQKRGGSS

YNPETIRVYISQKREIKVGDKVAGRHGNKGIISKILPRQDMPYLQDGRPVDMVFNPLG

VPSRMNVGQIFECSLGLAGGLLDRHYRIAPFDERYEQEASRKLVFSELYEASKQTANP

WVFEPEYPGKSRIFDGRAGSPFEQPVIIGKPYILKLIHQVDDKIHGRSSGHYALVTQQ

PLRGRAKQGGQRVGEMEVWALEGFGVAHILQEMLTYKSDHIRARQEVLGTTIIGGIIP

NPEDAPESFRLLVRELRSLALELNHFLVSEKNFQINRKEA"

gene 59922..62759

/gene="rpoC1"

CDS join(59922..60353,61149..62759)

/gene="rpoC1"

/codon_start=1

/transl_table=11

/product="RNA polymerase subunit beta'"

/translation="MIDRYKHQQLRIGLVSPQQISAWATKILPNGEIVGEVTKPYTFH

YKTNKPEKGGLFCERIFGPIKSGICACGNYRVIGDEKEDPKFCEQCGVEFVDSRIRRY

QMGYIKLACPVTHVWYLKRLPSYIANLLDKPLKELEGLVYCDFSFARPITKKPTFLRL

RGLFEYEIQSWKYSIPLFFTTQGFDTFRNREISTGASAIREQLADLDLRIILHNSLVE

WKELGEEGPTGNEWEDRKVGRRKDFLVRRMELAKHFLRTNIEPEWMVLCLLPVLPPEL

RPIIQIDGGKLMSSDINELYRRVIYRNNTLTDLLTTSRSTPGELVMCQEKLVQEAVDT

LLDNGIRGQPTRDGHNKAYKSFSDVIEGKEGRFRETLLGKRVDYSGRSVIVVGPSLSL

HRCGLPREIAIELFQTFVIRGLIRQHLASNIGVAKSKIREKEPIVWEILQEVMQGHPV

LLNRAPTLHKLGIQAFQPILVEGRAICLHPLVCKGFNADFDGDQMAVHVPLSLEAQAE

ARLLMFSHMNLLSPAIGDPISVPTQDMLIGLYVLTSGNRRGICINRYNPWNRKNYQNK

RSDNNKYKYTKEPFFSNSYDAIGAYRQKRINLDSPLWLRWRLDQRVIASRETPIEVHY

ESLGNYYEIYGHYLIVRSIKKEILFLYIRTTVGHISLYREIEEAIQGFSRACSYGT"

exon 59922..60353

/gene="rpoC1"

/number=1

intron 60354..61148

/gene="rpoC1"

/number=1

exon 61149..62759

/gene="rpoC1"

/number=2

gene 62914..67104

/gene="rpoC2"

CDS 62914..67104

/gene="rpoC2"

/codon_start=1

/transl_table=11

/product="RNA polymerase subunit beta''"

/translation="MEVLMAERANLVFHNKVIDGTAMKRLISRLIDHFGMAYTSHILD

QVKTLGFQQATATSISLGIDDLLTIPSKRWLVQDAEQQSFILEKHHHYGNVHAVEKLR

QSIEIWYATSEYLRQEMNPNFRMTDPLNPVHIMSFSGARGNASQVHQLVGMRGLMSDP

QGQMIDLPIQSNLREGLSLTEYIISCYGARKGVVDTAVRTSDAGYLTRRLVEVVQHIV

VRRIDCGTARGISVSPQNGMMPERIFIQTLIGRVLADDIYMGTRCIATRNQDIGIGLV

NRFITFRAQPIAIRTPFTCRSASWICRLCYGRSPTHGDLVELGEAVGIIAGQSIGEPG

TQLTLRTFHTGGVFTGGTAEHVRAPSNGKIKFNEDLVHPTRTRHGHPALLCSINLYVT

IESEDIRHNVNIPSQSFLLVQNDQYVESEQVIAEIRAGTSTLNFKEKIRKHIYSDSDG

EMHWSTDVYHAPEFTYGSVHLLPKTSHLWILLGEPRGSSLVSLSIYKDQDQMSANSRS

VKSFNLSGTDDQLREKFFTSDFSGKKEDRIPDYSDLSRIICTGRCNLIDPTLLYQNSD

LFSKRRRNRFIIPLQSIQERENGLTPPSDILIEIPINGIFRRNSILAYLDDPRYRRKS

SGITKYGTLEMHSIVKKEDLIEYRGGKEFSPKYQMKVDRFFFIPEEVHILPGSSSIMV

RNNSIIGVDTQITLNMRSRVAGLVRVERKKKRIELKIFSGDIHFPGETDKISRHSGVL

IPLRTGKRNSKESKKRENGIYVQRITPSKKKYFVLVRPVVTYEITDGITLGTLFPPDL

LQERDNVKLRVVNYILYGNGKPIRGISDTNIQLVRTCLVLNWDQDKKSSSSQEARASF

VEIRVNGLIRHLLRIDLLKSTFSYIGKRNDPSGSGLFSDNGSDCTKRNPFSSIYSKAI

IQQSLNQNKRTIHTLLNRNAGFQSLIILSSSNCFRMGPFNDVKYHNHNVIHNGIKESI

NITKDPVIPIQNSLGPLGTVPLIRIANVYSFYHLITHNQILVKNYLQLDNLKQTFQVI

KLKYYLIDEKEKIYNPDPCSNIIFNLNWYFLHPNYCQETSTIMSLGQFICENIYISKS

APHLKSGQVILVQVDSVVIRSAKTYLATPGATVHGHYGEILYEGDTLITFIYEKSRSG

DITQGLPKVEQVLEVRSIDSISMNLDKRIEGWNERITRILGMPWAFLIGAELTIVQSR

ISLVNKIQKVYRSQGVQIHNRHIEIIVRQITSRVLVSEDGMSNVFSPGELIGLVRAER

MGRALEEAVCYRALLLGITRASLNTQSFISEASFQETARVLAKAALRGRIDWLKGLKE

NVVLGGMIPVGTGLKGFVPPSKQHNRSPLEMKKKKKNLFEGEMRDILFHHRKLFDSFL

SNNLDDRPEQSFIGFNDS"

gene 62926..67104

/gene="rpoC2"

CDS 62926..67104

/gene="rpoC2"

/codon_start=1

/transl_table=11

/product="RNA polymerase subunit beta''"

/translation="MAERANLVFHNKVIDGTAMKRLISRLIDHFGMAYTSHILDQVKT

LGFQQATATSISLGIDDLLTIPSKRWLVQDAEQQSFILEKHHHYGNVHAVEKLRQSIE

IWYATSEYLRQEMNPNFRMTDPLNPVHIMSFSGARGNASQVHQLVGMRGLMSDPQGQM

IDLPIQSNLREGLSLTEYIISCYGARKGVVDTAVRTSDAGYLTRRLVEVVQHIVVRRI

DCGTARGISVSPQNGMMPERIFIQTLIGRVLADDIYMGTRCIATRNQDIGIGLVNRFI

TFRAQPIAIRTPFTCRSASWICRLCYGRSPTHGDLVELGEAVGIIAGQSIGEPGTQLT

LRTFHTGGVFTGGTAEHVRAPSNGKIKFNEDLVHPTRTRHGHPALLCSINLYVTIESE

DIRHNVNIPSQSFLLVQNDQYVESEQVIAEIRAGTSTLNFKEKIRKHIYSDSDGEMHW

STDVYHAPEFTYGSVHLLPKTSHLWILLGEPRGSSLVSLSIYKDQDQMSANSRSVKSF

NLSGTDDQLREKFFTSDFSGKKEDRIPDYSDLSRIICTGRCNLIDPTLLYQNSDLFSK

RRRNRFIIPLQSIQERENGLTPPSDILIEIPINGIFRRNSILAYLDDPRYRRKSSGIT

KYGTLEMHSIVKKEDLIEYRGGKEFSPKYQMKVDRFFFIPEEVHILPGSSSIMVRNNS

IIGVDTQITLNMRSRVAGLVRVERKKKRIELKIFSGDIHFPGETDKISRHSGVLIPLR

TGKRNSKESKKRENGIYVQRITPSKKKYFVLVRPVVTYEITDGITLGTLFPPDLLQER

DNVKLRVVNYILYGNGKPIRGISDTNIQLVRTCLVLNWDQDKKSSSSQEARASFVEIR

VNGLIRHLLRIDLLKSTFSYIGKRNDPSGSGLFSDNGSDCTKRNPFSSIYSKAIIQQS

LNQNKRTIHTLLNRNAGFQSLIILSSSNCFRMGPFNDVKYHNHNVIHNGIKESINITK

DPVIPIQNSLGPLGTVPLIRIANVYSFYHLITHNQILVKNYLQLDNLKQTFQVIKLKY

YLIDEKEKIYNPDPCSNIIFNLNWYFLHPNYCQETSTIMSLGQFICENIYISKSAPHL

KSGQVILVQVDSVVIRSAKTYLATPGATVHGHYGEILYEGDTLITFIYEKSRSGDITQ

GLPKVEQVLEVRSIDSISMNLDKRIEGWNERITRILGMPWAFLIGAELTIVQSRISLV

NKIQKVYRSQGVQIHNRHIEIIVRQITSRVLVSEDGMSNVFSPGELIGLVRAERMGRA

LEEAVCYRALLLGITRASLNTQSFISEASFQETARVLAKAALRGRIDWLKGLKENVVL

GGMIPVGTGLKGFVPPSKQHNRSPLEMKKKKKNLFEGEMRDILFHHRKLFDSFLSNNL

DDRPEQSFIGFNDS"

gene 67324..68034

/gene="rps2"

CDS 67324..68034

/gene="rps2"

/codon_start=1

/transl_table=11

/product="ribosomal protein S2"

/translation="MTRRYWNINLDEMLEAGVHFGHGTRKWNPKMAPYISAKRKGIHI

TNLTKTARFLSEACDLVFDAASRGKQFLIVGTKKKAADSVARAAIKARCHCVNKKWLG

GMLTNWSTTETRLHKFRDLRMEQKTGRLNGLPKRDAAVVKRQLYRLQTYLGGIKYMTG

LPDIVIIVDQHEEYTALRECITLGIPTICLIDTNCDPDLADISIPANDDAISSIRLIL

NKLVFAISEGHSSYIRNP"

gene 68265..69008

/gene="atpI"

CDS 68265..69008

/gene="atpI"

/codon_start=1

/transl_table=11

/product="CF0 subunit IV"

/translation="MNVLSCSINTLKGLYDISGVEVGQHFYWQIGGFQVHGQVLITSW

VVIAILLGSATIAVRNPQTIPTGGQNFFEYVLEFIRDVSKTQIGEEYGPWVPFIGTMF

LFIFVSNWSGALLPWKIIELPHGELAAPTNDINTTVALALLTSAAYFYAGLTKKGLSY

FGKYIQPTPILLPINILEDFTKPLSLSFRLFGNILADELVVVVLVSLVPSVIPIPVMF

LGLFTSGIQALIFATLAAAYIGESMEGHH"

gene 69989..70234

/gene="atpH"

CDS 69989..70234

/gene="atpH"

/codon_start=1

/transl_table=11

/product="CF0 subunit III"

/translation="MNPLISAASVIAAGLAVGLASIGPGVGQGTAAGQAVEGIARQPE

AEGKIRGTLLLSLAFMEALTIYGLVVALALLFANPFV"

gene 70604..71815

/gene="atpF"

CDS join(70604..70748,71406..71815)

/gene="atpF"

/codon_start=1

/transl_table=11

/product="CF0 subunit I"

/translation="MKNVTDSFVSLGHWPSAGSFGFNTDILATNPINLSVVIGVLIFF

GKGVLSDLLDNRKQRILNTIRNSEELRGGAIEQMEKARARLRKVEMEADQFRVNGYSE

IEQEKLNLINSTYNTLEQLENYKNETIQFEQQRAINQVRQRVFQQALQGAIGTLNSCL

NNELHLRTISANIGMLGAMKEITD"

CDS join(70604..70747,71405..71815)

/gene="atpF"

/codon_start=1

/transl_table=11

/product="CF0 subunit I"

/translation="MKNVTDSFVSLGHWPSAGSFGFNTDILATNPINLSVVIGVLIFF

GKGVLSDLLDNRKQRILNTIRNSEELRGGAIEQMEKARARLRKVEMEADQFRVNGYSE

IEQEKLNLINSTYNTLEQLENYKNETIQFEQQRAINQVRQRVFQQALQGAIGTLNSCL

NNELHLRTISANIGMLGAMKEITD"

gene 70604..70768

/gene="atpF"

CDS 70604..70768

/gene="atpF"

/codon_start=1

/transl_table=11

/product="CF0 subunit I"

/translation="MKNVTDSFVSLGHWPSAGSFGFNTDILATNPINLSVVIGVLIFF

GKGVCVGCLFQ"

exon 70604..70748

/gene="atpF"

/number=1

exon 70604..70747

/gene="atpF"

/number=1

intron 70748..71404

/gene="atpF"

/number=1

intron 70749..71405

/gene="atpF"

/number=1

exon 71405..71815

/gene="atpF"

/number=2

exon 71406..71815

/gene="atpF"

/number=2

gene 71904..73427

/gene="atpA"

CDS 71904..73427

/gene="atpA"

/codon_start=1

/transl_table=11

/product="CF1 subunit alpha"

/translation="MVTIQADEISNILRERIEQYNREVKIVNTGTVLQVGDGIARIYG

LDEVMAGELVEFEEGTIGIALNLESNNVGVVLMGDGLMIQEGSSVKATGRIAQIPVSE

AYLGRVINALAKPIDGRGEISASESRLIESPAPGIISRRSVYEPLQTGLIAIDSMIPI

GRGQRELIIGDRQTGKTAVATDTILNQQGQNVICVYVAIGQKASSVAQVVTTLQERGA

MEYTIVVAEMADSPATLQYLAPYTGAALAEFFMYRKQHTLIIYDDPSKQAQAYRQMSL

LLRRPPGREAYPGDVFYLHSRLLERAAKSSSSLGEGSMTALPIVETQSGDVSAYIPTN

VISITDGQIFLSADLFNAGIRPAINVGISVSRVGSAAQIKAMKQVAGKLKLELAQFAE

LEAFAQFASDLDKATQNQLARGQRLRELLKQSQAAPLAVEEQIMTIYTGTNGYLDSLE

IGQVRKFIVELRNYLKTNKPQFQEIISSTKIFTEEAEALLKEAIQEQMDRFLLQEQA"

gene complement(73524..73598)

/gene="trnR-UCU"

tRNA complement(73524..73598)

/gene="trnR-UCU"

/product="tRNA-Arg"

gene complement(73773..74530)

/gene="trnG-UCC"

tRNA complement(join(73773..73819,74508..74530))

/gene="trnG-UCC"

/product="tRNA-Gly"

exon complement(73773..73819)

/gene="trnG-UCC"

/number=2

intron complement(73820..74507)

/gene="trnG-UCC"

/number=1

exon complement(74508..74530)

/gene="trnG-UCC"

/number=1

gene 75089..75176

/gene="trnS-GCU"

tRNA 75089..75176

/gene="trnS-GCU"

/product="tRNA-Ser"

gene complement(75303..75413)

/gene="psbI"

CDS complement(75303..75413)

/gene="psbI"

/codon_start=1

/transl_table=11

/product="photosystem II subunit I"

/translation="MLTLKLFVYTVVIFFVSLFIFGFLSNDPGRNPGREE"

gene complement(75781..75966)

/gene="psbK"

CDS complement(75781..75966)

/gene="psbK"

/codon_start=1

/transl_table=11

/product="photosystem II subunit K"

/translation="MLNIVSLICICINSPFYSSSFFFGKLPEAYAFLNPIVDVMPVIP

LFFFLLAFVWQAAVSFR"

gene 76303..76374

/gene="trnQ-UUG"

tRNA 76303..76374

/gene="trnQ-UUG"

/product="tRNA-Gln"

gene 77374..78513

/gene="rps16"

CDS join(77374..77409,78283..78513)

/gene="rps16"

/codon_start=1

/transl_table=11

exon 77374..77409

/gene="rps16"

/number=1

intron 77410..78282

/gene="rps16"

/number=1

exon 78283..78513

/gene="rps16"

/number=2

gene 79448..82038

/gene="trnK-UUU"

tRNA join(79448..79484,82004..82038)

/gene="trnK-UUU"

/product="tRNA-Lys"

exon 79448..79484

/gene="trnK-UUU"

/number=1

intron 79485..82003

/gene="trnK-UUU"

/number=1

gene 80198..81733

/gene="matK"

CDS 80198..81733

/gene="matK"

/codon_start=1

/transl_table=11

/product="maturase K"

/translation="MEEIQRYLQLERSQQHDFLYPLIFQEYIYAFAHNRALSKLILSE

NLGYDNKSSLLIVKRLITRLYQQNHFLISPNDSNQNPFWVRNKNLYSQIISEGFAFIV

EIPFSLQFLSCLEGETNKIGKSQNLRSIHSIFPFFEDTFSHFNFVLDMVIPRPVHVEI

LVQILRYCVKDASSLHLLRVFLNEYCNWNSLLIPKKASSPSSKKNKRLFLFLYNSHVC

EYESIFVFLRNQSFHLRSTSSGVFLERIYFYIKIERLVNVFVKIKDLGANLRLVKEPF

MHYIRYQKRSILASKGTFIFMKKWKFYLVTFWQWHFSVWFHPKSIYINQLSKHSLEFL

GYLSSVRMNPSVVRSQILENSFLINNAINKFETLVPIIPLIASLAKAKFCNVLGHPVS

KPIRADLSDSNIIDRFGRICRNISRYHSGSSKKKSLYRIKYILRLSCARTLARKHKST

VRAFLKRLGSEFLEEFLMSEEDVLFLTFQKTSYTLRGVYRSRIWYLDMISINDLANHK

SKF"

exon 82004..82038

/gene="trnK-UUU"

/number=2

gene 82271..83329

/gene="psbA"

CDS 82271..83329

/gene="psbA"

/codon_start=1

/transl_table=11

/product="photosystem II protein D1"

/translation="MTAILERRESESLWGRFCNWITSTENRLYIGWFGVLMIPTLLTA

TSVFIIAFIAAPPVDIDGIREPVSGSLLYGNNIISGAIIPTSAAIGLHFYPIWEAASV

DEWLYNGGPYELIVLHFLLGVACYMGREWELSFRLGMRPWIAVAYSAPVAAATAVFLI

YPIGQGSFSDGMPLGISGTFNFMIVFQAEHNILMHPFHMLGVAGVFGGSLFSAMHGSL

VTSSLIRETTENESANEGYRFGQEEETYNIVAAHGYFGRLIFQYASFNNSRSLHFFLA

AWPVVGIWFTALGISTMAFNLNGFNFNQSVVDSQGRVINTWADIINRANLGMEVMHER

NAHNFPLDLASIEAPTNG"

gene 83559..83633

/gene="trnH-GUG"

tRNA 83559..83633

/gene="trnH-GUG"

/product="tRNA-His"

CDS complement(83590..83681)

/gene="rps19"

/codon_start=1

/transl_table=11

gene complement(83634..83681)

/gene="rps19-fragment"

CDS complement(83634..83681)

/gene="rps19-fragment"

/codon_start=1

/transl_table=11

repeat_region 83640..109277

/gene="rps12"

/note="inverted repeat A (IRA)"

/rpt_type=inverted

gene complement(83734..85225)

/gene="rpl2"

CDS complement(join(83734..84167,84835..85225))

/gene="rpl2"

/codon_start=1

/transl_table=11

/product="ribosomal protein L2"

/translation="MAIHLYKTSTPSTRNGTVDSQVKSNPRNNLIYGQHHCGKGRNAR

GIITSRHRGGGHKRLYRKIDFRRNEKDIYGRIVTIEYDPNRNAYICLIHYGDGEKRYI

LHPRGAIIGDTIVSGTEVPIKMGNALPLTDMPLGTAIHNIEITLGKGGQLVRAAGAVA

KLIAKEGKSATLKLPSGEVRLISKNCSATVGQVGNVGANQKSLGRAGSKRWLGKRPVV

RGVVMNPVDHPHGGGEGRAPIGRKKPTTPWGYPALGRRSRKRNKYSENLIVRRRSK"

exon complement(83734..84167)

/gene="rpl2"

/number=2

intron complement(84168..84834)

/gene="rpl2"

/number=1

exon complement(84835..85225)

/gene="rpl2"

/number=1

gene complement(85244..85525)

/gene="rpl23"

CDS complement(85244..85525)

/gene="rpl23"

/codon_start=1

/transl_table=11

/product="ribosomal protein L23"

/translation="MDGIKHAVFTDKSIRLLGKNQYTSNVESGSTRTELKHWVELFFG

VRVIAMNSHRLPGKGRRMGPIMGHTMHYRRMIITLQPGYSIPPLRKKRT"

gene complement(85691..85764)

/gene="trnI-CAU"

tRNA complement(85691..85764)

/gene="trnI-CAU"

/product="tRNA-Ile"

gene 85853..92698

/gene="ycf2"

CDS 85853..92698

/gene="ycf2"

/codon_start=1

/transl_table=11

/product="Ycf2 protein"

/translation="MKGHQFQSWIFELREIKNSHCFLDSWTQFNSVGSFIRIFFHQER

FLKLFDPRILSILLSRNSQGSTSNRYFTIKGVLLFVVAVLIYRINNRNMVERKNLYLR

GLLPIPMNSIGPINDTLEESVGSSNINRLIVSLLYLPKGKKISESCFLNPKESTWVLP

ITKKCSMPESNWGSRWWRNWIGKKRDSSCKISNETVTGIGILFKEKDLKYLEFLFVYY

MDDPIRKDHDWELFDRLSLRKRRNRINLNSGPLFEILVKHWISYLMSAFREKIPIEVE

GFFKQQRAGSTIQSNDIEHVSHLLSRNKRAISLQNCAQFHMWQFRQDLFVSWGKNPHE

SDFLRNVSRENWIWLDNVWLVNKDRFFRKVRNVSSNIQYDSTRSSFVQVTDSSQLKGS

SDQSRDHLDSISNEDSEYHTLINQREIQPLKERSILWDPSFLQTEGTEIESDRFPKCL

SGYSSMSRLFTEREKQMINHLLPEEIQEFLGNPTRSVRSFFSDRWSELHLGSNPTERS

TRDPKLLKKQQDLSFVPPRRSENKELVNIFKIITYLQNTVSIHPISSDPGCDRVLKDE

PDMDSSNKISFLNKNPFFDLFHLFHDRNRGGYTLHHDFESEERFQEMADLFTLSITEP

DLVYHKGFPFSIDSYGLDQKQFLNEARDESKKKSLLVLPPIFYEENESFSRRIRKKWV

RISCGNDLEDPKPKIVVFASNNIMEAVNQYRLIRNLIQIQYSTYGYIRNVWNRFFLMN

RSDRNFEYGIQRDQIGKDTLNHRTIMKYTINAHLSNLKKSQKKWFDPLILISRTERSM

NRAPDAYRYKWSNGSKNFQEHLEHFVSEQKSRFQIVFDRLRINQYSIDWSEVIDKKDL

SKPLRFFLSKSLLFLSKLLFFLSNSLPFFCVSFGNIPIHRSEIYIYELKGPNDQLCNQ

LLESIGLQIVHLKKWKPFLLDEHDTSRKSKFLINGGTPFLFNKIPKWMIDSFHTRNNR

RKSFDNADSYFSMIFHNQDNWLNPVKPFHISSLISSFYKANRLRFLNNPHHFCFYCNT

RFPFSVEKARINNYDFTYGQFLNILFIRNKIFSLCVGKKKHAFGGRDTISPIESQVSN

IFIPNDFPQSGDETYNLYKSFHFPSQHDPFVRRTIYSIADMFGTPLTEGQIVHFERTY

CQPLSDMNLSDSEGKNLHQYLNSNVGLIHTPCSEKYLPSEKRKKRSLCLKKCVEKGQM

YRTFQRDGAFSTLSKWNLFQTYIPWFLTSTGYKYLNLIFLDTFSDLLPILSSSQKFVS

IFHDIMHGSGIAWRILQKKWCLPQWNLISAISSKCFHNLLLSEEMIHRNNESPSTHLR

SPNVREFLYSILFLLLVAGYLVRTHLLFVSRASSELQTEFEKVKSLMIPSSMIELRKL

LDRYPTSAPNSFWLKNLFLVALEQLGDSLEEIRASGGNMPGPAYGVKSIRSKKKYLSI

NLIDLIPNPINRITFSRNTRHLSHTSKEIYSLIRKRKNVNGDWIDDKIESWVANSDSI

DDEEREFLVQFSALTTEKRIDQILLSLTHSDHLSKNDSGYQMIEQPGAIYLRYLVDIH

KKYLLNYEFNTSSLAERRVFLAHYQTITYSQTSCGTNTLHFPSHGKPFSLRLALSPSR

GILVIGSIGTGRSYLVKYLATNSYVPFITVFLNKFLDNKPKGFLFDDIDIDASDDIDA

SDDIDASDDIDASDDIDRDLDTELELLTMDRIPEIDRFYITLQFELAKAISPCIIWIP

NIHDLDVNESNYLSLGLLVNHLSERCSTRNILVIASTHIPQKVDPALIAPNKLNTCIK

IRRLLIPQQRKHFFTLSYTKGFHLEKKMFHTNGFGSITMGSNARDLVALTNEALSISI

TQKKSIIDTKTIRSALHRQTWDLRSQVRSVQDHGILFYQIGRAVAQNVLLSNCPIDPI

SIYMKKKSCNEGDSYLYKWYFELGTSMKKLTILLYLLSCSAGSVAQDLWSLPGSDEKN

GITSYGLVENDSDLVHGLLEVEGALVGSSRTEKDCSPFDNDRVTLLLRPEPRNPLDMM

QNGSCSIFDQRFLYEKYESEFEEGKGEGALDPQQIEEDLFNHIVWAPRIWRPWAFLFD

CIERPNELGFPYWSRSFRGKRIIYDEEDELQENDSEFLQSGTMQYQTRDRSSKEQGLF

QISQFIWDPADPLFFLFKDQPPGSVFSHRELFADEEMSKGLLTSQMDPPTSIYKRWFI

KNTQEKHFELLINRQRWLRTNSSLSNGSFRSNTLSESYQYLSTLFLSNGTLLDQMTKT

LLRKRWLFPDEMKIGFMEQEKDFPFLSRKDMWL"

gene 92741..92989

/gene="ycf15"

CDS 92741..92989

/gene="ycf15"

/codon_start=1

/transl_table=11

gene complement(93250..93330)

/gene="trnL-CAA"

tRNA complement(93250..93330)

/gene="trnL-CAA"

/product="tRNA-Leu"

gene complement(93880..96091)

/gene="ndhB"

CDS complement(join(93880..94635,95315..96091))

/gene="ndhB"

/codon_start=1

/transl_table=11

/product="NADH dehydrogenase subunit B"

/translation="MIWHVQNENFILDSTRIFMKAFHLLLFDGSFIFPECILIFGLIL

LLMIDSTSDQKDIPWLYFISSTSLVMSITALLFRWREEPIISFSGNFQTNNFNEIFQF

LILLCSTLCIPLSVEYIECTEMAITEFLLFVLTATLGGMFLCGANDLITIFVAPECFS

LCSYLLSGYTKKDVRSNEATMKYLLMGGASSSILVHGFSWLYGLSGGETELQEIVNGL

INTQMYNSPGISIALIFITVGIGFKLSPAPSHQWTPDVYEGSPTPVVAFLSVTSKVAA

SASATRILDIPFYFSSNEWHLLLEILAILSMILGNIIAITQTSMKRMLAYSSIGQIGY

VIIGIIVGDSNDGYASMITYMLFYISMNLGTFACIVLFGLRTGTDNIRDYAGLYTKDP

FLALSLALCLLSLGGLPPLAGFFGKLYLFWCGWQAGLYSLVLIGLLTSVVSIYYYLKI

IKLLMTGRNQEITPHVRNYRRSPLRSNNSVELSMIVCVIASTIPGISMNPIIAIAQDT

LF"

exon complement(93880..94635)

/gene="ndhB"

/number=2

intron complement(94636..95314)

/gene="ndhB"

/number=1

exon complement(95315..96091)

/gene="ndhB"

/number=1

gene complement(96366..96833)

/gene="rps7"

CDS complement(96366..96833)

/gene="rps7"

/codon_start=1

/transl_table=11

/product="ribosomal protein S7"

/translation="MSRRGTAEEKTAKSDPIYRNRLVNMLVNRILKHGKKSLAYQIIY

RAMKKIQQKTETNPLSVLRQAIRGVTPDIAVKARRVGGSTHQVPIEIGSTQGKALAIR

WLLAASRKRPGRNMAFKLSSELVDAAKGSGDAIRKKEETHKMAEANRAFAHFR"

exon complement(96887..96912)

/gene="rps12"

/number=3

intron complement(96913..97448)

/gene="rps12"

/number=1

exon complement(97449..97680)

/gene="rps12"

/number=2

gene 99296..99367

/gene="trnV-GAC"

tRNA 99296..99367

/gene="trnV-GAC"

/product="tRNA-Val"

gene 99595..101085

/gene="rrn16"

rRNA 99595..101085

/gene="rrn16"

/product="16S ribosomal RNA"

gene 101375..102383

/gene="trnI-GAU"

tRNA join(101375..101411,102349..102383)

/gene="trnI-GAU"

/product="tRNA-Ile"

exon 101375..101411

/gene="trnI-GAU"

/number=1

intron 101412..102348

/gene="trnI-GAU"

/number=1

exon 102349..102383

/gene="trnI-GAU"

/number=2

gene 102448..103326

/gene="trnA-UGC"

tRNA join(102448..102485,103292..103326)

/gene="trnA-UGC"

/product="tRNA-Ala"

exon 102448..102485

/gene="trnA-UGC"

/number=1

intron 102486..103291

/gene="trnA-UGC"

/number=1

exon 103292..103326

/gene="trnA-UGC"

/number=2

gene 103518..106328

/gene="rrn23"

rRNA 103518..106328

/gene="rrn23"

/product="23S ribosomal RNA"

gene 103518..103829

/gene="rrn23-fragment"

rRNA 103518..103829

/gene="rrn23-fragment"

gene 104076..106328

/gene="rrn23"

rRNA 104076..106328

/gene="rrn23"

/product="23S ribosomal RNA"

gene 106427..106529

/gene="rrn4.5"

rRNA 106427..106529

/gene="rrn4.5"

/product="4.5S ribosomal RNA"

gene 106785..106905

/gene="rrn5"

rRNA 106785..106905

/gene="rrn5"

/product="5S ribosomal RNA"

gene 107140..107212

/gene="trnR-ACG"

tRNA 107140..107212

/gene="trnR-ACG"

/product="tRNA-Arg"

gene complement(107786..107857)

/gene="trnN-GUU"

tRNA complement(107786..107857)

/gene="trnN-GUU"

/product="tRNA-Asn"

misc_feature 109278..126794

/gene="rps12"

/note="small single copy (SSC)"

gene 109351..113817

/gene="ycf1"

CDS 109351..113817

/gene="ycf1"

/codon_start=1

/transl_table=11

/product="Ycf1 protein"

/translation="MNINENQDNSRLKIFDQKTENKELIIFDKPLVTILFDSKRWNRP

FRYIKNKRFDKAIRNEMSQYFFDICQSDGKERISFTYPPSLSIFLEMIKKRISPPTIE

KFSFNELYNPWVYTNNQKEKNFNNEFLNRIKALDKENSYLNILETQTRLCNDYSTKEY

LSKRYDPFLNGSYRKTIYKSPSPSTLKKTLIENFLDPFGINRIHGILLPATDYQEFDQ

KINRFEIKSLSTEIVNFLTFISKFVKESGSTNLNPSSLYLFYLFSEGKIDSQKERKYF

NYLLNLNKIVTDANGQKINRKSIRIKEINKKVPRWSYKLITDLEQQSRKYKEDLPIGH

QIRSRRGKRVVILTATKGTPKTTNSKMSDIKTDVTLMRYSQQSDFRRGIIKGSMRAQR

RKVVIFKLFQANAKSPLFLERRHKAPPFYFNISGLIKLIFKNGLDKGEAFKIVEYTKE

QTKKQENNKRKENARIKVAEDWNRIPFAQAIRGCVLLTQSIFRKYILFPSLIIAKNVG

RIFLLQRPEWSEDFQEWNKEIYIKCTSNGIPLSETEFPKNWLTEGIQIKIVFPFCLKP

SHKSKLRSSQKDLMKKTKGDYCFLTVWGMETELPFSSPRKKPSLIKPILKEFPKKIGK

LKKKYFRVLTVFKVKTKLLRKVLKETKKWVIKSVFFRKRIIKELSKVNPILLFRLREV

GVDKSSEIKEEKDSIINNQTIHESFTQIQIASPSWTNSSLTEKKMKDLTDRTSTIRNQ

IERITKEKKKVTPRINNLSPTSYNAKKLEKQQMLKMLKRRNARLICKLSPFVKFFIEK

IYTDIFLYIINIARINTKLFLKLTKKIIDKSIYNNERKQERINKKKKTKILSISSIIL

RKELENISNIKANSHIFYDLSYVPQPYVFYKLGKIQVINSLRFVVQYQRIPFLLKAKI

KDSFETQGMLDSKSADNKITSYEMNPWKSWLRGHYQYHLSQIGWSRLIPEKWRNTFRQ

QRIAKKANFSKRHSYEKNPLMNSKKQKKFEVYSLSNQKDNFIKYYRSDLLSYKFIHYE

KKTECFFYGSPLQGNTKQEIYYNTPKKNFVAMLRNIPIKNDLGKIHMEKPADRKYFDW

KIFQFDLIQKVDIEAWIIIDTNRNQNTQVRTKNSQIISKKDFFYLQIPEINLPNSHKG

FYDWMGMNEKMLKHPISNLELWFFPEFLSIYKTYKMKPWFIPSKLLLLNLNRSANKKI

NEKGNFLIASNKKHRNQEEKEPTSRGERRSVLSPQKDTEENYARSNMEKGKNKKQYTK

AELRLFMKRYLLFQLQGDETLNERMINNINVYWFLHKLIDLTKITISSIQKKQMSLDI

MINNNLTLSEFMQKGVFILEPIRLSEQKDGQFIMYQTVGISLVHKNKHQKYQEQGHAS

NNNFDLLVPENILSFRRRRKLRILICFNSKKRNYIDQNPVFWNVKSSSQVLHDNNHLD

RDKNQLMKLKLFLWPNYRLEDLACMNRYWFDTNNGSRFGMLRIQMYPRLKIF"

gene 114199..114471

/gene="rps15"

CDS 114199..114471

/gene="rps15"

/codon_start=1

/transl_table=11

/product="ribosomal protein S15"

/translation="MVKNSSISLISQKENKETRGSVEFQVFSFTTKIRKLTSHLELHK

KDFSSQRGLRKILGKRQRLLAYLSKINRGRYKELIGELDIREIKTR"

gene 114573..115754

/gene="ndhH"

CDS 114573..115754

/gene="ndhH"

/codon_start=1

/transl_table=11

/product="NADH dehydrogenase subunit H"

/translation="MIPPTTRKDLMIVNMGPHHPSMHGVLRLIVTLDGEDVINCEPVL

GYLHRGMEKIAENRTIIQYLPYVTRWDYLATMFTEAITVNGPEQLGNIQVPKRASYIR

AIMLELSRIASHLLWLGPFMADIGAQTPFFYIFRERELIYDLFEAATGMRMMHNFFRI

GGVAADLPHGWIDKCLDFCDYFLTGVAEYQKLITRNPIFLERVEGVGIIGGEEALNWG

LSGPTLRASGIQWDLRKVDRYECYEEFDWEIQWQKEGDSLARYLVRIGEMTESIKIIQ

QALEGIPGGPYENLESRRFDRIKDPEWNDFEYRFISKKPSPTFELSKQELYVRVEAPK

GELGIFLIGDRSVFPWRWKIRPPGFINLQILPQLVKRMKLADIMTILGSIDIIMGEVD

R"

gene 115756..117921

/gene="ndhA"

CDS join(115756..116308,117383..117921)

/gene="ndhA"

/codon_start=1

/transl_table=11

/product="NADH dehydrogenase subunit A"

/translation="MIIDTTKIQAINSFFRLGSLKEVYGIIWMLIPIFILVLGITLGV

LVIVWLEREISAGIQQRIGPEYAGPFGILQALADGIKLLFKENILPSRGDTRLFSLGP

SIAVISILLSYSVIPFSYRFILADLSIGVFLWIAVSSLAPVGLLMSGYGSNNKYSFLG

GLRAAAQSISYEIPLTLCVLSISLLSNSLSTVDIVEAQSKYGFWGWNLWRQPIGFIVF

LISSLAECERLPFDLPEAEEELVAGYQTEYSGIKFGLFYVASYLNLLVSSLFVTVLYL

GGWNLSIPYLFVPELFDINKRSPVFGTIIGIFITLAKTYLFLFISIATRWTLPRLRMD

QLLNLGWKFLLPISLGNLLLTTSSQLLSL"

exon 115756..116308

/gene="ndhA"

/number=1

intron 116309..117382

/gene="ndhA"

/number=1

exon 117383..117921

/gene="ndhA"

/number=2

gene 118008..118514

/gene="ndhI"

CDS 118008..118514

/gene="ndhI"

/codon_start=1

/transl_table=11

/product="NADH dehydrogenase subunit I"

/translation="MFPMLTQFLNSGQQTIRAARYIGQGFMITLSHANRLPVTIQYPY

EKLITSERFRGRIHFEFDKCIACEVCVRVCPIDLPVVDWKLQTDIRKKRLLNYSIDFG

ICIFCGNCVEYCPTNCLSMTEEYELSAYDRHELNYNQIALGRLPVSIIEDYTIRTISS

NLPQIKNV"

gene 118897..119427

/gene="ndhG"

CDS 118897..119427

/gene="ndhG"

/codon_start=1

/transl_table=11

/product="NADH dehydrogenase subunit G"

/translation="MDLPGPIHDFLLVFLGLGLILGSLAVVLLPNPIYSAFSLGWVLF

CISLFYILSNSYFVAAAQLLIYVGAINVLIIFAVMFMNGSDYYKDFHLWTVGDGVTSI

VCISLFISLITTIPDTSWYGIIWTTKSNQILEQDLISNSQQIGIHLATDFFLPFELIS

IILLVALIGAIAIARQ"

gene 119643..119948

/gene="ndhE"

CDS 119643..119948

/gene="ndhE"

/codon_start=1

/transl_table=11

/product="NADH dehydrogenase subunit E"

/translation="MMLEHILVLSAYLFSIGIYGLITSRNMVRALMCLELILNSVNIN

FVTFSDIFDNRQLRGDIFSIFVITIAAAEAAIGLAIVSSIYRNRKSTRINQSNLLNK"

gene 120211..120456

/gene="psaC"

CDS 120211..120456

/gene="psaC"

/codon_start=1

/transl_table=11

/product="photosystem I subunit C"

/translation="MSHSVKIYDTCIGCTQCVRACPTDVLEMIPWDGCKAKQIASAPR

TEDCVGCKRCESACPTDFLSVRVYLWHETTRSMGLAY"

gene 120572..122074

/gene="ndhD"

CDS 120572..122074

/gene="ndhD"

/codon_start=1

/transl_table=11

/product="NADH dehydrogenase subunit D"

/translation="-

NHFPCLTIIVVLPIFAGCFIFFLPHRGNRVIRWYTICMCILELLLTTYAFCYHFQSDD

PLIQLMEDYKWIHFLDFHWRLGIDGLSIGPILLTGFITTLATLAAWPVTRDSRLFHFL

MLAMYSGQIGLFSSRDLLLFFLMWELELIPVYLLVSMWGGKKRLYSATKFILYTAGGS

VFLLMGVLGIGLYGSTEPTLNFEILANRSYPVNLEILFYIGFFLAFAVKLPIIPLHIW

LPDTHGEAHYSTCMLLAGILLKMGAYGLVRINMELFPHAHSIFSPWLMVVGAMQIIYA

ASTSLGQRNLKKRIAYSSVSHMGFIIIGIGSITDMGLNGALLQIISHGFIGAALFFLA

GTTYDRIRLVYLDEMGGIGIPMPKIFTMFSSFSMASLALPGMSGFVAELIVFFGLITS

PKYLLMTKLPITFVMAIGMILTPIYLLSMLRQMFYGYKIFNGPDSYFFDSGPRELFLS

VSIFLPVLGIGMYPDFVLSLSVEKVEVILSNSFFR"

gene complement(122255..123226)

/gene="ccsA"

CDS complement(122255..123226)

/gene="ccsA"

/codon_start=1

/transl_table=11

/product="cytochrome c biogenesis protein"

/translation="MIFSTLEHILTHISFSIVSIIITIHLITFLVDEIVKLDDSSKTG

MIMTFFCITGLLISRWIYSGHFPLSDLYESLIFLSWSFSFIYIVSYFKKNQNILSTII

GPSAIFSQGFATSGLLTEIHESTILVPALQSEWLIMHVSMMILGYAALLCGSLLSVSL

LVITVRKKLSLFSTSNHLLNLNESFFLGEIEYMNEQRDFFQKTSFFFARNYYRSQLIQ

KLDYWSYRVISLGFIFLTIGILSGAVWANEAWGSYWSWDPKETWAFITWIIFSIYLHT

RTNIKLKGTNSAIVASIGFLIIWICYFGVNLLGIGLHSYGSFTLTSN"

gene complement(123311..123390)

/gene="trnL-UAG"

tRNA complement(123311..123390)

/gene="trnL-UAG"

/product="tRNA-Leu"

gene complement(123920..124096)

/gene="rpl32"

CDS complement(123920..124096)

/gene="rpl32"

/codon_start=1

/transl_table=11

/product="ribosomal protein L32"

/translation="MAVPKKRTSASKKRIRKNFWKRKGYRAALKAFSLGKSLSTGNSK

SFFVRQTNKLSNKT"

gene 124603..126825

/gene="ndhF"

CDS 124603..126825

/gene="ndhF"

/codon_start=1

/transl_table=11

/product="NADH dehydrogenase subunit F"

/translation="MEQTYQYACILPFVPLLVPILIGVGLVIFPTATKNLHRMWAFPS

ILLLSIVMIFSTNLSIQQINSSYIYQYVWSWTLDNDFSLEFGCLIDPLTSIMLMLITT

VGIMVLIYSDNYMAHDQGYLRFFAYMSFFNTSMLGLVTSSNLIQIYIFWELVGMCSYL

LIGFWFTRPPAANACQKAFVTNRVGDFGLLLGILGFYWITGSFEFRDLFEILNNLISN

NEVNFPFVILCAALLFAGAVAKSAQFPLHVWLPDAMEGPTPISALIHAATMVAAGIFL

VARLLPLFVVIPYIMNLIALMGIITLLLGATLALAQKDIKRSLAYSTMSQLGYMMFAL

GMGSYRSALFHLITHAYSKALLFLGSGSVIHSMETLVGYSTDKSQNMVLMGGLTKHVP

ITKTSFLLGTLSLCGIPPLACFWSKDEILNDSWLYSPIFAIIALATAGLTAFYMFRIY

LLTFEGHLNIHFQNYSGNKNTSFYSISIWGKGYSKRINPNFSLLRNESSSFFWKKTCR

SDENARKKGGGHPFINILHFDNQKSFSYPYESANTMLFSLLLLVLFTLFVGSLGIPFN

QKGTDLDLLSKWLAPSINLLHQKSKDSASWYEFFKDALLSVSIAYCGIFLASFLYKPI

YSSFQNFDLINSFVKLGPKRKRLDKIINALYDWSYNRAYIDSFYTISFSRGVRELAQL

THFFDRRVIDGITNGVGVMSFFLGEGIKYLGGGRISSYLFFYFSFVSIFLISSLFSVF

"

gene complement(126771..127889)

/gene="ycf1"

CDS complement(126771..127889)

/gene="ycf1"

/codon_start=1

/transl_table=11

/product="Ycf1 protein"

/translation="MIFQSFLLGNLVSLCMKIINSVVVVGLYYGFLTTFSIGPSYLFL

LRAQVMEEGTEKKVSATTGFITGQLMMFISIYYAPLHLALGRPHTITVLALPYLLFHF

FWNNHKHFFDYGSTTRNSMRNFSIQCLFLNNLIFQLFNHFILPSSMLARLVNIYMFRC

NNKMLFVTSSFVGWLIGHILFMKWLGLVLVWIRQNHSIRSNVLIRSNKYLVSELINSK

ARILSILLFITCVYYLGRIPSPLFTKKLKETSKTGAGERVESAEERDVEIETASEMKG

TKQEQEGSTEEDPSPYFFSEERADPNKIDETEEIQVNGKEKEFHFRFTETGYQNRPVS

EESYLMNINENQDNSRLKIFDQKTENKELIKKIDTKEK"

repeat_region 126795..152432

/note="inverted repeat B (IRB)"

/rpt_type=inverted

gene 128215..128286

/gene="trnN-GUU"

tRNA 128215..128286

/gene="trnN-GUU"

/product="tRNA-Asn"

gene complement(128860..128932)

/gene="trnR-ACG"

tRNA complement(128860..128932)

/gene="trnR-ACG"

/product="tRNA-Arg"

gene complement(129167..129287)

/gene="rrn5"

rRNA complement(129167..129287)

/gene="rrn5"

/product="5S ribosomal RNA"

gene complement(129543..129645)

/gene="rrn4.5"

rRNA complement(129543..129645)

/gene="rrn4.5"

/product="4.5S ribosomal RNA"

gene complement(129744..132554)

/gene="rrn23"

rRNA complement(129744..132554)

/gene="rrn23"

/product="23S ribosomal RNA"

gene complement(129744..131996)

/gene="rrn23"

rRNA complement(129744..131996)

/gene="rrn23"

/product="23S ribosomal RNA"

gene complement(132243..132554)

/gene="rrn23-fragment"

rRNA complement(132243..132554)

/gene="rrn23-fragment"

gene complement(132746..133624)

/gene="trnA-UGC"

tRNA complement(join(132746..132780,133587..133624))

/gene="trnA-UGC"

/product="tRNA-Ala"

exon complement(132746..132780)

/gene="trnA-UGC"

/number=2

intron complement(132781..133586)

/gene="trnA-UGC"

/number=1

exon complement(133587..133624)

/gene="trnA-UGC"

/number=1

gene complement(133689..134697)

/gene="trnI-GAU"

tRNA complement(join(133689..133723,134661..134697))

/gene="trnI-GAU"

/product="tRNA-Ile"

exon complement(133689..133723)

/gene="trnI-GAU"

/number=2

intron complement(133724..134660)

/gene="trnI-GAU"

/number=1

exon complement(134661..134697)

/gene="trnI-GAU"

/number=1

gene complement(134987..136477)

/gene="rrn16"

rRNA complement(134987..136477)

/gene="rrn16"

/product="16S ribosomal RNA"

gene complement(136705..136776)

/gene="trnV-GAC"

tRNA complement(136705..136776)

/gene="trnV-GAC"

/product="tRNA-Val"

exon 138392..138624

/gene="rps12"

/number=2

exon 138392..138623

/gene="rps12"

/number=2

intron 138624..139159

/gene="rps12"

/number=1

intron 138625..139160

/gene="rps12"

/number=2

exon 139160..139185

/gene="rps12"

/number=3

exon 139161..139185

/gene="rps12"

/number=3

gene 139239..139706

/gene="rps7"

CDS 139239..139706

/gene="rps7"

/codon_start=1

/transl_table=11

/product="ribosomal protein S7"

/translation="MSRRGTAEEKTAKSDPIYRNRLVNMLVNRILKHGKKSLAYQIIY

RAMKKIQQKTETNPLSVLRQAIRGVTPDIAVKARRVGGSTHQVPIEIGSTQGKALAIR

WLLAASRKRPGRNMAFKLSSELVDAAKGSGDAIRKKEETHKMAEANRAFAHFR"

gene 139981..142192

/gene="ndhB"

CDS join(139981..140757,141437..142192)

/gene="ndhB"

/codon_start=1

/transl_table=11

/product="NADH dehydrogenase subunit B"

/translation="MIWHVQNENFILDSTRIFMKAFHLLLFDGSFIFPECILIFGLIL

LLMIDSTSDQKDIPWLYFISSTSLVMSITALLFRWREEPIISFSGNFQTNNFNEIFQF

LILLCSTLCIPLSVEYIECTEMAITEFLLFVLTATLGGMFLCGANDLITIFVAPECFS

LCSYLLSGYTKKDVRSNEATMKYLLMGGASSSILVHGFSWLYGLSGGETELQEIVNGL

INTQMYNSPGISIALIFITVGIGFKLSPAPSHQWTPDVYEGSPTPVVAFLSVTSKVAA

SASATRILDIPFYFSSNEWHLLLEILAILSMILGNIIAITQTSMKRMLAYSSIGQIGY

VIIGIIVGDSNDGYASMITYMLFYISMNLGTFACIVLFGLRTGTDNIRDYAGLYTKDP

FLALSLALCLLSLGGLPPLAGFFGKLYLFWCGWQAGLYSLVLIGLLTSVVSIYYYLKI

IKLLMTGRNQEITPHVRNYRRSPLRSNNSVELSMIVCVIASTIPGISMNPIIAIAQDT

LF"

exon 139981..140757

/gene="ndhB"

/number=1

intron 140758..141436

/gene="ndhB"

/number=1

exon 141437..142192

/gene="ndhB"

/number=2

gene 142742..142822

/gene="trnL-CAA"

tRNA 142742..142822

/gene="trnL-CAA"

/product="tRNA-Leu"

gene complement(143083..143331)

/gene="ycf15"

CDS complement(143083..143331)

/gene="ycf15"

/codon_start=1

/transl_table=11

gene complement(143374..150219)

/gene="ycf2"

CDS complement(143374..150219)

/gene="ycf2"

/codon_start=1

/transl_table=11

/product="Ycf2 protein"

/translation="MKGHQFQSWIFELREIKNSHCFLDSWTQFNSVGSFIRIFFHQER

FLKLFDPRILSILLSRNSQGSTSNRYFTIKGVLLFVVAVLIYRINNRNMVERKNLYLR

GLLPIPMNSIGPINDTLEESVGSSNINRLIVSLLYLPKGKKISESCFLNPKESTWVLP

ITKKCSMPESNWGSRWWRNWIGKKRDSSCKISNETVTGIGILFKEKDLKYLEFLFVYY

MDDPIRKDHDWELFDRLSLRKRRNRINLNSGPLFEILVKHWISYLMSAFREKIPIEVE

GFFKQQRAGSTIQSNDIEHVSHLLSRNKRAISLQNCAQFHMWQFRQDLFVSWGKNPHE

SDFLRNVSRENWIWLDNVWLVNKDRFFRKVRNVSSNIQYDSTRSSFVQVTDSSQLKGS

SDQSRDHLDSISNEDSEYHTLINQREIQPLKERSILWDPSFLQTEGTEIESDRFPKCL

SGYSSMSRLFTEREKQMINHLLPEEIQEFLGNPTRSVRSFFSDRWSELHLGSNPTERS

TRDPKLLKKQQDLSFVPPRRSENKELVNIFKIITYLQNTVSIHPISSDPGCDRVLKDE

PDMDSSNKISFLNKNPFFDLFHLFHDRNRGGYTLHHDFESEERFQEMADLFTLSITEP

DLVYHKGFPFSIDSYGLDQKQFLNEARDESKKKSLLVLPPIFYEENESFSRRIRKKWV

RISCGNDLEDPKPKIVVFASNNIMEAVNQYRLIRNLIQIQYSTYGYIRNVWNRFFLMN

RSDRNFEYGIQRDQIGKDTLNHRTIMKYTINAHLSNLKKSQKKWFDPLILISRTERSM

NRAPDAYRYKWSNGSKNFQEHLEHFVSEQKSRFQIVFDRLRINQYSIDWSEVIDKKDL

SKPLRFFLSKSLLFLSKLLFFLSNSLPFFCVSFGNIPIHRSEIYIYELKGPNDQLCNQ

LLESIGLQIVHLKKWKPFLLDEHDTSRKSKFLINGGTPFLFNKIPKWMIDSFHTRNNR

RKSFDNADSYFSMIFHNQDNWLNPVKPFHISSLISSFYKANRLRFLNNPHHFCFYCNT

RFPFSVEKARINNYDFTYGQFLNILFIRNKIFSLCVGKKKHAFGGRDTISPIESQVSN

IFIPNDFPQSGDETYNLYKSFHFPSQHDPFVRRTIYSIADMFGTPLTEGQIVHFERTY

CQPLSDMNLSDSEGKNLHQYLNSNVGLIHTPCSEKYLPSEKRKKRSLCLKKCVEKGQM

YRTFQRDGAFSTLSKWNLFQTYIPWFLTSTGYKYLNLIFLDTFSDLLPILSSSQKFVS

IFHDIMHGSGIAWRILQKKWCLPQWNLISAISSKCFHNLLLSEEMIHRNNESPSTHLR

SPNVREFLYSILFLLLVAGYLVRTHLLFVSRASSELQTEFEKVKSLMIPSSMIELRKL

LDRYPTSAPNSFWLKNLFLVALEQLGDSLEEIRASGGNMPGPAYGVKSIRSKKKYLSI

NLIDLIPNPINRITFSRNTRHLSHTSKEIYSLIRKRKNVNGDWIDDKIESWVANSDSI

DDEEREFLVQFSALTTEKRIDQILLSLTHSDHLSKNDSGYQMIEQPGAIYLRYLVDIH

KKYLLNYEFNTSSLAERRVFLAHYQTITYSQTSCGTNTLHFPSHGKPFSLRLALSPSR

GILVIGSIGTGRSYLVKYLATNSYVPFITVFLNKFLDNKPKGFLFDDIDIDASDDIDA

SDDIDASDDIDASDDIDRDLDTELELLTMDRIPEIDRFYITLQFELAKAISPCIIWIP

NIHDLDVNESNYLSLGLLVNHLSERCSTRNILVIASTHIPQKVDPALIAPNKLNTCIK

IRRLLIPQQRKHFFTLSYTKGFHLEKKMFHTNGFGSITMGSNARDLVALTNEALSISI

TQKKSIIDTKTIRSALHRQTWDLRSQVRSVQDHGILFYQIGRAVAQNVLLSNCPIDPI

SIYMKKKSCNEGDSYLYKWYFELGTSMKKLTILLYLLSCSAGSVAQDLWSLPGSDEKN

GITSYGLVENDSDLVHGLLEVEGALVGSSRTEKDCSPFDNDRVTLLLRPEPRNPLDMM

QNGSCSIFDQRFLYEKYESEFEEGKGEGALDPQQIEEDLFNHIVWAPRIWRPWAFLFD

CIERPNELGFPYWSRSFRGKRIIYDEEDELQENDSEFLQSGTMQYQTRDRSSKEQGLF

QISQFIWDPADPLFFLFKDQPPGSVFSHRELFADEEMSKGLLTSQMDPPTSIYKRWFI

KNTQEKHFELLINRQRWLRTNSSLSNGSFRSNTLSESYQYLSTLFLSNGTLLDQMTKT

LLRKRWLFPDEMKIGFMEQEKDFPFLSRKDMWL"

gene 150308..150381

/gene="trnI-CAU"

tRNA 150308..150381

/gene="trnI-CAU"

/product="tRNA-Ile"

gene 150547..150828

/gene="rpl23"

CDS 150547..150828

/gene="rpl23"

/codon_start=1

/transl_table=11

/product="ribosomal protein L23"

/translation="MDGIKHAVFTDKSIRLLGKNQYTSNVESGSTRTELKHWVELFFG

VRVIAMNSHRLPGKGRRMGPIMGHTMHYRRMIITLQPGYSIPPLRKKRT"

gene 150847..152338

/gene="rpl2"

CDS join(150847..151237,151905..152338)

/gene="rpl2"

/codon_start=1

/transl_table=11

/product="ribosomal protein L2"

/translation="MAIHLYKTSTPSTRNGTVDSQVKSNPRNNLIYGQHHCGKGRNAR

GIITSRHRGGGHKRLYRKIDFRRNEKDIYGRIVTIEYDPNRNAYICLIHYGDGEKRYI

LHPRGAIIGDTIVSGTEVPIKMGNALPLTDMPLGTAIHNIEITLGKGGQLVRAAGAVA

KLIAKEGKSATLKLPSGEVRLISKNCSATVGQVGNVGANQKSLGRAGSKRWLGKRPVV

RGVVMNPVDHPHGGGEGRAPIGRKKPTTPWGYPALGRRSRKRNKYSENLIVRRRSK"

exon 150847..151237

/gene="rpl2"

/number=1

intron 151238..151904

/gene="rpl2"

/number=1

exon 151905..152338

/gene="rpl2"

/number=2

gene 152391..152432

/gene="rps19-fragment"

CDS 152391..152432

/gene="rps19-fragment"

/codon_start=1

/transl_table=11

ORIGIN

1 aaaactgctc agcaacagtc ggacaagtgg ggaatgttgg ggcgaaccag aaaagtttgg

61 gtagagccgg atccaagcgt tggctaggta agcgtcctgt agtaagagga gtcgttatga

121 accctgtaga ccatccccat gggggtggtg aagggcgagc cccaattggt agaaaaaaac

181 ccacaacccc ttggggttat cctgcacttg gaagaagaag tagaaaaagg aataaatata

241 gtgaaaattt gattgttcgt cgccgtagta aataggcgag aaaatagaat ttctttcttc

301 gtctttaaaa aaaaatagga gtaagctgtg atacgttcac taaaaaaaaa tccttttgta

361 gccaatcatt tattaagaaa aattgacaag cttaataaaa aagcagaaaa agaaataatc

421 gtaacttggt cccgtgcatc taccattgta cccacaatga tcggccatac gattgctgtt

481 cataatggta aggagcattt gcctatttat ataacagatc gtatggtagg tcacaaattg

541 ggagaatttg tacctacttt gaatttccaa ggacacgcaa aaagcgataa tagatcccgc

601 cgttaatctt atcttaaaaa acatatagat agatacttat gattcattag taggagagaa

661 accttatgct aaagaagaaa aaaacagaag tatacgcttt gggtcgacat atatctttat

721 ccgctgacaa agcaagaaga ataattgatc aaattcgcgg tcgttcctat gaggaaacac

781 ttatgatact agaactcatg ccctataaag catgttatcc cattttcaaa ttggtttatt

841 ctgcagcagc aaatgcgagt ttcaatatgg gttccaacga ggccaattta gtaattcgta

901 aagctgaggt taacgagagt actgcctcga agaaattaaa actccgggct cgaggacgta

961 gttatgcgat aaaaaaagct acctgtcata taactattgt agtgaaagat atatctttag

1021 atgaatatga agagatagct ttctattcgt taaaaaaccc tagatggaaa aagacaacta

1081 tggtatatga tgatgcgtat aatagtgagg tagtatggga caaaaaataa atccacttgg

1141 tttccgactt ggtacaaccc aaagccacca ttccctttgg tttgcacaac caaaaaatta

1201 ttctgagggt ctacaagaag atcaaaaaat aagagatttt atcaaaaatt atgttcaaaa

1261 gaatatgaga atatcctccg gtgccgaggg aattgcccgc atagagattc aaaaaagaat

1321 tgatttgatc caggtcagaa tctttatggg gttcccgaag ttattaatcg aaagtagacc

1381 gcgaggaatc gaagaattac agatgaatct acaaaacgaa tttcattatg tgaaccgaaa

1441 acttaacatt gctatcacaa gaattgcaaa accttatgga aatcctaata ttcttgcaga

1501 atttatagcc ggacaattaa agaatagagt ttcatttcga aaagcaatga aaaaggctat

1561 tgaattgact gaacaagcag atacaaaagg aattcaagta caaatcgcag ggcgtatcga

1621 cggaaaagaa attgcacgtg tcgaatggat cagagaaggt cgggttcccc tacaaaccat

1681 tcgcgctaaa attgattatt gttcctatac agttcggact atctatgggg tattaggcat

1741 caaaatttgg atttttatag acaagggaga ggaataacaa aactttcgtt gtttttccgt

1801 cgatagaaca aaaaggggga aactcattca ttctttttct ggccaatcaa acaaattccg

1861 aattctttaa ttctataggg ttgaataaaa attagattga ccttttgttt tgatataatt

1921 gctatgctta gtgtgtgact cgttggtttt agggggttgg gattaaaaaa agaccggccc

1981 agtagtatga aaaccaaccc atcgcttcat attatctgga tctaaagaac ctgtcaagat

2041 atgccaaatc ggtcatatct ttgtagcaac tgaaattttt ttacaattaa actttaataa

2101 attctaagtt taaaacaaaa tacagaacaa gagtgtggat aaatggaagg gtgagagaaa

2161 gaaagaaaaa aatctcaatg atatagaatt ccaatatgta aggtctatga gtcctctcat

2221 aaaagacagt gtaataaagc atcaatacta attgattcat ccataatgaa atattaaatg

2281 aatccttctt atagattata gcttatagat tatagaagaa aaaactcaag agcttcgagc

2341 caataaagac taagaagatt gactcaagga taaattggat tagaagcttc gttgtagaat

2401 tctgacctaa ccatttagta cgaagtggtg gggacgaagg aacctgtgaa tgcaaaagat

2461 tttattgaac aaatgaatct taatgattca ctcgttagga tggcgaaatg aaccggaaat

2521 caattcatct attcggagaa atgacgaaca agtgctagga ctgaaataga gattgcaaga

2581 gtcaatattc gcccgcgata atgtgttttt tttttgaatt tgaattggta aacctggata

2641 aaagacaaaa gaaaataaag atttaatagg acgttccaaa aaaaaaacga gttttatcca

2701 attttttcta aaaatgttct aatatctatg caaattaatt gcaattccat tttgaatcct

2761 tttattcgcg aggagctgga tgagaagaaa ctctcacgtc cagttctgta gtagagatgg

2821 acttccgaaa caaccatcaa ttataacccc aaaagaacta gattccgtaa acaacataga

2881 ggacgaatga agggaatatc ttatcgaggt aatcatattt gtttcggtaa atatgctctt

2941 caggcgcttg agcctgcttg gatcacatct agacaaatcg aagcgggtcg acgagcaatg

3001 acacgaaatg cacgtcgtgg tggaaaaata tgggtccgta tatttccaga caaaccagtt

3061 acaataagac ccgccgaaac acgtatgggt tcggggaaag gatcccctga atattgggta

3121 gctgttgtta aaccggggcg aatactatat gaaatgggcg gagtaactga aaatatagct

3181 agaagggcga ttttaatagc agcatccaaa atgcctatac gaactcaatt tattatttcg

3241 gcataaaaat gtagaaccaa cacaaatggg tcttgggaat gaaagaaaac cgcaggtttc

3301 ttttttttga caaacaatat ttcttttatt ttcttcatcc tttgcattgg aagaatagac

3361 tcaaaacttc atatgattca acctcagacc catttaaatg tagcggataa cagcggggct

3421 cgaaaattga tgtgtattcg aatcctagga gctagtaatc gccgatatgc tcatattggt

3481 gacgttattg ttgctgtgat caaagaagca gtaccaaaca tgcccctaga aaaatcagaa

3541 gtagtaagag ctgtaattgt tcgtacctgt aaagaactta aacgtgacag cggtatgata

3601 atacgatatg atgacaatgc tgcagttgtg attgatcaag aaggaaatcc aaaaggaact

3661 cgaatttttg gtgcaatccc ccgcgaactg agacaattca attttactaa aataatttca

3721 ttagctcccg aggtattata aaataaaatg ggagcctgat atctttggga tctttgaaaa

3781 gaaatagatt aagaactaga ttaattcgta gattatgtct cacgcatata ccttgaaaaa

3841 ttcatattca taaaccaata aaaaaacatg ttaattagat ccaattttga ggcaccaaaa

3901 attttacttc atcatgggta gagacactat tgctgagata ataacctcta tacgaaatgc

3961 ggatatggat agaaaaagag ttgttcgcat agcatctact aatattaccg aaaatattgt

4021 taaaatactt ttccgagaag gttttctcga aaacgtcaga aaacatcgag aaaaaaacaa

4081 aaattttttg gttttaaccc tgcgacataa tagaaggaat aggaaaagac cccatacctg

4141 tagaaatttt ttaaatttaa aacggatcag ccgacccggt ctacgaatct attctaactc

4201 tcaacgaatt cctagaattt taggtggaat ggggattgta attctttcta cttctcgagg

4261 tataatgaca gaccgggagg ctcgactaga aagaatcggc ggagaaattt tatgttatat

4321 atggtaatcc ttttaatatc caaatgggat ccaaaacctc ttcctatttg taaaaaaaaa

4381 agaaaggggt gagttgtcta atatcgtttc tcctacatta gttgatactt caagggggct

4441 ttacctgaaa tgaaagaaca aaaatggatt catgagggtt taattaccga atcgcttccc

4501 aatggcatgt tccgggttcg gttagataat gaagatctga ttataggtta tgtttcagga

4561 aagatccgac gtagttttat acggatactg ccaggcgata aagtcaaaat tgaagtaagt

4621 cgttatgatt caactagagg acgtataatt tatcgactcc gaaacaagga ttcgaaagat

4681 taggtggttt ttattcaata tgattcgaga tgaaaaattg caagaaactt attttctacc

4741 aagaagtaga ttcagaatta agataaggaa tgaaaaatat gaaaataaga gcttccgtcc

4801 gtaaaatttg tgaaaaatgt cgactaatcc gcagacgggg gcgcattaga gtaatttgct

4861 ctaacccgag acataaacaa agacaaggat aatcagactc accaaaggaa taaacgtaca

4921 aataaagaat ctgttttgac atcaaatgga tatattccat atatttctga ctcatattta

4981 tgagatgcta caatatggca aaagctatac cgagaattgg ttcacgtaaa aatgtacgta

5041 ttggttcacg taaaagtgca cgtagaatac caaagggagt tattcatgtt caagcaagtt

5101 tcaataatac tattgtcacc gttacagatg tacggggtcg ggtcgtttcc tggtcctcgt

5161 ccggtacttg tggattcaag ggtacgagaa gggggacgcc ctttgccgct caaaccgcag

5221 cggcaaatgc tattcgtaca gtagtagatc aaggtatgca acgagccgaa gtcatgataa

5281 aaggtcccgg tctcggaaga gacgccgcat tacgagctat tcgtagaagt ggtatactat

5341 taacttttgt gcgggatgta acccccatgc cacataatgg ctgcagaccc cccaaaaaaa

5401 gacgtgtgta gaaatgaaga gtgaagaaat ttcacgagaa acaagagaaa taaataattc

5461 aatcaaataa aatattatta ctatggttcg agagaaagta acagtatcta ctcggacgct

5521 gcagtggaag tgtgttgaat caagaacaga cagtaaacgt ctttattacg ggcgctttat

5581 tctgtctcca cttatgaaag gacaggccga cacaataggc attgcgatga gaagagcttt

5641 gcttggagaa atagaaggaa catgtatcac acgcgtaaaa tctgagaatg tcccgcatga

5701 atattcgact ataacgggta ttcaagaatc ggtccacgaa attataatga atttgaaaga

5761 aattgtattg agaagtaatc tatatggaac ttgtggcgcg tcgatttgcg ccatgggccc

5821 tggatatgta actgctcaaa atatgatctt accaccttat gtggaaatcg tcgacaatac

5881 acaacatata gctagcttag cagaacccat taatttgtgt attggattag aaatcgagag

5941 aaatcgggga tatcttatca aaatgccaca tacctttcaa gatggaagtt atcctataga

6001 tgctgtattc atgcctgttc gaaacgtgaa tcatagtatt cattcctatg aaaatgggaa

6061 tgaaaaacaa gagatactat ttctcgaaat atggacaaat gggagtttaa ctccgaaaga

6121 agcacttcat gaagcctccc ggaatttgat tgatttgttt attccctttt tatataagga

6181 agaaaaaaac ttacctttag aggacaatca atatacgctt cccttatccc cttttacttt

6241 tcacgataaa ttggataaag tacgaaaaaa caaaaagaaa atagcattga aatcgatttt

6301 tattgatcaa tccgaattgt ctcccagggt ttataattgc ctcaaaaggt ccaatatata

6361 tacattattg gaccttttga ataatagtca agaagatctt atgaaaattg aagattttcg

6421 cctagaagat gtaaaacaga tattgggcat tctagaaaaa catttcgcaa ttgatttacc

6481 aaaaaacaag ttttaaatca attggattta attaagatat aagagttgaa tctgtagcac

6541 aattcatata ttcgaaagaa attgataatt ttattgaata gatgtatcta gggaaaattc

6601 gctttgaagc aactattccc tagatacata cgtcgtgtta tttcacaatt gaatcaaatt

6661 aaaaaagacc taaagtgagg gatttatcaa taggtaatgt tgcaccaata cccaaccaaa

6721 gagcgactgc ggtaccaatc aaaaagacgg ttgtcgctac tggacggcga aatggatttt

6781 ggaatttatt aacattctct aaaaagggta ctgttaataa tcccgcgggt actgaaacca

6841 ttaaaagaac acccaataat ttattgggca ctgtacgaag tatttgaaat acgggaaaga

6901 aataccattc aggcaatatt tccagagggg ttgcaaatgg atctgccggt tcaccaatca

6961 ttgacggttc tagaaccgct aagcctacgt tacatgcaat agtacctaga attactactg

7021 gaaaaatata caaaagatcg ttgggccatg cgggttctcc gtaataatta tgacccatac

7081 ctttagccaa tttagctctt aatacaggat cattcaaatc gggttttttt gttattggga

7141 taggtgaatt cttatggatc catcccccga aggaaccgga catgataatt ttttatcatc

7201 cagctcgagc aagactcaaa agaaccaaat gaatccataa aaaatctatg tgttggctat

7261 atctacacat atatgaatga ttcaatgtaa gaaaaattgg actcttttca ggaattgcaa

7321 ctaccccgtt gcaaagaatt cagctctaag tctaagtaaa tactcaatac ccaagttggc

7381 ttacaaagtg gatcatgata tgatcaagtg ctttttgggt cgtctcagac tttacgatta

7441 ctatgttatc tccaaaatag ttggagctac acacacaaaa ggtttccttg ttactaattt

7501 agtctagccc atatttttct ttagatctct tgtttcaccc cgatagtatt atcgatcata

7561 tcaatgcaga ggaaatgaat gcatttccat actattccat tttaaataag taggaaattc

7621 attaagtgaa atagataagg attctgtttt atttgatcta tttcacttaa ttatactaga

7681 ttttacggag cccactcatg tacaacacga tttagatctg atcatagaaa agattctctt

7741 caagtgaacc agcctatctt ctgtatagag gttacacagt ggttgtaaca aagacacatt

7801 tggttgtgaa ttctaatacg gcagatagat atctacatag accaatcaat agttcaagtc

7861 acacactccc ataatccatt ttctcttcgt agaattccct tcaactagaa aatcttattt

7921 atttgacatg attagttgaa gggaaatatc caaattcatg tcttattatt ttcaataatc

7981 catacttgta gcaataaaat cctattattc ctcctctaag tgataaatga ttgattacaa

8041 atccaaatat ctattatatt tctataaagg accagaaata ccttgtttac gtatcattgg

8101 aaagtgcatt aacataaata cagcagtaag aagcggcaat acaaaagtgt gtaaactata

8161 aaaacgagtc aaggtggatt gtcccacact tgcacttccg cgcaataatt ctaccacagg

8221 ggatcctatt acaggaatag cctcaggtac acctgttaca attttcaccg cccaataacc

8281 aatttggtcc cgaggtaagg aataaccagt tacgccaaaa gatgcggtca atactcccag

8341 aaccacaccc gtaacccaag tcaattcgcg gggtttttta aatccaccgg tgagatacac

8401 acgaaaaaca tgtagaatca tcattaggac catcatactt gccgaccatc gatgaactga

8461 tcggattaac caaccaaagt tagcttccgt cattatgtat tgaacagagg caaaagcttc

8521 agtaacggtc ggacggtagt aaaaagtcat agcaaaccct gtagctactt gtactaaaaa

8581 acaggtaagc gtaattcccc ctaaacaata aaatatattg acatggggag gaacatattt

8641 actagttata tcatccgcaa tcgcttgaat ctcgagacgt tcttcgaacc aatcatagac

8701 tttattgaga taggcgaaaa cccccctccc agaaccgtat atgagacttt catctcgtac

8761 agctcaagca aaaacacccc aataaaaaaa aagaatagtc ggatatgtaa atgaaataga

8821 aagtttgaaa cttctgaatc tccgattcaa tcttctctaa atgtacgaaa gaagaacaaa

8881 tccgaaagct cttctttgtg gtgatactac caaaatatca agttggctca atcgtcttta

8941 tgttgatcct tacgggcctc ttgaatcctc tctttaccta ttttttttct ttttaatttg

9001 gttttgtctc taatctggct tttttacttt ctttattatt gacttgatag gaattctctt

9061 gttaagaccc tatgaatcga ttaaaacctc agattcatac tagaaccacg atgattcaat

9121 aaaaaatcat aagctaaccc aaggattccc tgagtaagaa ccgttggttc atcacatatt

9181 ccataaatta gataaaccga ctaaaaaaaa aaaaaagaaa gatttttctt ttttttttca

9241 acagtttgcg attttttttg cagtccgggc acgaggtcga atattaagta tgaatcatag

9301 ttcaaatatt tcgtaatcta gttcatatag ttcgtgttcc agatactcga ataaatatcc

9361 gacgaagtag aaccatctcc atcaaggcga cagacctatt ctctgtacta tcaatagaat

9421 ccattataac aagtcgcaca ctcatattcc ggaaatacag aaagaaagaa attccacgat

9481 cgaactacca aaatacaata ggccagaaat ttgagttcta actgattgat tttttattca

9541 aaagccagga ctttgtgatt cttatagatt taattcattg aaattccatc caataaaacg

9601 gaagaattat aaatttccaa aataatagat agaaataccg caaatagggc cattgcgaca

9661 cccatcaaag gagtcgttcc ccaaccggga gctactttac catattccga attcaatggt

9721 tttaataaat cccctacgac agttcgtctt ggacccgatt tagaactgtt ctcaacagtt

9781 tgtgtagcca taaattattg tattcattga gatctgttga ctttgtatac cattgcgttg

9841 taaataaacg atcttatgat agatccgttg gggtcttgaa attatataat cataatggaa

9901 acagcaaccc tagtcgccat ctttatatct ggtttacttg taagttttac cgggtacgcc

9961 ttatataccg cttttgggca accttctcaa caactaagag atccattcga ggaacacggg

10021 gactagttga agtaatgagc cttccaatat tgggaggctc attacttcaa ttgagataat

10081 aaaaaattat tttatctttt tactttttag ttggaacttt aggtggttct cgaaaaaaaa

10141 tagcgaaaaa aattatccct agagtcgaga ctaaaaggaa tgtataaacc aatgcttcca

10201 taaatttgat cgtggtttac aattatagct ttcctacctg tttgtttttt cttttttttt

10261 ttcattttct ttttgggaat catctcaaaa aaagcaagaa tcaaaaaagg cggtgattca

10321 aattcactcc ggtactcgat gtaaaattcc ctcaaaaaag aaaatggaga tgaaagatac

10381 caaaggggtc ttggatcaga ctgcctgtct tcttgtagtt ggatccccaa gtttttggaa

10441 tgctccaaac tctacttgag catccaaatc tgggtcaata ccagcaaaaa catctctgaa

10501 caaggttcta gcaccatgcc aaatgtgtcc gaaaaagaag agtaaagcaa acgaagcatg

10561 cccaaaagta aaccaacccc ttggactgct acgaaaaaca ccatcggatt tcaaagtcgc

10621 acgatctaat tcaaaaattt cacccaattg ggcgcgtcta gcatattttt tcacagtagc

10681 aggatcacta taactgactc cattgagttc gccgccatag aactcaacgg ttacacctac

10741 ttgttcaaca ctatacttag attctgccct tctaaaagga acatccgctc taacaattcc

10801 gtcgccgtct accaaaacga ccggaaatgt ttcaaaaaaa gtgggcatac gccgtacaaa

10861 aagctcacgt ccttctttat ctctaaagat agggtgtcct aaccacccaa ccgctattcc

10921 atctccgcta tccattgagc ctgccctgaa taatcctcct tttgccggat tattgccgat

10981 ataatcataa aaagccaact tttcaggaat tttagaccag gcttctgata aactttgatt

11041 ttctgctagc ccggccctaa ctcttcgata tatctcttgc tggaagtaac cctgatccca

11101 ttgataacga gtgggaccaa ataattcgat gggggtagtt gctgaaccat accacatagt

11161 tccagcaact acaaaagctg caaaaaagac agccgcgata ctactggaga gtacggtttc

11221 aatatttccc atacgtaatc ctttgtatag acgttgtggc ggtcgaacgc taagatggaa

11281 tagacccgct aatatgccca atgtaccggc tgcaatatga tgagaagcta ttcctcccgg

11341 aacaaaagga tcaaaaccct ccacgcccca cgccggattt ataggttgta cttttcccgt

11401 tagtccgtaa ggatcagaca cccatattcc aggaccatac aggcctgtta catgaaatgc

11461 accaaaacca aagcaagcca ccccggagag aaataaatga attccaaaga tcttgggcaa

11521 atccaaagaa ggttttcctg tacgttcatc agaaaatatt tctagatccc aatagaccca

11581 atgccagata gctgccaaaa agcataagcc agaaaataaa atatgtgccc cagctacacc

11641 ttcgtaactc caaacccctg tattcgttac agtccctcct gtgatactcc aaccccccca

11701 cgaattggtt attcctaaac gagtcatgaa gggtataacg aacatacctt gtctccacat

11761 tggatcaaga acggggtcag aaggatcaaa aactgctaat tcatagagag ccatcgaacc

11821 cgcccaacca gcaaccagag ctgtatgcat tatatgaacg gaaagcaatc ggccgggatc

11881 attcaataca acggtatgaa cacgatacca aggcaaaccc atggaaaata cccctttatc

11941 aaagaaaaat aaacactacg taactttatt gcattggaat atactatgac tatgctgcgg

12001 acttcccctg ttcagtggat tatttcctaa caaggattat ttattctata ttctattgtg

12061 ttccaaataa tggaacaatt ctgttcgtaa gaacaaagag aagcagattt attctatact

12121 cgataagtac caatacgcaa tggtggattg ataccacttt ctatgagtaa atgcgtttat

12181 cattcgaaag gcctgttcgt aaaaatttcc tttatttttt tttctttctt tctgaatttt

12241 gctaatctat ggataaaata aatatgataa agacagtatt ctaaagacaa taaaaccaca

12301 ttattacgtt tccacatcaa agtgaaatat aggatttagt tcttttttct ttcaatttat

12361 gcctattggt gttccaaaag tacctttccg gagtcctgga gaggaagatg catcttgggt

12421 tgacgtatag tgcgacttgt gagatatatt gggtcatatg ggatttcccc gttctctccc

12481 ccgatcgaga tatcctctgt ttcgcccaag aagtagaaat ggaatcatcc ataaattgga

12541 gcgtgaagtg caattagatg cattgtttgg aggaattcat agtactatta tcaatttgaa

12601 taatttatgg tttactttga ttggactaaa aaaatgaagt atccaggctc cgtttagaaa

12661 aaacccaatt cgtaatatat ctaggattac tacgtgtatc ctaaacgatt cctgtttgtt

12721 atttggaaag tcataccaaa aagaaatggt ggtgagaaga tttgtcctat atgtgcaaat

12781 caaaacgggg ttaatcttta cccggagtag agcataaacc taaaaatatg aaagagaccc

12841 attcaggaac aagaaaatac catcgtgatt tggattgaat ctcgatgaaa caatacatca

12901 atgaaaagtg aattcgataa gtttttctat tatataataa agaagaaaaa aaatgcgtct

12961 ttattgaaaa atcgaagaaa aagcccttaa tctatacatt gattcttttt ttttcgctat

13021 tttttttttg aaccgtatgc accaaaagat gcatgtacgg ttcctaaggg atacaatttt

13081 gccctactca accgacttta tcgagaaaga ttactttttt taggccaaga agttgatagc

13141 gagatctcga atcaacttat cggtcttatg gtatatctca gtatcgagga tgataccaaa

13201 gatctgtatt tgtttataaa ctctcctggc ggatgggtaa tacccggagt cgctgtttat

13261 gatactatgc aatttgtgcg accagaggtc catacaatat gcatgggatt agccgcgtca

13321 atgggatctt ttatcctggt tggaggagaa attaccaaac gtctagcatt ccctcacgct

13381 tggcgccaat ggggtttttt atttgagcga aaaagtaaaa ctatgccttc gccatatgaa

13441 tattaagtaa taatagcatg gcacttcgaa ttcgatatga aaaatttttg cattgttttt

13501 ttcaaaagat tcgattatgt atcgagagag tagtatgaga taaaagatat ttccgatttt

13561 ctcttatcta tcggaagtcc aattcagcgt tacaaacttg gttgttttca caccgaaagt

13621 ctcttaacca tttttaagtt ataaaaaaaa gagtgcaaaa aaaaaaccaa atttttccct

13681 ttttggttgg atcaaaaaga aactttggga ttgctgaatc aaagaaaaaa atccattttc

13741 aaatagaaaa gcaacggagc catcatagta tttttgaact cctccaaaag gaagggtggc

13801 aatttgacca tttaccctcc tggggctgat agattctatt tttatctgga aagtaagggt

13861 caatttgatt gtatagccgt atgcaatgca caaaagatga tgcccgtacg gttgttcaat

13921 tctatctttt tttcctatta ttcttgatct tccttttctg ttcctttcat cacatcagat

13981 agagaaccct tctatcatca gggtaatgat ccatcaaccc gcgagttctt tttatgaggc

14041 gcagacggga gaatttatcc tggaagcgga agaactgctt aaactacgcg aaaccctcac

14101 aagggtttat gtacaaagga cgggcaaacc tttatgggtt gtatctgaag acatggaaag

14161 agacgttttt atgtcagcaa cagaagccca agcttatgga attgttgatc ttgtagcagt

14221 tgaatgaaaa aaaatggatt ttgtgaaaat ccgtgatgtg atattttatc tccgagttta

14281 actattaaat aaaaaaattc ataatcatcc ggttaggatc aatctaaacc agcccattat

14341 gtatatattc aacatgccaa ccattaaaca acttattaga aatacaagac agcccattcg

14401 aaatgtcacg aaatcccccg ctcttggggg atgccctcag cgtcgaggaa catgtactag

14461 ggtgtatgtg cgactcgttt agatcatgag ctgatacaaa aaagcaagaa accgcttcca

14521 gtatgaatga ttagtccagt gaataggatc gaatggaaga aggaactcca tttactatct

14581 acttactatc taaaaataag ccactcgatc cctctattgt gtataaaatt tatggtttcc

14641 actggtgcaa atccaatcac ctgaatttaa gatgagaaac aattctccat tggtagcaaa

14701 tcgttatcca ttaagcggag gaaatcttat tgaaattcaa aaaaaaaaca taaaaatgaa

14761 gttctcgctc ggtcaagaac ggactacgag ggtcagctac ccagcgaaat ttcctaattc

14821 aataccgtta ctgtataggc gggtcttatt gtgaaagacc tgttactgga taattcatga

14881 gtagagccaa agagtgtgat gtgaactata caagttacca ataacattga tgaaatatta

14941 aataaatgaa gtaaaggctc cggtgtatag agaagacctc accgtttaag aagtaaccat

15001 agaaacgagg aaacccacta tttctttatc tatttaattt ctatttcttt taaaatacca

15061 aaaaaaataa aaaaatcgtg gttggggagg ttatagtagc caaagccatt ggaatttgta

15121 ttttatacat tggaaaaatc cgtttggtta ttaatagacc aggacgggta aaagaataac

15181 tgaaagaaac gaaatcaatt agttatttgt caaaatttta tttattcaat gaccagaatt

15241 aaacgcggat atatagcccg gaggcgtaga acaaaaattc gtttatttgc atcaagtttt

15301 cgggggtctc attcaagact tactcgaact attactcaac agaaaataag agctttggtt

15361 tcggctcatc gggataggga taagcaaaag agaaattttc gtcgtttgtg gatcactcgg

15421 ataaacgcag taattcgaga agggagagta ttctatagtt atagtaaatt aatacatgat

15481 ctatacaaga agcagttgct tcttaatcgt aaaatattgg cccaaatggc tatatcaaat

15541 aggaattgtc tttatatgat ttccaacgaa ataagaaaaa aagtagattg gaaagaatac

15601 accggaataa tttaaaagga gttccccgga gaatgaactc cgggaaggtg gggtcaaaat

15661 gactataaga aaagatgcta aagtagtcaa aatgaatgaa cacgttcaat aaaaaaaatc

15721 tttttttctg gttcgttttt tttttttttc ttacgacagc ataataaaat ctggattctc

15781 caatttctca aacaaaacac caatccgcgt ttgagttcgg aaagaaaaag aataagccta

15841 tttatttctg gttctaagac cagtcgttct ggtggtcgac tcgattcttt caaattgttt

15901 ctcattattg agaaaaggta acaaagataa aatacgagct tgttttatag caatagtgat

15961 taatcgttgt tgtttcaagg tcaatctatt cactcgtcta gataatattt ttccttgttc

16021 actaataaat cgactaatta aactcatgtt tctataatca attcgatccc ccgattgaat

16081 cgggggcaaa cgcctacgaa aagatcgctt ggatttcaga aaagatcgct tggatttatc

16141 catggttttg ttgtttagtt tatttcttaa ttgtcatttt tttttttact ccaatttttt

16201 atttaaatga aatatcttct atttatattt caatatgttc tatataatgg catttttccc

16261 ctttcaagga taagacatac tcggttcgat ttatttcttg atttccacat gaatcatatg

16321 tttgtaacaa tagggacaga attttcttaa ttctagtcga ttcggcatat tgtgccggtt

16381 tttttgagta atatatctgg aaatgcccgt tgataccttc ttaacgccgt tttggataca

16441 accggtacat tccaaaatca ccgttacccg cgcatctttc ctcttagcca tgaacctcct

16501 tttgattttt gatttgctca actcttctat tttgattcga aatgaagaaa aagaaaggaa

16561 ggaaaaaata gaagttacta acgtgaaatc caactctaaa agatcaaaat caataaagta

16621 ataataaatc aaagcaaagt catagtaaag aataataagt aagacagtga tttagaaact

16681 ctatttaaac ccgaaggaca tcagttaaag atcttgtatt cttgccctag accccgattt

16741 tcctactcta cctcccgtga ctctattatt tattattaat attgatttaa ttcgaattgg

16801 aacctgagtt tcgcagacgt tgaatccact tttcttcttt ctctttcgtc ccttagtcta

16861 aaaattagaa aaaaaaaggg aaaaaagaga aaaggagtgg ggggattagt tacagatttg

16921 aaattgtatc tctattcttc ttcattcttt ccgatgtcaa taactaaaat gaaaaaaagg

16981 gaaatgtcaa cgcatccggg aaaaaacgat taatctctat caatagacct gctaaagccc

17041 cgaaccatag cgtacttagt actggggcca cggagagata tgtttttaga tctcgcattg

17101 aaaaacctcc cttttttatt tattgtaata cagaaaaaga taatgcatat atgatcagat

17161 gcatatgtag ttaagggcca cttatagttg tacacgaaat cctcaattcc acttgaaatg

17221 taaaacgctc tataagactt cccccttctt attatatgtt aaatgagacc aaaaagacga

17281 aatgggtaga aaatttgtgt ttctcaatgc aggaaatagg ggtgtggaaa acaagacagg

17341 aattctctac aatgacactg taggacaatt gaagggatgt ggcgcagctt ggtagcgcgt

17401 ttgttttggg tacaaaatgt cacgggttca aatcctgtca tccctaccta ttactgcttc

17461 tttgagcagt aacgaggaat caattgagat cgattcaaat tgcacggaat cattcatttt

17521 tttgaaacta tagaatatat gcatataaaa gtgttaaaaa aagaatcctt tatttttatg

17581 tttacattct tttctattct tataagaaag cgctcttagt tcagttcggt agaacgtggg

17641 tctccaaaac ccgatgtcgt aggttcaaat cctacagagc gtgatttttt cttcatgagt

17701 cgaattagac tgaaatagat tcagcagtca cttgcacaac aattccaatt tgacctcctg

17761 tggattaaat aaaggaagag gccaatcaaa aaaagaaatg ttaattaatc aaaggtccaa

17821 ctgatcacca cgcctgtatt gtaaatatgc agttacgaat aacccggcca aagtaatagg

17881 aattagacct aacacgattc caaatagaaa aacttcaatc atttcaattt tttgaaaagg

17941 agaaaaaaga ggtaatatct atacctaaat attaatccga attgatgcga atctcaatga

18001 ccaaaaattg acaattaacg tggtaactaa ctctagaata gacggaattt caaagaagga

18061 tttactttct gaattgtttc tttcaaatag aaattttaaa taagtcgtat cttgctcaga

18121 ccaataaaga gagctgaagt tatagttaaa gccactagta gaaaaccgaa ataactagtt

18181 atagtaagca tgaaggggct aaatgaaata tgttattttt atacatatgt ttctaaagca

18241 tttacctaag tttccatttt tttcaaaatg ggaagatgtc caaaaagacc cattgaaaga

18301 ataagttcaa ttgaaagttt ttgattcatc tatcattata gatagcacga acggggagaa

18361 agtgacgggc atataaaatg aaactgctta atcatttcta acatctagcc atctcgcgat

18421 tcttatcaaa cgagtcgttg agcataaact aataatacga actggagcgt gtaacttcat

18481 tcacaaatga aactctgttt gaattcttat ttgtgaatga aatttttagg gaatactctt

18541 ttttgttcga tattttcaaa taaagtatca tccttgtagc tagatcgaga tttggattga

18601 gatccaatcc acaacaattc attacaacta ttgattccaa ttattttatt ctttcttcgc

18661 tttctttcat cgaataggat atactactcg tatttggtac tggacgatta accaattcct

18721 gaaaatgaaa aataacgggg ggttcaaaca aaaaactgtc tatacaacca tgaaaagaaa

18781 attgaaggaa atttctatat ctctcatcta cttaaattga attgtggaaa taggatactc

18841 tttttcattg taagaatttt ctattaaata cagcaccatt ttacatcaac aggtaaagga

18901 aaagaataaa taaattcttt agcacttcct ttcgatactg attcaaattg cattgctgtg

18961 tcagaagaag gatagctata ctgattcggt atactctaaa gatgcccttg gtacaatatt

19021 gacgatccta caaagatcca atttcagtga ttgcctttta ctgatctcat cttttacgga

19081 atcgatcccc tttgactgta caagaatatg tggagctgaa catgtctgga agcacaggag

19141 aacgttcttt tgctgatatt attaccagta ttcgatattg ggtcattcat agcattacta

19201 taccttccct attcattgcg ggttggttat tcgtcagtac cggtttagct tacgatgtgt

19261 ttggaagccc tcggccaaat gagtatttca cagagagccg acaaggaatt ccattaataa

19321 ctggccgttt tgatcctttg gaacaactcg atgaatttag tagatcgttt taggaggccc

19381 taatcaaatg actatagatc gaacctatcc aatttttaca gtacgctggt tggctgttca

19441 cggcctagct gtacctaccg tctttttttt ggggtcaata tcagcaatgc agttcatcca

19501 acgataaacc taattccgaa ttatagagct atgacacaat caaacccgaa tgaacaaaat

19561 gttgaattga atcgtaccag tctctactgg gggttattac tcatttttgt acttgctgtt

19621 ttattttcca attatttctt caattaggaa aatgaaagaa aataaataag aattctaggc

19681 attctcttag cccattcgga aggatctcat ctcataatta tccacgactg tttatgtctc

19741 tagcatgacc acttgatgaa agaaattgga gggacacgga gtaaatggcc gatactactg

19801 gaaggattcc tctttggata ataggtactg cagctggtat tcttgtgatc ggtttaatag

19861 gtattttctt ttatggttca tattccggat taggttcatc cctgtagtaa tcggatgaat

19921 ggagttgtag acatgaaagc gtaggaactc aacgggattc ccttctttag tttgtctgat

19981 tcgaggggaa aggccccgtt gggttcttaa taagcagagt cttttttttc atctaactga

20041 caaagtggat ttctttttct gctggttcaa aaaactccat tctctttttt ctgatgatgt

20101 ttttgaaaaa tgaacttcat tgattgattc agaacacctc ttcctcgatc ttgatagtat

20161 tcatgggtac ttagaacaaa ggggggaatc acccaatttc ttggattata tatatatata

20221 tttctgtaga ttagatatcg tacaaacgat ttgtatactc ttaccctatt ttttttgagt

20281 atctcagaaa aatggaaagt tcgttgaata agttttcttt tttttgtcca ctactttatt

20341 ttaattttaa aattttatat ttatttgcat ttcgattttc ttgtacgtaa gaagtcgaaa

20401 aaaagttctg atacacctgg aaacccgaaa ccaagactag gaatttttga cgatttgacg

20461 aacaggatca aaaatcatta ctctttcgac acaagaaagg gaacttttga tctacacccc

20521 tttcttgtgt cgaagactag ggagacaaat aatgtctcct agaattcttt gtcccacgca

20581 tttttagttc gttctattac ccgcctactt atatctatta tctatcccca ttttattcta

20641 tttcaaatag aataaaatgg atctgtttta tgacattgaa attcggcaac agtaacatag

20701 tcctatcaat tcaatttggc taaaaaaaac aaagaagggg gtggtaaagt cataaagtaa

20761 aataaaatag ttttcttcaa acaaaaatat acctattatg actaggaatg cggtacaagg

20821 gattcgattc cccgaagctc gtagcaaaag agcaggtaaa taaaaggttt tttagaattg

20881 atttttttta atcatacata attgaaaaca acaattttgt tctttttacg aacctgatgt

20941 caataaatcc gcgagtctag aaattcattt cggccaattg aaccttctcg aactgtttct

21001 ttttaagaac caaaaagatt tgggccaaaa taacagacgc caagaagacc aaaagacctt

21061 ggacacgtaa tggatcttga agtactattt ctgcatctcc ctgaccaaat ccacccacat

21121 taggattact cgttaatggt tgatccaatt tgatggattc accctctgaa acaagaactt

21181 ctggtcccgg agggataata tcaaccactt cacgtccatc cgatggatct gttatggtta

21241 tttcataccc ccctttttct tttcgtatga ttttacttac tatgcccgcc cctgtagcat

21301 tataaactgt attgttactc ttgcttccgt cgggataaat ctgtcccctt cccctattcc

21361 cccctacgta tataggatat tttaagaagt gaacatcttt cttagtagcg gggtcggggg

21421 aaagaatagg aaaggtgatt tcactatatt tctgaccggg gacaggccct atcacaagaa

21481 tattttgttt attagggcga tagctctgaa aagacaaatt gcctatcttt tctttcatct

21541 cgggagaaat acgatcggga ggagctaatt caaacccctc cggtaaaata agaacagccc

21601 ccgcattcaa accccctttc ttaccattag caagaacttg tttcacttgc ttatcataag

21661 gaattcgaac aactgcttca aatacagtat cagggagtac cgcctgtgga acctcaatat

21721 ccacgggctt attagctaaa tggcagttgg cacatacaat acgcccagtc gcttctcgtg

21781 gattttcata accctgctgc gcaaaaatgg gatatgcact tgaaatggat gtccgagtta

21841 tgatatatat catgagtgat acggaaatag atcgagtaat atgttccttt atccaagaaa

21901 aagtctttct agtttgcatg gtctaatcat tgatccgaaa agttctacaa taaatttggc

21961 aggtctctag tatagttccc tgtccacgat tctgctattt attggaatca ttttactaga

22021 atactatagt ataagtatac tatgaaaata gtatgaaaat cgcctgtttt taatacttct

22081 gtagaactac aaagtaagaa acattctaat tggattaata tcggcggatc ttttatcaat

22141 cattcattga atgataaata actacaagtg acggagatac acgatttaaa taatgaaaaa

22201 tccaatattt aaaaatagta tcgagaataa ctggaaaagt ggaaacaaga ccagatataa

22261 tttgatcatt atgaccaaat ccaaaatctt tgtagacaga accaatcatt agttcccaac

22321 catggggtga atgaaatccg atacataaat cggttaataa aagaatcaaa aaagctttga

22381 ctgtatcact taagttatat aggaattctt gagtccaaga gttaagaata acaagttctt

22441 gattacctaa aatagaataa ccggttagaa taacaaaaca gattagattt gttgagaagt

22501 gcaaaatcgt atggatacga tcctcattgt gtatcttgat taattggatc gtttctttgt

22561 ggatttctat aggaagcttt tgtagatgtg tctccgagta ttccttgatc atttcttcca

22621 agaagaggat ttcctctaat tctatgaact tttctagaag acttttttct tgcatatcat

22681 tcaaaaaatt ttcggattga cccgtattcg accaactagt aacccaagat tccatacttt

22741 tagtaaatga gagagaaatc caccagggca gaaatactat agatgcaaga tacaaaagag

22801 gagtgaatgc tttctttttt gccattttta acctgtgaat ttttaattaa cccacttcat

22861 ttgtcgactc tatgtgatcc aatatttctt tcaaaccaaa catgagttct agacaaggta

22921 tcaaacctat gaatctattt tctttgtgat tttgtttgaa tctagaaaga atacagaaat

22981 agactaagac tccacttgga attcgactga agaaataata caaaattgtg tttccagata

23041 tctcaattca tcaaattcca aacaaaacac gtgtctcctt tcgatcaatg aacaaaagaa

23101 gtcctttaag gaatgaatat tgttgagatt tggagacgta aagaactctt tttctatcaa

23161 aatatccaat atttcaaaaa aattccaagg agtttccata gtcaagaata gaataattga

23221 gagaaagata ttgtgatgat caaaaaatcg attgacaaaa agaaaagaat ttacaagata

23281 tgatttatct gcatctaaag tatcacttag ttatagaaaa aaacaagatt cttgtatttt

23341 tccctcctgc ggagaaagca ttcgttcttc agcccaatcc ctttctcaaa agacttcaat

23401 tggtacgcgc aagaaatagg ccaattccgc ggctttttgt tcaatttctc gtggagtcaa

23461 attctcatca gtacgggtca acggaatagc cccccggcct cggatgtcca tataaaggat

23521 acggcgagca taaataccct ctttaacttc tattctaacg gattgaatat cttttatacg

23581 gaatcggagg aatatgcgac gattttttcc aggaaatccc caccgaaaaa tacacaccat

23641 tccttccttt ctatcgaatt gatcataacc actacctaca ttccaggaaa ttgtgcacca

23701 caagtaggaa ctaataaaga gacctgcgat cccgtagaaa gacatcacga tcccttgtgg

23761 aaaaaaaagg atttgctgag acggaacaaa agatatcaaa tttctaccaa gataactgga

23821 agttccaacc aataagaatc ctaatgaacc taaaaaaacg acaagcgccc agcaaaaatt

23881 acttagtttt cgagaccccg ttataagttc tatccatata tgttctgatc gccaactcat

23941 acttgatccg attgcatttt attggaattg agagaatacc ccaaatggtt ttgactttac

24001 ttttttgttt ccgaatgaac tttcatcaac tagcactagt ttaatgtgaa ctagcactag

24061 tttgatggga acctttagga gtaattcctc aaattggtta gatctaaaat aataacacac

24121 ccttcctatg atcccaatat acagatatac atatagatcc gccgacttta cattttgggt

24181 atccagcagt cctgatctat ttcaaaccag ctggaattca gttacccata gatcattttc

24241 tgcaggcata agagcttttt cctttatgtt cgtcagaaat cacatgttct accttatttc

24301 ccgaaataaa tatatgggtt atgctacaag tctaagtttc aaaaaaacgt atgagattgg

24361 gtcccctcag atctaaacaa tcttgttttt ttgaacatga agaaataaag aagccattgc

24421 aattgccgga aatacaaggc ctactaaagg cacaaaaata gagggaaatt ggaaagttgt

24481 cataaaatgg gtacctcgat ttactatttg tacctgttct tatttttttt aatatcatat

24541 ttattattta tactaattat aattaataat tgtaattagt atgaattcgt agttattatg

24601 aattcattca attcgaaaat tcttattaca gattacagat ataagtatct aattgagtat

24661 atagaataag tccaaggaat gtagaactca aaaaatggac ttattcgtct atctacatga

24721 aagatacata tgataatcca tttgattatt tttcttcatt tctttgtgtt ttgttattgt

24781 cttcgaattt aatattaata ggagtttctt acaaattatt gaaagattca ttagaatgcg

24841 gcacaaccgg gatttttagt gaaaatagga ttttcggggg atgaagaaag atacaaaacc

24901 atatatctat ttttttctat atttgaattt gattctatac ctaagacaaa atgcgtttta

24961 tttgcctaat ttcacgaaag gaagggaaga actcgtgttt ttatcttgtt gatagagact

25021 tcttaattag taatcgggaa tccaggtaaa aaaaagagtt atccgccact ttttattctt

25081 tttacaatta gatcttaggt caccaaagaa aacttcattc ttagttactt tgattccgat

25141 aagcaaacta cttgtttgct acaaataaaa taattgaacc tagcgcattg aattggaatt

25201 caagggaaag aaggcgtgga gcttaaataa ctcactcaga acacttttta aaagattacg

25261 tggtacgatt aggtcaaata agcccttctg gaataaatat tcagaagctt gtgaaccttc

25321 gggtatcgtt ttattcaatg tttgctcaat tactctttta cccgcaaatg caatgtagga

25381 atttggttct gcaataataa tatctcccaa cataccaaaa ctagctgtta ccccgccggt

25441 agtaggagat gtaaggattg atacatacaa taacttttta tttgattggt aatcaaataa

25501 ggcagacgag attttagcca tttgcatcaa gctcaaactt ccttcttgca tgcgcgcccc

25561 ccccgaagcg cacactataa taagaggtat aaattgattg gtagcgtact caatcaaacg

25621 ggttattttc tccccgacta cggatcccat actacccccc ataaatttaa aatccataac

25681 cccaattgct acgggaatac catttagttg acctacgcct gtttgaacag cctcggttaa

25741 tcctatgttt ctttgataaa aatcaatacg atctttataa gtctcctcct ccgaatgaaa

25801 tccaatggga tccccggaga ccatgtcttc atccatagga tcccaagtac cggggtcgat

25861 caaaacttcg attctttctg aactactcat tttcaaatga tatccacatt gttcacaaat

25921 attaatttgt gatttcaaaa gtttcttata atttaatcca taacaatttt cgcattgaac

25981 ccacaactgc ttgtattttt gagtttcatc gagatcgcta gctcttagag ttaaatcact

26041 actcttcgcg cgggttcgta tactgggttc actacgagtt ttacgtttct gaaaaacgca

26101 cctagaaatg taactgtcac tgctatcact tacagtgggt gtatcaatac agatttgaga

26161 ctgaagataa ctgtcaatgc aactagtaat atgattattc caactagatt gagtatcgta

26221 catgtaacga gtgtataagg aatcttcatt cgtagatcca ttattcatat aattcgaatt

26281 gcgataactg gaaaaagaac tttccagttc actccgaaaa gaatgatcgt tgtcaatctc

26341 aaaaatctga ttttcaatat caaaatagat agaataactg tctccattac tatccctaac

26401 taaaaaagta tcatccgaga tgaaattccg aatgtctttg gcaccaaata aaagatcgac

26461 attactgtaa ctagaattgt cacgaccgct ccaactatga atgtttttag ccctaccttt

26521 tctattcgga tcttcgcctt cactagtatt ttccataggc ccgagattgt ccgttaattt

26581 ctttatccca tacctgcgtt ctaactcctt cttaaagacc atcgaattaa accaacacct

26641 ttccatagag ttttcttgcc ccctatttgt ataaaaaata caatagatga atagttattc

26701 gatccacaat tctttatttt tatttgaata tcttatttcc tatcaaacta aacatcgaaa

26761 ttcaatcact actaaacagg aagtgtgaaa aataattctc tgagaatcca ggggtaagac

26821 ttcactatat atgaataggc tggaataccc ttttattcga aatttcatat aatgataaaa

26881 agtggttttt actctgtctt cgcatcgaac aaaaaaagga aatatttgtt ctaggagaga

26941 acaaatattt ttcgcaaaat ctcgtctaat aactaactaa taacaagtaa gaaattaagg

27001 ttccattcca acacggaacg aaaaagacaa tatacaggat gggtcgaaag atttgtgata

27061 cttcgcttgg ttcggggaaa ctacaggata tataaaagaa tataccaatc ctaaggatcc

27121 atgggtttaa ttgtggatcc aagacaacaa tagaaaaatt tgagtcttcg atttctattt

27181 caaatctttc tatatctaga gatacatgta tttatccaaa atacatgcat atagaatctt

27241 tatattcggc tcaatccttt tagtaaaaga ttgggccgag ttaaattgca attcaattaa

27301 gagaacggag agtaattagt ttttcttttc ctcatccaaa gtatccactg gtttaaattc

27361 aaatttgatc tctttccata cctcacaagc ggcagctagt tcaggactcc acttgcaagc

27421 ctcgcggata attgcattac cctcagcagc aagatcacgt ccttcattac gagcttgtac

27481 acatgcttct agagcgactc ggttagctac agcacctggc gcattacccc aagggtgtcc

27541 taaagttcct ccgccgaact gtagtacgga atcatcccca aagatctcgg tcagagcagg

27601 catatgccaa acgtgaatac ccccggaagc cacgggaata acacccggta gagagaccca

27661 atcttgagtg aaataaatac cgcgacttcg atctttttca atataatcat cacgcagtaa

27721 atcaacaaag cctaaagtaa tttctctttc tccttcaagt ttacctacta cggtaccgga

27781 gtgaatatga tctccaccgg acagacgtag cgctttagct agtacacgga agtgcatacc

27841 atgattcttc tgtctatcaa taactgcatg cattgcacgg tgaatgtgaa gaagtaagcc

27901 attatctcgg caataatgag ataagctagt atttgcagtg aatcctcctg ttaagtagtc

27961 atgcattacg ataggaactc ccaattctct agcaaataca gcccttttga tcatttcttc

28021 gcatgtaccc gcagtagcat tcaaataatg ccctttgatt tcacctgttt cagcctgtgc

28081 tttataaatt gcttcggcac aaaataagaa gcgatctctc caacgcataa atggctggga

28141 gttcacgttt tcatcatctt tggtaaaatc aagtccaccg cgaagacatt cataaactgc

28201 tctaccatag tttttagcag ataaccccaa tttcggttta atagtacatc ccaacagagg

28261 acgaccatac ttgttcaatt tatctctctc aacttggatc ccatgaggtg ggccttggaa

28321 agttttaata taagcaggag ggactcgcag atcttccaga cgtagagcac gtagggcttt

28381 gaatccaaat acatttccta caatggaagt aaacatgtta gtaacagaac cttcttcaaa

28441 aaggtctaaa gggtaagcta cataacagat atattgatct ttttctccaa gaacgggctc

28501 gatgtggtag catcgccctt tgtaacgatc aaggctggta agtccatcgg tccacacagt

28561 tgtccatgta ccagtcgaag attcggcagc taccgcggcc cctgcttctt cgggcggaac

28621 tccaggttga ggagttactc ggaatgctgc caagatatca gtatctttgg tttcgtattc

28681 aggggtataa taagtcaatt tgtactcttt aacacccgct ttgaatccaa cacttgcttt

28741 agtctctgtt tgtggtgaca tacatccctc cctacaactc atgaattaag aattctgaca

28801 acaacaaggt ctactcgaca cgaatgaatt gggcgttaat gaaacttttc acaggaatct

28861 ttcacaaact tcccaactaa tactaatatt atcaactaat cagaatgttt gattattaga

28921 ccatggtatt tgatttacca aacacatcat tattgtatac tctttcatat atatagcgca

28981 accccatttt tctttttgcc ccttttttgg aatcgaaata cctaacctaa ctaataataa

29041 attccccctt gacagtgtat atgttgtata tgtaaatcct agatgtgaaa ataggcgcaa

29101 ttcatctatg aaatgatgat ggaagggtat aaaaaaacga aaagaaagga ataggcggct

29161 atagtatagt cctaaatgct gaaataaaaa tacaaaatga agaagagcca ataacataga

29221 aataatgaat cgtaatgtaa atagagttcg ggttcgaatt ccatagaata ttttttctaa

29281 tatagatgag attgtctatc attatagaca aatgaaaaac tttctcaaga tccttattca

29341 tcattcatcc acttgaaatt ttaaaaaaag cttggttgac ctttcaaatt cactcattga

29401 aattgaacaa gtaaacaatt gaattggatt cgattggatg gcactaacga aatcaaatgc

29461 taactctcat ttgttattga attaaccgat cgacgtgtta gcggacattt ctttttttga

29521 attcgataat tttcacaaaa aaaatttcga catatatttt tattattatt atgataatta

29581 atcctactac ttctggttct ggggtttcca cgcttgaaaa aaaaaaccag gggcgtatca

29641 tccaaataat cggtccggta ctagatgtag cctttccgcc gggcaagatg cccaatattt

29701 ataacgccct ggtagttaaa ggccaagata ctgctggtca accaattaat gtgacttgtg

29761 aggtacagca attattagga aataatcggg ttagggctgt agctatgagt gctacagatg

29821 gtctgatgag aggaatggaa gtgattgata cgggatctcc tctaagcgtt cccgtcggtg

29881 gagcgactct gggacgaatt ttcaacgtgc ttggagagcc tgttgataat ttaggtcctg

29941 tagatactcg taccacattt cctattcatc gatctgcgcc cgcctttata cagttagata

30001 caaaattatc tatttttgaa acgggaatta aagtagtcga tcttttagca ccttatcgcc

30061 gtgggggaaa aatcggacta tttgggggag ctggggttgg taaaacggta ctcattatgg

30121 aattgattaa caatattgcc aaagcccatg ggggcgtatc cgtatttggc ggagtgggtg

30181 aacgtactcg tgaaggaaat gatctttaca tggaaatgaa agaatctgga gtgattaatg

30241 aagaaaatat tgcagaatca aaagtggctc tagtttacgg ccagatgaat gagccgccgg

30301 gagctcgcat gagggttggt ttgactgccc taacgatggc ggaatatttc cgagatgtta

30361 atgaacaaga cgtacttcta tttatcgaca atatcttccg tttcgtccaa gcaggatccg

30421 aagtatcggc cttattgggt agaatgcctt ccgctgtcgg ttatcaaccc accctgagta

30481 ccgaaatggg ctctttacaa gaaagaatta cttctaccaa agaagggtcc ataacttcta

30541 ttcaagcagt ttatgtaccc gcagacgatt tgaccgatcc tgcccctgct acgacatttg

30601 cacatttaga tgctactacc gtactatcaa gaggattggc tgccaaaggg atctatccag

30661 cagtagatcc tttagattca acttcaacca tgcttcaacc tcggatcgtt ggcgaggaac

30721 attatgaaat tgcgcaaaga gttaagcaaa ctttacaacg ttataaagag cttcaggaca

30781 ttatagctat ccttgggttg gacgaattat ccgaagagga tcgtttaacc gtagcaagag

30841 cgcgcaaaat tgagcgtttc ttatcacaac ccttttttgt agccgaagta tttaccggtt

30901 ctccggggaa atatgttggt ctagcagaaa ccattagagg gtttcaattg atcctttcgg

30961 gagaattaga cggtcttccc gaacaggcct tttatttagt cggtaatatc gatgaagctg

31021 ccgcgaaggc tatgaactta gaaatggaga gcaatttgca gaaatgactt taaatctttg

31081 tgtactgacc cctaatcgaa ttgtttggga ttcagaagtg aaagaaatca ttttatctac

31141 aaatagtggt caaattggcg tattagcaaa tcatgctcct gttgctacag ctgtagatat

31201 agggatttta agaatacgcc ttaaggacca atggttaacg atggctctaa tgggcggttt

31261 tgctagaata ggcaataatg aaatcactgt tttagtaaat gatgcggaaa agggtagtga

31321 tattgatcca caagaagctc agcaaactct tgaaatagcg gaagctaatt tgagaaaagc

31381 tgaaggaaag agacaaataa ttgaggcaaa tctagctctc cggcgagcta ggacaagagt

31441 agaggctgtc aatgttattt cataactagt tggtgcgttc aaataatcaa aagaagttct

31501 gtttctaaat cctattttga ttggattctg tcgagtgaat ccaatcaagc agaatcccat

31561 tttgatacaa cgcaattcaa aaataaaatt gaactagatt caataaaaaa aaatctctaa

31621 aaatagatag aagagggtgg ggcaaaaaac ttattagatg tcggagtcaa tggtatctaa

31681 taagttctac ctactattgg atttgaacca atgactcccg ccgtatgaaa gcaatactct

31741 aaccactgag ttaagtaggt catttatcat cccaaagaga accaaacgga acccatccca

31801 tcgatggatt ataaatagca tattgcttat aagcaataat aatctaagca ataccactca

31861 accactcaca aatttaggat gttggatcat agaatattca tcttgacaac aaattatata

31921 catgataaaa tatgcatcac aagcactaag ggctatagct cagttggtag agcacctcgt

31981 ttacacgcgc gccaatgttt ttcaggggag tccatcatac aatccaaaaa atgtatctta

32041 ttgagaaatc gatgtcttac tccataactt tacgagaaca atagcctgac aaagggttcg

32101 gttcaatttg agtgcccgtt taggtaccaa acagacccca tgattgattt gagatattga

32161 taaggtgaat accagtacat tcaatgctag gcataatgag tacaaggccc tcaaaaaatc

32221 tcttttcgtc ctatgaactt gaaggtgtat gaagtttcat attggatttt ttaagccgaa

32281 gaatagagac ttgatttaac ttaaaacgat ctaggccaga ggcagaccta cgtcaagata

32341 accccaccct tgaaacactt tggtagtgct tctgaatcag aatcccaaat aatgaatcag

32401 agcacgtgga gccatcccct tatcttattt ttctgtcaag aaaaaatatg gcggattggc

32461 tgatatttct atcagttaat gaaagagccc aatgcaaaaa aaatgcatgt tgggtctttg

32521 aaacagttca gatcattttg ataataataa gtttgatctg ttttaccgag aaggtctacg

32581 gttcgagtcc gtatagccct agagccctat aaaatgaata aaatccaaat ttgaaataca

32641 aaattgtata attttcattt cttcattctt gtttgttgct tgtaactgac cggttggttc

32701 atccaaaccc aatttgtcct agaaactcac tcacaggaat cgtttaaagc cgaacagaaa

32761 actgaaagaa aaacgtattt caagagatcg aatcgatcct tttccaaccg tgccaatcgt

32821 ggataaacaa accaaccctt cgtcattaat cttcgtaggg aaattctata tgaattttcc

32881 tgtccataat caaggggtta agctagccag actccccttt ttggtatatc taaaaactag

32941 acctagcgaa gcacaccaaa acaaaaaggg gatggtcttc tttttctcta ttcaatcaag

33001 agaaaacccc acccttattt tcataaacgg aatataccaa cactcaaatt aagatattcg

33061 aaatcctgag atggaaatac ctacccattt gcttccattt gaatactcgt tctattctaa

33121 atcactaaga aaaaaaaacg acaaaaagac ctcccgattc tattcctaaa aaataaaaat

33181 ctataaatcg aatttttata aaaaaaaatt caaataatca aaagaagaat tcgattttat

33241 tttgaagtcg ctttctttag caagaatcct aggcgaaaac aagaaaattt gtctaagcgt

33301 aacatataga gttatactaa ctaaagcaat taaataaaat ggaaataaaa aattaaatag

33361 gctggaaata ggatccctgt caagtcagtc gataaatctt gaaatgagat atgccccgca

33421 ataatcaaag gagactagaa gatttggtca aaacaagaat tcattttatt tggaccaacc

33481 agttatggat gactttcaaa attcaattcg aatcaaccaa tccggcataa tttccacgtt

33541 cataggagtg cgtctatgtt tttgctttac gaatatgata ttttttgggc atttctaata

33601 atatcaagtc ttattcctat tttggcattt tttatttccg ggattttagc cccgattagg

33661 aaagggccgg agaaactttc tagttatgaa tcgggtatag aaccgatggg cgatgcttgg

33721 ttacaatttc gaatccgtta ttatatgttt gctctagttt ttgttgtttt tgatgttgaa

33781 acggtttttc tttatccatg ggcaatgagt ttcgatgtat tgggcgtatc cgtatttata

33841 gaagctttaa ttttcgtgct tatcttaatt gttggtttag tttatgcatg gcgaaagggg

33901 gcattggaat ggtcttagct cctgaatatt cagacaataa aaagaaaggg gaaaaaaaga

33961 ttgaaaaagt tatgaatccc atcgagtttc ctttacttaa tcgaacagcc gaaatttcag

34021 ttatttcaac tacattaaat gatctttcaa attggtcaag actctctagt ttatggccgc

34081 ttctctatgg taccagttgt tgttttattg aatttgcttc actaatagga tcacggtttg

34141 actttgatcg ttatggacta gtaccaagat cgagtcctag acaagcggat ctaattttaa

34201 cagccggaac agtaacaatg aaaatggccc cctccttagt gagattatat gagcaaatgc

34261 ccgacccaaa atatgttatt gctatgggag catgtacaat tacaggaggg atgttcagta

34321 ccgattctta tagtactgtt cggggagtcg ataaactaat tcctgtggat gtctatttgc

34381 caggttgtcc ccctaaaccg gaagcggtta tagatgctat aacaaaactt cgtaagaaaa

34441 tatctcgaga aatctatgaa aatagaatta ggtctcaaca agcaaatcgg tgttttacga

34501 ctaatcacaa gtttcgtgtt ggacgcagta tgaatactgg aaattatgat caaagattcc

34561 tctatcaacc gccatctact tcagagatcc ctactgaaaa ctttttcgaa tataaaagtt

34621 cagtatcttc tcatgaattg gtgaattagg ccggactcct ttgtacagaa taacaagcaa

34681 cgggtcaatc ttcatcaatt tgatcgagaa tgtgaaatat tctaaataaa aatgtgggag

34741 ataaaaaaga tgcagggtcg tttgtctgct tggttagtca agcatgggat aattcataga

34801 tctttgggct ttgattatca aggaatagag actttacaaa taaagcccga ggattggcac

34861 tccattgctg tcattttata tgtatatggt tacaattatc tacgctccca atgtgcctat

34921 gatgtagcac ccggcggact gttagctagt gtgtatcatc ttaccagaat agagtatggt

34981 gtggatcaac cagaagaggt atgcataaaa gtgtttacct caaggaggaa tcctagaatt

35041 ccgtccgttt tctgggtttg gaaaagcgtg gattttcaag aacgagaatc ttatgatatg

35101 ttgggaatct cttatgataa tcatccgcgc cttaaacgta tcttaatgcc tgaaagttgg

35161 ataggatggc ccttacgtaa ggattatatt gcccccaatt tttatgaaat acaagatgct

35221 cactgaatga gaagaaagtc atttccactt ataaaatttc agagattcaa ggattggctt

35281 tctgttctag attaacagaa taattgaggt ctcttttgta tttgtattag ggaattgtgg

35341 ataggctaac aaaaaaggtt gaattaggga ttcgttggga ttcttagatt tatttgcaag

35401 atatttattt cgttcgtatg agccgaacca ataggatgaa tcaaacgagt tctgcgtcat

35461 gaaccttgta ctgcgcctat tgcttagacc ggttgtagaa agtcatgtcg agacgaatta

35521 ggaaatcgaa tagagatgaa aatactaatc atgaaatgaa agggtatcca attcgatttc

35581 ttttttttgt atctgagccg aatcttgtct atttcaactt tgactttttg ttctttcaac

35641 caactaattg aacctttcaa ataggaaata tgtataaagt attaatattc tttttgcacg

35701 aaagaaaaaa aagagaaaat cgaatttcat tttatttatt taagaattta agaaactcta

35761 cccatctata aaatagatgg ggtatatctc gctaagatag ataaaagtat gtattatgat

35821 ccagattcaa aatcaatatg tatctcgctt catatcaatt aatttataaa agaaagacct

35881 catccaattt aaccatgtgc caggaaccag atttgaactg gtgacacgag gattttcagt

35941 cctctgctct accagctgag ctatcccgac cattcccaat gtagcatccc atcttcattt

36001 tattagatga ctggagtctc tgtcaattaa agggacaggg gggattacaa agttttctcc

36061 aagtcctgca gctattcatt gtgaattatg caaataggga ttccttgctt aaagtggatg

36121 tgtattctat atcacatgtg ataagagaaa gttatttacg cccaaatacg aaagtcaaag

36181 aatcagaaag ataaaggaat ttggaaccgc taacgaaaag gggggacggg ataactattt

36241 tattttagga gtcaaatagg cttttttggg gatagaggga cttgaaccct cacgattttt

36301 aaagtcgacg gattttcctc ttactataaa tttcattgtt gccagtattg acatgtagaa

36361 tgggactcta tctttattct cgtccgatta atcagttctt taaaagatct atcggactat

36421 ggagtgaatg atttgatgaa tgaatattcg attttttctt caattataga ataaattcac

36481 accaattctt ctatttttca tattaaaaat acggattcgg gccatcatta atcattttat

36541 atagtattcg atagatacgt ttatcctttc tgaagtttcc gtagaaagga ttcctctacc

36601 aacgcagtca actccatttg ttagaacagc ttccattgag tctctgcacc tatcctttag

36661 ttcgtttttt gaacctttgt tttgaaaaaa caggatttgg atcaggatcg cccattttta

36721 ttaattccgg ggtttctctg aatttgaaag ttatcactta gtaggtttcc cacactaagg

36781 ctcaatttaa ttataattaa gtccgtagcg tctaccgatt tcgccatatc ccctcttact

36841 tttcttttgt tttgagctta ggatcttatt atgatatcat tccctttttt ctttcattta

36901 taccggaacc cttgaattta ttagataggg attactatct cgaaaaaagt attttctttc

36961 ttacctatag gaagcccgta ttggatccaa tccaattgga ttttatcatt tctgtatcgg

37021 caattcaata tagattgata tataccccat atatatcttt ctattcctcc cattttacca

37081 ttcaaggggc gatacttgaa gggtcgattt ttccctttac gaataaaagc cgaggaatgt

37141 ctgattagtt ataactaatc aatcgaattc aaaagaaata tagaattgga aatatatagg

37201 atagaaaata tagaagtgta acaaaaagaa tttcaaatgt catgtgaatt caaaataata

37261 aaaaaaaatt ggactagact ctctaaaaat atttaagatt tactatcgaa tagaataata

37321 ttcgaatttc gaattgtcag aaagaaatca aaattctata ttcgctatat aaatcgttat

37381 cttctagaat atccaatatt tattgggaat tatagattct attcttttct atatttctat

37441 agacactata cttatactat agtgtattga attcctatgc atagaaaatt tctattatgc

37501 gggcccgctt agctcagagg ttagagcatc gcatttgtaa tgcgatggtc atcggttcga

37561 ttccgatagc cggctttttc ctatttttca tttttttatt caagttcaag aaaaacggac

37621 aatcttcctt tgtttgaaat caaagaatat tgaaaaagtg tcttcccctc tttccaaaaa

37681 ccatgaattt cttaagttat aggttaagtt atagggaact cctaactatg tcagtaaaag

37741 gatcttatat atgtaatatg taataataga ataatatata ataatagtat atatatatat

37801 aataatagaa agtttgaata tttatccaat ttctctcatt cttctttata gtacaagtac

37861 actacttcag caaacttcgc ttcatttagt tcagttttag tttaggttta ttctgtaaaa

37921 tccaattttc aaaaaaagaa tgaaatgaat tctaaataag aataaggagt ctttatgtcg

37981 cgttaccgag gacctcgttt caaaaaaata cgccgtcttg gggctttacc aggactaacg

38041 aataaaaggc ctaaagccgg aagtgatctt agaaaccaat cgcgttccgg gaaaaaatct

38101 caatatcgta ttcgcctaga agaaaaacaa aaattgcggt ttcattatgg tcttacagaa

38161 cgacaattac ttaaatacgt tcgtattgcc ggaaaagcca gggggtcaac gggtcaagtt

38221 ttactacaat tacttgaaat gcgtttggat aacatccttt ttcgattggg tatggcttcg

38281 actattcccg cagcccgcca attagttaac catagacata ttttagtgaa tgggcgtata

38341 gtagatatac caagttatcg ctgcaaaccc cgagatatta ttacggggaa ggatgaacaa

38401 aaatccagaa ctctgattca aaattctctc aactcttccc ctcaggcgga agtgccaaac

38461 catttgaccc ttcacccatt ccaatataaa ggattagtca atcaaataat agatagtaag

38521 tgggtcggtt tgaaaataaa tgaattgcta gtcgtagaat attattctcg tcagacttaa

38581 acctaaactg aaataaaata caagggctcg ggcaattttc ttccccttca tcaacaaata

38641 aaattaacct taagtaagaa aaatgtgggg ttgaccccga ttttatccca tttatgtatc

38701 acagcccgcg gggatatttt tctatttttt tttcagtatt tttcctagtc acggcaattc

38761 ggaatagaga atccataggt ctaactggtg gaataagggt ctccattgct agttgatccg

38821 gtgtttttaa taaattaaaa aaacgggtct attaagtgaa ggtacggaaa gagagggatt

38881 cgaaccctcg gtaaacaaaa gcctacatag cagttccaat gctacgcctt gaaccactcg

38941 gccatctctc ctacataatg attatgaacc aaaaccctag ttaatagtgg gttcttcata

39001 ttccattcta gattattgga taggtacgac caatccattt taactatata ttaatattac

39061 aaataaaaat agaaaatttt tcatcaaagt aaagaaagat ctttctttcg aggctccaag

39121 ataaagagac aattgtttta ttatttctta aaattttttt tcaatcaatc tgtttttatg

39181 ttatttcgta ctgtacaatt cttttctttg tgggatcatt tttccaaaga aagggaatga

39241 gaagaattat aaaatggatt gctggctatg cctagatcgc gaataaatgg aaattttatt

39301 gataagacct tttcaattgt agccaatatc ttattgcaaa taattccgac aacttcagga

39361 gaaagggagg catttaccta ttacagagat ggtgtgattt gattcttttt ttttattgca

39421 tatctctcgg tcttacgaga aagacacgcg attcgggaaa ctttttgaaa taaatctacg

39481 cttgttgaag gtgaagtttg gaaatagaac aattccttct gtcgtgtatc ctcgattaat

39541 gcaacctcag atgctatgtt tccatttttg attctagtat tgagcgaaag gttacaccta

39601 gaggttctgg attatggggc aatcctactc cactagtacc aacggacagc ataagggaag

39661 aagcactaca cccaggaatc aacaacacga aaaccttgtt agaaattacc cctttcctta

39721 ttgggctcgg gactaaaaga atggttggga caacaaatat ccatctcgtt cgtactttgg

39781 ataccaatat aaccatcgaa ggttgttgaa gtgactaatt cctggaaatt cggaggcgtt

39841 gaaaacaaag aaattgttcg agttctcatt tatatctagt cccaacacgc ttgatgttaa

39901 gaaaagatct cttgcaggaa ggttggctag agatttcttg taaaaacact agccctgctc

39961 agtccataat gagaatattt tcatcttttt tgatttcatg tattattctc cttatgaaca

40021 taagggagga gccgtatgag gtgaaaatct cacgtacggt tctggaacgg agattctctt

40081 aattgaatgg cgaccgtaac ggatgtcagc tcaatccgaa ggaaattatg cggaagcttt

40141 acagaattat tatgaagcta tgcgactaga aatcgatccc tatgatcgaa gttatatact

40201 ctataatata ggtcttatcc atacaagtaa tggagaacat acgaaagctt tggaatatta

40261 ttttcgagca ctagagcgaa atccgttctt accacaagct tttaataata tggccgtgat

40321 ctgtcattac gtgcgactat cttcactata gaagtaaaaa aaagaaaaac aaaagggctt

40381 tctacatatg catcgtctaa aacaacgatt tttatcagct gtagaaaaga aagaaacttc

40441 atattcataa aagtggaaat atgaagaaat agatatacct agctactttt ttctatggat

40501 aaaaaaaaaa aagaatctaa ttggtaaaag aagcaccgta aagatgaatt agcggggttt

40561 ggggccgata caataataac tgcgtactta tgtcatgtga tgatggtaga tatacttatc

40621 tcatgatgtg agataaaaat taggaatcca cttatgtaat agagtcgatc cactaaagtc

40681 ttgagcagcg gtgtaggatc agatcccaaa gatagtaagt cttttctttc ttaggaagga

40741 aagtcttttt caaagattct atataaattt ctatacgaaa ccgagatagt tacctttcag

40801 aaaattctaa cgataggggg aattttttct tctgaaggtg ggaaaaaaga taaaacgaaa

40861 gaaaacaatc caaatttcag tttaaaactc atgtaatgca cctctttggt taacccgaaa

40921 ggtgggatag atccatgata aatcccattc gttagatatg ataacgggac accaaaagaa

40981 ttgaacgagg agccgtatga ggtaggaaac tctcaagtac ggttctaagg gaagggatta

41041 atccacctat tccgaccggg gagaacaggc cattctccag ggagattctg aaattgcgga

41101 ggcttggttt gatcaagctg ctgagtattg gaaacaagct atagcgctta ctcctggtaa

41161 ttatattgaa gcacataatt ggttgaagat cacgaggcgt ttcgaataaa agaaggcgct

41221 atttatttag ttagtttatt aatctcttat ttagtataat atatatatat atatatttga

41281 tatctttgtt ataattaata tgaattgttt ttggcctcat tggtcaaata aaaaaatgga

41341 tcattccgtt gttttgtgga aagagccaat caaagaattt cattatatcc cttctaagca

41401 ttctatttca tctgatattt cgtaaggata aaatgaaggg atagagtaca aaatacactt

41461 ctttttttta tttccatttt cttaaaacaa tttgaattaa aactaatata aggaaaccta

41521 aattaaatat cgaaaatcaa aattaacaac taaattatta aaatatattt caatatatat

41581 atacagtaat gtttcttagc aacggctgtt ctcgtgattt ggaatagatg aattcatatc

41641 accaggttgt acaaaaaaaa agatcgtttt tgtatcaatc cgaagctaca aaagggtttt

41701 atgagattca aaaggtccgt tgagcacctc atgactatgt cataatagat ccgaacactt

41761 gccccggatc gacttccaga tcataattgc tctagtaaat aactaaatca aatagataaa

41821 tgggagatag aaaaatagaa aagaaacaaa ctaattctag aaatatctct caaaggttta

41881 ctaattgttt gactgttggc aggcaggtct ctgtgtatgt gttgtccgga aagaggagga

41941 ctcaatgatt attcgttcgc cggaaccaga agtaaaaatt ttggtggata aggatcccgt

42001 aaaaacttca ttcgagcaat gggccaaacc tggtcatttc tcaagaacaa tagctaaagg

42061 gcctgatact accacttgga tctggaacct acatgctgat gctcacgatt tcgatagcca

42121 taccagtgat ttggaggaga tctctcgaaa agtatttagt gcccatttcg ggcaactctc

42181 catcatcttt ctttggctga gcggcatgta tttccacggt gctcgttttt ccaattatga

42241 agcgtggcta agtgatccaa ctcacattgg gccaagtgcc caggtggttt ggccaatagt

42301 gggacaagaa atattgaatg gtgatgtggg cgggggtttc cgaggaatac aaataacctc

42361 tggttttttt cagatttggc gagcatccgg aataactaat gaattacaac tctattgtac

42421 cgcaattggt gcattggtct ttgcagcgtt aatgcttttt gctggttggt ttcattatca

42481 taaagcggcc ccaaaattgg cttggtttca agatgtagaa tctatgttga atcaccattt

42541 agcggggcta ctaggacttg ggtctctctc ttgggcgggg catcaagtac atgtatcttt

42601 accgattaac caatttctaa acgctggagt agatcctaaa gagataccgc ttcctcatga

42661 atttatcttg aatcgcgacc ttttggccca actttatccg agttttgccg agggagcaac

42721 cccatttttc accttgaatt ggtcaaaata tgcggaattt cttacgtttc gtggaggatt

42781 agatccagta actggaggtc tgtggttgac cgatattgcg caccatcatt tagctattgc

42841 tattttgttc ctgatagcgg gtcacatgta taggaccaac tggggcattg gtcatggact

42901 aaaagatatt ttagaagctc ataaaggtcc atttacaggc cagggccata aaggcctata

42961 tgagatccta acaacgtcat ggcatgctca attatctctt aacctagcca tgttgggctc

43021 gttaaccatt gttgtagctc accatatgta ttccatgccc ccttatccat atctagctac

43081 tgactatggt acacaactgt cattgttcac acatcacatg tggattggtg gatttctcat

43141 agttggtgct gctgcgcatg cagccatttt tatggtaaga gattatgatc caactactcg

43201 atacaacgat ctattggatc gtgtccttag acatcgtgat gcaatcatat cacatctcaa

43261 ctgggcatgt atatttctgg gctttcacag ttttggtttg tatattcata acgataccat

43321 gagtgctttg gggcgtcctc aagatatgtt ttcagatacc gctatacaat tacaacccgt

43381 ttttgctcaa tggatacaaa atacccacgc tttagcaccc ggcgcgacgg cacctggtgc

43441 aacggcaagc accagtttaa cctggggggg tggtgattta gtagcagtgg gcggcaaagt

43501 ggctttgtta cctattccgt taggaaccgc ggattttttg gtacatcata tccatgcatt

43561 tacgattcat gtcacggtat tgatactcct gaaaggtgtt ctctttgctc gcagctcccg

43621 tttgatacca gataaagcaa atcttggttt tcgttttcct tgtgatggac ctggaagagg

43681 gggtacatgt caagtatcgg cttgggatca tgtcttctta ggactattct ggatgtacaa

43741 ttcgatttcg gtagtaatat tccatttcag ttggaaaatg cagtcagatg tttggggcag

43801 tataagtgat caaggggtag taactcatat cacgggagga aactttgcgc agagttctat

43861 tactattaat gggtggctcc gggatttctt atgggcacag gcatcccagg taattcagtc

43921 ttatggttct tcattatctg catatggcct ttttttccta ggtgctcatt ttgtatgggc

43981 ttttagttta atgtttctat tcagcggacg tggttattgg caagaactta ttgaatccat

44041 cgtttgggct cataataaat taaaagttgc tcctgctact cagccgagag ccttgagcat

44101 tgtacaagga cgcgctgtag gagtaaccca ttaccttctg ggtggaattg ccacaacatg

44161 ggcgttcttc ttagcaagaa ttattgcagt aggataatgg ctaggaggat ttgaaaggca

44221 ttatggcatt aagatttcca aggtttagcc aagggttagc tcaggacccc actactcgtc

44281 gtatttggtt tggtattgct accgcacatg acttcgaaag tcatgatgat attactgagg

44341 aacgtcttta tcagaatatt tttgcttctc acttcggtca attagcaatc atttttctgt

44401 ggacttccgg aaatctgttt catgtagctt ggcaaggaaa ttttgaatca tgggtacagg

44461 accctttaca tgtaagacct attgctcatg caatttggga tcctcatttt ggtcaaccgg

44521 ccgtggaagc ttttactcga gggggtgctc ttggcccagt gaatatcgct tattctggtg

44581 tttatcagtg gtggtataca atcggtttac gcactaatga agatctttat actggagctc

44641 tttttctatt atttctttct gccatatcct taatagcagg ttggctacac ttacaaccga

44701 aatggaaacc gagcgtttcg tggttcaaaa atgccgaatc tcgtctgaat catcatttgt

44761 caggactctt cggcgtaagt tccttggctt ggacaggaca tttagtacat gtcgctattc

44821 ctggatccag aggggagtac gttcgatgga ataatttctt agatgtatta ccgcatcccc

44881 aagggttagg cccgcttttt accggtcagt ggaatcttta tgctcaaaac cctgattcaa

44941 gtaatcattt atttgggacc tcccaaggtg caggaactgc cattctaacc cttcttggag

45001 gattccatcc acaaacacaa agtttatggc tgactgatat ggctcatcat catttagcta

45061 ttgcatttat ttttcttgtt gctggtcata tgtatagaac taatttcggg attggacata

45121 gtatcaagga tcttttagat gcacacgttc ctccaggagg gcgattggga cgtgggcata

45181 agggtcttta tgatacaatt aacaattccc ttcattttca attaggcctt gctctagctt

45241 ctttaggggt tattacctcc ttggtagctc aacacatgta ctctttacct gcttatgcat

45301 tcatagcaca agactttact actcaagctg cattatatac tcatcaccaa tatattgcag

45361 gattcatcat gacaggggct tttgctcatg gagctatctt tttcattaga gattacaacc

45421 cggagcaaaa tgaagataat gtattggcaa gaatgttaga ccataaggaa gctatcatat

45481 ctcatttaag ttgggccagc ctctttctgg gattccatac cttgggcctt tatgttcata

45541 atgatgtcat gcttgctttt ggtactccgg aaaagcaaat tttgatcgaa cccatatttg

45601 ctcaatggat acaatcggct catggtaaaa cttcatatgg gttcgatgta cttttatctt

45661 caacgagtgg cccggcattc actgcgggtc gaagcatctg gttacccggc tggttaaatg

45721 ctgttaatga aaatactaat tcattatttt taacaatagg gcctggagac tttttggttc

45781 atcatgctat tgctctgggt ttacatacaa ctacattgat cttagtaaaa ggtgctttag

45841 atgcacgtgg ttccaagtta atgccagata aaaaggattt tggttatagt tttccgtgcg

45901 atggtcctgg acgagggggt acttgtgata tttcggcatg ggacgcattt tatttggcag

45961 ttttttggat gctaaatact attggatggg ttacttttta ttggcattgg aagcatatca

46021 cattatggca gggtaacgtt tcgcagttta atgaatcttc tacttatttg atgggctggt

46081 taagagatta tttatggtta aactcttcac aacttatcaa tggatataac ccttttggta

46141 tgaatagttt atctgtctgg gcgtggatgt tcttatttgg acatcttgtt tgggctactg

46201 gatttatgtt cttaatctcc tggcgtggat attggcagga attgattgaa actttagcat

46261 gggctcatga acgcacacct ttggccaatt tgattcgatg gagagataaa cctgtggccc

46321 tttccattgt gcaagcaaga ttggttggat tagcccattt ttctgtaggt tatatattca

46381 cttatgcagc tttcttgatt gcttctacgt cgggaaaatt tggctaattt gggaatgtgt

46441 tatatccgcg ataatcacat ttttttcgat ggggagaagg cctgccttct tctattttat

46501 ttctacatct aggatttgac ttgtatcatg gatactaata ggacctgaac cattatggca

46561 aggaaaagtt tgattcagag ggagaagaag aggcaaaaat tggaacagaa atatcatttg

46621 attcgtcgat cctcaaaaaa agaaataagt aaagttccat cattgagtga caaatggcaa

46681 atttatggaa agttacaatc cccaccacga aatagtgcac ctacacgcct tcatcgccgt

46741 tgtttttcga ccggaaggcc gagagctaac tatcgagact ttggactatc cggacacata

46801 cttcgtgaaa tggttcatgc atgtttgttg ccaggagcaa caagatcaag ttggtaagga

46861 ttaatctttc attttattta tacggtcgat gatcataaag ggcctcttta ccattctgta

46921 taaatgtact attctatttg tacagatatg gtagaggggc acattcaatc cttctttgtc

46981 tattagtttt tacttcttct cttcagcgcg gggtagagca gtttggtagc tcgcaaggct

47041 cataaccttg aggtcacggg ttcaaatcct gtctccgcaa catctttttt tattttatat

47101 aaaattttga ggtttggctc ttaccccctt ttggggggag taggaagggg aggggggggg

47161 ggatagactt actatactat cacgaccaac tatacccaac cctttagcat attcaaaaaa

47221 ttactttatc ttgggcggat agcgggaatc gaacccgcat cttctccttg gcaaagagaa

47281 attttaccat tcgaccatat ccgcattttt gtttgtgata cacaatatat ccacacatat

47341 atgataacct gtatcatatt tatgcagtat cgggccggag actctcttca gcgttattcc

47401 aatcacttta gtaatctcat taattgtatt tttttcatcc aagaagtttg acccccctct

47461 aatttaaaat ttcatttttt tcattttctt tttttggggg gtcgttttgg ttgtggatct

47521 gggacaaata ggttcaagag atgagagaat ttaggatacc caccagaaag actaacccaa

47581 tccataatga tgtaccggaa aatacaacat ttttgttact tgaccaaccg tcgggagaag

47641 caaatacgac aggtacgcta atcaataaga ttgatgaagt agcaattaat gcaaaaacag

47701 ccaattggaa aacaagagtc atccttttaa tcctccaaga taccaacaaa taaactatac

47761 catttgatcc ctctatcagc caaaaaattg taagaaaata caccatcaag ggattttagt

47821 ttaccacgaa tcaattggac ttattactac ccctttgacc actctattcg tacatggagt

47881 cggtggaaat ggaattttat tttttatttc acaaacgggg catgctggat tctatatatg

47941 gatagatcga tcggtagatt cacacctgag atctttctac agatagtagt ggtatccccc

48001 cccctatggc catgttctat tcggaggaat aaaataaaaa ttgtctttcg gagagatggc

48061 tgagtggttg atagccccgg tcttgaaaac cggtatagtt ttgaacaaag aactatcgag

48121 ggttcgaatc cctctctctc ctttagtttg ctcattgaat cgatttgttt ctattggttt

48181 tgccgagatt ggtatcataa agaaagggga atggctcggc tatcccacct agccaagcca

48241 taaaaaatag aaaaatcgat tagtgggttg aaaaaaaaaa gaaatacttt tcaagtataa

48301 cccgatccca atatgtatga tggaatcaaa gtgattccta cttcaattca agcattggat

48361 ctcctgtctc atctcaatta agaggggtca tggaaagaac aggttcaaaa tcacgatcaa

48421 ttcctttttc aaatcctgct gcagctgcac gagcccttcc cgcgtgccac aaatgaccta

48481 cgaagaagaa gaatcctaga acaaaatgag aggtagctaa ccaacttcta ggagagacat

48541 aattgactgc attaatctcg gtagctacgc cacccacaga atttaaagaa cctaaaggag

48601 catgagtcat atattctgcg gaacgccgtt cttgccaagg ttgtatgtct tttttcaacc

48661 tactcaagtc caatccattt ggacctctta aaggttctaa ccaaggagca cgcagatccc

48721 aaaaacgcat agtttctcct ccaaaaatga cttctccggt cggggagcgc attaggtatt

48781 tacctaaacc agtaggtcct tgagcggatc ctacattagc cccaagacgt tggtctctaa

48841 ctagaaaagt aaatgcttga gcctgagaag cttctggtcc agtaggaccg taaaactcgc

48901 taggataggc ggtattattg aaccagacaa aacaacaagc agtgaaacca aagatggcta

48961 aagcccctaa actataagat aagtaagcct ccccagacca tacaagggcg cgtcgagccc

49021 acgcgaaggg tttagttaag atatgccaga ttccaccaag tatacaaatg gaacctaacc

49081 atacgtgtcc tccgattata tcttctaaat cgtccacact aacaatccac ccttctcccc

49141 caaagggaga ttttagtaaa taaccaaata tgatacttgg gctaagggtc aagttggtaa

49201 tttttcttac atctccccct cccggagccc aagtatcata tacgccccca aaataaagag

49261 ctttgaagac tagaagaaaa gcacctagac ccaataagat taagtgaatg cctaaaattg

49321 tggtcatttt atttctatct ttccacacat aaccgaagaa tggaaaagat tcttccagtg

49381 tctcgggtcc cagaagtgca tgataaatac cgccaaaacc caatactgca gaggaaatta

49441 aatgaagtac tccagataca aagtatggaa aggtatctat aacttctccc ccagggccta

49501 ccccccaacc tagagtagct agatggggaa gtaaaattaa tccttgttca tacataggct

49561 tctctggtac gaaatgagcc acttcaaata ggttcattgc tccggcccag aatacgatta

49621 atccggcatg ggctacatga gctcctagta gtttaccgga taaattaata agtcgggcat

49681 ttccggccca ccaagcaaaa ccggtggttt cttggtcacg accagctaca gataaagttc

49741 cattaaagag cgtttccacg tggtagaacc tcctcaggga atataaggtt ttcatgaggc

49801 tgatcttgag ccgccatcca agcacgaata ccttcgttta atagaatatt tttggtgtag

49861 aaagtctcaa attccggatc ttccgctgcg cgaatttcct gagaaacgaa gtcataggcg

49921 cgtaggttca gggccaaacc gactactcca agagcactca tccataaacc ggttactggt

49981 acaaataaca taaagaaatg taaccaacgt ttattggaaa aagcaacccc aaagatttgg

50041 gaccaaaagc ggttagcggt gaccatggaa taagtttctt cggcttgagt tgggttaaaa

50101 gcacggaatg tatttgcacc atcaccatct tcaaataaag tattttctac ggtagcacca

50161 tgaatagcgc atagcaaagc agcccccaat acgccggcaa ctcccatcat atgaaatggg

50221 ttcagcgtcc aattatgaaa tccttgaaaa aataggatga atcgaaatat agctgctaca

50281 ccaaaactag gtgcaaagaa ccaaccagac tgtcccagcg gataaatcag gaatacagaa

50341 acaaaaaccg caattggacc agagaatgcg attgcattat aaggtcgcaa ttgaacagat

50401 cgagcaagct caaattgacg taacatgaaa cctattaggg cgaaagcgcc atgcagagca

50461 acaaaagtcc acagaccgcc taattgacac caacgagtaa aatctccttg tgcttcagga

50521 ccccatagta acaataaaga atgcgctaaa ctattagcag gagtcgaaac tgcggctgtt

50581 aagaaattac agccttccaa ataggaactg gctaatccat gggtatacca tgaagttaca

50641 aaggttgtac ctgtgaacca accccctaaa gcgaaatagg cacaaggaaa gagcaatagg

50701 ccggaccagc ctacaaaaac gaaacggtcc ctccgtaacc agtcatccat aatatcaaat

50761 aaatcctttt cgtctttggt aaatttacca agggctatag tcatagtgat cctcctattc

50821 aactacttca accatttccg agcacctcat agtattttcg gggcgtctga agattcgatc

50881 atttctatat gatttctctt gcacggcccc taccgacggg tttcgaagat aaaaatcctt

50941 tattggccca caaactgacc tgagtaaatc catgaactca atttgattat tctttgttac

51001 taagtatatg aaccgtgaat tgtcttctta tgactcatca atcagataca tgaatctttt

51061 caaagaactt ttcaaggaac aagcgacccc ccttactact aaccgattcc cagatctatc

51121 cccccctctt cttcgattaa ctcgtgttat tctttgttat tcttagatct tgttcttgag

51181 attgcgaaaa agaaagctat ttctagattg ttcgaatttc tatgtatact aaacgattcc

51241 aattccatat acataatttt attaatttct tttttcattt tttttgtcag ataaatcaaa

51301 aattccagag tatatcttcc cacgggtcaa agcaacaaag acccttccag tatttttcta

51361 ttgagtctta tctcttagaa agaaagagac tttcctcccc ctttcccttt tttttcgtta

51421 aattcaataa aaaaaataga agacttgaaa tgaaagtttc tttctaacat ccattacact

51481 gtcgtaacaa aaatatcgga tgcgacaatc atagaaatgt ttggtacatg aagtcgtgat

51541 ctgatagcta tacatctaaa tagacttaga tagatagaaa ttcatcttga tccgtaatca

51601 aagaacaata gtggaaatgg ctcttatctt gtgacttgaa agataggaac aagaagattg

51661 aatcggaaat atcgacaaat tctttacttt ccaagtccaa agaaatgaaa ttaaaaaggg

51721 ggggtgtccg gctgttctaa ttcaaattcg atctctatct aatttgaata tcagaatagc

51781 ggatatagtt ataattaaat gggtcaggtc cacttacttt ttcttttgtt ttcttgattt

51841 caaatctttc cggtggattt gcttggtata atggaaaata gggatctttg gcgattcaag

51901 aggcggctta tgattattca taataatgaa aattttccga acaaatgttc cattttttaa

51961 ctagaactac tatactctaa ctcaactcta actcagtata atgataacgt attatccggt

52021 tagttccttt tttatcccaa atacaaaaaa aagcccctta tcggatttga accgatgact

52081 tacgccttac catggcgtta ctctaccact gagttaaaag ggcttatttg atttcattcc

52141 cggacgagtc actatgtaga taaaatctct atatgcaaat attatatatt atatacatat

52201 acatatatat atatataaat agattatata catatatata taaatagatt atatacatat

52261 atatatatac agtatactca tagtgactag tagcttattt ttgactaatc caatggattt

52321 tcctagaaaa tctagggtaa aatgaattgt ttcaagaccg gccctaattt cttttattga

52381 gatgatcctt aatgaaaaca aaattcactt aattgataca taacatacac ttactaattt

52441 gatctaaaat gaatttcaag acatttcatc gaatcatgtc atgaagagat tctactccct

52501 cctgaaacta aagtcttaaa gaacattttt gataactttt ttattttgct cccgcttact

52561 agattcgatt tatctagaga atgtcgattc taatgaacga ttcatgaata tgaatgacga

52621 atcaaaaaat tctatacaat tatgaaaaac ggaaggatcc cccggatctg atcattccat

52681 tatattgaca atttcaaaaa actgatcata ctatgatcat agtatgaggg cggttggtca

52741 agttggcccc catcgtctag tggtttagga catctctctt tcaaggaggc agcggggatt

52801 cgaattcccc tgggggtagg atactatgaa aggaagttga tcatggatta ctaataagcc

52861 taaaattgat tcttcctggg tcgatgcccg agcggttaat ggggacggac tgtaaattcg

52921 ttggcaatat gtctacgctg gttcaaatcc agctcggccc aataatccgc taatctaaca

52981 tgagatggta caaatcccct gtccttcaga aagacccaac gaagataaaa aaaagaatct

53041 aattttctgc gagatcccct atttcctttc cccgggattg tagttcaatc ggttagagca

53101 ccgccctgtc aaggcggaag ctgcgggttc gagccccgcc agtcccgacg gatcaaaatc

53161 aaataaatac atcaattcat tttttccctt ctatgaacta gtaaagggtg gcgaggggca

53221 tactttcatt tccaaagcaa acattcccaa agcaaaaagg agtctttatt tctttttgct

53281 ttcctatttt ttccatttcg cttttattgt ttatttaggt ttagaactat gtaattaatc

53341 gaagtggaaa ggcaatctga tgatccttta tcagacgatt gtccaaggaa gacgcaaggc

53401 aggttataga aaaaacaagg aaacagatga gaaataaagg aattttggat tgattgagtc

53461 aggaatccaa atttggattc ggtatggata aaagaacttc ctatgttata ctattcaagt

53521 cttgacgacg ggttgatttg ctagatcaga tattgttatt catgatattg atccgattca

53581 agatcatcga gaggtaattc attcattgaa tttacagccg aaagatttat catctctagg

53641 ggattaaatc ccgagttatt gcgaagtaaa aaaaccgatg agattatgga agtaaatatt

53701 cttgcattta ttgctactac actattcatt ctagttccta ccgcctttct gcttatcatt

53761 tacgtaaaaa cagtcagcca aaatgattaa ttcaaatgaa tttgaaaatt catttgaatt

53821 aaactttcct tctgagttct tatcaaaaat tgacgaagtc aaatgaaaag gattccaatt

53881 ttacgtctta acttaaatga aacgagcgca ctataatcag cagttttcta agagatagat

53941 agtatggtag aaagatgcat ctttctacca tactattaac taataagcaa ggggaaactt

54001 tgcctatttt taacgcccaa accctgatat gaaataagtc gagaaagcaa aaagcgcctt

54061 tctcaaatta gaaaacaaag ggtaaatgcc catgcctcgc cccagtcaag atcttattat

54121 tgcccagtct ctcgttagac aaccaccatc cctatatcat ttctcctaga aagtgcgtat

54181 acacgtaaca aaacgactta ttctttgaag agtaaagagg gaaacaaata aaaaaagggg

54241 ggtccgtctt cctcagtatc catctataaa aatagtaaga gcccattccc gttgattgaa

54301 atagaaaatt tcggaagaat ttgaggttgg aataaatttc caatcatctg aatccctttc

54361 ttttcgaaat acaatacaag ggcgggaatc gatgaaatat ctctttaatt gaaaattgaa

54421 agagtatgat tcatatcata gattactcga ttggagtcca atgagattcc aaattcagag

54481 attttgattt gaaatagttt caaatcaaat gcgttgcaga acgatctata aaacaatcga

54541 tacggtttta tttttgattc ctgaactcaa ttagtagtac ttctagagcc cacttcttcc

54601 ccacactacg agtgaaaggg aaaatgtaaa gactaccatt aaagcagccc aagcgagact

54661 gactatatcc atgtgcatta tgtcccctat ctctataaat acgaaggaat tattccatta

54721 ttcctcacta ataatagtgg aatcaatgcc gcagagtcaa aaaggggttc tgaccgaaca

54781 ctattgagaa agcaatccaa taaatcgatt atgatttcga atcaggaaag attaaaaaca

54841 caagcaaaat acatagtaac atctcaagga aatgggaaca tgtaggaata ataaggtcta

54901 ttcttttttt gttttgttta cattctaagt aaaaacagaa ggggtgaaat cactaaaaac

54961 gagaaatcac tatctattac agatctctat ttccccattt gatcgaatcc gtaccccctt

55021 ttccgattat ccctgcgaaa cagggggctt gggtaggcgt taccgaaaag aaagcatttc

55081 tttttactct cttttctaga aaagtcaatt attcatccaa tctaatattg attggatacc

55141 tgagcactca gagattctcg caagtccgtc ttatataaag caacttccga cccctcccca

55201 aactattaat tgaatttcta ggctctacaa tttgattcaa gatccagtca ttagccactt

55261 ccttagtaat agaagggaat cgtgaagtct tgataacccc tctaccagta gattcgaata

55321 tttccttgaa tcctagatgc gaacggggct tcacaatctt ttcttttaga aaacctttag

55381 accacatact tagaatcaaa caaagtatca acagcccgtt tcctatgctt ctacggagga

55441 agcatttttc gagttctatc agaagaacag aaaagaagaa gagatttcga gtttttggta

55501 atcaggcgac acccggattt gaactgggga aaaaggattt gcagtccccc gccttaccac

55561 tcggccatgc cgccaaaatg atacaaaaat acttttcttc caaaccccct tttggttgta

55621 tttgatccgc cccttcaatt tattgtatag tatctgaaaa tacacatttg attgttcaat

55681 agaaaaacga gagaatcttt ttagcattgt gtattttcag ccgataaatt ggaaccatta

55741 actattgact ccttagttcg aattcatgaa cgattctggg atttgaattt caaattgtgt

55801 tttcctaatg acataagaaa ataaaaattg agaccgaacc aatcagtatt gattttttca

55861 atcaaaaaat ctttctccac ttcaattata acaaaacata tgagtatatg taaaccccag

55921 aacataaatt gttacacaga tatctattgt ttagatatgt ttatttagat atgttgtcga

55981 tagggaatta tcataaaatg atcctacttc atgcattgaa tagaaattat cttttgatag

56041 agcacagcca gggctattcc gaatagaacc ggcaataaaa gagaagtcgg atattcatat

56101 tctagctatc tggatacgtg tttaataaat gcaacccttc ttatacaata cacttccaat

56161 tgtattctac tcaaaaataa gtaaagtaca aatatctctc ttctctttga atcgtttttt

56221 gaacaaggaa atccctagct aaggtggtta agtgctaaaa aatggattct atcttatttt

56281 ttgtctttgt ctcccaaata ccgaatcttt ttctttttct caaattcgaa tatgtaatat

56341 cataataatg gtctaatttg aatcattttt ttttgttttg cgattctata caaaacccta

56401 tgaccttaat aagcctaagt ggcagaattc tgtttctagg attgttctat caactctttt

56461 ccattttgat ctgtcgcaac ttccaattta agtagaaaat tatctgtatt tcttcgttca

56521 tacgtagttc atacatttca tttcaaatat ggagttggct aaaatttcat gtgattcagt

56581 aaacagaata gaaattccat cagtactaga agcgtcgatc taattcgacg aagaatgtac

56641 ttaataatat aatgggattt tttgataaat gggaaattca aaatgctcgg ggatgcaaat

56701 gaggcaatgt ctacaatacc tggatttcat cagatccaat ttgaaggatt ttgtaggttc

56761 atcaatcggg gtttgacaga agaactttac aagtttccaa aaattgaaga tacagatcat

56821 gaaatcgaat ttcaattatt tttggaaaga tatcaattgg tagaaccgtt gataaaggaa

56881 agaaatgctg tatatgaatc actcacatat tcttctgaat tatatgtatc cgcgggatta

56941 atttggaaaa ccagtaggga tatgcaagaa caaactattt tggttggaaa cattcctcta

57001 atgaattccc taggaacttc tatagtaaat ggaatatata gaattgtgat caatcaaata

57061 ctgcaaagtc ccggtattta ttaccggtca gaattggacc ataacggaat ttcggtctat

57121 accggcacca taatatcaga ttggggagga agatcagaat tagagattga tagaaaagca

57181 aggatatggg ctcgcgttag taggaaacaa aaaatatcta ttctagttct atcatcagct

57241 atgggttcga atctaagaga aattctagac aatgtttatt atcctgaaat ttttttgtct

57301 tttctgaatg ataaggagag aaaaaaaatt ggatcaaaag aaaacgccat tttggagttt

57361 tatcaacaat ttgcttgtgt aggcggggat ccggtatttt ctgaatcctt atgtaaggaa

57421 ttacaaaaga agttctttca acaaagatgt gaattaggaa ggattggtcg acgaaatatg

57481 aaccgaagac tgaaccttga tatccccccg aacaatacat ttttgttacc acgagacata

57541 ctggcggccg ctgatcattt aattggactg aaatttggaa tgggtacgct tgacgatatg

57601 aatcatttga aaaataagcg cattcgttct gtagcggatc ttttacaaga tcaattcgga

57661 ttgtctctgg ttcgtttaga aaatgtggtt cgaggaacta tatgtggagc aattcggcat

57721 aaattgatac caactcctca aaatttggta acttcaactc cattaacaac tacttatgaa

57781 tctttttttg gtttacaccc tttatctcaa gttttagatc gaactaatcc gttgacacaa

57841 atagttcatg gtagaaaatt gagttattta ggtcctggag gactaacagg gcgaactgct

57901 agttttcgga tacgagatat ccatcctagt cactatggtc gtatttgccc aattgacaca

57961 tcggagggaa tcaatgttgg gcttattgga tccttagcaa ttcatgcgag gatgggttat

58021 tggggatctc tagaaagccc gttttataaa atttcggaga gatcaacagg gttacggctg

58081 ctttatttat cacccggcag agatgaatac tatatgttag cggcaggaaa ttctttggcg

58141 ttgaatcagg atattcagga agaacaggtt gttccagctc gataccgcca agaattcctg

58201 actattgcat gggaacgggt tcatcttcga agtatttttc ccttccaata tttttctatt

58261 ggagcttccc tcattccttt tattgaacat aatgatgcga atagggcttt aatgagttct

58321 aatatgcaac gtcaggcagt tccgctttct cggtccgaga aatgtattgt tgggactggg

58381 ttggaacgac aagcagctct agattcaggg gctcttgcta tagccgaacg cgggggaaag

58441 atcatttata tagatactga caagatcctt ttctcgggga atggagatac tctaagcatt

58501 tcattagtta tgtatcaacg ttccaacaaa aatacttgta tgcatcaaaa aacccgcgtt

58561 cagcggggta aatgcattaa aaagggacaa attttagcag atggtgctgc tacggttggt

58621 ggcgaacttg ctttgggaaa aaatgtatta gtagcttata tgccgtggga aggttacaat

58681 tctgaagatg cggtactcat tagtgagcgt ttggtatatg aagatattta tacttctttt

58741 cacatacgga aatatgagat tcagactcat gcgacaagtc aaggccccga aaggatcact

58801 aacgaaatac cacatttaga agcccgttta cttcgcaatt tagataaaaa tggaattgtg

58861 atgttgggat cttgggtaga gacgggtgat attttagtag gtaaattaac gccccaaatg

58921 gtgaaagaat cctcgtatgc ccccgaagat agattgttac gagctatact cggcattcag

58981 gtatctactt caaaagaaac ttgtttaaaa ctccctatag gtggtaaggg ccgggttatt

59041 gatgtgaggt ggatccagaa aagaggaggt tctagttata atccagaaac gattcgtgta

59101 tatatttcac agaaacgtga aatcaaagta ggcgataaag tagctggaag acacggaaat

59161 aaaggtatca tttcaaaaat tttgcctaga caggatatgc cttatctgca agatggaaga

59221 cctgttgata tggtcttcaa cccattagga gtaccctcac gaatgaatgt aggacagata

59281 tttgaatgtt cgctggggtt agcagggggt ttgctagaca gacactatcg aatagcgcct

59341 tttgatgaga gatatgaaca agaagcttcc agaaaactag tgttttctga attatatgaa

59401 gccagtaagc aaacagctaa tccatgggta tttgaacccg agtatccagg aaaaagtaga

59461 atatttgatg gaagggcggg gagtcctttt gaacaacccg ttataatagg aaaaccttat

59521 atcttgaaat taattcatca agttgatgat aaaatccatg ggcgttccag tggacattat

59581 gcgcttgtta cacaacaacc ccttagggga agggcaaaac aggggggaca gcgagtagga

59641 gaaatggagg tttgggctct agaaggattt ggtgttgctc atattttaca agagatgctt

59701 acttataaat ctgatcatat tagagcgcgc caagaagtac ttggtactac gatcatcgga

59761 ggaattatac ctaatcccga ggatgctcca gaatcctttc gattgctcgt tcgagaacta

59821 cgatctttag ctctggaact gaatcatttc cttgtatctg agaaaaactt ccagattaat

59881 aggaaggaag cttaatcaga ataaatccga atttttattc tatgatcgat cggtataaac

59941 accaacagct ccgaattggc ttagtttctc ctcaacaaat aagcgcttgg gctactaaaa

60001 tcctgcccaa tggggagatc gttggagagg tgacaaaacc ctatactttt cattacaaaa

60061 ccaataaacc ggaaaaaggt ggattatttt gtgaaagaat ttttgggcct atcaaaagtg

60121 gaatttgtgc ttgtggaaat tatcgagtaa tcggagatga aaaagaagac ccgaaatttt

60181 gtgaacaatg cggagtcgaa tttgttgatt ctcggatacg aagatatcaa atgggctaca

60241 tcaaactcgc atgcccagta acccacgtgt ggtatttaaa acgtcttccc agttatattg

60301 cgaatctttt agataaacct cttaaagaat tagaaggcct agtatactgc gatgtgtgat

60361 ttgatcaaaa ttcttatttt acagattcgg aatgagaaac tgtcatccca atcaatccaa

60421 ttgggatgcc ctggatctaa catgtgactt ggtaagagta ccatgaagct cagacttagg

60481 ggtgtagtca atactcccaa atcaaaaggg ggattgatct atggtcgatt ttggaacaaa

60541 taaaaaaaat ttgatttgta cctcgtaaaa aatatttatt tgttttgtga gattaagcat

60601 ttactttctt ttggaaagaa attttatgtt caagcaagca aatatgtcgt ggttacagga

60661 gtctatccat cgcgtatagg ctttaataag ggcatcgtgg cataaccgtc gaggcgaagt

60721 agggacctaa cagatcgaat ggaacaatac atagacaagt aaatccctta tgaattacaa

60781 ggtaatcctt tattttttta ttttaatttt actttaattc gaattaagaa ttaagggatt

60841 catcattcga agggaagtag actactcaag aatttcacat ttcatttctg tcgtaatgga

60901 ataacacgaa ttaatcaaat cgaaataata tgaataaata aaaaatacaa agaaggcgga

60961 atcaatgaaa ttttgcttag tcttcgctgg gaacttgagt aaagagtaga tctttttgtt

61021 ttgaggtttt ctataatttg aaagggagaa ctcctttctt gaattggtgt acctacttga

61081 gccggatgaa aggaaacttt cacgtccgat tttgaagggg gggagatcct ataggatcct

61141 atctcaattt ttcttttgct aggcccataa cgaaaaaacc cactttcttg cgattacgag

61201 gtttattcga atatgaaatc caatcttgga aatacagcat cccacttttt tttactaccc

61261 agggcttcga tacatttcga aatcgagaaa tctcgaccgg tgcaagtgct attcgagaac

61321 aattagccga cctagattta cgcattattc tacataattc tttggtagaa tggaaagaat

61381 taggggaaga agggcccacg gggaatgaat gggaagatcg aaaggttgga agaagaaagg

61441 attttttggt tagacgcatg gaattggcta agcattttct tcgaacaaat atagaaccag

61501 aatggatggt tttgtgtcta ttaccagttc ttcctcctga gttaagaccc atcattcaga

61561 tagatggggg taaattaatg agctcggata ttaatgaact ctatagaaga gttatctatc

61621 ggaacaatac ccttaccgat ctattaacaa caagtagatc tacgccggga gaattagtaa

61681 tgtgtcagga gaaattagta caagaagctg tggatacact tcttgataat ggaatccggg

61741 gtcaaccaac gagggacggt cataataaag cttacaagtc attttcagat gtaattgaag

61801 gcaaagaggg aagatttcgt gagactctcc ttgggaaacg ggttgattat tcagggcgtt

61861 ccgtcattgt cgtaggtcct tcactttcat tacatcgatg tggattgccg cgcgaaatag

61921 caatagagct tttccagaca tttgtaattc gtggtctaat tagacaacat cttgcttcaa

61981 acataggagt tgctaagagt aaaattcgag aaaaagaacc gattgtatgg gaaatactgc

62041 aggaagttat gcaggggcat cctgtattgc tgaatagagc acccactctg cataaactgg

62101 gcatacaagc attccagccc attttagtgg aggggcgcgc tatttgttta catccattag

62161 tttgtaaggg attcaatgca gattttgacg gggatcaaat ggctgttcat gtacccttat

62221 ctttggaggc tcaagcagag gcccgtttac ttatgttttc tcatatgaat cttttgtctc

62281 cagctattgg agatcccatt tccgtaccaa ctcaagatat gcttattgga ctctatgtat

62341 taacgagcgg gaatcgtcga ggtatttgta taaataggta taatccgtgg aatcgcaaaa

62401 actatcaaaa taaaagaagt gacaataata aatataagta tacgaaagaa cctttttttt

62461 ctaattccta tgatgcaatt ggggcttatc ggcagaaacg aatcaattta gatagtcctt

62521 tgtggcttcg gtggcgacta gaccaacggg ttattgcttc aagagaaact cccatcgaag

62581 ttcactatga atctttgggt aattattatg agatttatgg acactatcta atagtaagaa

62641 gtataaaaaa agaaattctt tttctataca ttcgaaccac tgttggtcat atttctcttt

62701 atcgagaaat cgaagaagcc atacaggggt tttcccgggc ttgttcatat ggtacctaac

62761 taggctaagg aattctacga taccaatagg aattcagatc tcttcacttc aaccaggccc

62821 gtagttccgg aatttctacg cgaatcaaga tcgagaaggg gaagttttcc aaccactgac

62881 tcaaacccat tgtcaaatcg tactcagcag aatatggagg tacttatggc agaacgggcc

62941 aatctggtat ttcacaataa agtgatagat ggaactgcca tgaaacgact tattagtaga

63001 ttaatagatc acttcggaat ggcatataca tcacacatcc tggatcaagt aaagactttg

63061 ggttttcaac aagctactgc tacatccatt tcattaggaa ttgatgatct tttaacaata

63121 ccctcgaaga gatggctagt tcaagatgct gaacaacaaa gttttatttt ggaaaaacac

63181 caccattatg ggaatgtaca cgcggtagaa aaattacgcc aatccattga aatatggtat

63241 gccacaagtg aatatttgcg gcaagaaatg aatcctaatt ttaggatgac tgaccccctt

63301 aatccagttc atataatgtc tttttcggga gctagaggaa atgcatccca ggtacatcaa

63361 ttagtaggta tgaggggatt aatgtcggac cctcaaggac aaatgattga tttacccatt

63421 caaagcaatt tacgcgaagg gctttcttta acagaatata taatttcttg ctatggagcc

63481 cgtaaagggg ttgtagatac tgctgtacga acatcagatg ctggatatct cacgcgcaga

63541 cttgttgaag tagttcaaca cattgttgta cgtcgaatag attgtggcac cgcccgtggc

63601 atttctgtga gtcctcagaa tggtatgatg ccagaaagga tttttatcca aacattaatt

63661 ggtcgtgtat tagccgatga tatatatatg ggcacacgct gtattgccac tagaaatcaa

63721 gacattggga ttggacttgt aaatcgattc ataacctttc gagcacaacc aatagctatt

63781 cgaactccct ttacttgtag gagtgcatct tggatctgtc gattatgtta tggccggagt

63841 cctactcatg gcgacctggt tgaattggga gaagctgtag gtattattgc aggccaatca

63901 attggagaac cgggcactca attaacatta agaacttttc ataccggtgg agtattcaca

63961 gggggtactg cagaacacgt gcgagcccct tctaatggaa aaatcaaatt caatgaggat

64021 ttagttcatc cgacacgtac acgccatggg catcccgccc ttctctgttc tataaacttg

64081 tatgtaacta ttgagagtga agatattcga cataatgtaa atattccatc ccaaagtttt

64141 cttttagttc aaaacgatca atatgtagaa tcagaacaag tgattgccga gattcgcgcg

64201 ggaacatcca ctttgaattt taaagagaag attcgaaaac atatttattc tgactcagac

64261 ggagaaatgc actggagcac cgatgtatat catgcacccg aatttacata cggcagtgtt

64321 catctattac caaaaacaag tcatttatgg atattattag gagagccacg cggatccagt

64381 ctagtttcac tttcgatcta caaggatcaa gatcaaatga gtgcgaattc tcgttctgtc

64441 aagagtttta acctttcagg gacggatgat cagttgagag aaaaattctt tacttcagat

64501 ttttcgggta aaaaagaaga taggattcct gattattcag accttagtcg aattatatgt

64561 actggtcgtt gtaatctcat agatccgacc cttctctacc agaattctga tttattctca

64621 aagaggcgaa gaaatagatt catcatccca ctccaatcga ttcaagaacg cgagaacgga

64681 ctaacgcccc cttcagatat cttgattgaa atccctatca acggcatttt ccgtagaaat

64741 agtattcttg cttatttgga cgatcctaga tacagaagaa agagttcggg cattactaaa

64801 tatgggactc tagaaatgca ttcaatcgtc aaaaaagagg atttgattga gtatcgagga

64861 ggcaaggaat ttagtccaaa ataccaaatg aaagtcgatc ggtttttttt cattcccgag

64921 gaagtgcata tattacccgg atcttcttcc ataatggtac ggaacaatag tatcattggg

64981 gtagatacac aaattacttt aaatatgaga agccgcgtag ccgggttggt ccgagtggag

65041 agaaaaaaaa aaagaattga acttaaaatc ttttctggag atatccattt tcccggagag

65101 acagataaga tatcccgaca tagcggcgtt ttgataccac taagaacagg aaaacgaaat

65161 tctaaggaat cgaaaaaacg ggaaaatggg atctatgttc aacggatcac acctagtaag

65221 aaaaagtatt ttgttttggt tcggcctgtc gtcacatatg aaataacgga cggtataact

65281 ttaggaacac ttttccctcc ggatctgttg caggaaaggg ataatgtgaa acttcgagtt

65341 gtcaattata tcctttatgg aaatggtaaa ccaattcgag gaatttctga tacaaatatt

65401 caattagttc ggacttgttt agtattgaat tgggaccaag acaaaaaaag ttcttctagt

65461 caagaagccc gtgcttcctt tgttgaaata agggtaaatg gtttgattcg acatttacta

65521 agaatcgact tgctgaaatc cactttttca tatatcggaa aaagaaatga tccctcgggc

65581 tcaggattgt tctcggataa tggatcagat tgcacaaaaa gaaatccgtt ttcttcgatt

65641 tattccaagg caataattca acaatccctt aatcaaaata aaagaactat tcatacgttg

65701 ttgaatagaa atgcgggatt ccaatcgttg ataattttgt catcatccaa ttgttttcga

65761 atgggtccat tcaacgatgt aaaatatcac aatcacaatg tgatacacaa tgggataaaa

65821 gaatcaatta acattacaaa agatcctgta attccaattc agaattcgct ggggccttta

65881 ggaacagtcc ctctaattcg aattgcgaat gtttattcat tttaccattt aataactcat

65941 aatcagatct tggtaaagaa ctatttgcaa cttgacaatt taaaacagac ctttcaagta

66001 attaaattga aatattattt aatcgacgaa aaggagaaaa tttataaccc cgatccatgc

66061 agtaacatta ttttcaattt gaattggtat tttctccatc ccaattattg tcaagaaaca

66121 tctacaataa tgagtcttgg gcagtttatt tgtgaaaata tatatatatc caaaagcgca

66181 ccacacctaa aatcgggtca agttatactt gttcaagttg actctgtagt aatacgatca

66241 gctaagactt atttggccac tccgggagca actgttcatg gccattatgg ggaaatcctg

66301 tatgaaggag atactttaat tacatttata tatgaaaaat cgagatccgg tgatataacc

66361 caagggcttc caaaagtgga acaggtgtta gaagtgcgtt cgattgattc aatatcgatg

66421 aatctagaca agagaattga gggttggaac gaacgcataa caagaattct tggaatgccg

66481 tgggcattct tgattggtgc tgagctaact atagtacaaa gtcgtatctc tttggttaat

66541 aagatccaaa aggtttatcg atcccagggg gtgcagattc ataataggca tatagaaatt

66601 attgtacgtc aaataacatc aagggtcttg gtttcagaag atggaatgtc taatgttttt

66661 tcgccgggag aactaattgg attggtgcgt gcggaacgaa tggggcgtgc gttggaagaa

66721 gcggtttgtt atcgagccct tttattggga ataacaagag catctctcaa tactcaaagt

66781 ttcatatctg aggcgagttt tcaagaaact gctcgagttt tagcaaaagc agctctccgg

66841 ggtcgaatcg attggttgaa aggcctgaaa gagaacgttg ttttgggggg tatgataccc

66901 gtcggtaccg gattgaaagg attcgtgccc ccttcaaaac aacataacag gagccccttg

66961 gaaatgaaaa aaaaaaaaaa aaatctattt gaaggggaaa tgagagatat tttgtttcac

67021 catagaaaat tatttgattc ttttctttca aataatttgg atgatagacc agaacaatca

67081 tttataggat ttaatgattc ctaagagcag attcatcttt ttcactatcg gtatatgtat

67141 ttggtgcagt catttgattc ggtaatcaaa atagtaagca atagaaaaga ctcattaatg

67201 gcttattcgt ctatctacag ccaatctacg gtagaagaga aggttccatc ggaacaattt

67261 tttatttcag ttcgggggtc ctcgtctctt tgttttttga aaaaaggggg ggtgtgggga

67321 gaaatgacaa gaagatattg gaacatcaac ttggacgaga tgctggaggc aggagttcat

67381 tttggccacg gtactaggaa atggaatcct aaaatggcac cttatatctc tgcaaagcgt

67441 aaaggtattc atattacaaa tctgactaaa actgctcgtt ttttatccga agcctgcgat

67501 ttggtttttg atgcagcaag caggggaaaa caattcttga ttgttggtac caaaaaaaaa

67561 gcagctgatt cagtagcacg ggctgcaata aaggcccggt gtcattgtgt taataaaaaa

67621 tggctcggcg ggatgttaac aaattggtcc actacagaaa cgagacttca taagttcagg

67681 gacttgagga tggaacaaaa aacgggaaga ctcaacggtc ttccgaaaag agatgcggct

67741 gtggtgaaaa gacaattata tcgcttacaa acatatctgg gcgggattaa atatatgaca

67801 gggttacccg atattgtaat catcgtggat cagcatgaag aatatacggc cctgcgagag

67861 tgtatcactt tgggaattcc aacgatttgt ttaatcgata ctaattgtga ccccgatctt

67921 gcagatattt cgattccagc gaacgatgac gctatatctt caatccgatt aattcttaat

67981 aaattagtat tcgcaattag tgagggccat tctagctata taagaaatcc ttgattaata

68041 atgattacta ataagctaaa tcacttttcc tcgaaagatt tatggaatcg ggtattattt

68101 atgaattttt tcaaatagat aaaaatacct ggggatatta tgtgattagt tagtattcaa

68161 aatattagtt ggtattcaaa atatccgatt caagtagaca aagagaaaga gatggttgaa

68221 tcaaaataat tttgttttaa gttcgatttt tttcagaggg caatatgaat gtgctatcat

68281 gttccatcaa cacactaaaa ggattatatg atatatccgg tgtggaagta ggccaacatt

68341 tctattggca aatagggggt ttccaagtac acggccaagt acttattact tcttgggttg

68401 taattgccat cttattaggt tcagctacta tagctgttcg gaacccacaa accatcccga

68461 ccggaggtca gaatttcttc gaatatgttc ttgaattcat tcgagatgtg agtaaaactc

68521 aaattggaga agaatatggc ccttgggttc cttttattgg aactatgttt ctatttattt

68581 ttgtttctaa ttggtcagga gctcttttac cttggaaaat catagaatta cctcatggag

68641 agttagccgc acctacgaat gatataaata ctactgttgc tttggcttta ctcacgtcag

68701 cggcatattt ctatgcgggt cttaccaaaa agggattaag ttatttcggg aaatatattc

68761 aaccaacccc aatcctttta cccattaata ttttagaaga ttttacaaag ccgttatcgc

68821 ttagttttcg acttttcggg aatatcttag cggatgaatt agtagttgtt gttcttgttt

68881 ctttagtacc ttcggtgatt cctatccccg tcatgttcct tggattattt acaagtggta

68941 ttcaagctct tatttttgca acgttagccg cggcttatat aggtgaatcc atggagggtc

69001 atcattgaaa tcgttttttt cgcttagcgc aaagcaatgt atgcatggct caagatgatt

69061 gacttaagga atagaaatat acaagactct atatacgata gaaagaaata ggaacaaaaa

69121 attaaaaaaa ttacgatatt atagagttgg aaaaaaaaaa ggaaagagat ctaaaagatc

69181 tttttcctaa aattatcttt ttgtacactt aatatcctac ctttgcttga cgaatattca

69241 ctttctatta tattggtcag ccaatattat tattcaaatc ataatttgaa taagatctat

69301 tttacgacgt gtcattaaat aaaaaacagg gggggttcta ctttacagtt gattttattc

69361 aacaattgac caaaagaaaa ttataataaa taatatcatg aacttagatc aatcaaattt

69421 taaataataa aatttctatg caataattcg cactaatcaa ttgaatttac ccatttagat

69481 tgtactctcc gataaacaaa gcggaagtga gaaaagaatt tacgcgggat tcctaaaaaa

69541 gtggattgat tgtcgaatcc gattgaatct aatggtttac gttatggaag aaagacatgt

69601 atatgtgata ttagatattg actcgttata tatatgaact aaagatattt tttatttgcc

69661 ctactcctgt gtgtgagttc taatagaagt cctttcatat cgttgtggct gtgaattggc

69721 tgaaaaaaga gatgaaatcc agaaaagatg gaagaattta aataacagtt cgaaatcacg

69781 aaataaagtt ctacgcattc acaaaaactc tgtggataga aacaaaaaag agatatcgaa

69841 gtagttctga tgattcaata atactcttac ttaaattcga agttcttagt tatttccact

69901 ggatgaatcc tatcgatgga agtcctgcct agttcattgg ttgattgtat cattaaccat

69961 ttctttttgt tggtatgagg aatttatcat gaatccactg atttctgccg cttctgttat

70021 tgctgctgga ttggctgtag ggcttgcttc tattggacct ggagttggtc aagggaccgc

70081 tgcgggtcaa gccgtagagg gtatcgcgag acagcccgag gcggagggaa aaatacgagg

70141 tactctattg ctgagtttag cttttatgga agctttaacg atttatggat tggttgtggc

70201 actagcactt ttatttgcga atccttttgt ttaatcttcg aaataggaaa aataaaaatt

70261 ttcctatttt attgccttgg gcttgtcgct tgctttttca aattagatca cgatttcact

70321 ccgacaattc cttattcgtt gagaaaataa cctacgggaa aggctgattc gcggatgcgg

70381 aattagtatg ccgactcgct ttcatccttc ccgttcgtag tcaaagggaa actctttttt

70441 tttatgaggt gttgcaacaa tgaaggggta gttcatactt taactagaag attttttgac

70501 tcgatttcat aaaaaaaaaa aaaagaataa atcaaaaaga ggggcaaagt ggtagaaaaa

70561 gaactctcgt cgatttttta gtctatctat aagaggagat tatatgaaaa atgtaaccga

70621 ttctttcgtt tctttgggcc actggccatc tgccgggagt ttcggattta ataccgatat

70681 tttagcaaca aatccaataa atctaagtgt agtgattggt gtattgatct tttttggaaa

70741 gggagtgtgt gtgggttgtt tatttcaaga ataggctgta tccaatcagc tgtatcccct

70801 ggatgggtgc atgatcacgc gaattacttc ttaataaatt atatgtaaga accacagcat

70861 ttcgtgatta attggtaaat ccactttgat tctctagcaa ccaataacgt ggggatgtta

70921 agatggttaa atcaaatcgt ttgaagtcta gacgcagcac ggtactcttt ctaccgctat

70981 gttaatatag aggaggtttt catttaaaag gaatattttc tcgatataga acactcatct

71041 cgataaaaaa atggaaccac tcgggtattt tcccctttta tccaatgctg aatcgacgac

71101 ctatgcctta ttgtataatt tttggatttg aactgaagaa aaaaaaaaag aaacaacttt

71161 gctgacaatt agatattttg gattttgtca gaagagtcct ctaagtattt tggttttgca

71221 ttagattcgt tttttcattt ttttttttta ctatgaagag gataggctca ttacattcat

71281 aaaaagctat gagaattgac cctaagtaat tgagcgcgag agccaaatga atcgaaagat

71341 tcatgtttgg ttcgggaagg gattatggaa gttttaaaat gaacggaaag ataatctact

71401 ttcattaagt gatttattag ataatcgaaa acagaggatc ttgaatacta ttcgaaattc

71461 agaagaactg cgtggggggg ccattgaaca gatggaaaaa gcccgggccc gcttacggaa

71521 agtagaaatg gaagcagatc agtttcgggt gaatggatac tctgagatag aacaagaaaa

71581 attgaatttg attaattcaa cttataacac tttggaacaa ttagaaaatt acaaaaatga

71641 aacgattcag tttgaacagc aaagggcaat taatcaagtc cgacaacggg ttttccaaca

71701 agccttacaa ggagctatag gaactctgaa tagttgtttg aataatgagt tacatttacg

71761 taccattagt gccaatattg gcatgttggg agcgatgaaa gaaataactg attagtcttt

71821 ctcctttcac tttatttagt attatctact ccaaggcatt attttttttt cttttcaaaa

71881 aataattaaa ttaagaaaaa ctcatggtaa ccattcaagc cgacgaaatt agtaatattc

71941 tccgtgaacg tattgaacaa tataatagag aagttaagat tgtaaatacc ggtaccgtac

72001 ttcaagtagg cgacggtatt gctcgtattt atggtcttga tgaagtaatg gcgggtgaat

72061 tagtcgaatt tgaagaaggt acaataggta ttgctctgaa tttggaatca aataatgttg

72121 gtgttgtatt aatgggtgat ggtttgatga tacaagaagg aagttctgta aaagcaacag

72181 gaagaattgc tcagatacca gtgagtgagg cttatttggg ccgtgttata aacgccctgg

72241 ctaaacctat tgacggtagg ggggaaattt cggcttctga atctcgatta attgaatctc

72301 ccgctccggg tattatttcc cggcgttccg tatacgagcc tcttcaaacc gggcttattg

72361 ctattgattc gatgatccct atagggcgtg gtcagcgaga attaattatt ggagacagac

72421 agaccggtaa aacagcagta gccacagata cgattctcaa tcaacaaggt caaaatgtaa

72481 tatgtgttta tgtagctatt gggcaaaaag catcgtctgt ggctcaggta gtaacgactt

72541 tacaggaaag gggggcgatg gaatacacta ttgtggtagc cgaaatggcg gattcccccg

72601 ctacattaca atatctcgct ccttatacag gagccgcttt ggctgaattt tttatgtacc

72661 gtaaacaaca cactttaatc atttatgatg atccctccaa acaagcccaa gcttatcgcc

72721 aaatgtctct tctattacga agaccgcccg gtcgcgaagc ttatccaggg gatgtttttt

72781 atttgcattc acgccttttg gaaagagccg ctaagtcaag ttctagttta ggcgaaggaa

72841 gtatgactgc cttaccaata gttgaaaccc aatcgggaga tgtttcggct tatattccta

72901 ctaatgtaat ttccattact gatggacaaa tatttttatc tgccgatcta ttcaatgctg

72961 gaatcagacc cgccattaat gtgggtatct ccgtttccag agtagggtct gcggctcaaa

73021 ttaaagccat gaaacaagta gctggtaaat taaaattgga attggcgcaa tttgcagaat

73081 tagaagcctt tgcacaattt gcttccgatc ttgataaagc tactcagaat caattggcaa

73141 gaggtcaacg attgcgtgaa ttgcttaaac aatcccaagc cgcccctctt gcggtagaag

73201 aacagataat gactatttat accggaacaa atggttatct tgattcatta gaaattggac

73261 aggtaaggaa atttattgtt gaattacgta attacttaaa aaccaataaa cctcagttcc

73321 aagaaatcat atcttctact aagatattta ctgaagaagc ggaagccctt ttgaaagaag

73381 ctattcagga acaaatggat cggtttctac ttcaggaaca agcataaaga aacggttcac

73441 ttttcttttc aaaaaggact caagtgtctc ggattcaaat tatgcaaaaa gtctttcgaa

73501 atcaaaaata gatggaaata aattgcgtcc aataggattt gaacctatac caaaggttta

73561 gaagacctct gtcctatcca ttagacaatg gacgccgttc attccgattt ttgcgtattt

73621 tgatttttct tgaattgaaa acaaaaaaaa taggattata tgtgagatca tttttggaat

73681 tgtatattca atgattcact aatcgtaatt aaaatagatt ttctatattc tagaatagaa

73741 ttcgaataga atatttagaa aaaaggaaat aggcgggtag cgggaatcga acccgcatcg

73801 ttagcttgga aggctagggg ttatagtcga cgttcaattc attgctttga acgtctctaa

73861 ttcaaaaccg aacatgaaac tttggtttca ttcggctcct ttatggaata tgagtaaatt

73921 catagatcta agatgtgaac aaaatgaaca aaatgatacc cataacacct acgtcagctt

73981 tttgtttgaa tacaaacaaa caaaatgaat cgctttctag atgatccttc tagaagaagg

74041 tgattctaac aatctttcta gttacttcgt tctctatttc tatttgagag gatcctaagg

74101 aaaaggattt tgtttccacc gagctaaaac aatatgtcga tgtctctagt aaaccaaagt

74161 cgtcgttgaa tagctatttt gctccaattt atttctttag aaaaaaaatg gaagatttag

74221 ttacgattcg aaatggactt tctatcttct gccatggatc ctttactcat acttattcaa

74281 ttggaatttt tggtccaatt ccaaaattat gtttcgcaat ttcataatcc aacttttcaa

74341 tttataagtg acgcatggat acaaatcacg agaattcgta tttttttctc gaatttctca

74401 ttgagaggta aaggattaaa tctttttaag aaataaagtt tttggtcgga atatgaataa

74461 aaccgaaaga ccctttaact attaacggtt aatagaacga atcacacttt taccactaaa

74521 ctatacccgc tacaatatga ttattgtata caaatggatc ttttgtcgaa caagatcgtt

74581 gagcatgagc aagataggat catcaaagaa tcatcaaaat gagaacgttg cacttttttg

74641 cggggagatc aaaatgaaaa ttgtggaaga atcgatagtt tctttttgag ttcggtaaaa

74701 tataacaaga aaaagaatgt aaatagtctt tgttcttttt ttttgttgct tgaatataaa

74761 atatccaatt gcatataata atataataac aaaagtcttt ttattttcaa atcagatatc

74821 agataaatca ttataattcg ttggaacaaa aaaaagagcc cggctaggta ctgaccgggc

74881 cgggccatga gaataagaag ggcctttcga acaaaatcaa cacaaatggg tcttgctgat

74941 tttttttagt tcgattgttc gggcggtcga gcccatcttt tagataaagg aaattcccga

75001 tttatggatc tctatatata taatagaata gagaaagaag aaagattctt tacattatgt

75061 gtaatgtagt aattatcttt ttcaattggg agagatggct gagtggacta aagcgtcgga

75121 ttgctaatcc gttgtacgag ttattcgtac cgagggttcg aatccctctc tttccgtttc

75181 cgttgatgaa tttatttggt tttttttttt caaattttaa aaatcttagt gaaacgtgta

75241 gaatagataa aatcctaaga aaatttgcaa attaactaaa accaaggaaa accccctgta

75301 ttttattctt cacgtccagg atttcgccct ggatcattag ataggaatcc aaagataaag

75361 agagacacaa aaaatatcac tacggtataa acgaagagtt tcagagtaag cattacacaa

75421 tctccaagat ggtttttttg aaaaaaaaaa gagaatagat cttctatttt tctaccacat

75481 atcctaaaga aatgaggttt ttctagaaat gcattgtcac aaattcattt gtttttcatt

75541 aacccaaatt tcggtttata tccaaattag atattatatt gtgggagacc ctaatagggt

75601 tagggtcttt cactggaaag ggggtcaaaa acgagggtaa gcctgggttt tgtcgactat

75661 acacgaattt cagcgtgtga atcgagggtt catactctaa aacttatctt attttttcaa

75721 ttgttagaat ttttttatcg aggaaatcat acattttcta ggatattatt attcaggatc

75781 tcatcgaaaa cttacagcag cttgccaaac aaaagctaag agaaaaaaaa acaaaggtat

75841 gactggcata acatccacga ttggattcaa aaaagcatag gcttcgggca atttgccgaa

75901 gaaaaaacta ctcgaataaa agggagaatt aatacaaata cagatcaaac taacgatatt

75961 aagcataaca gacattttgt tattggagat aattgcattt tgattgcgtt tttgatataa

76021 ggaaaaagaa agtaagtaag gtagacaaaa acccttcttt ttctttgaat ccacccaacc

76081 aaaaatcctc caattctcaa aggaggatag aagaaatcat actttcatac aatatcttaa

76141 ttgaattcta tgtaatgatc aataaattca gttgaattct atatctatat gtatcaaaat

76201 cctagtacca atcgattcta tagatctata gatatttgga ctcgagttga caaacaagca

76261 ataccaatat tattgtttct tagtgtcgat tagaaattga aatggggcgt ggccaagtgg

76321 taaggcaacg ggttttggtc ccgctattcg gaggttcgaa tccttccgtc ccagcgcagt

76381 ccatttttga tgctccatta ttcctataga aagaaatagg ggagtgggta ggagttaatc

76441 catattaatt tgaatatctg tgtctgttcg aatttcatta ataaatgatc aagttccata

76501 ttatatagaa atatgttttg gagctgatgt tttttctttg aatctaattt gatttaggta

76561 tttcgtgttt gactcgaatg aaaaatggga aatctattat ctatttaagg cttgaggcag

76621 gggtgattta tcctatccct ttgtattcac tttctcacaa gagtcgatat tcctcaaccc

76681 catttaaacc aaatacatat caatcatata atataatcat ataaatgtta cgtatcattc

76741 tatttcaatg acaagaaaat tttgggttct ttgtgaaaaa gatttgtaat ccttaaatta

76801 attaaactta aattatagtt atagatattc gataatctag aatatctata acagtgacaa

76861 ttgttcagtc tatctgatag atacgaagaa cctccccttt caaatttata tttgaaattc

76921 aatcagtgat gagtcaattc aaaaagaaag accgatcctc ctatgaagat aaaaggtgat

76981 gcttattgtc caattttctt caaattccat ttaataataa taatgatgta gaaataggaa

77041 accttttcaa tttcaattag aaagattcct tgtatgcgga agctttgaaa aagtaagaaa

77101 tcgtttaatc tatcctattg agtcacttga agatgcagat tcaaaaagtt attcggttct

77161 ctatttctat ttttttctcg gatctatata ttatttatgt atgttaaaaa tgctgtcttg

77221 ctaagacttt ttcagaaaaa tagttccaca tatcatatat tcatatatag aagaaaagtc

77281 tagattacga tgtctatgga cagctgaacc agtgactatt catgattcca taattgaatc

77341 aatttcatac cggttccaaa ttagaggaat gttatggtaa aacttcgttt gaaacgatgt

77401 ggtagaaagc aacgtgcgac ttgaaggaca cgatccgttg tggattttta catccaccat

77461 ttttgatttg tctaggaata agggtgctct tggctcgaca ttgtttgttc tgttacaccc

77521 gaacccttcg ttttttgttc ggttgtaaat agtgcacgct ggagctcgaa tagaaagtat

77581 taatttattt ctcggaggca aggatctagg gttaatgcca atcaattgga ggaacaactt

77641 cgtaaatata tcgaacatat ataggaatcg aaaggatcca attcgagcaa gtttccaatt

77701 caaaagcaaa acttgttgaa attgattcaa aattttcgat tcaaagtgta tcacgcggga

77761 atcgactgtc cataggattt tttgatcgaa agaaatcaaa agggggtatg ttgctgccat

77821 tttattttga aagaattaag aaacaccgaa gtaatgtcta aacccaatga ttaaaaataa

77881 ggaaaggatc taagaacaag gaaacaccct tttaattgtc tcaatcgtct caataactgg

77941 atcgcactaa agaatccaaa tagaatttga aatggtttaa acgagacaaa caaaagggag

78001 taaagacgac tcaataaatg aaattgacta aagatttttc ctttgaacta tttgagagtt

78061 atccaacttg agttatgagt acgaatggtt tatttttcat tttcaggaag aaaaaaagac

78121 tgaaatcata gtctaattta ttttatgggc gcatttgtca tttaatttat tagaattgca

78181 tatatagaca aaacttcgaa tcaaatcatt ttttcgcgag ctgtacgagg agaaaacttc

78241 ctatacggtt ctaggggggg tattgttcat ctacatctat cccaatgagc catctatcga

78301 atcgttgcaa ttgatgttcg atcccgaaga gaaggaaaag atcttagaag ggtgggcttt

78361 tatgatccaa tgaagaatca aactttttta aatgttcctc ttattctcta tttccttgaa

78421 aggggtgctc aacccacggg aactgttcag aatcttttaa agaaagccgc agtttttaag

78481 gaacttccgc ctaatcaaat gaaattcaat taaagaaata ctaggggggg tagcgacttg

78541 tatataactt tgtctaactt ttttctactt gccccccttt ttcttttttt cttccgtatt

78601 catatttatt tatcatagtt tctatcgaat cttatggtct agatggtcta gaatgaccaa

78661 attgattgat cttataaata tcaaatttcc gcatttcctt tatttaattg tgtggttttc

78721 attggaactc gactaagcaa gaacaaggaa tctactttgc ttattccact tagattgatg

78781 aaatacatta gcattaggga ctaaaaaatc cgatattttt ttttgaattc ttcctttttt

78841 attcttcgat ttatacttta ttctatctat gtagattaga tatgtagtgt caatccaaga

78901 caatttggaa tattattgta tttcattgtt aaattgaaat tcaaaggatt tcaaaaagga

78961 ttttatttca tgcaatttct cttttccaat gtattctttt ctcaacattt atattgacaa

79021 cagtgtattg aacaaatata attcatcgtg ataagcgatc cgggtctatt tatttttgtc

79081 ccatagtttt gttactttat cttattcaaa tgatgttttc tttgatacaa gaggtttttt

79141 tgattgaaag aaagctagat aacataagag atgctctatc cattttcaca atgctgtatc

79201 tttttttttc ttttatttta tctgacaatt tcttgtattt tcatctaatt gtattttcat

79261 cgaatcactt catttttctg ttttgaatcc attatcaaat ctgttttttt gttagatttg

79321 ttaactcaag ggtttgatta gtttgtataa tatatttttt tctctttgtt taaaatcaat

79381 tgaatttaat ttgattgtat ctatacaccc tttgttgagg ttttgtttaa tctatgtttg

79441 ttttttcggg ttgctaactc aacggtagag tactcggctt ttaagtgcgg ctagaatctt

79501 ttacacattt ggatgaagtg acgaattcgt ccgtaacctt ggtaaacttt ggaagaccgc

79561 gactgatcct gaaagggaat aaatggaaaa aatagcatgt cgtatcaatg gagagttctg

79621 aggatatttc attcttatcg gattggtata aaaccttttt cgaattcttg gaacggaaca

79681 aaagaaagtt gggtcgaatg aataaatgga taggagccct gtggcttcaa ttaattatca

79741 gaaagaaaaa gcaaccggct tctgttctta atttgaataa tttcccgatc taactagacg

79801 ttaaaaataa attggtgcct gatacgggaa gcacttatca ctccacgagt ggattctatt

79861 tttttttaat gaatcctaac tattgacatt ctccattatg gactgaagat gcgtgtgtaa

79921 aagaagcagt atattgataa agaaagtttt ttccgaaatc aaaagagcga ttcggtttaa

79981 aaaataaaag atttctaacc atcttgttat tctataacat aaacatagac gaattaaatg

80041 aaatggatgg aaaaaggaga gggtagagaa tctgttgata agtttatctg tccccgaggt

80101 atcgattttt acagaatacc ttgttttgac tgtatcgcac tatgtatcat ttgataaccc

80161 ccccaatctt ctacctttaa ttcaaatcga atttcaaatg gaggaaatcc aaagatattt

80221 acagctggag agatctcaac aacatgactt cctatatcca cttatctttc aggagtatat

80281 ttatgcattt gctcataatc gtgctttgag taaattgatt ttgtcggaaa atctgggtta

80341 tgacaataaa tccagtttac tgattgtgaa acggttaatt actcgactgt atcaacagaa

80401 tcattttctg atttctccta atgattctaa ccaaaatcca ttttgggtgc gcaacaagaa

80461 tttgtattct caaatcatat cagaggggtt tgcttttatt gtcgaaattc cattttcttt

80521 acaattccta tcttgtctag aaggggaaac gaacaagata ggaaaatctc agaatttacg

80581 atcaattcat tcaatatttc cctttttcga ggacactttt tcacatttta attttgtgtt

80641 agatatggta ataccccgcc ctgttcatgt ggaaatcttg gttcaaatcc ttcgctattg

80701 cgtaaaagat gcttcctcct tgcatttatt acgagtcttt ctcaatgaat attgtaattg

80761 gaatagtctt cttattccaa agaaagccag ttccccttct tcaaaaaaaa ataaaagatt

80821 attcttattc ttatataatt ctcacgtatg tgaatatgaa tccattttcg tctttctacg

80881 taaccaatct tttcatttac gatcaacatc ttctggagtt tttcttgaac gaatctattt

80941 ctatataaaa atagaacgtc ttgtgaacgt ctttgttaag attaaggatt tgggggcaaa

81001 cctgcggttg gtcaaggaac ctttcatgca ttatattagg tatcaaaaaa gatccattct

81061 ggcttcaaaa gggacattta ttttcatgaa gaaatggaaa ttttaccttg tcactttttg

81121 gcaatggcat ttttcggtgt ggtttcatcc aaaaagcatt tatataaacc aattatccaa

81181 gcattccctt gagtttttgg gctatctttc aagcgtgcga atgaaccctt ctgtagtacg

81241 cagtcaaatt ctagaaaatt catttctaat caataatgct attaataagt ttgagactct

81301 tgttccaatt attcctctga ttgcgtcatt ggctaaagcc aaattttgta acgtattggg

81361 gcatcctgtt agtaagccga ttcgggctga tttatcagat tctaatatta ttgaccgatt

81421 tgggcgtata tgcagaaata tttctcgtta tcatagtgga tcttccaaaa aaaagagttt

81481 gtatcgaata aagtatatac ttcgactttc ttgcgctaga actttggctc gaaaacacaa

81541 aagcactgta cgtgcttttt tgaaaagatt gggctcggaa tttttagaag aatttcttat

81601 gtcggaagaa gacgtccttt ttttgacctt ccaaaaaacc tcttacactt tgcgaggagt

81661 atatagaagt cggatttggt atttggatat gatttctatc aatgatctgg cgaatcacaa

81721 atccaaattc taaaatcttg aaaatatcca tttttcctaa aaaaaaaaaa aatggatcga

81781 gagagaaaaa gatccaaatt tgattcccac tattctgaaa tgttgatgta gtatgtaata

81841 agggttaaat caactgagta ttcaactttt gttttgaaag tctttctaag aaaggaaatg

81901 atgtatacat agggaaagcc gtgtgcaatg aaaaatgcaa gcacggcttg gggaggggtt

81961 tttctttatt taattaacat aacaaagaaa ttatctactc catccgacta gttccgggtt

82021 cgaatcccgg gcaacccacc accatataga aattctatgt atagcaattc atattttctt

82081 tttgtaatcc acttcattta gttataataa attcgaatag atctagatat ttattcggat

82141 tggttgacac gaacatataa gtcatgttat actgttgaat aacaagcctt ccgttttcta

82201 tttctattta tcgtatagaa aattttggtg cttgggagtc cctgatgatt aaataaacca

82261 agattttacc atgactgcaa ttttagagag acgcgaaagc gaaagcctgt ggggtcgctt

82321 ctgtaactgg ataactagca ccgaaaaccg tctttacatt ggatggtttg gtgttttgat

82381 gatccctacc ttattgaccg caacttctgt atttattatt gccttcattg ctgctcctcc

82441 agtagatatt gatggtattc gtgagcctgt ttctggatct ctactttacg gaaacaatat

82501 tatctcaggt gccattattc ctacttctgc agccatcggt ttgcactttt acccaatctg

82561 ggaagcagca tccgttgatg aatggttata caacggcggt ccttatgaac taattgttct

82621 acacttctta cttggtgtag cttgttacat gggtcgtgag tgggagctta gtttccgttt

82681 gggtatgcga ccttggattg ctgttgcata ttcagctcct gttgcagcgg ctaccgccgt

82741 tttcttgatt tacccaatcg gtcaaggaag tttttctgat ggtatgcctc taggaatttc

82801 tggtactttc aacttcatga ttgtattcca ggctgagcac aacatcctta tgcacccgtt

82861 tcacatgtta ggcgtggctg gtgtattcgg cggctcccta ttcagtgcta tgcatggttc

82921 cttggtaact tctagtttga tcagggaaac cacagaaaat gaatctgcta atgaaggtta

82981 cagattcggt caagaggaag aaacttataa tatcgtagcc gctcacggtt acttcggccg

83041 attgatcttc caatatgcta gtttcaacaa ttcccgttca ttacacttct tcctggctgc

83101 ttggcctgta gtgggtatct ggttcactgc tttaggtatt agcactatgg ctttcaacct

83161 aaatggtttc aatttcaacc aatctgtagt tgatagtcaa ggccgtgtaa ttaatacttg

83221 ggctgatatc atcaaccgtg ctaaccttgg tatggaagtt atgcatgaac gtaatgctca

83281 taatttccct ctagacctag cttctatcga agctccaaca aatggataag acttggtctt

83341 agtgtatagg agtttttgaa aatagaatag ataaatataa ggagcaataa actctttctt

83401 gttttatcaa gaggggttat tgctccttaa ttttcttttg aattactttt tttctttcca

83461 ttacaggatt cagaaaaaga aagaagaaaa aaatgattta aattcaattt attttgaatt

83521 taaaagtcaa ttcattttca ttatagtaat agtagagggg cggatgtagc caagtggatc

83581 aaggcagtgg attgtgaatc caccatgcgc gggttcaatt cccgtcgttc gcccagccca

83641 tgattggcta caaaaggatt tttttttagt gaacgtatca cagcttactc ctattttttt

83701 ttaaagacga agaaagaaat tctattttct cgcctattta ctacggcgac gaacaatcaa

83761 attttcacta tatttattcc tttttctact tcttcttcca agtgcaggat aaccccaagg

83821 ggttgtgggt ttttttctac caattggggc tcgcccttca ccacccccat ggggatggtc

83881 tacagggttc ataacgactc ctcttactac aggacgctta cctagccaac gcttggatcc

83941 ggctctaccc aaacttttct ggttcgcccc aacattcccc acttgtccga ctgttgctga

84001 gcagtttttg gatattaaac ggacctcccc agaaggtaat tttaatgtgg ccgatttccc

84061 ctcttttgca atcagtttcg ctacagcacc cgctgctcta actaattgtc caccctttcc

84121 aagtgtgatt tctatgttat gtatggccgt gcctaagggc atatcggttg aagtagattc

84181 ctctttttga tcaatcaaaa ccccttccca aactgtacaa gcttcttcca aagcatacgg

84241 ctttctggat gtagatgatg atatctatac agacggatct tatatatatg gtacaatgaa

84301 gtaccgcatg ggtggatatc tatatgaatc caaatctgcc gaatcactca tggtatgatc

84361 ttctacatcc taggttttcc cgttccatca tctggcttat gttcttcatg tagcattcag

84421 accgaatgac tctatgaaat tacgtcgata cttccacata ttatgggtaa cgtaggagac

84481 atctctattt ttcccccggg gaatctttag aattcccact gcttagcttt caattcgcct

84541 ctgaccatca aatgaaatgt gaataacccg tcctcctctc tttgaaagaa ggggcgcttc

84601 cggttctgtc ggtgcttgaa actattttgt cttctccata ttactatatc tctagagtca

84661 ataattttat atgaggaact gctgaactca atcacttgct gccgttactc ttcagttttc

84721 tgttgaggtc tatcctgtag aggtactcaa attggatcag tgatcgattt ctaggtttcg

84781 tcgtaaacct aattggttac ttccaattac gtaaatcaat agttcaaacc gcactcaaag

84841 gtagggcatt tcccattttt atcggaactt ctgtaccaga aacaatggta tctccaatta

84901 tagcccctct gggatgtaaa atatatctct tctcaccatc cccatagtgt atgagacaaa

84961 tgtatgcatt tcgattaggg tcgtattcta tggttacgat tctaccatat atgtcttttt

85021 cattccgtcg aaaatcgatt ttacggtata gacgcttatg acctccccct ctatgccttg

85081 aggtaatgat tcctctggca ttacgacctt taccacaatg atgctgccca tagatcaaat

85141 tatttcgtgg attggatttc acttgactgt ctacggttcc attgcgtgtg ctcggggtag

85201 aagttttgta taaatgtatc gccatgctat taagtatttt gatttaagtt cttttctttc

85261 taagaggtgg aatagaataa cccggttgaa gcgtaatgat catacgtctg taatgcattg

85321 tatgtcccat aatgggtccc attcttctac cctttcccgg aagtcgatga ctattcatag

85381 ctattaccct gacaccaaag aagagttcga cccaatgctt tagttctgtc ctagttgatc

85441 ctgattcgac attagaagta tattgatttt tccccaataa ccgaatactt ttgtctgtaa

85501 atactgcatg tttgattcca tccataaatc gaatttcttc cctatgagtt ctagtctcaa

85561 taagaatgct atttcttact gttcatatac tatgatatga atatactaca ccaattcgtt

85621 atgtatggat gatgagattc cattgataca gagccaattc caatagactt attggagggt

85681 tccattggcg tgcatccagt aggaattgaa cctacgaatt cgccaattat gagttgggcg

85741 ctttaaccat tcagccatgg atgcttaggg gggatcctcg tacatggtga ataaccaaat

85801 tccaattgaa atgaaatctt taggataaat caatgcaatt taggaggaat caatgaaagg

85861 acatcaattc caatcctgga ttttcgaatt gagagagatc aagaattctc actgtttctt

85921 agattcatgg acccaattca attcagtggg gtctttcatt cgcatttttt tccaccaaga

85981 acgttttcta aaactctttg acccccgaat tttgagtatc ctactttcac gcaattcaca

86041 gggttcaaca agcaatcgat atttcacgat caagggtgta ctactatttg tagtagcggt

86101 ccttatatat cgtattaaca atcgaaatat ggtcgaaaga aaaaatctct atttgagggg

86161 gcttcttcct atacctatga attccattgg acccataaat gatacattgg aagaatcggt

86221 tgggtcttcc aatatcaata ggttgattgt ttcgctcctg tatcttccaa aaggaaaaaa

86281 gatctctgag agttgtttcc tgaatccgaa agagagtact tgggttctcc caataactaa

86341 aaagtgtagc atgcctgaat ctaactgggg ttcgcggtgg tggaggaact ggatcggaaa

86401 aaagagggat tctagttgta agatatctaa tgaaaccgtc actggaattg ggatcttatt

86461 caaagagaaa gatctcaaat atctggagtt tctttttgta tattatatgg atgatccgat

86521 ccgcaaggac catgattggg aattgtttga tcgtctttct ctgaggaaga ggcgaaatag

86581 aatcaacttg aattcgggcc cgctattcga aatcttagtg aaacactgga tttcttatct

86641 catgtctgct tttcgtgaaa aaataccaat tgaagtggag ggtttcttca aacaacaaag

86701 ggctgggtca actattcaat caaatgatat tgagcatgtt tcccatctcc tctcgagaaa

86761 caagcgggct atttctttgc aaaattgtgc tcaatttcat atgtggcaat ttcgccaaga

86821 tctcttcgtt agttggggga agaatccgca cgaatcggat tttttgagga acgtatcgag

86881 agagaattgg atttggttag acaatgtgtg gttggtaaac aaggatcggt tttttagaaa

86941 ggtacggaat gtatcgtcaa atattcaata tgattccaca agatccagtt tcgttcaagt

87001 aacggattct agccaactga aaggatcttc tgatcaatcc agagatcatt tggattccat

87061 tagtaatgag gattcggaat atcacacatt gatcaatcaa agagagattc aaccactaaa

87121 agaaagatcg attctttggg atccttcctt tcttcaaacg gaaggaacag agatagaatc

87181 agaccgattc ccgaaatgcc tttctggata ttcctcaatg tcccggctat tcacggaacg

87241 tgagaagcag atgattaatc atctgcttcc ggaagaaatc caagaatttc ttgggaatcc

87301 tacaagatcg gttcgttctt ttttctctga tagatggtca gaacttcatc tgggttcgaa

87361 tcctactgag aggtccacta gagatccgaa attgttgaag aaacaacaag atctttcttt

87421 tgtccctccc aggcgatcgg aaaataaaga actggttaat atattcaaga taattacgta

87481 tttacaaaat actgtctcaa ttcatcctat ttcatcagat ccggggtgtg atagggttct

87541 gaaggatgaa ccggatatgg acagttccaa taagatttca ttcttgaaca aaaatccatt

87601 ttttgattta tttcatctat tccatgaccg gaacagggga ggatacacgt tacaccacga

87661 ttttgaatca gaagagagat ttcaagaaat ggcagatcta ttcactctat caataaccga

87721 gccggatctg gtgtatcata agggatttcc cttttctatt gattcctacg gattggatca

87781 aaaacaattc ttgaatgagg ccagggatga atcgaaaaag aaatctttat tggttctacc

87841 tcctattttt tatgaagaga atgaatcttt ttctcgaagg atcagaaaaa aatgggtccg

87901 gatctcttgc gggaatgatt tggaagatcc aaaaccaaaa atagtggtat ttgctagcaa

87961 caacataatg gaggcagtca atcaatatag attgatccga aatctgattc aaatccaata

88021 tagtacctat gggtacataa gaaatgtatg gaatcgattc tttttaatga atcgatccga

88081 tcgcaacttc gaatatggaa ttcaaaggga tcaaatagga aaggatactc tgaatcatag

88141 aactataatg aaatatacga tcaacgcaca tttatcgaat ttgaaaaaga gtcagaagaa

88201 atggttcgat cctcttatct tgatttctcg aaccgagaga tccatgaatc gggctcctga

88261 tgcatataga tacaaatggt ccaacgggag caagaatttc caggaacatt tggaacattt

88321 cgtttctgag cagaagagcc gttttcaaat agtgttcgat cgattacgta ttaatcaata

88381 ttcgattgat tggtctgagg ttatcgacaa aaaagatttg tctaagccac ttcgtttctt

88441 tttgtccaag tcacttcttt ttttgtccaa gttgcttttc tttttgtcga actcacttcc

88501 ttttttctgt gtgagtttcg ggaatatccc cattcatagg tccgagatct acatctatga

88561 attgaaaggt ccaaatgatc aactctgcaa tcagttgtta gaatcaatag gtcttcaaat

88621 tgttcatttg aaaaaatgga aacccttctt attggatgag catgatactt cccgaaaatc

88681 gaaattcttg atcaatggag gaacgccctt tttgttcaat aagataccaa agtggatgat

88741 tgactcattc catactagaa ataatcgcag gaaatccttt gataacgcgg attcctattt

88801 ctcaatgata ttccacaatc aagacaattg gctgaatccc gtgaaaccat ttcatataag

88861 ttcattgata tcttcttttt ataaagcaaa tcgacttcga ttcttgaata atccacatca

88921 cttctgcttc tactgtaaca caagattccc cttttctgtg gaaaaggccc gtatcaataa

88981 ttatgatttt acgtatggac aattcctcaa tatcttgttc attcgcaaca aaatattttc

89041 tttgtgcgtc ggtaaaaaaa aacatgcttt tggggggaga gatactattt caccaatcga

89101 gtcacaggta tctaacatat tcatacctaa cgattttcca caaagtggtg acgaaacgta

89161 taacttgtac aaatctttcc attttccaag tcaacacgat ccattcgttc gtagaactat

89221 ttactcgatc gcagacatgt ttggaacacc tctaacagag ggacaaatag tccattttga

89281 aagaacttat tgtcaacctc tttcagatat gaatctatct gattcagaag ggaagaactt

89341 gcatcagtat ctcaattcaa acgtgggttt gattcacact ccatgttctg agaaatattt

89401 accatccgaa aagaggaaaa agcggagtct ttgtctaaag aaatgcgttg agaaagggca

89461 gatgtataga acctttcaac gagatggtgc tttttcaact ctctcaaaat ggaatctatt

89521 ccaaacatat ataccatggt tccttacttc gacagggtac aaatatctaa atttgatatt

89581 tttagatact ttttcagacc tattgccgat actaagtagc agtcaaaaat ttgtatccat

89641 ttttcatgat attatgcatg gatccggtat agcatggcga attcttcaga aaaaatggtg

89701 tcttccacaa tggaatctga taagtgcgat ttcgagtaag tgtttccata atcttcttct

89761 gtccgaagaa atgattcatc gaaataatga gtcaccatcg acacatctga gatcgccaaa

89821 tgttcgggag ttcctctatt caatcctttt ccttcttctt gttgctggat atctcgttcg

89881 tacacatctt ctctttgttt cccgggcctc tagtgagtta cagacagagt tcgaaaaggt

89941 caaatctttg atgattccat catctatgat tgagttacga aaacttctgg ataggtatcc

90001 tacatctgca ccgaattctt tctggttaaa gaatctcttt ctagttgctc tggaacaatt

90061 aggagattct ctagaagaaa tacgggcttc tggcggcaac atgcctggtc ccgcttatgg

90121 ggtcaaatca atacgttcta agaagaaata tttgagtatc aatctcatcg atctcatacc

90181 aaatcccatc aatcgaatca ctttttcgag aaatacgaga catctaagtc atacaagtaa

90241 agagatctat tcattgataa gaaaaagaaa aaacgtgaat ggggattgga ttgatgataa

90301 aatcgaatcc tgggtcgcga acagtgattc gattgatgat gaagaaagag aattcttggt

90361 tcagttctcc gccttaacga cagaaaaaag gattgatcaa attctattga gtctgactca

90421 tagtgatcat ttatcaaaga atgactctgg ttatcaaatg attgaacaac cgggagcaat

90481 ttacttacga tacttagttg acattcataa aaagtatcta ttgaattatg agttcaatac

90541 atcctcttta gcagaaagac gggtattcct tgctcattat cagacaatca cttattcaca

90601 aacttcgtgt gggactaata ctttgcattt cccatctcat ggaaaaccct tttcgctccg

90661 cttagcctta tccccctcta gggggatttt agtgataggt tctataggaa ctggacgatc

90721 ctatttggtc aaatacctag caacaaactc ctatgttcct ttcattacgg tatttctgaa

90781 caagttcctg gataacaagc ctaaaggttt tctttttgat gatatcgata ttgatgctag

90841 tgacgatatt gatgctagtg acgatattga tgctagtgac gatattgatg ctagtgacga

90901 tattgatcgt gaccttgata cggagctgga actgctaact atggatagga taccggaaat

90961 agaccgattt tatatcaccc ttcaattcga attggcaaaa gcaatatctc cttgcataat

91021 atggattcca aacattcatg atctggatgt gaatgagtcg aattacttat ccctcggtct

91081 attagtgaac catctctctg aaagatgttc cactagaaat attcttgtta ttgcttcgac

91141 tcatattccc caaaaagtgg atcccgctct aatagctccg aataaattaa atacgtgcat

91201 taagatacga aggcttctta ttccacaaca acgaaagcac tttttcactc tttcatatac

91261 taagggattt cacttggaaa agaaaatgtt ccatactaac ggattcgggt ccataaccat

91321 gggttccaat gcaagagatc ttgtagcact taccaatgag gccctatcga ttagtattac

91381 acagaagaaa tcaattatag acactaagac aattagatcc gctcttcata gacaaacttg

91441 ggatttgcga tcccaggtaa gatcggttca ggatcatggg atccttttct atcagatagg

91501 aagggctgta gcacaaaatg tacttctaag taattgcccc atagatccta tatctatcta

91561 tatgaagaag aaatcatgta acgaagggga ttcttatttg tacaaatggt acttcgaact

91621 tggaacgagc atgaagaaat taacgatact tctttatctt ttgagttgtt ctgccggatc

91681 ggtcgctcaa gatctttggt ctctacccgg atccgatgaa aaaaatggga tcacttctta

91741 tggactcgtt gagaatgatt cggatctagt tcatggccta ttagaagtag aaggcgctct

91801 ggtgggatct tcacggacag aaaaagattg cagtccgttt gataatgatc gagttacatt

91861 gcttcttcgg cccgaaccga ggaatccctt agatatgatg caaaatggat cttgttctat

91921 ctttgatcag agatttctct atgaaaaata cgaatcggag tttgaagaag ggaaggggga

91981 aggagccctc gacccgcaac agatagagga ggatttattc aatcacatag tttgggctcc

92041 tagaatatgg cgcccttggg cctttctatt tgattgtatc gaaaggccca atgaattggg

92101 atttccctat tggtccaggt catttcgggg caagcggatc atttatgatg aagaggatga

92161 gcttcaagag aatgattcgg agttcttgca gagtggaacc atgcagtacc agacacgaga

92221 tagatcttcc aaagaacaag gcctttttca aataagccaa ttcatttggg accctgcgga

92281 tccgctcttt ttcctattca aagatcagcc ccctggctct gtgttttcac atcgagaatt

92341 atttgcagat gaagagatgt caaaggggct tcttacttcc caaatggatc ctcctacatc

92401 tatatataaa cgctggttta tcaagaatac acaagaaaag cacttcgaat tgttgattaa

92461 tcgtcagaga tggcttagaa ccaatagttc attatctaat ggatctttcc gttctaatac

92521 tctatccgag agttatcagt atttatcaac tctgttccta tctaacggaa cgctattgga

92581 tcaaatgaca aagacattgt taagaaaaag atggcttttc ccggatgaaa tgaaaattgg

92641 attcatggaa caggagaaag atttcccatt ccttagccgg aaagatatgt ggctatgaaa

92701 gagggattca gtggaacaga atggactggg tggtagagtg gtggaaacgc ttctttcttc

92761 catattttgg accttagctc catggaacaa tatgttactg ctgaaacacg gaagaattta

92821 aatcttagat caaaacacta tgtatggatg gtatgaactg cccaaacaag aattcttgaa

92881 cagcgaacaa ccagttcaga tattcacgac caagaagtac tggattctct ttcggatagg

92941 ccctgaaagg agaaggaagg gtggaatgcc aacaggcgtc tattattgaa ttgacccgac

93001 ccgatagtac cccttttggg cacgtccagt gccaaagtca ctgaatgggt aagtcgtcaa

93061 tccctggact atgtaatgta ctttatccgc tgggtgactg gcgggcattt taccagaggt

93121 ttctaatcta cccttgtgtg attcctgttg aatcgtatac tctggggcgg gtgcaggtcg

93181 gtatatcaat accgattcga tccgagctct cttattgaat tgcttattca atgagcattc

93241 tcaatattat gccttgaaga ggactcgaac ctccacgctc tttagcacga gattttgagt

93301 ctcgcgtgtc taccatttca ccaccaaggc atcttgaaag tgcatcctat tccatgaata

93361 tgatatctat ctagtgtgat gtatggaata tatgacaagg gtgtagtatt tctattgatc

93421 ggtcatatag gcccgagttg gacatccaat tgcttcgatt tgaattatcc ggagaatgcc

93481 ttatgtatat atcaaaaaaa aaagatggcc aatcaaacct atttgatttc tcgattcaat

93541 agaagctcaa aggggtgaat agggtcccaa aaataacgag aaatatgggg aaaaagcagg

93601 tccgattacg cctattccta atcctaaatg gaacggcgta gggacccata tgtaaacata

93661 gtatctattt agatacgctc gaatgacccc ttgagaatgt atataaccct attccggccc

93721 ggtccggtat ggaatgaact tataatcatg gaatcgactc gatcatcaga ttataagttc

93781 ataaccccag cccattcccg ttttggtcgg aacagatcta ctaattcttt gattccagtt

93841 agtaagaggg atcttgaact aagaaataga ccctagaagc taaaaaaggg tatcctgagc

93901 aattgcaata atcgggttca ttgatattcc tggtatagta gatgctatca cacatacaat

93961 catactcaat tcgacggaat tgtttgatct taaaggagat cttctataat ttcgcacgtg

94021 aggggtgatt tcttggtttc gtccagtcat taataacttg attattttta gataatagta

94081 gatagaaaca acgcttgtaa ggagtcctat taaaaccaag gaatataggc ctgcctgcca

94141 tccacaccag aataaataga gttttccgaa aaaacctgct agtggaggaa gacctcctag

94201 ggataagaga catagggcta aagagagagc caaaaaagga tctttcgtgt ataatcctgc

94261 ataatctcga atgttatcag ttccggtacg tagaccaaat aatacaatgc aagcaaaagt

94321 tcctagattc atggagatat agaacagcat ataagttatc atgcttgcat atccatcatt

94381 tgagtctcca acaattattc caataattac atatccgatt tgacctatgg acgaatatgc

94441 aagcatacgt ttcatgcttg tttgagtaat agcaatgata tttcccaata tcatgctaag

94501 aatagctagg atttccagaa gaagatgcca ttcgtttgat gagaaataaa aaggaatatc

94561 caaaattcga gtggctgaag ctgaagcagc tactttcgaa gtaacagaaa gaaaagcaac

94621 gactggagtg ggagagtcag agtcgaaaag aggattcctc acttctttct ctcattcaaa

94681 accgtgcatg agactttcat ctcacacggc tcctaagtga taaaagaaag aagaactcgt

94741 tttctttctt ttttgattac cttcctcgcg tatgtataag accgaatcca ttcgatttct

94801 aaaaaagatt actaatcctt aacttttcga ggaatccttc atcagtggtt gtgaatgact

94861 tattttttca atcttttcaa ccttggttcc gtaggagcaa gtcagaaaga ttgagaaata

94921 gaaccatctg atttaattcg ttctcaatag ccatgaaatg atcatcttag ggtgatcctt

94981 ttgtcgacgg atgctcctat tacactcgta gtctctgaag gatgagaacc aactatgtag

95041 catctacatc gagaattcaa gtattgtata cgtcattagt cggatccttt gtaggaacta

95101 cccgtaataa cgaacttgca aaatggatct gtttatcata aagagattcg tcgttcctga

95161 ccctgcttca ccttaattgt tatttgaaca agtaaaagtt ctgtcttggt ccgagtgggg

95221 atagcatttc tcttctgcat gtccatggag ttttgaaaaa tccaaacatc tcagagatag

95281 atagagaggt aggaatttct cgaacgaacc gcactccttc gtatacgtca ggagtccatt

95341 gatgagaagg ggctggggaa agcttgaacc caattcctac agtgatgaat atgagcgcaa

95401 ttgaaattcc cggggagtta tacatttgtg tattgataag accattcact atttcttgaa

95461 gctcggtctc tcccccggat aaaccatata gccaagagaa accatgaacc agaatagaag

95521 agcttgcccc acccatgagt aaatatttca tagtagcctc attagaccgt acatctttct

95581 tggtatatcc agataatagg taggagcata aactgaaaca ttctggagct acaaagatag

95641 ttattaaatc gttagcaccg cataaaaaca ttcctcctag agtagctgtt aatacgaata

95701 agagaaactc tgttatagcc atttctgtac attcaatgta ctctacggat agaggaatac

95761 atagagttga acatagtaaa ataagaaatt gaaagatttc gttgaaattg ttcgtttgga

95821 aatttcccga aaagctaatt ataggttctt ctctccatcg gaacaatagg gccgttatgc

95881 tcattactaa acttgttgaa gagatgaaat ataaccaagg tatatctttt tgatcagagg

95941 ttgaatcgat catcagaaga agaattaggc caaaaattag gatacattct gggaaaataa

96001 aacttccatc gaagagaagc aaatgaaagg ctttcataaa aattctcgta gaatcgagaa

96061 tgaagttttc attctgtaca tgccagatca tgaattagta actgcatcca atttccaaaa

96121 aaaatcccaa ttgtgtcgaa ctttccgttt ttggaatgga ataggatcaa gatcaaacct

96181 tattccatgg tatttacatg aggttcctct ttaagaaagt ccccgagagg gcttagttga

96241 tccatgattt ttgtttcatc tttcgtttcc ttttcgtttg tttcgagaaa tctatcgatc

96301 aattccgatt ctttcttttt ctcttgattc ttttccgatc gagatgtata gatcctgttc

96361 atggattaac gaaaatgtgc aaaagctcta tttgcctctg ccattttatg agtctcttcc

96421 tttttgcgta tggcatcgcc actccctttg gcagcatcca ctaattcgga acttaatttg

96481 aaagccatat ttcgacccgg acgttttcgg gatgccgcta ataaccaacg aatggcaagt

96541 gcttttcctt gtgtggatcc tatttcaatg ggaacttgat gagtcgatcc acctacacgt

96601 cttgctttta ctgctatatc gggagttact ccacgtattg cttgacgtaa aacagatagt

96661 ggatttgttt ctgtcttttg ttgaattttt ttcatggctc gatagataat ttgataagcc

96721 aatgattttt ttccgtgttt cagaatacgg ttaaccaaca tgttaactaa tcgattacga

96781 taaattggat cggattttgc tgttttttct tctgcagtac ctcgacgtga catgagcgtg

96841 aaaggggttt aagaatcagt tttcttttta taagggctaa aatcacttat tttggctttt

96901 ttaccccata ttgtagggtg gatctcgaaa gatatgaaag atctccctcc aagccgtaca

96961 tacgactttc atcgaatacg gctttccgca gaattctata tgtatctatg agatcgagta

97021 tggaattctg tttactcact ttaaattgag tatccgtttc cccccctttc ctgctaggat

97081 tggaaatcct gtattttaca tatccatacg attgagtcct tgggtttccg aaatagtgta

97141 aaaagaagtg cttcgaatca ttgctatttg actcggacct gttctaaaaa agtcgaggta

97201 tttcgaattg tttgttgaca cggacaaagt cagggaaaac ctctgaaatt atttcaatat

97261 tgaaccttgg acatataaga gttccgaatc gaatctcttt agaaagaaga tcttttgtct

97321 catggtagcc tgctccagtc cccttacgaa actttcgtta ttgggttagc catacacttc

97381 acatgtttct agcgattcac atggcatcat caaatgatac aagtcttgga taagaatcta

97441 caacgcacta gaacgccctt gttgacgatc ctttactccg acagcatcta gggttcctcg

97501 aacaatgtga tatcttacac cgggtaaatc cttaaccctt ccccctctta ctaagactga

97561 agaatgttct tgtgaattat ggccaatacc gggtatataa gcagtgattt caaatccaga

97621 ggttaatcgt actctggcaa ctttacgtaa ggcagagttt ggttttttgg gggtgatggt

97681 ggaaaagttg acagataagt cacccttact gccactctac agaaccgtac atgagatttt

97741 cacctcatac ggctcctcgt tcaattcttt cgaattcatt ggatcccttt ccgcgctcga

97801 gaatcccctc ccctcttcca ctctgtcccg aatagtaact aggacccatt tagtcacgtt

97861 ttcatgttcc aattgaacac tttccatttt tgattattct caaaggagaa gattattctc

97921 tttaccaaac atatgcggat ccaatcacga tcctataata agaacaagag atctttctcg

97981 accaaccccc ctgcccctca ttcttcgaga atcagaaagg tcctttcctt ttcaagtttg

98041 aatttgttca tttggaatct gggttcttct acttcatttt tagttaatat tcatttcttt

98101 ttccctctgt tttttttata tcattcctta agtcccatag gtttgatcct gtagaatttg

98161 accattgaac gaagggtacg aaataaatct gattgatttt tcgatcaaaa gtactatgtg

98221 aaatcttcgg ttttttcctc ttcctctatc cctatcccat aggtacagaa tcaatagaga

98281 accttttctt ctgtatgaat cgatcttatt ccattccaat tccttcccga tacctcccca

98341 ggaaaatatc gaattggatc ccaaattgac gggttagtgt aagcttatcc atgcagttat

98401 gcactcttct cgaataggaa tccgttttct gaaagatcct ggctttcgta ctttggtggg

98461 tctccgagat cctttcgatg acctatgttg aagggatatc tatctaatcc gatcgattgc

98521 gtaaagcccg cgatagcaac ggaaccgagg aaagtatact gaaaagacag ttcttttcta

98581 ttatattagt attttcgatt atattagatt agtattagtt agtgatttag attagttagt

98641 gatcccggct tagtgagtcc tttcttccgt gatgaactgt tggcaccagt actacatttt

98701 atctctgtgg gccgaggaga aaaggggctc ggcgtgtaca tgagagaagc aaggaggtca

98761 acctctttca aatagacaac atggattctg gcaatgcagt tggactctca tgtcgatcca

98821 aacgaatcat cctttccacg caggtaaatc ttttctttgc ctgctaggca agagactagc

98881 aaattaaaaa ttctgtctcg gtaggacatg tatttctatt actatgaaat tcataaatga

98941 agtagttaat ggtggggtta ccattatcct ttttgtagtg acgaatcttg tatgtgttct

99001 taagaaaagg aaaaaaggaa tttgtccatt tttcggggtc tcaaaggggc gtggaaacac

99061 ataagaactc ttgaatggaa atggaaaaag gatgtaactc cagttccttc ggaatcgcta

99121 gtcaatccta ggggcagttg acaattgaat cctattttga ccattatttt catatccgaa

99181 atagtgcgaa aagaagaccc ggctctaagt tgttcaagat caagaatagc ggcgttgagt

99241 ctctcgaccc tttgacttag gattagtcag ttctatttct cgatgggggc agggaaggga

99301 tataactcag cggtagagtg tcaccttgac gtggtggaag tcatcagttc gagcctgatt

99361 atccctaaac ccaatgtgag tttttctgtt tggatttgcc cccccgccgt gattcaatga

99421 gaatggataa gaggctcgtg ggattgacgt gagggggcag ggatggctat atttctggga

99481 gcgaactccg ggcgaatatg aagcgcatga atacaagtta tgccttcgaa tgaaagacaa

99541 ttccgaatcc gctttgtcta cgaacaagga agctataagt aatgcaacta tgaatctcat

99601 ggagagttcg atcctggctc aggatgaacg ctggcggcat gcttaacaca tgcaagtcgg

99661 acgggaagtg gtgtttccag tggcggacgg gtgagtaacg cgtaagaacc tgcccttggg

99721 aggggaacaa cagctggaaa cggctgctaa taccccgtag gctgaggagc aaaaggagga

99781 atccgcccga ggaggggctt gcgtctgatt agctagttgg tgaggcaata gcttaccaag

99841 gcgatgatca gtagctggtc cgagaggatg atcagccaca ctgggactga gacacggccc

99901 agactcctac gggaggcagc agtggggaat tttccgcaat gggcgaaagc ctgacggagc

99961 aatgccgcgt ggaggtagaa ggcccacggg tcgtgaactt cttttcccgg agaagaagca

100021 atgacggtat ctggggaata agcatcggct aactctgtgc cagcagccgc ggtaagacag

100081 aggatgcaag cgttatccgg aatgattggg cgtaaagcgt ctgtaggtgg ctttttaagt

100141 ccgccgtcaa atcccagggc tcaaccctgg acaggcggtg gaaactacca agctggagta

100201 cggtaggggc agagggaatt tccggtggag cggtgaaatg cgtagagatc ggaaagaaca

100261 ccaacggcga aagcactctg ctgggccgac actgacactg agagacgaaa gctaggggag

100321 cgaatgggat tagatacccc agtagtccta gccgtaaacg atggatacta ggcgctgtgc

100381 gtatcgaccc gtgcagtgct gtagctaacg cgttaagtat cccgcctggg gagtacgttc

100441 gcaagaatga aactcaaagg aattgacggg ggcccgcaca agcggtggag catgtggttt

100501 aattcgatgc aaagcgaaga accttaccag ggcttgacat gccgcgaatc ctcttgaaag

100561 agaggggtgc cttcgggaac gcggacacag gtggtgcatg gctgtcgtca gctcgtgccg

100621 taaggtgttg ggttaagtcc cgcaacgagc gcaaccctcg tgtttagttg ccatcgttga

100681 atttggaacc ctgaacagac tgccggtgat aagccggagg aaggtgagga tgacgtcaag

100741 tcatcatgcc ccttatgccc tgggcgacac acgtgctaca atggccggga caaagggtcg

100801 cgatcccgcg agggtgagct aaccccaaaa acccgtcctc agttcggatt gcaggctgca

100861 actcgcctgc atgaagccgg aatcgctagt aatcgccggt cagccatacg gcggtgaatt

100921 cgttcccggg ccttgtacac accgcccgtc acactatggg agctggccat gcccgaagtc

100981 gttaccttaa ccgcaagggg ggggatgccg aaggcagggc tagtgactgg agtgaagtcg

101041 taacaaggta gccgtactgg aaggtgcggc tggatcacct ccttttcggg gagagctaat

101101 gcttgttggg tattttggtt tgccactgct tcacacccaa aacaaaaaga agggagctgc

101161 gtctgagtta aaggaagtct tctttcgttt ctcgacggtg aagtaagacc aagcccatga

101221 gcttattatc ctaggtcgga acaagttgat aggatcccct ttgttacgtc cccatgcccc

101281 ccgtgtgggg gcatgggggc gaaaaaagga aagagaggga tggggtttct ctcgcttttg

101341 gcatagcggg ccccctgtgg gaggctcgca cgacgggcta ttagctcagt ggtagagcgc

101401 gcccctgata attgcgtcgt tgtgcctggg ctgtgagggc tctcagccac atggatagtt

101461 caatgtgctc atcggcgcct gaccctgaga tgtggatcat ccaaggcaca ttagcatggc

101521 gtactcctcc tgttcgaaca ggggtttgaa accaaacctc tcctcaggag gatcgatggg

101581 gcgattcggg tgagatccaa tgtagatcca actttcgatt cactcgtggg atccgggcgg

101641 tccggggggg gaccaccgcg gctcctctct tctcgagaat ccatacatcc cttatcagtg

101701 tatggacagc tatctctcga gcacaggttt aggttcggcc tcaatgggaa aagaaaatgg

101761 agcacctaac aacgcatctt cacagaccaa gaactacgag atcaccccct tcattctggg

101821 gtgacggagg gatcgtacca ttcgagccgt ttttttcatg cttttcccgg aggtctggag

101881 aaagctgcat ttccctaatc ctcccttccc gaaaggaaga gcgtgaaatt ctttttcctt

101941 tccgcaggga ccaggagatt ggatctagcc ataagaagaa tgcttggtat aaataactca

102001 cttcttggtc ttcgaccccc tcagtcacta cgaacgcccc cgatcagtgc aatgggatgt

102061 gtctatttat ctatctcttg attcgaaatg ggagcaggtt tgaaaaagga tcttagagtg

102121 tctagggttg ggccgggagg gtctcttaac gccttctttt ttcttctcat cagagttatt

102181 tcacaaagac ttgccggggt aaggaagaag gggcgaacaa gcacacttgg agagcgcagt

102241 acaacggaga gttgtatgct gcgttcggga aggatgaatc gctaccgaaa aggaatctat

102301 tgattctctc ccaattggtt ggaccgtagg tgcgatgatt tacttcacgg gcgaggtctc

102361 tggttcaagt ccaggatggc ccagctgcgc cagggaaaag aatagaagaa gcatctgact

102421 acttcatgca tgctccactt ggctcggggg gatatagctc agttggtaga gctccgctct

102481 tgcaattggg tcgttgcgat tacgggttgg atgtctaatt gtccaggcag taatgatagt

102541 atcttgtacc tgaaccggtg gctcactttt tctaagtaat ggggaagagg accgaaacat

102601 gccactgaaa gactctactg agacaaagat gggctgtcaa gaacgtagag gaggtaggat

102661 gggcagttgg tcagatctag tatggatcat acatggacgg tcgttggagt cggcggctcc

102721 cccagggtcc ctcatctgag atccctgggg aagaggatca agttggccct tgcgaacagc

102781 ttgatgcact atctcccttc aaccctttga gcgaaatgcg gcaaaagaaa aggaaggaaa

102841 atccatggac cgaccccatc atctccaccc cgtaggaact acgagatcac cccaagggcg

102901 ccttcggcat ccaggggtca cggaccgacc atagaaccct gttcaataag tggaacgcat

102961 tagctgtccg ttctcaggtt gggcagtaag ggtcggagaa gggcaatcac tcattcttaa

103021 aaccagcgtt cttaagacca aagagtcggg cggaaaaagg ggggctctcc gttcctggtt

103081 ctcctgtagc tggaacctcc ggaaccgcaa gaatcctgag ttagaatggg attccaactc

103141 agcacccttt gagtgagatt ttgagaagag ttgctctttg gagagcacag tacgatgaaa

103201 gttgtaagct gtgttcggag ggggttattg tctatcgttg gcctctatgg tagaatcagt

103261 cgggggcatg agagacggtg gtttaccctg cggcggatgt cagcggttcg agtccgctta

103321 tctccaactc atgaacttag ccgatacaaa gctatatgat agcacccaat ttttccgatt

103381 cggcggttcg atctatgatt tctcattcat ggacgttgat aagatccatc catttagcag

103441 caccttagga tggcatagcc ttatttataa ttgattttat aatttataag atttttataa

103501 gggcaataag ggcgaggttc aaacgaggaa aggcttacgg tggataccta ggcacccaga

103561 gacgaggaag ggcgtagtaa tcgacgaaat gcttcgggga gttgaaaata agcatagatc

103621 cggagattcc cgaatagggc aacctttcaa actgctgctg aatccatggg caggcaagag

103681 acaacctggc gaactgaaac atcttagtag ccagaggaaa agaaagcaaa agcgattccc

103741 gtagtagcgg cgagcgaaat gggagcagcc taaaccgtga aaccggggtt gtgggagagc

103801 aatacaagcg tcgtgctgct aggcgaagca gcataaatgc ggcaccctag atggcgaaag

103861 tccagtagcc gaaagcatca ctagcttacg ctctgacccg agtagcatgg gacacgtgga

103921 atcccgtgtg aatcagcaag gaccaccttg caaggctaaa tactcctggg tgaccgatag

103981 cgaagtagta ccgtgaggga agggtgaaaa gaacccccat cggggagtga aatagaacat

104041 gaaaccgtaa gctcccaagc agtgggagga gccatggctc tgaccgcgtg cctgttgaag

104101 aatgagccgg cgactcatag gcagtggctt ggttaaggga acccaccgga gccgcagcga

104161 aagcgagtct tcatagggca atggtcactg cttatggacc cgaacctggg tgatctatcc

104221 atgaccagga tgaagcttgg gtgaaactaa gtggaggtcc gaaccgactg atgttgaaga

104281 atcagcggat gagttgtggt taggggtgaa atgccactcg aacccagagc tagctggttc

104341 tccccgaaat gcgttgaggc gcagcagttg actggacatc taggggtaaa gcactgtttc

104401 ggtgcgggcc gcgagagcgg taccaaatcg aggcaaactc tgaatactag atatgacctc

104461 aaaataacag gggtcaaagt cgaccagtga gacgatgggg gataagcttc atcgtcgaga

104521 gggaaacagc ccggatcacc agctaaggcc cctaaatgat cgctcagtga taaaggaggt

104581 aggggtgcag agacagccag gaggtttgcc tagaagcagc cacccttgaa agagtgcgta

104641 atagctcact gatcgagcgc tcttgcgccg aagatgaacg gggctaagcg atctgccgaa

104701 gctgtgggat gtaaaaatac atcggtaggg gagcgttccg ccttagaggg aagtttccgc

104761 gcgagcggcg gtggacgaag cggaagcgag aatgtcggct tgagtaacgc aaacattggt

104821 gagaatccaa tgccccgaaa acctaagggt tcctccgcaa ggttcgtcca cgtagggtga

104881 gtcagggcct aagatcaggc cgaaaggcgt agtcgatgga caacaggtga atattcctgt

104941 actacccctt gttggtcccg agggacggag caggctaggt tagccgaaag atggttatcg

105001 gttcaagaac gtaaggtgtc cctgcttttt cagggtaaga aggggtagag aaaatgcctc

105061 gagccaatgt tcgagcacca ggcgctacgg cgctgaagta acccacgcca tactcccagg

105121 aaaagctcga acgaccttta aacaaaaggg tacctgtacc cgaaaccgac acaggtgggt

105181 aggtagagaa tacctagggg cgcgagacaa ctctctctaa ggaactcggc aaaatagccc

105241 cgtaacttcg ggagaagggg tgcctcctca caaagggggt cgcagtgacc aggcccgggc

105301 gactgtttac caaaaacaca ggtctccgca aagtcgtaag accatgtatg ggggctgacg

105361 cctgcccagt gccggaaggt caaggaagtt ggtgacctga tgacagggga gccggcgacc

105421 gaagccccgg tgaacggcgg ccgtaactat aacggtccta aggtagcgaa attccttgtc

105481 gggtaagttc cgacccgcac gaaaggcgta acgatctggg cactgtctcg gagagaggct

105541 cggtgaaata gacatgtctg tgaagatgcg gactacctgc acctggacag aaagacccta

105601 tgaagcttga ctgttccctg ggattgggtt tgggcctttc ctgcgcagct taggtggaag

105661 gcgaagaagg cctccttccg ggggggcccg agccatcagt gagataccac tctggaagag

105721 ctagaattct aaccttgtgt caggacctac gggccaaggg acagtctcag gtagacagtt

105781 tctatggggc gtaggcctcc caaaaggtaa cggaggcgtg caaaggtttc ctcgggccgg

105841 acggagattg gccctcgagt gcaaaggcag aagggagctt gactgcaaga cccacccgtc

105901 gagcagggac gaaagtcggc cttagtgatc cgacggtgcc gagtggaagg gccgtcgctc

105961 aacggataaa agttactcta gggataacag gctgatcttc cccaagagct cacatcgacg

106021 ggaaggtttg gcacctcgat gtcggctctt cgccacctgg ggctgtagta tgttccaagg

106081 gttgggctgt tcgcccatta aagcggtacg tgagctgggt tcagaacgtc gtgagacagt

106141 tcggtccata tccggtgtgg gcgttagagc attgagagga cctttcccta gtacgagagg

106201 accgggaagg acgcacctct ggtgtaccag ttatcgtgcc cacggtaaac gctgggtagc

106261 caagtgcgga gcggataact gctgaaagca tctaagtagt aagcccaccc caagatgagt

106321 gctctcctat tccgacttcc ccagagcttc cggcagcaca gccgagacag cgacgggttc

106381 tctgcccctg cggggatgga gcgacagaag ttttgagaat tcaagagaag gtcacggcga

106441 gacgagccgt ttatcattac gataggtgtc aagtggaagt gcagtgatgt atgcagctga

106501 ggcatcctaa cagaccggta gacttgaacc ttgttcctac atgacctgat caattcgatc

106561 aggcactcgc catctatttt cattgttcaa ctctttgaca acacgaaaaa ccattgttca

106621 actctttgac aacatgaaaa aaccaaaagc cccgccctcc ctctctatcc atccaaggga

106681 tggaagggca gaggcctttg gtgtcccttc cagtcaagaa ttggggcctc acaatcacta

106741 gccaataggc ttttctctca tgcccttctt cgttcatggt tcgatattct ggtgtcctag

106801 gcgtagagga accacaccaa tccatcccga acttggtggt taaactctac tgcggtgacg

106861 atactgtagg ggaggtcctg cggaaaaata gctcgacgcc aggatgataa aaagcttaac

106921 acctctcatt cttattactt tttcaacgaa taaaatgaaa aatcaaaagg tcgtcttatt

106981 caaaacccca attatgacat cccttctctc ccacttcaca cctcggaacg taccgagata

107041 aacgcgcttt cacatcttct taacccgaaa tggctgggga gaggaaaggt tccttttttt

107101 gagggtactc ccgggaacag atccagtgga gacggggtgg ggcctgtagc tcagaggatt

107161 agagcacgtg gctacgaacc acggtgtcgg gggttcgaat ccctcctcgc ccacaaccgg

107221 cccaaaaggg aagtaccttt ccccccgggg gtaggaaaat catgatcggg atagcggacc

107281 aaaagctatg gaacttgggt gtgggtcttt ggaatggctt tttcttttta tttcttattt

107341 atcgtaaatg atggaatcat tacacatagt atgccccccc gccatcagcg tatttttttg

107401 ttttacgccc tgtaactctt cctcagccag gcttgggcag aataggcgag caagtacaag

107461 tattagtagc ataacaaact tcctcgtcat taatatgttt gctcgcggca attgtgaact

107521 ctcgggagaa tcgatgactg catctttgat tttgatgcag tgctaataca tctgagaatt

107581 cttaattggc tagttgtaaa tagccccagg gctgtggatt atccggggcc tacaccgagg

107641 tattgacggt tattttaaaa tctcgcagaa cggaatggta tacgatgaga tagaaacaaa

107701 gacagggaac aggttcccta ctcttaacgg tcaaagtgag cccctttatt ctattctgaa

107761 ttcgttaatt cagaatgaat caaatctccc caagtaggat tcgaacctac gaccaatcgg

107821 ttaacagccg accgctctac cactgagcta ctgaggaaca acgggagatt agatctcata

107881 gagttcaatt cccgttctca acccatgaac aatatgagct cgaaccctcc ttcgtaactc

107941 ccggaacttc ttcgtagtgg ctcccttcca tgcctcattt cggagggaac ctcaaagcgg

108001 ctctatttca ttatattata ttccatccat atcccaattc cattcattta ataccccctt

108061 ttgtgtcatt gacataattt ctagtctatc tctttctatt tcttttctat atatggaaag

108121 ttcaaaaatc atcatataat aatccagaag ttgcaataga aaagaaaagg gggaggtttg

108181 tgatgatttt tcaatctttt ctactaggta atctagtatc cttatgcatg aagataatca

108241 attcggtcgt tgtggtcgga ctctattatg gatttctgac cacattctct atagggccct

108301 cttatctctt ccttctccga gctcaggtta tggaagaagg aaccgagaag aaggtatcag

108361 caacaactgg ttttattacg ggacagctca tgatgttcat atcgatctat tatgcgcctc

108421 tgcatctagc attgggtaga cctcatacaa taactgtcct agctctacca tatcttttgt

108481 ttcatttctt ctggaacaat cacaaacact tttttgatta tggatctact accagaaatt

108541 caatgcgtaa tttcagcatt caatgtttat ttctaaataa tctcattttt caattattca

108601 accatttcat tttaccaagt tcaatgttag ccagattagt caacatttat atgtttcgat

108661 gcaacaacaa gatgttattt gtaacaagta gttttgttgg ttggttaatt ggtcacattt

108721 tattcatgaa atggcttgga ttggtattag tctggatacg gcaaaatcat tctattagat

108781 cgaatgtact tattcgatct aataagtacc ttgtatcaga attgataaat tctaaggctc

108841 ggatcttgag tattctctta tttattacct gtgtctacta tttaggcaga ataccgtcac

108901 ccctttttac taagaaactg aaagaaacct caaaaacggg agcgggagaa agggtggaaa

108961 gtgcggaaga aagagatgta gaaatagaaa cagcttccga aatgaagggg actaaacagg

109021 aacaagaggg atccaccgaa gaagatcctt ctccttactt tttttcggaa gaaagggcgg

109081 atccgaacaa aatcgatgaa acggaagaaa tccaagtgaa tggaaaggaa aaagaattcc

109141 actttcgatt tacagagaca ggctatcaaa atagacccgt ttctgaagag tcttatctaa

109201 tgaatatcaa tgaaaatcag gataattcaa gattgaaaat atttgatcaa aaaactgaaa

109261 ataaagaact aattaaagga aaaagaattc cactttcgat ttacagagac aggctatcaa

109321 aatagacccg tttctgaaga gtcttatcta atgaatatca atgaaaatca ggataattca

109381 agattgaaaa tatttgatca aaaaactgaa aataaagaac taattatctt tgacaaacct

109441 cttgtaacta ttctttttga ttcgaagcga tggaatcgac catttcgata cataaaaaat

109501 aaacgatttg acaaggctat cagaaatgag atgtcacaat atttttttga catatgtcaa

109561 agtgatggaa aagaaagaat ctcttttaca tatcctccga gtttatccat ttttttggaa

109621 atgataaaaa aaaggatatc cccgcctaca atcgaaaaat tttcatttaa tgaactatat

109681 aacccttggg tttataccaa caaccaaaaa gagaaaaatt ttaacaacga gtttctaaat

109741 cgaattaaag ctttagataa agaaaatagt tatttgaata tactcgaaac acagactcga

109801 ttgtgtaatg actattctac aaaagaatac ttatcaaaaa ggtatgatcc cttcctgaac

109861 ggatcatatc gtaaaacaat ctacaaaagc ccttcgccct caaccctaaa aaaaactttg

109921 atagaaaatt tcctagatcc gtttgggata aatcggattc acggtatact tctcccggct

109981 actgattacc aagaatttga ccaaaaaata aatagatttg agataaaatc attatcaaca

110041 gaaattgtta attttttaac tttcatcagt aaatttgtta aagaatcggg atctacgaat

110101 ctaaatccga gtagccttta tttattttat ttattttcag aaggaaaaat agattctcaa

110161 aaagaaagaa aatattttaa ctatttatta aatttaaata aaattgtaac tgatgctaat

110221 ggtcaaaaaa ttaatagaaa atcgattaga ataaaagaaa tcaataaaaa agtcccccga

110281 tggtcataca aattaatcac cgatttagaa caacaatcaa gaaaatataa agaagaccta

110341 ccaataggtc atcaaattcg ttcaagaagg ggcaagcgtg tagttatttt gactgctacc

110401 aaaggtactc ctaagactac gaatagtaaa atgtccgata taaaaaccga cgtgacttta

110461 atgcgctatt cacaacaatc agactttcgg cgcggtataa tcaaaggttc tatgcgggct

110521 caaaggcgta aagtggttat tttcaaattg tttcaagcaa atgcgaagtc cccccttttt

110581 ttggagagac gacacaaagc ccctcccttt tattttaata tttccgggtt gattaaacta

110641 atttttaaaa atgggttgga taaaggggaa gcattcaaaa ttgtagagta tacaaaagaa

110701 caaacaaaaa aacaagaaaa caacaaaaga aaggaaaacg cacgaataaa agtcgcagag

110761 gattggaatc gtattccatt tgcgcaagca ataagaggtt gcgtgttact aactcaatct

110821 atttttagaa aatatattct attcccttca ttgataattg ccaaaaatgt tggacgtata

110881 ttcctattgc aacgtcctga atggtctgag gatttccaag aatggaataa agagatctat

110941 attaaatgca cctctaatgg tattccatta tccgaaaccg aatttccaaa aaattggttg

111001 acagaaggta ttcaaataaa aatcgtattt cctttctgtc tgaaaccttc gcacaaatcg

111061 aaactacgat cctctcaaaa agatctaatg aaaaagacaa aaggtgatta ttgtttttta

111121 acagtttggg gaatggaaac ggaactcccc ttttcttcac cccgaaaaaa accttctctt

111181 attaaaccca ttttaaagga attcccaaaa aaaattggaa aattgaaaaa gaagtatttt

111241 cgagttctaa cagttttcaa agtaaaaaca aaattacttc gaaaagtttt aaaagaaaca

111301 aaaaaatggg ttatcaaaag tgtttttttt agaaaaagaa taataaaaga actttcaaaa

111361 gtaaatccaa ttctattatt tagattaaga gaagtcggag tcgataaatc aagcgaaatt

111421 aaagaagaaa aagattccat aataaacaac caaacgattc acgaatcatt tactcaaatt

111481 caaattgcat ctccgagttg gacaaactct tcgttgacag aaaaaaaaat gaaggatctg

111541 actgatagaa caagtacaat tcgaaatcaa atagaaagaa tcacaaaaga gaaaaaaaaa

111601 gtaactccaa gaataaataa tcttagtcct acaagttata atgctaaaaa attagaaaaa

111661 cagcaaatgt taaaaatgtt aaaaagaaga aatgctcgat taatctgtaa attatcccct

111721 tttgtaaaat ttttcattga aaaaatatac acggatatat ttttatatat cattaatatt

111781 gccagaataa atacaaaact ttttcttaaa ttaacaaaaa aaattattga taaatccatt

111841 tacaataatg aaagaaaaca agaaagaatt aataaaaaaa agaaaactaa aattctgtct

111901 atttcgagta taattctaag aaaggagctt gagaatatta gtaatattaa agcaaattca

111961 catatttttt atgacttatc ctacgtgcca caaccatatg tattttataa attaggaaaa

112021 atccaagtta ttaactcgtt aagatttgtt gttcaatatc aacgaatccc ctttttgctt

112081 aaggctaaaa taaaggattc ttttgaaaca caaggaatgc ttgattcgaa atcagcagat

112141 aacaaaatta cgagttatga aatgaatcca tggaaaagct ggttaagagg acattatcaa

112201 taccatttat ctcagattgg atggtctaga ttaataccag aaaaatggcg aaatacattt

112261 cgtcagcagc gtatagctaa aaaggcaaat tttagcaaac ggcattcata tgaaaaaaac

112321 ccattaatga attccaaaaa acaaaaaaaa tttgaagtat attcattatc taatcaaaaa

112381 gataatttta taaaatacta tcgatctgat cttttatcat ataaatttat tcattatgaa

112441 aagaaaacgg aatgcttttt ttatggatct ccccttcaag gaaatacgaa acaagaaatt

112501 tattataaca cgcctaaaaa aaactttgtt gctatgctga ggaacatccc tattaaaaat

112561 gatctaggaa agatccatat ggaaaaaccg gccgatagaa aatattttga ttggaaaatt

112621 tttcaatttg atcttataca aaaagtcgat attgaggcct ggatcataat cgataccaat

112681 aggaatcaaa atactcaagt tcgtactaaa aattctcaaa taatttctaa aaaagatttt

112741 ttttatcttc agattccaga gatcaattta ccaaactctc acaaggggtt ttacgattgg

112801 atgggaatga atgaaaaaat gctaaagcat cccatatcca atctagaact ttggttcttc

112861 ccagaatttt tgtcaattta taaaacatat aaaatgaaac cttggtttat accaagcaaa

112921 ttacttcttt taaatttaaa tagaagtgca aataaaaaga tcaacgaaaa gggcaatttt

112981 ttgatagcat caaataaaaa gcatcgaaat caagaagaaa aagaaccgac aagtcgagga

113041 gagcggagat ccgttctctc accccaaaaa gatacggaag aaaattatgc aagatcaaac

113101 atggaaaaag ggaaaaataa aaaacaatac acgaaagcag aactccgttt gttcatgaaa

113161 cgttatttgc tttttcaatt gcaaggggat gagactttga atgaaagaat gatcaataat

113221 atcaatgtat attggttcct gcataaactg atagatttaa caaaaattac tatatcctcg

113281 attcaaaaga aacaaatgag tttggatata atgattaata ataatttaac tctttcagaa

113341 tttatgcaga agggagtatt tattctagaa cccattcgtc tgtctgaaca aaaagatggg

113401 caatttatta tgtatcaaac cgtgggtatt tcgttggttc ataagaataa gcatcaaaaa

113461 taccaagagc aaggacatgc ttctaacaat aattttgatt tacttgttcc cgaaaatatt

113521 ttatccttta gacgtcgtag aaaattgaga attctaattt gtttcaattc aaaaaagaga

113581 aattatatag atcaaaatcc ggtattttgg aacgtaaaaa gcagcagcca agttttacat

113641 gacaataacc atcttgatag agataaaaat caattaatga aattaaagct ctttctttgg

113701 cctaattatc gattagaaga tttagcttgt atgaatcgtt attggtttga taccaataat

113761 ggcagccgtt tcggtatgtt aaggatacag atgtatccac gattgaaaat tttttgataa

113821 tacacttttc tgtatatcct ataccctata tatataatag ggtatatgaa agcaaacaaa

113881 tacacagatc agatgtctta tctcacattt cattctagta tccaatatca aatgaataat

113941 gtctgaattc gatctcgaaa tgaatcaacg gaaccttctt tattttaggt gtaaattctt

114001 cgatcaaaaa atgtaccaac ctttatattc ctatagaaaa ccaattcaaa attggattga

114061 tttatttgaa ttcaggaaat taggagttca taaagaaatt ttgattagat tcttgtatat

114121 accacattca aattaatggc attattatta ctgatcggta aaatccatat ctgtaaaaag

114181 ggcaaggggc gtttttttat ggtcaaaaat tcatcgattt cgcttatttc tcaaaaagaa

114241 aacaaagaaa ccaggggatc tgttgaattt caagtattca gtttcaccac taaaataagg

114301 aaactcactt cccatttgga attgcacaaa aaagactttt catctcagcg aggtttgcga

114361 aaaattttgg gaaaacgtca acgactgctg gcctatttgt caaaaataaa tagggggcgc

114421 tataaagaat taattgggga gttggatatt cgagagataa aaactcgtta attttgaagc

114481 gtgagaccgt ttgagcttag tcgtttaaat tttgatgaac ttctttcttt ttactttcag

114541 caatgcatgg aagaatgact cgagaaaaac ttatgattcc accaactaca agaaaagacc

114601 tcatgatagt caatatgggc cctcaccacc catcaatgca tggtgttctt cgactgatcg

114661 ttactctcga tggtgaagat gttattaact gtgaaccagt attgggttat ttacatagag

114721 ggatggagaa aattgcggaa aatcgaacaa ttatacaata tttgccttat gtaacacgtt

114781 gggattattt agctactatg ttcacagaag caataaccgt aaacgggccc gaacagctag

114841 ggaatattca agtgcctaaa agggctagct atatcagagc tattatgttg gagttgagtc

114901 gtatcgcttc ccatttgtta tggcttggtc cttttatggc agatattggg gcgcagactc

114961 ctttcttcta tatttttcga gaacgagaat tgatatatga cctatttgaa gcggctaccg

115021 gcatgcgcat gatgcataat ttttttcgta ttggaggagt cgctgctgat ctacctcatg

115081 gctggataga taaatgtttg gatttttgcg attatttttt aaccggggtt gctgaatatc

115141 aaaagcttat tacacggaat cctatttttt tagaacgggt tgagggcgta ggcattattg

115201 gcggagaaga ggcactaaac tggggtttgt cgggaccaac gctacgagct tccggaatac

115261 aatgggatct tcgtaaagtt gatcgttatg agtgttacga ggaatttgat tgggagattc

115321 aatggcaaaa agagggggat tcattagctc ggtatttagt acgaattggc gaaatgacag

115381 aatctataaa aattatccag caggctctgg aaggaattcc agggggaccc tatgagaatt

115441 tagaaagccg ccgctttgat agaataaaag accccgaatg gaatgatttt gaatatcgat

115501 ttattagtaa aaaaccttct cctacttttg aattgtccaa gcaagaactt tatgtaagag

115561 tcgaagcacc aaaaggagaa ttaggaattt ttctgatagg agatcggagt gtttttcctt

115621 ggagatggaa aattagaccg cctggtttta tcaacttgca aattcttccg cagttagtta

115681 aaagaatgaa attggctgat attatgacga tactaggtag catagatatt attatgggag

115741 aagttgatcg ttgaaatgat aattgataca acaaaaatac aagctatcaa ttcttttttc

115801 agattgggat ccttaaaaga agtctatggg atcatatgga tgcttatccc tattttcatt

115861 cttgtattag gaatcacact aggtgtacta gtaattgttt ggttagaaag agaaatatct

115921 gcagggatac aacaacgtat tggacccgaa tacgcgggtc ctttcggaat tcttcaagct

115981 ttagcagatg gtataaaact acttttcaaa gaaaatattc ttccatctag aggagatact

116041 cgtttattca gtctcgggcc atccatagca gtcatatcca ttttactaag ttattcagta

116101 attcctttta gctatcgctt tattctagcc gatcttagta ttggtgtttt tttatggatt

116161 gctgtttcaa gtcttgctcc cgttggactt cttatgtcgg gatatggatc aaataataaa

116221 tattcctttt taggtggatt aagggctgct gctcaatcaa ttagttatga aataccatta

116281 accctatgtg tattatcaat atctctacgt gtgattcggt gagacataaa atttccccta

116341 ttgattttct ttctaggaag aaagtgaaat ggttggaata tccatttttt tattcattat

116401 tgggttgatg aattaaacca gatagttata tgagtgaaat aaaacggctt aaaattttgc

116461 tgttaaagga atttaatctc atttcctatg tacaagaagt aagtgaaagt aaacataagc

116521 agccgagact gtttatccca agattggttg attagtcatt ttgtcttgaa gcgggttcaa

116581 aagatcaacc gtatggggtt tttactatac tattacgata gacgtattac cctaaatgag

116641 agattcaaaa aaaacgagtg gatggttagg aagaccaaga tacacaaagg agtagtaatg

116701 gagattctgt aaattatcca acaggatatt ttttttatag aaaagtaatt ctaattgggg

116761 ctttaagttg gtagaaattc ttaagaagta ctccccacga tttcatccag agtatgttcc

116821 tatccaccaa ttaagtaaat aactatcaaa aacgacgaaa tcttttactt tgggtaatgt

116881 gccttttctg agaaaggaga ataggaacga actaaaatcc aaaaacgaga attgcaaaaa

116941 gagatctttt ttttattcct gactatttcg attttattta cagaaataca attcttgtta

117001 tgtaatgcaa tatgtaattt tttttcgtaa tcaaaagtga gaaaatgata ggttgaaata

117061 tctatgcgat attctctaaa aagtattttt tcattcaagg ataaagttat taatcaacaa

117121 agaaaaataa aaattcttaa aagatgagat caattcagaa gcactttttt attaaaaatc

117181 tagcagacag aattccattg gtctaattct aggccctagg ataataattt gattctatat

117241 agatcctaca aacacatcaa gagaaataat gttgacaggt ccttagattt atttctgacc

117301 tatgaggagc cgtatgaggt gaaaatctca tgtacggttc tgtaatagcg acgggaacgg

117361 tgatgttagc gtcgactagg attatctaac agtttaagta cagttgatat agttgaagcg

117421 caatcaaaat atggtttttg gggatggaat ttatggcgtc aacctatagg gtttattgtt

117481 tttctgattt cttctctagc cgagtgtgag agattacctt ttgatttacc agaagcagaa

117541 gaagaattag tagcaggtta tcaaaccgaa tattcaggta tcaaatttgg tttattttat

117601 gttgcttcgt atctaaatct actcgtttct tcattatttg taacagttct ttacttgggg

117661 ggttggaatc tttctattcc atacctattc gttcctgagc tttttgacat aaataaaaga

117721 agtcctgttt ttggaacaat aatcggtatc tttattacat tagctaaaac ttatttgttc

117781 ttgttcattt ctattgcaac aagatggact ttaccgagac tgagaatgga ccaactttta

117841 aatcttggat ggaaattcct tttaccaatt tctctaggta atctattatt aacaacttcg

117901 tcccaacttc tttcactgta aaggaataga aatagaatag gcttttcttt ataacttgtc

117961 tcaaacaaga gaaagaaaaa agtaaaatta tttatagata ttcagatatg ttccctatgc

118021 taactcagtt cttaaactcc ggtcaacaaa caatacgagc tgccaggtac attggtcaag

118081 gtttcatgat caccttgtcc cacgcgaatc gtttacctgt aactattcaa tacccctacg

118141 aaaaattgat cacatccgaa cgtttccgag gccgaatcca ctttgaattt gataaatgca

118201 ttgcttgtga agtatgtgtt cgtgtatgtc ctatagatct acccgttgta gattggaaat

118261 tgcaaactga tattcgaaag aaacgattac ttaattacag tattgatttt ggaatctgta

118321 tattttgcgg taattgcgtt gagtattgtc caacaaattg tttatcaatg actgaagaat

118381 atgaactttc tgcctatgat cgtcacgaat tgaattataa tcaaattgct ttaggtcggt

118441 taccggtatc aataattgaa gattacacaa ttcgaacaat ttcttcgaat ttacctcaaa

118501 taaaaaatgt ataaaacctt cgattcacaa aacatttaaa aattcaaatc acaaaagctt

118561 tataggtttt ggatctaaag aaaggaatga ggtttttgat gaggtttttg cttggtcaat

118621 acaaaagaac ggctgggcgg tgaaaatcgc ggcttatttt tgattaaaaa tggatttcta

118681 ttcgaataat gatttgaaaa tataaagttt gaacctctgc ttcgattgct tcgataataa

118741 gagtagtcca ataactgtat ttacatcaat ccaaatcaac cccgttccaa tttcattcaa

118801 ttaagaagta tcctaaaaaa aaaaaaagga taacttcgtt tttcctggtc aggtcaaaaa

118861 ggacatgaaa tatttcgatt ttttttataa aatcaaatgg atttacctgg accaatacat

118921 gattttcttt tagtctttct gggattgggt cttatattag gaagtttggc agtagtatta

118981 cttccgaatc caatttattc ggccttttcg ttgggatggg ttcttttttg tatatcctta

119041 ttctatattc tatccaactc ctattttgta gctgctgcgc agctccttat ttatgtagga

119101 gctataaacg ttttaatcat ttttgctgtg atgttcatga atgggtcaga ctattacaaa

119161 gattttcatc tttggaccgt tggagatgga gttacttcaa tagtttgtat aagtcttttt

119221 atttcactaa ttactactat tccagatacg tcctggtatg ggatcatttg gactacaaaa

119281 tcaaatcaga ttctagaaca agatttgata agtaatagtc aacaaattgg aattcattta

119341 gcaacggatt tttttcttcc atttgaactc atttcaataa ttcttttagt cgctttaata

119401 ggtgctattg ctatagctcg tcaataagaa atctttaaaa tgagtaattc aaatagaatc

119461 cacgcaaaag gaatgttata attcgttaat ttgaatttct gtctaaaaaa atacaataaa

119521 aagactttaa ttttatattg atctcttacc aatctattcc attattccat atttgttaga

119581 atcgaatcga tttaattatt gttcagatta aaaatgaatc aaaattgata aggagttggt

119641 taatgatgct cgaacatata cttgttttga gtgcctactt attttctatt ggtatctatg

119701 gattaatcac aagtcgaaat atggttagag cccttatgtg tcttgaactt atattaaatt

119761 cagttaatat caattttgtc acattttctg atatttttga taatcgtcaa ttaagaggag

119821 atattttctc catttttgtt ataactattg cagccgctga agcagctatt ggattggcta

119881 ttgtttcatc aatttatcgt aacagaaaat caactcgtat taaccaatcc aatttgttga

119941 ataaatagta ttaatcatat aaatgctaat tttgaatatt aataaataaa ttaaaattcg

120001 cattagcggg tttgatatgt ctatagtagg gtataatcgt agttgcaaaa acataagaaa

120061 tcaaagtatt ttggccctcc ctcataaatc aattcggaag taaattgatt cttatcactc

120121 attgattcgt ctggtatcta actataggat tcatttataa gttagtttac taaaccgaaa

120181 attgatggcg ttcaaaaacg tatagatcca atgtcacatt cagtaaagat ttatgataca

120241 tgtataggat gtactcaatg tgtccgggcg tgccccacgg atgtattaga aatgataccc

120301 tgggacggat gtaaagctaa acaaatcgcc tctgctccaa ggaccgagga ctgtgttggt

120361 tgtaagagat gtgaatccgc ctgtccaacg gattttttga gcgttcgggt ttatttatgg

120421 catgaaacaa ctcgcagtat gggtctagct tattgataca ttccataaaa atctacttga

120481 atccattttt tttttatcga aaaaatccgt gctcaattca atttgatttt gagcacggat

120541 ttttctggcc caagtgtatc ttgtctttac cacgaaccat tttccttgct taacaataat

120601 tgtagtttta ccaatatttg cgggttgctt catttttttt cttccacaca ggggaaatag

120661 agtaatacgc tggtatacta tatgtatgtg tatattagaa cttcttctaa cgacttatgc

120721 attctgctat cattttcaat cggatgatcc actaattcaa ctaatggagg attataaatg

120781 gatccatttt ttggatttcc attggagatt aggaatagac ggactctcta taggacccat

120841 tttactgacg ggattcatca ccactttagc tactttagcg gcttggccgg ttactcgaga

120901 ttctcgatta tttcatttcc tgatgttagc aatgtacagt gggcaaatag gattattttc

120961 ttctagagat cttttacttt ttttcctcat gtgggagtta gaattaattc ccgtttatct

121021 acttgtatcg atgtggggag gaaaaaaacg tctgtactca gctacaaaat ttattttgta

121081 cacggcgggg ggttctgttt ttcttttaat gggagttctg ggtatcggtt tatatggttc

121141 tactgaacca acattaaatt ttgaaatatt agccaaccgg tcctatcctg tgaacttgga

121201 aatactattt tatattggat tttttcttgc ttttgctgtc aaattgccaa tcatacccct

121261 acatatatgg ttacccgata cccatggaga agcacattat agtacttgta tgcttctagc

121321 cggaatctta ttaaaaatgg gagcgtatgg attagttcgg atcaatatgg aattatttcc

121381 tcatgctcat tctatatttt ccccttggtt aatggtagta ggcgcaatgc aaataatcta

121441 tgcggcttca acatctctcg gccaacggaa tttaaaaaaa agaatagcct attcctctgt

121501 atctcatatg ggtttcataa ttataggaat tggttctatc acagatatgg gactcaacgg

121561 agccctttta caaataatct ctcatggatt tattggcgcg gcgctttttt tcttggccgg

121621 aacaacttat gatagaatac gtcttgttta tcttgacgaa atgggcggaa taggtattcc

121681 aatgccaaaa atattcacga tgttcagtag cttttcgatg gcctcccttg cattacctgg

121741 catgagtggt tttgttgctg aattaatagt tttttttgga ctaattacta gtccaaaata

121801 tcttttaatg acaaaactcc caattacttt tgtaatggca attggaatga tattaactcc

121861 tatttattta ttatctatgt tacgacagat gttttatgga tacaagatat ttaatggccc

121921 agactcttat ttttttgatt ccgggccgcg agagttattt ctttcggttt ctattttttt

121981 acccgtactc ggtattggta tgtaccccga tttcgttctt tcactatcag ttgaaaaggt

122041 tgaagttatt ctatctaatt ctttttttag ataattgata aatcttatca tttacaaaag

122101 cgttcgttgt aaaatggtac aatgacaaag agcctgtcga atcatatatg attcgacagg

122161 ctctcgccaa ttaacacaaa gtttttttat ctgatctgcg ttgtacaccc ttttattgct

122221 atttgtaata accccccttt ttcttttttt gaattcaatt agatgttaat gtaaatgaac

122281 cataactatg tagtcctatt cctaatagat tgactccaaa atagcatatc caaattataa

122341 gaaatccaat agacgccaca attgctgaat ttgtaccctt cagttttata tttgttcgag

122401 tatgtaaata aattgaaaat atgatccaag taataaaagc ccaagtttcc tttgggtccc

122461 aactccaata ggatccccat gcttcgttag cccatactgc tcccgaaaga attcctatgg

122521 ttaaaaagat aaatcctaga ctaataactc gataactcca ataatccaat ttttgaatca

122581 actgtgatct ataataattc cttgcgaaaa aaaaagaagt tttttggaaa aaatcccttt

122641 gttcattcat gtattcgatt tcaccaagga aaaatgactc atttaaattc aataaatgat

122701 tactcgtaga aaaaagagaa agcttttttc taactgtaat gactagaagt gatactgata

122761 ataatgatcc acataaaagg gccgcatagc ctaatatcat catacttacg tgcattatta

122821 gccactcgga ttgaagagcg ggtactaaga ttgtcgattc gtgtatttca gttaaaagac

122881 ctgaagtagc aaagccctgg gaaaaaatag cacttgggcc aattattgtg cttagaatat

122941 tttggttttt tttgaaatat gaaactatat aaataaagga aaaactccat gaaagaaaaa

123001 ttaacgattc gtataaatca cttagtggaa aatgtcccga ataaatccaa cgagaaatta

123061 ataatcctgt tatacagaaa aaagtcatta tcatgcccgt tttggatgaa tcatctagtt

123121 ttacgatttc atcgactaaa aaggttatca aatgaattgt aattataatt gaaacgatcg

123181 aaaaagaaat atgagttaat atatgctcta aggttgaaaa tatcataaaa taaagagttc

123241 cttaattata aaatcgaaat tagcaattga atttattata ggatctattt tattttttta

123301 agagatgata tgccgccact cggactcgaa ccgagatgct ctagcactgc ttcctaagag

123361 cagcgtgtct accgatttca ccatagcggc ctgtcttgac ctcataataa cctatgaata

123421 aataatcgtc tagatttacg aatttaattg agtgcaatat tttttaggaa agattcaaat

123481 tgatgaataa aaatttggtc aaataggtat ttgaaggttc attagtttcg tttaggattt

123541 ttgttttatt ttgtatttta gactcttctc gactcaactc ttgtaaatcc tactatgaat

123601 taaggaatag aaccctccct ccctcaaaaa catttttttt aattaactgt ttttgattta

123661 tttgatttca aaaagtgaaa ccaaaaagcc gttttatcga tgaacaaaaa aaaaagaacc

123721 tattttacat cattcaaaat ttacacatat ttatccaatt tggattgggt aaggtaaaca

123781 gaaaaattct tattcgccat atgctataag agcagaacga attccattcc tcgttgaagg

123841 aaatggaatt cgggaatttg atttttgaaa tgaaatgact ccacttttga gtcaggccgg

123901 tttagattat tccaacgtgt tatgttttat tacttagttt atttgtttgt cgcacaaaaa

123961 aactttttga attcccggtg gaaagagatt tacctaagga aaatgctttt aacgctgccc

124021 gatacccctt ccttttccaa aaatttttac gaatacgctt ttttgatgca gaagtacgtt

124081 tttttggaac tgccatttaa ataaaaatta agagattact cattggtata gctggatgtg

124141 aaagacatct attgttcaaa aaaatatgag cttttctgat tcactatgta tttcttttct

124201 agaaaatcct tttttcgaaa aatagaaaag aatcgataag atgggtgaac agtatggcaa

124261 aatagcataa aatagttcta aaaagagaaa aaaatcagat ttttaataac ttatcaaatc

124321 cgggaaaaat atgcccaatt tgacttgaat ttgaaaattg gaaggaaatt ggtaattaat

124381 ctatttcttt catatgattt gaccaattgt aacttcttga tttgaatatt tgaagtattt

124441 agcagaaatt cagaacaaat aagtaagaag aaataaaaat tccaaaattt tatttaagtt

124501 agtacatatt ttcatttttt tcttgactcc tttcaaaaga ggtaaggtcg ggtgaattgg

124561 aaaccgttaa tattaattaa aaattttaaa aacctttttt ttatggaaca gacatatcaa

124621 tatgcgtgta ttttaccttt cgttccactt ctagttccta tattaatagg agtgggactt

124681 gttatttttc cgacagcaac aaaaaatctt catcgtatgt gggcttttcc aagtatttta

124741 ttgttaagta tagtcatgat tttttcaact aatctgtcta ttcaacaaat aaatagcagt

124801 tatatctatc aatatgtatg gtcttggacc ctcgataatg atttttcttt agaatttggc

124861 tgcttgattg atccacttac ttctattatg ttgatgttaa tcactactgt tggaattatg

124921 gttcttattt atagtgataa ttatatggct cacgatcaag gatacttgag attttttgct

124981 tatatgagtt ttttcaatac ttccatgttg ggattagtta ctagttcaaa tttgatacaa

125041 atttatattt tttgggaatt ggttggaatg tgttcctatc tattaatagg gttttggttc

125101 acacgacctc ctgcggcaaa tgcttgtcaa aaagcgtttg taactaatcg tgtaggggat

125161 tttggtttat tattaggaat tttgggtttt tattggataa caggtagttt tgaatttcga

125221 gatttattcg aaatactcaa taacttgatt tctaataatg aagtcaattt tccatttgtt

125281 attttgtgtg ctgctctatt atttgccggc gcagttgcta aatctgcaca atttcccctt

125341 catgtgtggt tacctgatgc tatggagggc cccactccta tttcggctct tatacatgcc

125401 gctactatgg tagcggcggg gatttttctt gtagctcgcc ttcttcctct tttcgtagtt

125461 ataccttata taatgaattt aattgcgttg atgggcataa ttacactatt attaggagct

125521 actttagctc ttgctcaaaa agacattaag aggagtttag cttattcgac aatgtctcaa

125581 ttgggttata tgatgttcgc tctaggaatg gggtcttatc gaagtgcttt atttcatttg

125641 attactcatg cttattccaa agcattatta tttttggggt ccggatccgt tattcattca

125701 atggaaactc ttgttggtta ttctacggat aaaagtcaga atatggttct tatgggtggt

125761 ttaacaaaac atgtaccgat taccaaaacc tcttttttat taggtacact ttctctttgt

125821 ggtattccgc cacttgcttg tttttggtca aaagatgaaa ttcttaatga tagttggttg

125881 tattcgccga ttttcgcaat aatagcgttg gccacggcag gattaacggc attttatatg

125941 tttcgtattt atttacttac ttttgagggg catttgaaca ttcatttcca aaattatagt

126001 ggcaacaaaa atacctcttt ctattccata tccatatggg gtaaagggta ttcaaaaaga

126061 attaacccca atttttcttt attaagaaat gaaagttctt cttttttttg gaaaaagaca

126121 tgtcgaagtg atgagaatgc aagaaaaaaa gggggggggc acccttttat taatattctc

126181 cattttgata atcaaaagtc cttttcctat ccttatgaat ccgcgaatac gatgttattt

126241 tctttacttc tattagtcct atttacttta tttgttggat ctctaggaat tccttttaat

126301 caaaaaggaa cagatttgga tctattatcc aaatggttag ctccgtctat taacctttta

126361 catcaaaagt caaaggattc ggcaagttgg tatgaatttt ttaaagatgc ccttttgtca

126421 gttagtatag cttattgcgg aatatttcta gcgtcctttt tatataaacc tatttattcc

126481 tctttccaaa atttcgactt aataaattca tttgtgaagt taggtccgaa aagaaaacgt

126541 ttggataaaa ttataaacgc cctatatgat tggtcatata atcgtgctta tatcgattct

126601 ttttatacaa tatccttttc caggggggtc agggaattgg cccaattaac tcattttttt

126661 gatagacgag taattgatgg aattacgaat ggagttggtg tcatgagttt ctttttagga

126721 gaagggatca aatatctagg gggcggtcgt atttcttctt atcttttctt ctatttttct

126781 tttgtatcga tttttttaat tagttcttta ttttcagttt tttgatcaaa tattttcaat

126841 cttgaattat cctgattttc attgatattc attagataag actcttcaga aacgggtcta

126901 ttttgatagc ctgtctctgt aaatcgaaag tggaattctt tttcctttcc attcacttgg

126961 atttcttccg tttcatcgat tttgttcgga tccgcccttt cttccgaaaa aaagtaagga

127021 gaaggatctt cttcggtgga tccctcttgt tcctgtttag tccccttcat ttcggaagct

127081 gtttctattt ctacatctct ttcttccgca ctttccaccc tttctcccgc tcccgttttt

127141 gaggtttctt tcagtttctt agtaaaaagg ggtgacggta ttctgcctaa atagtagaca

127201 caggtaataa ataagagaat actcaagatc cgagccttag aatttatcaa ttctgataca

127261 aggtacttat tagatcgaat aagtacattc gatctaatag aatgattttg ccgtatccag

127321 actaatacca atccaagcca tttcatgaat aaaatgtgac caattaacca accaacaaaa

127381 ctacttgtta caaataacat cttgttgttg catcgaaaca tataaatgtt gactaatctg

127441 gctaacattg aacttggtaa aatgaaatgg ttgaataatt gaaaaatgag attatttaga

127501 aataaacatt gaatgctgaa attacgcatt gaatttctgg tagtagatcc ataatcaaaa

127561 aagtgtttgt gattgttcca gaagaaatga aacaaaagat atggtagagc taggacagtt

127621 attgtatgag gtctacccaa tgctagatgc agaggcgcat aatagatcga tatgaacatc

127681 atgagctgtc ccgtaataaa accagttgtt gctgatacct tcttctcggt tccttcttcc

127741 ataacctgag ctcggagaag gaagagataa gagggcccta tagagaatgt ggtcagaaat

127801 ccataataga gtccgaccac aacgaccgaa ttgattatct tcatgcataa ggatactaga

127861 ttacctagta gaaaagattg aaaaatcatc acaaacctcc cccttttctt ttctattgca

127921 acttctggat tattatatga tgatttttga actttccata tatagaaaag aaatagaaag

127981 agatagacta gaaattatgt caatgacaca aaagggggta ttaaatgaat ggaattggga

128041 tatggatgga atataatata atgaaataga gccgctttga ggttccctcc gaaatgaggc

128101 atggaaggga gccactacga agaagttccg ggagttacga aggagggttc gagctcatat

128161 tgttcatggg ttgagaacgg gaattgaact ctatgagatc taatctcccg ttgttcctca

128221 gtagctcagt ggtagagcgg tcggctgtta accgattggt cgtaggttcg aatcctactt

128281 ggggagattt gattcattct gaattaacga attcagaata gaataaaggg gctcactttg

128341 accgttaaga gtagggaacc tgttccctgt ctttgtttct atctcatcgt ataccattcc

128401 gttctgcgag attttaaaat aaccgtcaat acctcggtgt aggccccgga taatccacag

128461 ccctggggct atttacaact agccaattaa gaattctcag atgtattagc actgcatcaa

128521 aatcaaagat gcagtcatcg attctcccga gagttcacaa ttgccgcgag caaacatatt

128581 aatgacgagg aagtttgtta tgctactaat acttgtactt gctcgcctat tctgcccaag

128641 cctggctgag gaagagttac agggcgtaaa acaaaaaaat acgctgatgg cgggggggca

128701 tactatgtgt aatgattcca tcatttacga taaataagaa ataaaaagaa aaagccattc

128761 caaagaccca cacccaagtt ccatagcttt tggtccgcta tcccgatcat gattttccta

128821 cccccggggg gaaaggtact tcccttttgg gccggttgtg ggcgaggagg gattcgaacc

128881 cccgacaccg tggttcgtag ccacgtgctc taatcctctg agctacaggc cccaccccgt

128941 ctccactgga tctgttcccg ggagtaccct caaaaaaagg aacctttcct ctccccagcc

129001 atttcgggtt aagaagatgt gaaagcgcgt ttatctcggt acgttccgag gtgtgaagtg

129061 ggagagaagg gatgtcataa ttggggtttt gaataagacg accttttgat ttttcatttt

129121 attcgttgaa aaagtaataa gaatgagagg tgttaagctt tttatcatcc tggcgtcgag

129181 ctatttttcc gcaggacctc ccctacagta tcgtcaccgc agtagagttt aaccaccaag

129241 ttcgggatgg attggtgtgg ttcctctacg cctaggacac cagaatatcg aaccatgaac

129301 gaagaagggc atgagagaaa agcctattgg ctagtgattg tgaggcccca attcttgact

129361 ggaagggaca ccaaaggcct ctgcccttcc atcccttgga tggatagaga gggagggcgg

129421 ggcttttggt tttttcatgt tgtcaaagag ttgaacaatg gtttttcgtg ttgtcaaaga

129481 gttgaacaat gaaaatagat ggcgagtgcc tgatcgaatt gatcaggtca tgtaggaaca

129541 aggttcaagt ctaccggtct gttaggatgc ctcagctgca tacatcactg cacttccact

129601 tgacacctat cgtaatgata aacggctcgt ctcgccgtga ccttctcttg aattctcaaa

129661 acttctgtcg ctccatcccc gcaggggcag agaacccgtc gctgtctcgg ctgtgctgcc

129721 ggaagctctg gggaagtcgg aataggagag cactcatctt ggggtgggct tactacttag

129781 atgctttcag cagttatccg ctccgcactt ggctacccag cgtttaccgt gggcacgata

129841 actggtacac cagaggtgcg tccttcccgg tcctctcgta ctagggaaag gtcctctcaa

129901 tgctctaacg cccacaccgg atatggaccg aactgtctca cgacgttctg aacccagctc

129961 acgtaccgct ttaatgggcg aacagcccaa cccttggaac atactacagc cccaggtggc

130021 gaagagccga catcgaggtg ccaaaccttc ccgtcgatgt gagctcttgg ggaagatcag

130081 cctgttatcc ctagagtaac ttttatccgt tgagcgacgg cccttccact cggcaccgtc

130141 ggatcactaa ggccgacttt cgtccctgct cgacgggtgg gtcttgcagt caagctccct

130201 tctgcctttg cactcgaggg ccaatctccg tccggcccga ggaaaccttt gcacgcctcc

130261 gttacctttt gggaggccta cgccccatag aaactgtcta cctgagactg tcccttggcc

130321 cgtaggtcct gacacaaggt tagaattcta gctcttccag agtggtatct cactgatggc

130381 tcgggccccc ccggaaggag gccttcttcg ccttccacct aagctgcgca ggaaaggccc

130441 aaacccaatc ccagggaaca gtcaagcttc atagggtctt tctgtccagg tgcaggtagt

130501 ccgcatcttc acagacatgt ctatttcacc gagcctctct ccgagacagt gcccagatcg

130561 ttacgccttt cgtgcgggtc ggaacttacc cgacaaggaa tttcgctacc ttaggaccgt

130621 tatagttacg gccgccgttc accggggctt cggtcgccgg ctcccctgtc atcaggtcac

130681 caacttcctt gaccttccgg cactgggcag gcgtcagccc ccatacatgg tcttacgact

130741 ttgcggagac ctgtgttttt ggtaaacagt cgcccgggcc tggtcactgc gacccccttt

130801 gtgaggaggc accccttctc ccgaagttac ggggctattt tgccgagttc cttagagaga

130861 gttgtctcgc gcccctaggt attctctacc tacccacctg tgtcggtttc gggtacaggt

130921 acccttttgt ttaaaggtcg ttcgagcttt tcctgggagt atggcgtggg ttacttcagc

130981 gccgtagcgc ctggtgctcg aacattggct cgaggcattt tctctacccc ttcttaccct

131041 gaaaaagcag ggacacctta cgttcttgaa ccgataacca tctttcggct aacctagcct

131101 gctccgtccc tcgggaccaa caaggggtag tacaggaata ttcacctgtt gtccatcgac

131161 tacgcctttc ggcctgatct taggccctga ctcaccctac gtggacgaac cttgcggagg

131221 aacccttagg ttttcggggc attggattct caccaatgtt tgcgttactc aagccgacat

131281 tctcgcttcc gcttcgtcca ccgccgctcg cgcggaaact tccctctaag gcggaacgct

131341 cccctaccga tgtattttta catcccacag cttcggcaga tcgcttagcc ccgttcatct

131401 tcggcgcaag agcgctcgat cagtgagcta ttacgcactc tttcaagggt ggctgcttct

131461 aggcaaacct cctggctgtc tctgcacccc tacctccttt atcactgagc gatcatttag

131521 gggccttagc tggtgatccg ggctgtttcc ctctcgacga tgaagcttat cccccatcgt

131581 ctcactggtc gactttgacc cctgttattt tgaggtcata tctagtattc agagtttgcc

131641 tcgatttggt accgctctcg cggcccgcac cgaaacagtg ctttacccct agatgtccag

131701 tcaactgctg cgcctcaacg catttcgggg agaaccagct agctctgggt tcgagtggca

131761 tttcacccct aaccacaact catccgctga ttcttcaaca tcagtcggtt cggacctcca

131821 cttagtttca cccaagcttc atcctggtca tggatagatc acccaggttc gggtccataa

131881 gcagtgacca ttgccctatg aagactcgct ttcgctgcgg ctccggtggg ttcccttaac

131941 caagccactg cctatgagtc gccggctcat tcttcaacag gcacgcggtc agagccatgg

132001 ctcctcccac tgcttgggag cttacggttt catgttctat ttcactcccc gatgggggtt

132061 cttttcaccc ttccctcacg gtactacttc gctatcggtc acccaggagt atttagcctt

132121 gcaaggtggt ccttgctgat tcacacggga ttccacgtgt cccatgctac tcgggtcaga

132181 gcgtaagcta gtgatgcttt cggctactgg actttcgcca tctagggtgc cgcatttatg

132241 ctgcttcgcc tagcagcacg acgcttgtat tgctctccca caaccccggt ttcacggttt

132301 aggctgctcc catttcgctc gccgctacta cgggaatcgc ttttgctttc ttttcctctg

132361 gctactaaga tgtttcagtt cgccaggttg tctcttgcct gcccatggat tcagcagcag

132421 tttgaaaggt tgccctattc gggaatctcc ggatctatgc ttattttcaa ctccccgaag

132481 catttcgtcg attactacgc ccttcctcgt ctctgggtgc ctaggtatcc accgtaagcc

132541 tttcctcgtt tgaacctcgc ccttattgcc cttataaaaa tcttataaat tataaaatca

132601 attataaata aggctatgcc atcctaaggt gctgctaaat ggatggatct tatcaacgtc

132661 catgaatgag aaatcataga tcgaaccgcc gaatcggaaa aattgggtgc tatcatatag

132721 ctttgtatcg gctaagttca tgagttggag ataagcggac tcgaaccgct gacatccgcc

132781 gcagggtaaa ccaccgtctc tcatgccccc gactgattct accatagagg ccaacgatag

132841 acaataaccc cctccgaaca cagcttacaa ctttcatcgt actgtgctct ccaaagagca

132901 actcttctca aaatctcact caaagggtgc tgagttggaa tcccattcta actcaggatt

132961 cttgcggttc cggaggttcc agctacagga gaaccaggaa cggagagccc ccctttttcc

133021 gcccgactct ttggtcttaa gaacgctggt tttaagaatg agtgattgcc cttctccgac

133081 ccttactgcc caacctgaga acggacagct aatgcgttcc acttattgaa cagggttcta

133141 tggtcggtcc gtgacccctg gatgccgaag gcgcccttgg ggtgatctcg tagttcctac

133201 ggggtggaga tgatggggtc ggtccatgga ttttccttcc ttttcttttg ccgcatttcg

133261 ctcaaagggt tgaagggaga tagtgcatca agctgttcgc aagggccaac ttgatcctct

133321 tccccaggga tctcagatga gggaccctgg gggagccgcc gactccaacg accgtccatg

133381 tatgatccat actagatctg accaactgcc catcctacct cctctacgtt cttgacagcc

133441 catctttgtc tcagtagagt ctttcagtgg catgtttcgg tcctcttccc cattacttag

133501 aaaaagtgag ccaccggttc aggtacaaga tactatcatt actgcctgga caattagaca

133561 tccaacccgt aatcgcaacg acccaattgc aagagcggag ctctaccaac tgagctatat

133621 ccccccgagc caagtggagc atgcatgaag tagtcagatg cttcttctat tcttttccct

133681 ggcgcagctg ggccatcctg gacttgaacc agagacctcg cccgtgaagt aaatcatcgc

133741 acctacggtc caaccaattg ggagagaatc aatagattcc ttttcggtag cgattcatcc

133801 ttcccgaacg cagcatacaa ctctccgttg tactgcgctc tccaagtgtg cttgttcgcc

133861 ccttcttcct taccccggca agtctttgtg aaataactct gatgagaaga aaaaagaagg

133921 cgttaagaga ccctcccggc ccaaccctag acactctaag atcctttttc aaacctgctc

133981 ccatttcgaa tcaagagata gataaataga cacatcccat tgcactgatc gggggcgttc

134041 gtagtgactg agggggtcga agaccaagaa gtgagttatt tataccaagc attcttctta

134101 tggctagatc caatctcctg gtccctgcgg aaaggaaaaa gaatttcacg ctcttccttt

134161 cgggaaggga ggattaggga aatgcagctt tctccagacc tccgggaaaa gcatgaaaaa

134221 aacggctcga atggtacgat ccctccgtca ccccagaatg aagggggtga tctcgtagtt

134281 cttggtctgt gaagatgcgt tgttaggtgc tccattttct tttcccattg aggccgaacc

134341 taaacctgtg ctcgagagat agctgtccat acactgataa gggatgtatg gattctcgag

134401 aagagaggag ccgcggtggt ccccccccgg accgcccgga tcccacgagt gaatcgaaag

134461 ttggatctac attggatctc acccgaatcg ccccatcgat cctcctgagg agaggtttgg

134521 tttcaaaccc ctgttcgaac aggaggagta cgccatgcta atgtgccttg gatgatccac

134581 atctcagggt caggcgccga tgagcacatt gaactatcca tgtggctgag agccctcaca

134641 gcccaggcac aacgacgcaa ttatcagggg cgcgctctac cactgagcta atagcccgtc

134701 gtgcgagcct cccacagggg gcccgctatg ccaaaagcga gagaaacccc atccctctct

134761 ttcctttttt cgcccccatg cccccacacg gggggcatgg ggacgtaaca aaggggatcc

134821 tatcaacttg ttccgaccta ggataataag ctcatgggct tggtcttact tcaccgtcga

134881 gaaacgaaag aagacttcct ttaactcaga cgcagctccc ttctttttgt tttgggtgtg

134941 aagcagtggc aaaccaaaat acccaacaag cattagctct ccccgaaaag gaggtgatcc

135001 agccgcacct tccagtacgg ctaccttgtt acgacttcac tccagtcact agccctgcct

135061 tcggcatccc cccccttgcg gttaaggtaa cgacttcggg catggccagc tcccatagtg

135121 tgacgggcgg tgtgtacaag gcccgggaac gaattcaccg ccgtatggct gaccggcgat

135181 tactagcgat tccggcttca tgcaggcgag ttgcagcctg caatccgaac tgaggacggg

135241 tttttggggt tagctcaccc tcgcgggatc gcgacccttt gtcccggcca ttgtagcacg

135301 tgtgtcgccc agggcataag gggcatgatg acttgacgtc atcctcacct tcctccggct

135361 tatcaccggc agtctgttca gggttccaaa ttcaacgatg gcaactaaac acgagggttg

135421 cgctcgttgc gggacttaac ccaacacctt acggcacgag ctgacgacag ccatgcacca

135481 cctgtgtccg cgttcccgaa ggcacccctc tctttcaaga ggattcgcgg catgtcaagc

135541 cctggtaagg ttcttcgctt tgcatcgaat taaaccacat gctccaccgc ttgtgcgggc

135601 ccccgtcaat tcctttgagt ttcattcttg cgaacgtact ccccaggcgg gatacttaac

135661 gcgttagcta cagcactgca cgggtcgata cgcacagcgc ctagtatcca tcgtttacgg

135721 ctaggactac tggggtatct aatcccattc gctcccctag ctttcgtctc tcagtgtcag

135781 tgtcggccca gcagagtgct ttcgccgttg gtgttctttc cgatctctac gcatttcacc

135841 gctccaccgg aaattccctc tgcccctacc gtactccagc ttggtagttt ccaccgcctg

135901 tccagggttg agccctggga tttgacggcg gacttaaaaa gccacctaca gacgctttac

135961 gcccaatcat tccggataac gcttgcatcc tctgtcttac cgcggctgct ggcacagagt

136021 tagccgatgc ttattcccca gataccgtca ttgcttcttc tccgggaaaa gaagttcacg

136081 acccgtgggc cttctacctc cacgcggcat tgctccgtca ggctttcgcc cattgcggaa

136141 aattccccac tgctgcctcc cgtaggagtc tgggccgtgt ctcagtccca gtgtggctga

136201 tcatcctctc ggaccagcta ctgatcatcg ccttggtaag ctattgcctc accaactagc

136261 taatcagacg caagcccctc ctcgggcgga ttcctccttt tgctcctcag cctacggggt

136321 attagcagcc gtttccagct gttgttcccc tcccaagggc aggttcttac gcgttactca

136381 cccgtccgcc actggaaaca ccacttcccg tccgacttgc atgtgttaag catgccgcca

136441 gcgttcatcc tgagccagga tcgaactctc catgagattc atagttgcat tacttatagc

136501 ttccttgttc gtagacaaag cggattcgga attgtctttc attcgaaggc ataacttgta

136561 ttcatgcgct tcatattcgc ccggagttcg ctcccagaaa tatagccatc cctgccccct

136621 cacgtcaatc ccacgagcct cttatccatt ctcattgaat cacggcgggg gggcaaatcc

136681 aaacagaaaa actcacattg ggtttaggga taatcaggct cgaactgatg acttccacca

136741 cgtcaaggtg acactctacc gctgagttat atcccttccc tgcccccatc gagaaataga

136801 actgactaat cctaagtcaa agggtcgaga gactcaacgc cgctattctt gatcttgaac

136861 aacttagagc cgggtcttct tttcgcacta tttcggatat gaaaataatg gtcaaaatag

136921 gattcaattg tcaactgccc ctaggattga ctagcgattc cgaaggaact ggagttacat

136981 cctttttcca tttccattca agagttctta tgtgtttcca cgcccctttg agaccccgaa

137041 aaatggacaa attccttttt tccttttctt aagaacacat acaagattcg tcactacaaa

137101 aaggataatg gtaaccccac cattaactac ttcatttatg aatttcatag taatagaaat

137161 acatgtccta ccgagacaga atttttaatt tgctagtctc ttgcctagca ggcaaagaaa

137221 agatttacct gcgtggaaag gatgattcgt ttggatcgac atgagagtcc aactgcattg

137281 ccagaatcca tgttgtctat ttgaaagagg ttgacctcct tgcttctctc atgtacacgc

137341 cgagcccctt ttctcctcgg cccacagaga taaaatgtag tactggtgcc aacagttcat

137401 cacggaagaa aggactcact aagccgggat cactaactaa tctaaatcac taactaatac

137461 taatctaata taatcgaaaa tactaatata atagaaaaga actgtctttt cagtatactt

137521 tcctcggttc cgttgctatc gcgggcttta cgcaatcgat cggattagat agatatccct

137581 tcaacatagg tcatcgaaag gatctcggag acccaccaaa gtacgaaagc caggatcttt

137641 cagaaaacgg attcctattc gagaagagtg cataactgca tggataagct tacactaacc

137701 cgtcaatttg ggatccaatt cgatattttc ctggggaggt atcgggaagg aattggaatg

137761 gaataagatc gattcataca gaagaaaagg ttctctattg attctgtacc tatgggatag

137821 ggatagagga agaggaaaaa accgaagatt tcacatagta cttttgatcg aaaaatcaat

137881 cagatttatt tcgtaccctt cgttcaatgg tcaaattcta caggatcaaa cctatgggac

137941 ttaaggaatg atataaaaaa aacagaggga aaaagaaatg aatattaact aaaaatgaag

138001 tagaagaacc cagattccaa atgaacaaat tcaaacttga aaaggaaagg acctttctga

138061 ttctcgaaga atgaggggca ggggggttgg tcgagaaaga tctcttgttc ttattatagg

138121 atcgtgattg gatccgcata tgtttggtaa agagaataat cttctccttt gagaataatc

138181 aaaaatggaa agtgttcaat tggaacatga aaacgtgact aaatgggtcc tagttactat

138241 tcgggacaga gtggaagagg ggaggggatt ctcgagcgcg gaaagggatc caatgaattc

138301 gaaagaattg aacgaggagc cgtatgaggt gaaaatctca tgtacggttc tgtagagtgg

138361 cagtaagggt gacttatctg tcaacttttc caccatcacc cccaaaaaac caaactctgc

138421 cttacgtaaa gttgccagag tacgattaac ctctggattt gaaatcactg cttatatacc

138481 cggtattggc cataattcac aagaacattc ttcagtctta gtaagagggg gaagggttaa

138541 ggatttaccc ggtgtaagat atcacattgt tcgaggaacc ctagatgctg tcggagtaaa

138601 ggatcgtcaa caagggcgtt ctagtgcgtt gtagattctt atccaagact tgtatcattt

138661 gatgatgcca tgtgaatcgc tagaaacatg tgaagtgtat ggctaaccca ataacgaaag

138721 tttcgtaagg ggactggagc aggctaccat gagacaaaag atcttctttc taaagagatt

138781 cgattcggaa ctcttatatg tccaaggttc aatattgaaa taatttcaga ggttttccct

138841 gactttgtcc gtgtcaacaa acaattcgaa atacctcgac ttttttagaa caggtccgag

138901 tcaaatagca atgattcgaa gcacttcttt ttacactatt tcggaaaccc aaggactcaa

138961 tcgtatggat atgtaaaata caggatttcc aatcctagca ggaaaggggg ggaaacggat

139021 actcaattta aagtgagtaa acagaattcc atactcgatc tcatagatac atatagaatt

139081 ctgcggaaag ccgtattcga tgaaagtcgt atgtacggct tggagggaga tctttcatat

139141 ctttcgagat ccaccctaca atatggggta aaaaagccaa aataagtgat tttagccctt

139201 ataaaaagaa aactgattct taaacccctt tcacgctcat gtcacgtcga ggtactgcag

139261 aagaaaaaac agcaaaatcc gatccaattt atcgtaatcg attagttaac atgttggtta

139321 accgtattct gaaacacgga aaaaaatcat tggcttatca aattatctat cgagccatga

139381 aaaaaattca acaaaagaca gaaacaaatc cactatctgt tttacgtcaa gcaatacgtg

139441 gagtaactcc cgatatagca gtaaaagcaa gacgtgtagg tggatcgact catcaagttc

139501 ccattgaaat aggatccaca caaggaaaag cacttgccat tcgttggtta ttagcggcat

139561 cccgaaaacg tccgggtcga aatatggctt tcaaattaag ttccgaatta gtggatgctg

139621 ccaaagggag tggcgatgcc atacgcaaaa aggaagagac tcataaaatg gcagaggcaa

139681 atagagcttt tgcacatttt cgttaatcca tgaacaggat ctatacatct cgatcggaaa

139741 agaatcaaga gaaaaagaaa gaatcggaat tgatcgatag atttctcgaa acaaacgaaa

139801 aggaaacgaa agatgaaaca aaaatcatgg atcaactaag ccctctcggg gactttctta

139861 aagaggaacc tcatgtaaat accatggaat aaggtttgat cttgatccta ttccattcca

139921 aaaacggaaa gttcgacaca attgggattt tttttggaaa ttggatgcag ttactaattc

139981 atgatctggc atgtacagaa tgaaaacttc attctcgatt ctacgagaat ttttatgaaa

140041 gcctttcatt tgcttctctt cgatggaagt tttattttcc cagaatgtat cctaattttt

140101 ggcctaattc ttcttctgat gatcgattca acctctgatc aaaaagatat accttggtta

140161 tatttcatct cttcaacaag tttagtaatg agcataacgg ccctattgtt ccgatggaga

140221 gaagaaccta taattagctt ttcgggaaat ttccaaacga acaatttcaa cgaaatcttt

140281 caatttctta ttttactatg ttcaactcta tgtattcctc tatccgtaga gtacattgaa

140341 tgtacagaaa tggctataac agagtttctc ttattcgtat taacagctac tctaggagga

140401 atgtttttat gcggtgctaa cgatttaata actatctttg tagctccaga atgtttcagt

140461 ttatgctcct acctattatc tggatatacc aagaaagatg tacggtctaa tgaggctact

140521 atgaaatatt tactcatggg tggggcaagc tcttctattc tggttcatgg tttctcttgg

140581 ctatatggtt tatccggggg agagaccgag cttcaagaaa tagtgaatgg tcttatcaat

140641 acacaaatgt ataactcccc gggaatttca attgcgctca tattcatcac tgtaggaatt

140701 gggttcaagc tttccccagc cccttctcat caatggactc ctgacgtata cgaaggagtg

140761 cggttcgttc gagaaattcc tacctctcta tctatctctg agatgtttgg atttttcaaa

140821 actccatgga catgcagaag agaaatgcta tccccactcg gaccaagaca gaacttttac

140881 ttgttcaaat aacaattaag gtgaagcagg gtcaggaacg acgaatctct ttatgataaa

140941 cagatccatt ttgcaagttc gttattacgg gtagttccta caaaggatcc gactaatgac

141001 gtatacaata cttgaattct cgatgtagat gctacatagt tggttctcat ccttcagaga

141061 ctacgagtgt aataggagca tccgtcgaca aaaggatcac cctaagatga tcatttcatg

141121 gctattgaga acgaattaaa tcagatggtt ctatttctca atctttctga cttgctccta

141181 cggaaccaag gttgaaaaga ttgaaaaaat aagtcattca caaccactga tgaaggattc

141241 ctcgaaaagt taaggattag taatcttttt tagaaatcga atggattcgg tcttatacat

141301 acgcgaggaa ggtaatcaaa aaagaaagaa aacgagttct tctttctttt atcacttagg

141361 agccgtgtga gatgaaagtc tcatgcacgg ttttgaatga gagaaagaag tgaggaatcc

141421 tcttttcgac tctgactctc ccactccagt cgttgctttt ctttctgtta cttcgaaagt

141481 agctgcttca gcttcagcca ctcgaatttt ggatattcct ttttatttct catcaaacga

141541 atggcatctt cttctggaaa tcctagctat tcttagcatg atattgggaa atatcattgc

141601 tattactcaa acaagcatga aacgtatgct tgcatattcg tccataggtc aaatcggata

141661 tgtaattatt ggaataattg ttggagactc aaatgatgga tatgcaagca tgataactta

141721 tatgctgttc tatatctcca tgaatctagg aacttttgct tgcattgtat tatttggtct

141781 acgtaccgga actgataaca ttcgagatta tgcaggatta tacacgaaag atcctttttt

141841 ggctctctct ttagccctat gtctcttatc cctaggaggt cttcctccac tagcaggttt

141901 tttcggaaaa ctctatttat tctggtgtgg atggcaggca ggcctatatt ccttggtttt

141961 aataggactc cttacaagcg ttgtttctat ctactattat ctaaaaataa tcaagttatt

142021 aatgactgga cgaaaccaag aaatcacccc tcacgtgcga aattatagaa gatctccttt

142081 aagatcaaac aattccgtcg aattgagtat gattgtatgt gtgatagcat ctactatacc

142141 aggaatatca atgaacccga ttattgcaat tgctcaggat accctttttt agcttctagg

142201 gtctatttct tagttcaaga tccctcttac taactggaat caaagaatta gtagatctgt

142261 tccgaccaaa acgggaatgg gctggggtta tgaacttata atctgatgat cgagtcgatt

142321 ccatgattat aagttcattc cataccggac cgggccggaa tagggttata tacattctca

142381 aggggtcatt cgagcgtatc taaatagata ctatgtttac atatgggtcc ctacgccgtt

142441 ccatttagga ttaggaatag gcgtaatcgg acctgctttt tccccatatt tctcgttatt

142501 tttgggaccc tattcacccc tttgagcttc tattgaatcg agaaatcaaa taggtttgat

142561 tggccatctt ttttttttga tatatacata aggcattctc cggataattc aaatcgaagc

142621 aattggatgt ccaactcggg cctatatgac cgatcaatag aaatactaca cccttgtcat

142681 atattccata catcacacta gatagatatc atattcatgg aataggatgc actttcaaga

142741 tgccttggtg gtgaaatggt agacacgcga gactcaaaat ctcgtgctaa agagcgtgga

142801 ggttcgagtc ctcttcaagg cataatattg agaatgctca ttgaataagc aattcaataa

142861 gagagctcgg atcgaatcgg tattgatata ccgacctgca cccgccccag agtatacgat

142921 tcaacaggaa tcacacaagg gtagattaga aacctctggt aaaatgcccg ccagtcaccc

142981 agcggataaa gtacattaca tagtccaggg attgacgact tacccattca gtgactttgg

143041 cactggacgt gcccaaaagg ggtactatcg ggtcgggtca attcaataat agacgcctgt

143101 tggcattcca cccttccttc tcctttcagg gcctatccga aagagaatcc agtacttctt

143161 ggtcgtgaat atctgaactg gttgttcgct gttcaagaat tcttgtttgg gcagttcata

143221 ccatccatac atagtgtttt gatctaagat ttaaattctt ccgtgtttca gcagtaacat

143281 attgttccat ggagctaagg tccaaaatat ggaagaaaga agcgtttcca ccactctacc

143341 acccagtcca ttctgttcca ctgaatccct ctttcatagc cacatatctt tccggctaag

143401 gaatgggaaa tctttctcct gttccatgaa tccaattttc atttcatccg ggaaaagcca

143461 tctttttctt aacaatgtct ttgtcatttg atccaatagc gttccgttag ataggaacag

143521 agttgataaa tactgataac tctcggatag agtattagaa cggaaagatc cattagataa

143581 tgaactattg gttctaagcc atctctgacg attaatcaac aattcgaagt gcttttcttg

143641 tgtattcttg ataaaccagc gtttatatat agatgtagga ggatccattt gggaagtaag

143701 aagccccttt gacatctctt catctgcaaa taattctcga tgtgaaaaca cagagccagg

143761 gggctgatct ttgaatagga aaaagagcgg atccgcaggg tcccaaatga attggcttat

143821 ttgaaaaagg ccttgttctt tggaagatct atctcgtgtc tggtactgca tggttccact

143881 ctgcaagaac tccgaatcat tctcttgaag ctcatcctct tcatcataaa tgatccgctt

143941 gccccgaaat gacctggacc aatagggaaa tcccaattca ttgggccttt cgatacaatc

144001 aaatagaaag gcccaagggc gccatattct aggagcccaa actatgtgat tgaataaatc

144061 ctcctctatc tgttgcgggt cgagggctcc ttcccccttc ccttcttcaa actccgattc

144121 gtatttttca tagagaaatc tctgatcaaa gatagaacaa gatccatttt gcatcatatc

144181 taagggattc ctcggttcgg gccgaagaag caatgtaact cgatcattat caaacggact

144241 gcaatctttt tctgtccgtg aagatcccac cagagcgcct tctacttcta ataggccatg

144301 aactagatcc gaatcattct caacgagtcc ataagaagtg atcccatttt tttcatcgga

144361 tccgggtaga gaccaaagat cttgagcgac cgatccggca gaacaactca aaagataaag

144421 aagtatcgtt aatttcttca tgctcgttcc aagttcgaag taccatttgt acaaataaga

144481 atccccttcg ttacatgatt tcttcttcat atagatagat ataggatcta tggggcaatt

144541 acttagaagt acattttgtg ctacagccct tcctatctga tagaaaagga tcccatgatc

144601 ctgaaccgat cttacctggg atcgcaaatc ccaagtttgt ctatgaagag cggatctaat

144661 tgtcttagtg tctataattg atttcttctg tgtaatacta atcgataggg cctcattggt

144721 aagtgctaca agatctcttg cattggaacc catggttatg gacccgaatc cgttagtatg

144781 gaacattttc ttttccaagt gaaatccctt agtatatgaa agagtgaaaa agtgctttcg

144841 ttgttgtgga ataagaagcc ttcgtatctt aatgcacgta tttaatttat tcggagctat

144901 tagagcggga tccacttttt ggggaatatg agtcgaagca ataacaagaa tatttctagt

144961 ggaacatctt tcagagagat ggttcactaa tagaccgagg gataagtaat tcgactcatt

145021 cacatccaga tcatgaatgt ttggaatcca tattatgcaa ggagatattg cttttgccaa

145081 ttcgaattga agggtgatat aaaatcggtc tatttccggt atcctatcca tagttagcag

145141 ttccagctcc gtatcaaggt cacgatcaat atcgtcacta gcatcaatat cgtcactagc

145201 atcaatatcg tcactagcat caatatcgtc actagcatca atatcgatat catcaaaaag

145261 aaaaccttta ggcttgttat ccaggaactt gttcagaaat accgtaatga aaggaacata

145321 ggagtttgtt gctaggtatt tgaccaaata ggatcgtcca gttcctatag aacctatcac

145381 taaaatcccc ctagaggggg ataaggctaa gcggagcgaa aagggttttc catgagatgg

145441 gaaatgcaaa gtattagtcc cacacgaagt ttgtgaataa gtgattgtct gataatgagc

145501 aaggaatacc cgtctttctg ctaaagagga tgtattgaac tcataattca atagatactt

145561 tttatgaatg tcaactaagt atcgtaagta aattgctccc ggttgttcaa tcatttgata

145621 accagagtca ttctttgata aatgatcact atgagtcaga ctcaatagaa tttgatcaat

145681 ccttttttct gtcgttaagg cggagaactg aaccaagaat tctctttctt catcatcaat

145741 cgaatcactg ttcgcgaccc aggattcgat tttatcatca atccaatccc cattcacgtt

145801 ttttcttttt cttatcaatg aatagatctc tttacttgta tgacttagat gtctcgtatt

145861 tctcgaaaaa gtgattcgat tgatgggatt tggtatgaga tcgatgagat tgatactcaa

145921 atatttcttc ttagaacgta ttgatttgac cccataagcg ggaccaggca tgttgccgcc

145981 agaagcccgt atttcttcta gagaatctcc taattgttcc agagcaacta gaaagagatt

146041 ctttaaccag aaagaattcg gtgcagatgt aggataccta tccagaagtt ttcgtaactc

146101 aatcatagat gatggaatca tcaaagattt gaccttttcg aactctgtct gtaactcact

146161 agaggcccgg gaaacaaaga gaagatgtgt acgaacgaga tatccagcaa caagaagaag

146221 gaaaaggatt gaatagagga actcccgaac atttggcgat ctcagatgtg tcgatggtga

146281 ctcattattt cgatgaatca tttcttcgga cagaagaaga ttatggaaac acttactcga

146341 aatcgcactt atcagattcc attgtggaag acaccatttt ttctgaagaa ttcgccatgc

146401 tataccggat ccatgcataa tatcatgaaa aatggataca aatttttgac tgctacttag

146461 tatcggcaat aggtctgaaa aagtatctaa aaatatcaaa tttagatatt tgtaccctgt

146521 cgaagtaagg aaccatggta tatatgtttg gaatagattc cattttgaga gagttgaaaa

146581 agcaccatct cgttgaaagg ttctatacat ctgccctttc tcaacgcatt tctttagaca

146641 aagactccgc tttttcctct tttcggatgg taaatatttc tcagaacatg gagtgtgaat

146701 caaacccacg tttgaattga gatactgatg caagttcttc ccttctgaat cagatagatt

146761 catatctgaa agaggttgac aataagttct ttcaaaatgg actatttgtc cctctgttag

146821 aggtgttcca aacatgtctg cgatcgagta aatagttcta cgaacgaatg gatcgtgttg

146881 acttggaaaa tggaaagatt tgtacaagtt atacgtttcg tcaccacttt gtggaaaatc

146941 gttaggtatg aatatgttag atacctgtga ctcgattggt gaaatagtat ctctcccccc

147001 aaaagcatgt ttttttttac cgacgcacaa agaaaatatt ttgttgcgaa tgaacaagat

147061 attgaggaat tgtccatacg taaaatcata attattgata cgggcctttt ccacagaaaa

147121 ggggaatctt gtgttacagt agaagcagaa gtgatgtgga ttattcaaga atcgaagtcg

147181 atttgcttta taaaaagaag atatcaatga acttatatga aatggtttca cgggattcag

147241 ccaattgtct tgattgtgga atatcattga gaaataggaa tccgcgttat caaaggattt

147301 cctgcgatta tttctagtat ggaatgagtc aatcatccac tttggtatct tattgaacaa

147361 aaagggcgtt cctccattga tcaagaattt cgattttcgg gaagtatcat gctcatccaa

147421 taagaagggt ttccattttt tcaaatgaac aatttgaaga cctattgatt ctaacaactg

147481 attgcagagt tgatcatttg gacctttcaa ttcatagatg tagatctcgg acctatgaat

147541 ggggatattc ccgaaactca cacagaaaaa aggaagtgag ttcgacaaaa agaaaagcaa

147601 cttggacaaa aaaagaagtg acttggacaa aaagaaacga agtggcttag acaaatcttt

147661 tttgtcgata acctcagacc aatcaatcga atattgatta atacgtaatc gatcgaacac

147721 tatttgaaaa cggctcttct gctcagaaac gaaatgttcc aaatgttcct ggaaattctt

147781 gctcccgttg gaccatttgt atctatatgc atcaggagcc cgattcatgg atctctcggt

147841 tcgagaaatc aagataagag gatcgaacca tttcttctga ctctttttca aattcgataa

147901 atgtgcgttg atcgtatatt tcattatagt tctatgattc agagtatcct ttcctatttg

147961 atccctttga attccatatt cgaagttgcg atcggatcga ttcattaaaa agaatcgatt

148021 ccatacattt cttatgtacc cataggtact atattggatt tgaatcagat ttcggatcaa

148081 tctatattga ttgactgcct ccattatgtt gttgctagca aataccacta tttttggttt

148141 tggatcttcc aaatcattcc cgcaagagat ccggacccat ttttttctga tccttcgaga

148201 aaaagattca ttctcttcat aaaaaatagg aggtagaacc aataaagatt tctttttcga

148261 ttcatccctg gcctcattca agaattgttt ttgatccaat ccgtaggaat caatagaaaa

148321 gggaaatccc ttatgataca ccagatccgg ctcggttatt gatagagtga atagatctgc

148381 catttcttga aatctctctt ctgattcaaa atcgtggtgt aacgtgtatc ctcccctgtt

148441 ccggtcatgg aatagatgaa ataaatcaaa aaatggattt ttgttcaaga atgaaatctt

148501 attggaactg tccatatccg gttcatcctt cagaacccta tcacaccccg gatctgatga

148561 aataggatga attgagacag tattttgtaa atacgtaatt atcttgaata tattaaccag

148621 ttctttattt tccgatcgcc tgggagggac aaaagaaaga tcttgttgtt tcttcaacaa

148681 tttcggatct ctagtggacc tctcagtagg attcgaaccc agatgaagtt ctgaccatct

148741 atcagagaaa aaagaacgaa ccgatcttgt aggattccca agaaattctt ggatttcttc

148801 cggaagcaga tgattaatca tctgcttctc acgttccgtg aatagccggg acattgagga

148861 atatccagaa aggcatttcg ggaatcggtc tgattctatc tctgttcctt ccgtttgaag

148921 aaaggaagga tcccaaagaa tcgatctttc ttttagtggt tgaatctctc tttgattgat

148981 caatgtgtga tattccgaat cctcattact aatggaatcc aaatgatctc tggattgatc

149041 agaagatcct ttcagttggc tagaatccgt tacttgaacg aaactggatc ttgtggaatc

149101 atattgaata tttgacgata cattccgtac ctttctaaaa aaccgatcct tgtttaccaa

149161 ccacacattg tctaaccaaa tccaattctc tctcgatacg ttcctcaaaa aatccgattc

149221 gtgcggattc ttcccccaac taacgaagag atcttggcga aattgccaca tatgaaattg

149281 agcacaattt tgcaaagaaa tagcccgctt gtttctcgag aggagatggg aaacatgctc

149341 aatatcattt gattgaatag ttgacccagc cctttgttgt ttgaagaaac cctccacttc

149401 aattggtatt ttttcacgaa aagcagacat gagataagaa atccagtgtt tcactaagat

149461 ttcgaatagc gggcccgaat tcaagttgat tctatttcgc ctcttcctca gagaaagacg

149521 atcaaacaat tcccaatcat ggtccttgcg gatcggatca tccatataat atacaaaaag

149581 aaactccaga tatttgagat ctttctcttt gaataagatc ccaattccag tgacggtttc

149641 attagatatc ttacaactag aatccctctt ttttccgatc cagttcctcc accaccgcga

149701 accccagtta gattcaggca tgctacactt tttagttatt gggagaaccc aagtactctc

149761 tttcggattc aggaaacaac tctcagagat cttttttcct tttggaagat acaggagcga

149821 aacaatcaac ctattgatat tggaagaccc aaccgattct tccaatgtat catttatggg

149881 tccaatggaa ttcataggta taggaagaag ccccctcaaa tagagatttt ttctttcgac

149941 catatttcga ttgttaatac gatatataag gaccgctact acaaatagta gtacaccctt

150001 gatcgtgaaa tatcgattgc ttgttgaacc ctgtgaattg cgtgaaagta ggatactcaa

150061 aattcggggg tcaaagagtt ttagaaaacg ttcttggtgg aaaaaaatgc gaatgaaaga

150121 ccccactgaa ttgaattggg tccatgaatc taagaaacag tgagaattct tgatctctct

150181 caattcgaaa atccaggatt ggaattgatg tcctttcatt gattcctcct aaattgcatt

150241 gatttatcct aaagatttca tttcaattgg aatttggtta ttcaccatgt acgaggatcc

150301 cccctaagca tccatggctg aatggttaaa gcgcccaact cataattggc gaattcgtag

150361 gttcaattcc tactggatgc acgccaatgg aaccctccaa taagtctatt ggaattggct

150421 ctgtatcaat ggaatctcat catccataca taacgaattg gtgtagtata ttcatatcat

150481 agtatatgaa cagtaagaaa tagcattctt attgagacta gaactcatag ggaagaaatt

150541 cgatttatgg atggaatcaa acatgcagta tttacagaca aaagtattcg gttattgggg

150601 aaaaatcaat atacttctaa tgtcgaatca ggatcaacta ggacagaact aaagcattgg

150661 gtcgaactct tctttggtgt cagggtaata gctatgaata gtcatcgact tccgggaaag

150721 ggtagaagaa tgggacccat tatgggacat acaatgcatt acagacgtat gatcattacg

150781 cttcaaccgg gttattctat tccacctctt agaaagaaaa gaacttaaat caaaatactt

150841 aatagcatgg cgatacattt atacaaaact tctaccccga gcacacgcaa tggaaccgta

150901 gacagtcaag tgaaatccaa tccacgaaat aatttgatct atgggcagca tcattgtggt

150961 aaaggtcgta atgccagagg aatcattacc tcaaggcata gagggggagg tcataagcgt

151021 ctataccgta aaatcgattt tcgacggaat gaaaaagaca tatatggtag aatcgtaacc

151081 atagaatacg accctaatcg aaatgcatac atttgtctca tacactatgg ggatggtgag

151141 aagagatata ttttacatcc cagaggggct ataattggag ataccattgt ttctggtaca

151201 gaagttccga taaaaatggg aaatgcccta cctttgagtg cggtttgaac tattgattta

151261 cgtaattgga agtaaccaat taggtttacg acgaaaccta gaaatcgatc actgatccaa

151321 tttgagtacc tctacaggat agacctcaac agaaaactga agagtaacgg cagcaagtga

151381 ttgagttcag cagttcctca tataaaatta ttgactctag agatatagta atatggagaa

151441 gacaaaatag tttcaagcac cgacagaacc ggaagcgccc cttctttcaa agagaggagg

151501 acgggttatt cacatttcat ttgatggtca gaggcgaatt gaaagctaag cagtgggaat

151561 tctaaagatt ccccggggga aaaatagaga tgtctcctac gttacccata atatgtggaa

151621 gtatcgacgt aatttcatag agtcattcgg tctgaatgct acatgaagaa cataagccag

151681 atgatggaac gggaaaacct aggatgtaga agatcatacc atgagtgatt cggcagattt

151741 ggattcatat agatatccac ccatgcggta cttcattgta ccatatatat aagatccgtc

151801 tgtatagata tcatcatcta catccagaaa gccgtatgct ttggaagaag cttgtacagt

151861 ttgggaaggg gttttgattg atcaaaaaga ggaatctact tcaaccgata tgcccttagg

151921 cacggccata cataacatag aaatcacact tggaaagggt ggacaattag ttagagcagc

151981 gggtgctgta gcgaaactga ttgcaaaaga ggggaaatcg gccacattaa aattaccttc

152041 tggggaggtc cgtttaatat ccaaaaactg ctcagcaaca gtcggacaag tggggaatgt

152101 tggggcgaac cagaaaagtt tgggtagagc cggatccaag cgttggctag gtaagcgtcc

152161 tgtagtaaga ggagtcgtta tgaaccctgt agaccatccc catgggggtg gtgaagggcg

152221 agccccaatt ggtagaaaaa aacccacaac cccttggggt tatcctgcac ttggaagaag

152281 aagtagaaaa aggaataaat atagtgaaaa tttgattgtt cgtcgccgta gtaaataggc

152341 gagaaaatag aatttctttc ttcgtcttta aaaaaaaata ggagtaagct gtgatacgtt

152401 cactaaaaaa aaatcctttt gtagccaatc at

//
